# Identification of a Transferrable Terminator Element That Inhibits Small RNA Production and Improves Transgene Expression Levels

**DOI:** 10.3389/fpls.2022.877793

**Published:** 2022-05-16

**Authors:** Felipe Fenselau de Felippes, Kylie Shand, Peter M. Waterhouse

**Affiliations:** ^1^Centre for Agriculture and the Bioeconomy, Institute for Future Environments, Queensland University of Technology, Brisbane, QLD, Australia; ^2^Australian Research Council (ARC) Centre of Excellence for Plant Success in Nature and Agriculture, Queensland University of Technology, Brisbane, QLD, Australia

**Keywords:** PTGS, small RNAs, silencing, transcription termination, terminator, HSP terminator, gene expression

## Abstract

The role of terminators is more commonly associated with the polyadenylation and 3′ end formation of new transcripts. Recent evidence, however, suggests that this regulatory region can have a dramatic impact on gene expression. Nonetheless, little is known about the molecular mechanisms leading to the improvements associated with terminator usage in plants and the different elements in a plant terminator. Here, we identified an element in the Arabidopsis *HSP18.2* terminator (tHSP) to be essential for the high level of expression seen for transgenes under the regulation of this terminator. Our molecular analyses suggest that this newly identified sequence acts to improve transcription termination, leading to fewer read-through events and decreased amounts of small RNAs originating from the transgene. Besides protecting against silencing, the tHSP-derived sequence positively impacts splicing efficiency, helping to promote gene expression. Moreover, we show that this sequence can be used to generate chimeric terminators with enhanced efficiency, resulting in stronger transgene expression and significantly expanding the availability of efficient terminators that can be part of good expression systems. Thus, our data make an important contribution toward a better understanding of plant terminators, with the identification of a new element that has a direct impact on gene expression, and at the same time, creates new possibilities to modulate gene expression via the manipulation of 3′ regulatory regions.

## Introduction

The formation of the 3′ end is a key part of the biogenesis of mRNAs; during this step, the synthesized transcript is cleaved, defining the borders of the new molecule and the site where polyadenylation will occur. The presence of a poly(A) tail is of great importance, having an impact on the stability of the mRNA, as well as in the export of the transcript from the Nucleus to the cytoplasm, and in its translation into proteins. In addition, factors involved in the maturation of the 3′ end seem to interact with RNA POLYMERASE II to assist with the termination of the transcriptional process, assuring that the polymerase is released from the template at the appropriate moment (Mandel et al., 2007; Kumar et al., 2019).

The terminator is the genic element that contains the information necessary for the proper maturation of the mRNA 3′ end. Several protein complexes recognize and bind to short sequence motifs located in the terminator section of the pre-mRNA. Most of our knowledge about this step comes from mammalian and yeast systems, but it is believed to be conserved among different species, including plants. In mammals, the main protein complex is the cleavage and polyadenylation specificity factor (CPSF), which binds to a highly conserved motif (AAUAAA) known as the poly(A) signal and promotes cleavage of the pre-mRNA 10–30 nt downstream of the binding site. CPSF also functions as a platform for the POLY(A) POLYMERASE (PAP), which is responsible for adding the poly(A) tail at the cleavage site (CS). The other three major complexes involved in 3′ end formation are the cleavage factor I and II (CFIm and CFIIm), and the cleavage stimulation factor (CstF), which contribute to the efficiency and specificity of this process (Mandel et al., 2007; Kumar et al., 2019; Thore and Fribourg, 2019). Despite all similarities, the formation of the 3′ end in plants shows important differences, especially regarding the terminator architecture. In contrast to mammals, the conserved AAUAAA motif is only found in a minority of the poly(A) signals in plant terminators (Loke et al., 2005). In addition, sequences recognized by CFIm, CFIIm, and CstF seem to be absent as well. Instead, three alternative elements are found in plant 3′ regulatory regions: the far upstream element (FUE), the near upstream element (NUE), and the cleavage element (CE). Among the three, the NUE is the one best defined; an A-rich region located near the CS (10–30 nt), analogous to the mammalian poly(A) signal. The CE is a region rich in U that contains the CS, from where polyadenylation occurs. Comparatively, FUEs are not so well-defined. This element is characterized by having U+G-rich regions which could start anywhere from 50 to 160 nt upstream of the CS (Hunt, 2008, 2020; Bernardes and Menossi, 2020).

In addition to its role in the maturation of mRNAs, terminators can also have a dramatic effect on gene expression, especially when transgenes are involved (Ingelbrecht et al., 1989; Richter et al., 2000; Nagaya et al., 2009; Yang et al., 2009; Hirai et al., 2011; Diamos and Mason, 2018; Pérez-González and Caro, 2018; Rosenthal et al., 2018; de Felippes et al., 2020). For reasons still not completely understood, transgenes are particularly susceptible to silencing mediated by small RNAs (sRNAs) (Rajeevkumar et al., 2015; de Felippes and Waterhouse, 2020; Tan et al., 2020). One of the reasons for this susceptibility is the increased production of aberrant mRNAs, such as transcripts missing the poly(A), which are targeted by RNA-DEPENDENT RNA POLYMERASE 6 (RDR6) (Luo and Chen, 2007; Baeg et al., 2017). This enzyme converts single-stranded RNA to double-stranded molecules, which are mainly processed by DICER LIKE 4 (DCL4) to give origin to 21 nt long small interfering RNAs (siRNAs). Together with an ARGONAUTE protein (AGO), siRNAs form the RNA-induced silencing complex (RISC) that will promote silencing of sequence-related transcripts, including the transgene of origin (Bologna and Voinnet, 2014; de Felippes, 2019). Different terminators can have distinct effects on the production of siRNAs from transgenes and their expression levels (de Felippes and Waterhouse, 2020). The 3′ regulatory element of the *HEAT SHOCK PROTEIN 18.2* (*HSP18.2*, AT5G59720) gene from *Arabidopsis thaliana* is one of them. Compared to other terminators, transgenes under the control of the *HSP18.2* 3′ regulatory region show a higher level of expression due to increased mRNA accumulation and reduced susceptibility to silencing (Nagaya et al., 2009; Hirai et al., 2011; Kurokawa et al., 2013; Limkul et al., 2015; Diamos and Mason, 2018; Pérez-González and Caro, 2018; de Felippes et al., 2020). Nonetheless, the mechanisms supporting the superior performance of terminators such as the *HSP18.2*, as well as the *cis*-elements involved are largely unknown. The characterization of such elements would be an important step toward a better understanding of the role terminators have in protecting transcripts from the RNA silencing machinery.

Here, we report the identification of a region in the *HSP18.2* terminator from *A. thaliana* involved with its ability to improve transgene expression and protection against sRNA silencing. We found that elements in the 5′ end of the terminator are essential to support high levels of gene expression, mostly due to the combination of reduced read-through transcription, low predisposition to the generation of transcript-derived sRNAs, and efficient splicing. Moreover, we show that the 5′ end of the *HSP* terminator can be used to create chimeric terminators with improved efficiency, allowing for new strategies to achieve strong and efficient transgene expression.

## Materials and Methods

### Plant Material and Transformation

*Nicotiana benthamiana* plants (Lab ecotype) were grown at 24°C using a 16 h light/8 h dark condition. Agroinfiltration was performed on three to 4 weeks-old plants as previously described (de Felippes and Weigel, 2010) and analysed 3 days post-inoculation. To improve green fluorescent protein (GFP) quantification and phenotypic comparisons within experiments, leaves were infiltrated with a test and a reference construct side-by-side. In each leaf, two spots were inoculated for each construct. Unless stated otherwise, agroinfiltration was performed in the two first tender leaves (the two younger leaves which can be easily infiltrated, corresponding roughly to the 4–5th youngest leaf of the plant), using five different plants. The youngest leaf was used to quantify GFP fluorescence, while the remaining one was sourced for molecular analyses. As a negative control and to allow comparisons to background levels of fluorescence, inoculation using only infiltration buffer was used (referred to as “buffer sample”).

### Reporter Constructs

The pRBCS::GiFiP::tRBCS, pRBCS::GiFiP::tNOS and pRBCS::GiFiP::tHSP reporter constructs have been described previously (de Felippes et al., 2020). For mutations affecting the putative NUEs (1st_NUE_mut, 2nd_NUE_mut, and both_NUE_mut), for the generation of terminators carrying one copy of the tHSP 5′ (tACS2_tHSP_5′, tACS2_tHSP_5′ _short, tACS2_tHSP_5′_long, tNOS_tHSP_5′, tRBCS_tHSP_5′, and tH4_tHSP_5′) and for the characterization of the tHSP 5′ region (tH4_tHSP_5′_min, tH4_tHSP_5′_mut1, tH4_tHSP_5′_mut2, tH4_tHSP_5′_mut3, tH4_tHSP_5′_mut4, and tH4_tHSP_5′_mut5), gBlocks (Integrate DNA Technologies, IDT, Coralville, USA) containing the terminator sequence with the respective modifications were synthesized, PCR amplified and used to replace the original terminator in the pRBCS::GiFiP::tHSP reporter construct using NotI and NcoI restriction sites. The deleted versions of the tHSP (tHSP_3′Δ43, tHSP_5′Δ32, tHSP_5′Δ42, and tHSP_5′Δ104), the tH4 and the tACS2 were amplified by PCR and cloned as described above. To construct the tACS2_2x_tHSP_5′, tNOS_2x_tHSP_5′, tRBCS_2x_tHSP_5′, and tH4_2x_tHSP_5′ reporters, primers containing the tHSP 5′ region flanked by PspOMI sites were allowed to anneal and cloned into the NotI site of the plasmid carrying the respective terminator already incorporating one copy of the tHSP 5′ fragment. All final vectors were introduced into *Agrobacterium tumefaciens* strain GV3101, which were used for agroinfiltration experiments. The sequences of all terminators and primers used for the constructions described above are given in supplementary information (Supplementary Data 1 and Supplementary Table 1).

### GFP Images and Quantification

For the visualization of the GFP fluorescence, a combination of blue light (Dark Reader Hand Lamp HL32T, Clare Chemical Research, Dolores, USA) and an orange filter was used. Images were taken with a Canon EOS550D camera fitted with an orange G filter (Orange G HMC filter, HOYA filters, Tokyo, Japan). Camera settings were adjusted to each experiment as required. For the quantification of GFP fluorescence, tissue was collected using a cork borer (8 mm diameter) for input normalization, frozen in liquid nitrogen and ground using a TissueLyser II (Qiagen, Hilden, Germany). The resulting powder was resuspended in 150 μl of lysis buffer (50 mM Tris-HCl pH 7.5, 150 mM NaCl, 1 mM EDTA and 1% Nonidet P-40). Next, 10 μl of the sample were further diluted in 90 μl of lysis buffer and analysed with the Glomax^®^ Discover (Promega, Madison, USA) using the GFP quantification protocol (excitation–475 nm, emission–500–550 nm). To minimize the impact of variations associated with the agroinfiltration procedure, reads were normalized to the respective reference sample (reference construct inoculated next to the test sample).

### RNA Extraction and sRNA Northern Blots

We have used total RNA obtained with TRIZOL reagent (Thermo Fisher Scientific, Waltham, USA) for all molecular analyses described in this work. Small RNA detection was done by sRNA Northern blots. In short, five or six micrograms of total RNA was separated by electrophoresis in a 17% polyacrylamide gel containing 7 M urea, transferred to a positively charged membrane and UV fixed. Hybridization was performed in the presence of specific probes (Supplementary Table 1) labelled with α-32P-dCTP using either the Rediprime II random priming kit (GE Healthcare, Chicago, USA) or the terminal deoxyribonucleotidyl transferase (Fermentas, Thermo Fisher Scientific, Waltham, USA) for random-primed and oligonucleotide DNA probes, respectively.

### Poly(A) Characterization

The circular RT-PCR (cRT-PCR) protocol for the poly(A) analyses was adapted from Rosenthal et al. (2018). Five micrograms of total RNA had the 5′ cap removed using RppH (New England Biolabs, Ipswich, USA) in a reaction containing NEB Thermopol buffer (New England Biolabs, Ipswich, USA) and RNAseOut (Thermo Fisher Scientific, Waltham, USA) for 1 h at 37°C. To stop the de-capping reaction, RQ1 DNAse stop solution (Promega, Madison, USA) was added and samples were heated for 5 min at 65°C. RNA was purified with the RNeasy^®^ Mini Kit (Qiagen, Hilden, Germany) before circularization, which was performed using T4 RNA ligase 1 (New England Biolabs, Ipswich, USA), 10% PEG 8000, 50 μM ATP and RNAseOut (Thermo Fisher Scientific, Waltham, USA). cDNA was synthesized using the RevertAid first strand cDNA kit (Thermo Fisher Scientific, Madison, USA) with a specific primer located near the 5′end of the GFP. The region containing the poly(A) was PCR amplified using nested primers (30 cycles reaction) and cloned into pGem^®^-T Easy (Promega, Madison, USA). Individual clones were sequenced by Sanger. Sequencing results are provided in Supplementary Material (Data sheet S3 and S4). Annotation of the poly(A) site considered the first nucleotide of the terminator as position “1.” Sequence of all primers used in this experiment can be found in Supplementary Table 1.

### RT-PCR and RT-qPCR Analyses

cDNA for RT-PCR and RT-qPCR was prepared using 1 μg of total RNA and superscript III (Invitrogen, Thermo Fisher Scientific, Waltham, USA) in the presence of oligodT and a specific primer (RT-primer) binding to a region downstream to the terminator. The RT-PCR reaction to study splicing efficiency was performed using a pair of primers flanking either intron 1 or intron 2 in the GiFiP sequence. For the RT-qPCR, the reaction was prepared using GoTaq^®^ qPCR Master Mix (Promega, Madison, USA), 10 ng of cDNA and 10 pmol of either, the gene specific or read-through pair of primers (final volume of 10 μl) and performed on an Mx3000P machine (Stratagene, San Diego, USA); PCR conditions were 95°C for 10 min, followed by 40 cycles of 95°C for 30 s, 60°C for 1 min and 72°C for 1 min. For the melting curve analysis, samples were heated from 65 to 95°C with increments of 0.5°C for 5 s. For each biological replicate, three technical replicates were performed. Primers utilized in these analyses are listed in the supplementary information (Supplementary Table 1).

Materials and methods for experiments only shown in Supplementary Figures can be found in Supplementary Material and Methods. Replicates for the sRNA Northern blots and RT-PCR experiments, as well as the Source data for figures can be found in Supplementary Material (sRNA blots_replicates, Splicing analyses_replicates, Raw_source data for Manuscript figures, respectively).

## Results

### The Second Near Upstream Element Is the Dominant Poly(A) Signal in the HSP Terminator

**FIGURE 1:**
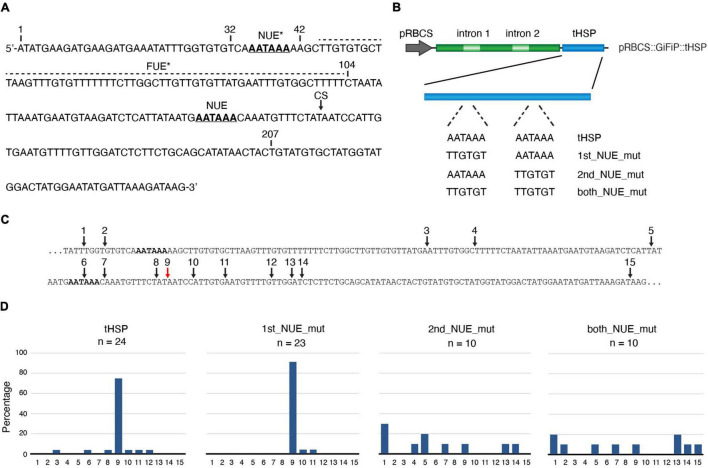
Analysis of the tHSP poly(A) signal. **(A)** The sequence of the *HSP18.2* terminator, with putative poly(A) signals represented in bold/underlined characters. The functional and a potential second near upstream element (NUE and NUE*, respectively) are shown. A region with characteristics compatible with a far upstream element (FUE*) is indicated by a dashed line. The black arrow points to the dominant poly(A) cleavage site (CS). Numbers indicate the nucleotide position relative to the start of the sequence, as well as regions deleted to produce the different constructs shown in Figure 2. **(B)** Scheme representing the pRBCS::GiFiP::tHSP reporter and variations carrying mutations in either one or both NUEs. **(C)** A fragment of the tHSP sequence containing the mapped poly(A) sites indicated by numbered arrows. The expected dominant site is shown with a red arrow. **(D)** Graphs representing the percentage that each of the positions depicted in panel **(C)** was cloned. Poly(A) mapping data are from a single (2nd_NUE_mut and both_NUE_mut) or two independent experiments (tHSP and 1st_NUE_mut).

Aiming to identify factors involved with the ability of certain terminators to deliver more efficient gene expression, we decided to characterize in more detail the *HSP18.2* terminator (tHSP) from *A. thaliana*. We started by identifying in its sequence the most common elements found in plant 3′ regulatory regions (Figure 1A). Previously, the major cleavage site (CS) for the addition of the poly(A) tail in the tHSP has been mapped to be in between nucleotides 158 and 159 (Nagaya et al., 2009; de Felippes et al., 2020). The Near Upstream Element (NUE) is located 13 nt upstream of the CS and consists of the canonical “AAUAAA” sequence found in the majority of animal and yeast poly(A) signals (Figure 1A). The Far Upstream Element (FUE) in plants are characterized to be a sequence rich in “G” and “U” residues, seating 50 nt or more ahead of the CS. A region with such characteristics can be found between nucleotides 45 and 104, which is located 52 nt upstream of the main poly(A) site. Interestingly, a second region containing the canonical “AAUAAA” poly(A) signal motif can be found at the beginning of the terminator sequence (nucleotide 35), raising the possibility of the existence of a second NUE in this regulatory sequence. To investigate the role of these putative NUEs in the formation of the 3′ end of transcripts relying on the tHSP, we have mutated each, or both poly(A) signals in the previously described pRBCS::GiFiP::tHSP reporter (de Felippes et al., 2020), creating the following variants: 1st_NUE_mut, 2nd_NUE_mut and both_NUE_mut (Figure 1B). Using circular RT-PCR (cRT-PCR), we have mapped the location of the poly(A) tail in transcripts originating from each of the constructs after agroinfiltration in *N. benthamiana* leaves (Figures 1C,D). As expected, in most of the cases (75%, 18 out of 24 clones) the poly(A) in tHSP transcripts was located at position 159 from the beginning of the terminator sequence, in agreement with previous reports (Nagaya et al., 2009; de Felippes et al., 2020). Mutations in the first NUE (1st_NUE_mut construct) resulted in a similar pattern, with the majority of transcripts (91%, 21 out of 23) having the poly(A) mapped at the wild-type dominant location. In contrast, mutations in the second, or both NUEs (2nd_NUE_mut and both_NUE_mut constructs) result in no clear CS, with poly(A) sites spread throughout the terminator sequence (Figures 1C,D). These results confirm that the second AAUAAA is the poly(A) signal (NUE) in the tHSP.

### The 5′ Region of the tHSP Is Required for a Strong GFP Expression

**FIGURE 2:**
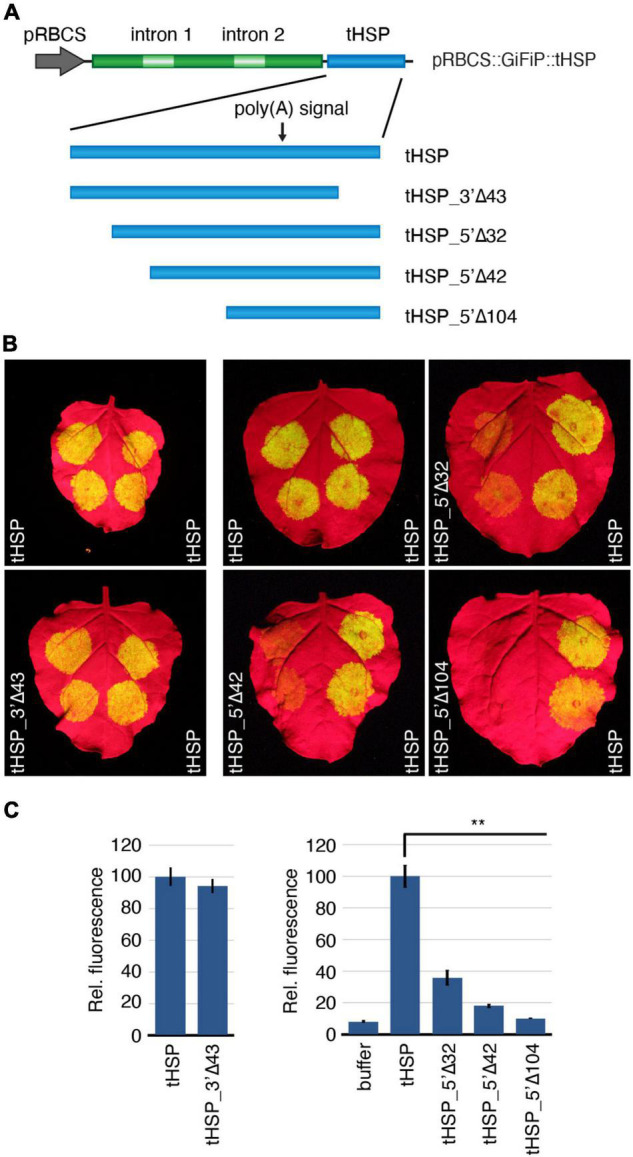
Deletion analysis to assess the effect of different regions of the tHSP on the terminator activity. **(A)** Representation of the pRBCS::GiFiP reporter using the wild-type tHSP or different versions containing deletions of specific regions. **(B)** *Nicotiana benthamiana* leaves agroinfiltrated with the different constructs. Leaves were infiltrated with the test sample (left side) and the wild-type terminator (right side) as reference. Infiltrations for 5′ and 3′ end mutants were carried out in independent experiments and hence, shown separately. **(C)** GFP fluorescence for deletions affecting the 5′ end of the terminator was calculated from five infiltration spots (originating from four leaves, each from a different plant). Values referent to the 3′ end mutations are from 10 infiltration spots coming from five leaves, each leaf from an independent plant. All values are relative to the wild-type tHSP. Standard error bars are given. Statistically significant differences between the wild-type and each of the mutants (Mann-Whitney *U* test) are indicated by “**” (*p* ≤ 0.01).

In the next step of our effort to characterize the tHSP and identify regions important for the high level of expression supported by this regulatory element, we have deleted different regions of the terminator in the pRBCS::GiFiP::tHSP reporter and analysed the GFP expression in agroinfiltrated *N. benthamiana* leaves (Figure 2). We started by removing the first 32 nt of the terminator, which contains most of the region anterior to the first putative NUE (tHSP_5′Δ32). We have also generated two extra versions of the reporter, which expand the deleted region to include the putative first NUE and also the FUE that most likely supports the second, dominant NUE (tHSP_5′Δ42 and tHSP_5′Δ104, respectively; Figures 1A, 2A). In addition, we have generated a reporter carrying a deletion of the last 43 nt of tHSP (tHSP_3′Δ43; Figures 1A, 2A). This last construct was specifically designed to test the role of a hairpin-like structure in the tHSP with the potential to be a substrate for DCL4 (Supplementary Figure 1). DCL4 was previously identified to be involved in the susceptibility of transgenes to silencing (de Felippes et al., 2020) and it has also been shown to be important for the proper transcription termination of *FCA*; a process that involves the DCL4-mediated cleavage of the nascent transcript downstream of the CS (Liu et al., 2012).

We did not detect significant changes in GFP expression when the 3′ end of the tHSP (tHSP_3′Δ43) was removed, indicating that this region has no elements involved in the terminator efficiency. In contrast, constructs lacking 5′ regions of the tHSP showed a significant reduction in expression of the reporter gene (Figures 2B,C). Deletion of the first 32 nt of the terminator (tHSP_5′ Δ32) was already sufficient to decrease the reporter expression by more than half. GFP fluorescence was further reduced in tHSP_5′Δ42, eventually reaching values close to background levels in the tHSP_5′Δ104 version.

### Generation of Chimeric Terminators With Improved Efficiency Using the tHSP 5′ Region

**FIGURE 3:**
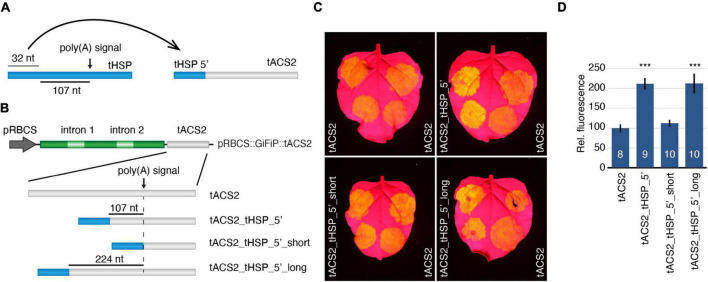
Investigation of the capacity of the tHSP 5′ end to improve other terminators’ efficiency. **(A)** Schematic of the strategy used to test the ability of the tHSP 5′ fragment in improving the activity of weaker terminators. **(B)** Graphic representation of the different reporter constructs generated to test the impact of the 5′ region of the tHSP on the activity of the *ACS2* terminator. **(C)** Agroinfiltrated leaves representing the GFP expression obtained with different terminator constructs (left side) compared to the wild-type reference (tACS2, right side). **(D)** Relative GFP fluorescence of at least eight infiltration spots originating from five leaves, each leaf from a different plant (the exact number of independent measurements for each construct is indicated by the numbers in the graph columns). The standard error is shown. Statistically significant differences (*p* ≤ 0.001; Mann-Whitney *U* test) are indicated by a “***”.

Our results so far indicate that the initial sequence of the tHSP (consisting of the first 32 nt of this element) have features that are necessary for the stronger gene expression obtained when this terminator is used. If this assumption is correct, we hypothesized that other 3′ regulatory regions could be “improved” when the tHSP 5′ sequence is added to these terminators (Figure 3A). To test this idea, we have modified the *ACS2* (1-aminocyclopropane-1-carboxylate synthase 2; At1g01480) terminator (tACS2) from *A. thaliana* to include the tHSP initial 32 nt. We have chosen this particular terminator for its well-defined poly(A) site, an NUE that also consists of the canonical “AAUAAA” motif, and for supporting lower levels of gene expression when compared to the tHSP (Nagaya et al., 2009). Three versions of the hybrid terminator were created (Figure 3B). In the first one, the tHSP 5′ sequence was used to replace the entire region of the tACS2 located more than 107 nt upstream of the poly(A) signal (tACS2_tHSP 5′), mimicking the layout found in the “donor” terminator. In the other two constructs, the 32 nt sequence from tHSP was placed at different distances from the tACS2 NUE, either directly in front of it or 224 nt from the poly(A) signal (tACS2_tHSP 5′_short and tACS2_tHSP_5′_long, respectively). We have then generated pRBCS::GiFiP reporters carrying each one of these hybrid versions in addition to the wild-type tACS2 (Figure 3B). The efficiency of the different terminators was analysed by measuring the levels of GFP expression in *N. benthamiana* agroinfiltrated leaves. Compared to the tACS2, constructs carrying hybrid terminators containing the 5′ region of the tHSP located at least 107 nt to the NUE (tACS2_tHSP_5′ and tACS2_tHSP_5′_long) showed an increase in GFP expression levels. On the other hand, placing the tHSP 5′ sequence directly in front of the terminator NUE (tACS2_tHSP_5′_short) did not have an impact on the intensity of GFP detected (Figures 3C,D). To exclude the possibility that the improvement seen in the chimeric constructs was due to the deletion of a suppressor region in the tACS2 or was not caused specifically by the tHSP 5′ sequence, we have tested three new constructs carrying the following terminators: tACS2_5′_del, which is missing the whole region located 107 nt upstream of the NUE; and tACS2_tNOS_5′ and tACS2_tRBCS_5′, which are similar to tACS2_tHSP_5′, with the difference that the 32 nt sequence was originated from the initial region of the terminators of the *NOPALINE SYNTHASE* gene (*NOS*) from *Agrobacteria tumefaciens* or the RuBisCO small subunit from *A. thaliana* (*RBCS1A*, AT1G67090; the respective terminators will be referred to as tNOS and tRBCS). In all three cases, GFP expression was lower than in the tACS2 (Supplementary Figure 2). These results corroborate our hypothesis that the first 32 nt of the tHSP has important elements, which are, at least in part, responsible for the outstanding efficiency of this regulatory element. The requirement of a minimal distance between the 5′ region of tHSP and the tACS2 NUE for the rise in GFP expression most likely reflects the need for other features, such as an FUE. In line with this idea, in our initial characterization of the tHSP, GFP expression was further reduced when the FUE before the second poly(A) signal was included in the deleted fragment (Figures 2B,C). Similarly, the specific deletion of that same FUE also resulted in decreased GFP signal (Supplementary Figure 3).

**FIGURE 4:**
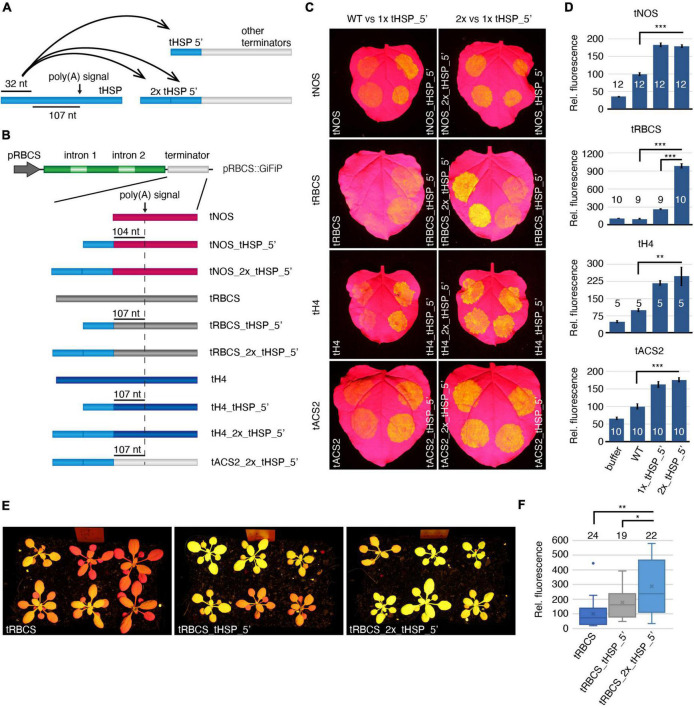
Impact of the tHSP 5′ fragment on the activity of different terminators. Illustration of the different approaches **(A)** and constructs **(B)** utilized to test the impact of the tHSP 5′ end on different terminators. **(C)** *Nicotiana benthamiana* agroinfiltrated leaves representing the improvement of GFP expression as a result of the incorporation of one or two copies of the tHSP 5′ region into the sequence of different terminators. **(D)** The relative fluorescence of the reporter under the control of wild-type or chimeric terminators is shown. Values are from independent infiltration spots collected from four (tH4), five (tACS2 and tRBCS), or six leaves (tNOS), each one from a different plant. The exact number of replicates analyzed is indicated for each line (numbers shown in the graph columns). Statistically significant differences between wild-type and chimeric terminators, and between regulatory elements carrying one or two copies of the tHSP 5′ fragment are indicated with “**” or “***” for a *p*-value of *p* ≤ 0.01 and *p* ≤ 0.001, respectively (Mann-Whitney *U* test). **(E)** GFP fluorescence in 21-days old T1 plants representing a population carrying the wild-type tRBCS compared to chimeric versions containing one or two copies of the tHSP 5′ region (tRBCS_tHSP_5′ and tRBCS_2x_tHSP_5′, respectively). **(F)** GFP fluorescence relative to the wild-type tRBCS. The number of plants analyzed is indicated for each group. Statistically significant differences (Welsh *t*-test; two-sample unequal variance; two-tailed distribution) between wild type and chimeric terminators (“**”; *p* ≤ 0.01), and between regulatory elements carrying one or two copies of the tHSP 5′ fragment are given (“*”; *p* ≤ 0.05).

We next tested if the enhancing features of the tHSP initial 32 nt were limited to the tACS2. The same design applied to tACS2_tHSP_5′ was reproduced in three additional terminators: tNOS, tRBCS and tH4 (*histone H4* from *A. thaliana*; At5g59690; Figures 4A,B). The two first regulatory elements were chosen due to their poor efficiency in driving the expression of the GiFiP reporter in *N. benthamiana* agroinfiltrated leaves (de Felippes et al., 2020), while the latter has been previously characterized and showed weaker efficiency when compared to the tHSP (Nagaya et al., 2009). In addition, we assessed whether the effects of the tHSP 5′ fragment were accumulative. To do so, chimeric terminators were generated containing two copies in tandem of the tHSP 32 nt sequence, resulting in the following versions: tACS2_2x_tHSP_5′, tNOS2_2x_tHSP_5′, tRBCS_2x_tHSP_5′, tH4_2x_tHSP_5′ (Figures 4A,B). Supporting the notion that the tHSP 5′ fragment has some specific feature that promotes gene expression, all terminators carrying that region showed an increase in the amount of GFP when compared to their respective wild-type versions (Figures 4C,D). Nonetheless, increasing the number of tHSP 5′ fragments in a hybrid terminator does not seem to further improve its efficiency. The only exception was the tRBCS_2x_tHSP_5′, which presented a nine- and a three times fold increment in GFP expression when compared to tRBCS and tRBCS_tHSP_5′, respectively (Figures 4C,D). Interestingly, the tRBCS is the only terminator for which GFP expression could not be detected above background level when its wild-type version was used. Thus, it is possible that an additive effect of the tHSP 5′ fragment only occurs in such extreme cases.

Given the known differences between transient and stable expression systems, we also tested the efficiency of chimeric terminators in stably transformed *A. thaliana* (Figures 4E,F and Supplementary Figure 4). To minimise bias caused by variations in expression associated with differences in the number of insertions and genomic location of the transgene, we have analysed a population of T1 plants instead of a few isolated lines. As previously noticed (de Felippes et al., 2020), tRBCS seems to perform better in *A. thaliana* than in *N. benthamiana* leaves, with most plants showing some degree of GFP fluorescence (Figure 4E). Analysis of at least 19 independent lines showed an increase in GFP fluorescence of around 77% in plants expressing the chimeric tRBCS (tRBCS_tHSP_5′) compared to the original terminator (tRBCS, Figure 4F). Similarly to what was seen in the transient system, transgene expression levels were further increased in plants carrying the tRBCS_2x_tHSP_5′ terminator. Thus, the use of chimeric terminators carrying the tHSP 5′ region is an efficient way to improve transgene expression in transient and stable systems.

### The Enhancing Features of the tHSP 5′ Fragment Are Contained in an 18 nt Long Sequence

**FIGURE 5:**
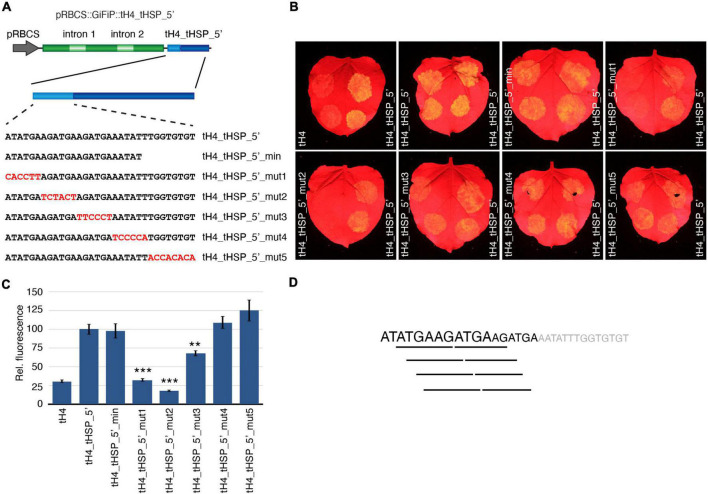
Characterization of tHSP 5′ region. **(A)** Constructs designed to identify sequence elements contributing to the positive effect exhibited by the tHSP 5′ end. Representative GFP phenotypes **(B)** and the relative levels of fluorescence **(C)** of 10 infiltration spots are given (replicates collected from five leaves, one leaf per plant). Mutations resulting in statistically significant changes in fluorescence when compared to tH4_tHSP_5′ are marked with a “**” or “***” (*p* ≤ 0.01 and *p* ≤ 0.001, respectively; Mann-Whitney *U* test). **(D)** The tHSP 5′ region. Sequences contributing to the improvement of gene expression are shown in black, with the degree of impact reflected in the size of the characters. Lines underneath the sequence highlight possible motifs presented in this fragment.

Prompted by the results with the chimeric terminators, we decided to further characterize the tHSP 5′ region to identify elements that contribute to its ability to improve gene expression. Using the tH4_tHSP_5′ terminator as a reference, we have first tested if the transferred sequence could be further reduced by adding only the first 23 nt of the tHSP to the tH4 (tH4_tHSP_5′_min). In addition, we have tested the importance of different regions in the 32 nt fragment by sequentially mutating blocks of 6–8 nt in the tHSP 5′ portion (constructs tH4_tHSP_5′_mut1 to tH4_tHSP_5′_mut5; Figure 5A) and checking for GFP expression. Mutations in either of the first two blocks (tH4_tHSP_5′_mut1 and tH4_tHSP_5′_mut2) were sufficient to abolish any positive effect of the tHSP 5′ fragment on the reporter expression (Figures 5B,C). GFP levels were also reduced in tH4_tHSP_5′_mut3, but to a lower extent. On the other hand, changes to the terminal part of the tHSP segment (tH4_tHSP_5′_mut4 and tH4_tHSP_5′_mut5), including using a shorter section of this element (tH4_tHSP_5′_min), still led to improvement in the tH4 efficiency. Therefore, we conclude that the first 18 nt of the tHSP carry some element(s) that contributes to the high efficiency of this terminator and, when transferred to weaker 3′ regulatory sequences, promotes an increment in gene expression. Curiously, in this region, a six nt long repeat can be found with four possible combinations (ATGAAG, TGAAGA, GAAGAT, or AAGATG; Figure 5D). To investigate if these sequences or any other part of the 18 nt fragment could be recognized by RNA binding proteins, we have initially performed a search against *A. thaliana* motifs using the MEME Suite tool (see Supplementary Material). However, we did not retrieve any significant match, probably due to the low representation of this species in the database (Ray et al., 2013). Therefore, we have expanded our search to include databases from other organisms, mainly *Homo sapiens* and *Drosophila melanogaster*. With this new approach, we found eight proteins for which the tHSP sequence showed some significant resemblance with their binding motifs (Supplementary Figure 5). Interestingly, many of these proteins were involved in splicing. Nonetheless, the implications of this finding are still unclear and will require further effort to elucidate.

### The Effect of the 5′ Region of the HSP Terminator on the Poly(A) Tail Formation

**FIGURE 6:**
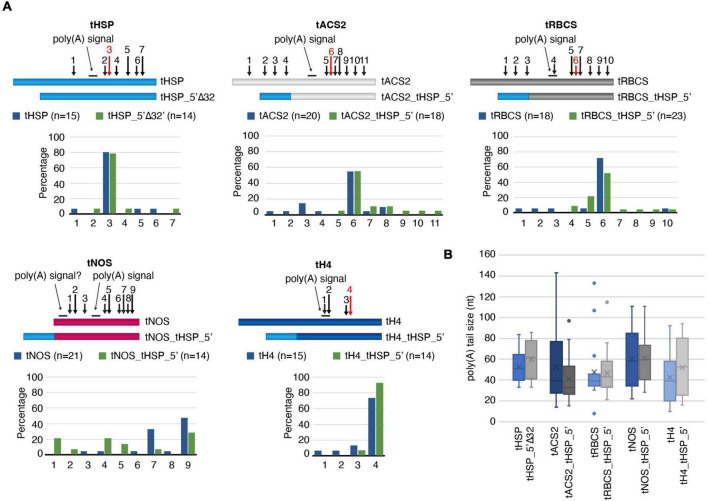
Analysis of the poly(A) tail formation. **(A)** Mapping of the poly(A) sites in wild-type and mutant terminators. For each regulatory sequence, a graphic representation of the different terminators with the identified cleavage positions (numbered arrows) and the percentage of these sites are given. The dominant poly(A) sites (as expected from previous publications) are indicated by red arrows. The poly(A) signals are shown as a line above the illustration. **(B)** The poly(A) tail average size with standard error is presented. The number of clones analyzed is the same as indicated in panel A, except for tHSP (14 clones), tHSP_5′Δ32 (13 clones) and tRBCS (17 clones). All data originated from a single experiment.

Since one of the main roles of terminators is in the formation of the 3′ end of nascent transcripts, we investigated whether the positive effects of the tHSP 5′ region on gene expression could be due to changes in the poly(A) tail. Using cRT-PCR, we have compared the poly(A) sites in transcripts under the regulatory control of the tHSP versus the version missing the first 32 nt (tHSP_5′Δ32). The same comparison was also done between “improved” terminators and their wild-type forms (Figure 4B). As expected, the majority of transcripts (12 out of 15; 80%) originating from the construct using the tHSP had the poly(A) inserted at position 159 from the beginning of the terminator (Figure 6A and Supplementary Figure 6). The same pattern can be seen for tHSP_5′Δ32, where 78.6% of clones (11 out of 14) could be mapped to the predicted main CS. Similarly, the location and frequency of the main poly(A) site in tACS2, tRBCS and tH4 does not show considerable changes when compared to their “improved” versions (tACS2_tHSP_5′, tRBCS_tHSP_5′ and tH4_tHSP_5′, respectively; Figure 6A and Supplementary Figure 6). The only case where the poly(A) site pattern seems to significantly differ between wild-type and “improved” terminators was with tNOS (Figure 6A and Supplementary Figure 6). While in tNOS samples the CS was mainly detected in the same area (positions 8–10), in tNOS_tHSP_5′ three major locations for the poly(A) can be found (positions 2–3, 5–6, and 8–10). Because we could previously map most poly(A) sites in tNOS at regions represented by positions 5–6 and 8–10 (de Felippes et al., 2020), we believe that the enrichment in the former location in the “improved” terminator is rather fortuitous. On the other hand, the addition of the tHSP 5′ region in the *NOS* terminator seems to activate a cryptic NUE starting at position 20 when considering the wild-type sequence. Interestingly, this cryptic poly(A) signal has been shown before to be activated when the CaMV 3′ regulatory sequence is inserted in front of the *NOS* terminator (Sanfacon et al., 1991). Besides mapping the CS locations, we have also checked for changes in the length of the poly(A) tail. Given that cRT-PCR is based on the sequencing of a circular molecule, this technique can also be used to directly estimate the size of the poly(A). We did not detect any statistically significant variation in the length of the poly(A) between wild-type and “improved” terminators with the analysis of a small number of samples (Figure 6B). Taken all together, we cannot exclude that, in specific cases (such as with the activation of the cryptic NUE in tNOS), the presence of the tHSP 5′ region has an impact on the poly(A) formation. However, our data suggests that another mechanism is behind the positive effect observed when this region is part of the terminator sequence.

### The tHSP 5′ Region Dramatically Reduces the Occurrence of Transcriptional Read-Throughs and the Generation of sRNAs

**FIGURE 7:**
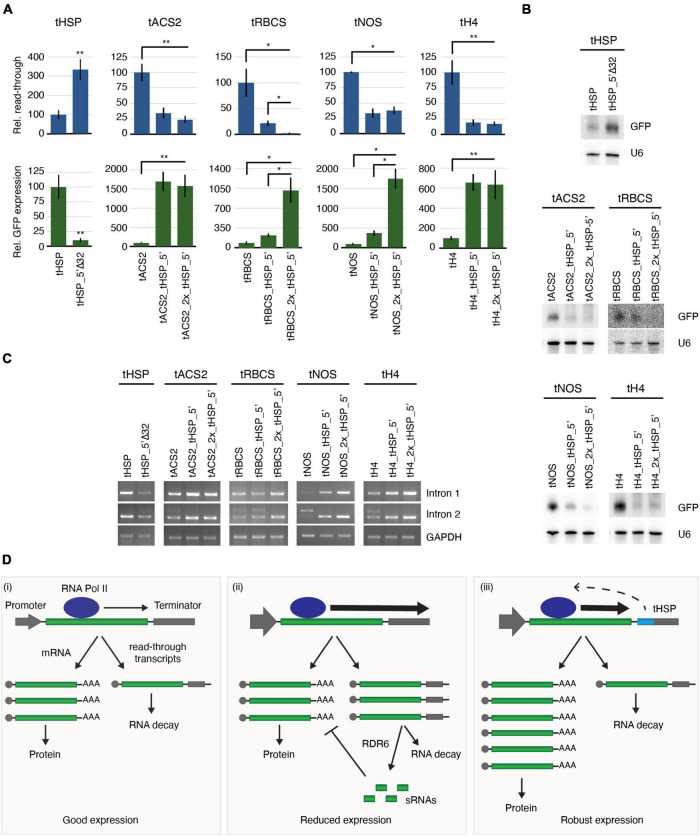
The role of the tHSP 5′ fragment on transcription termination, protection against sRNA production and splicing. **(A)** RT-qPCR analysis to detect read-through and *GiFiP* expression levels. All values are normalized to *GAPDH* (*GLYCERALDEHYDE 3-PHOSPHATE DEHYDROGENASE*) and are relative to the wild-type regulatory sequence. Read-through refers to the detection of a region of the T-DNA downstream of the terminator sequence. For the RT-qPCR analyses, three (tRBCS), four (tNOS), or six (tHSP, tACS2 and tH4) biological replicates were used. Each replicate is an infiltration spot originating from an independent plant. Standard error bars were calculated for the biological replicates. Statistically significant differences observed between the original and modified terminators, or between constructs carrying one versus two fragments of the tHSP (Mann-Whitney *U* test) are indicated by “*” (*p* ≤ 0.05) or “**” (*p* ≤ 0.01). **(B)** Northern blot to detect sRNAs derived from the *GiFiP* reporter. U6 was used as the RNA loading control. **(C)** RT-PCR to test splicing efficiency using primers flanking each of the introns in the *GiFiP* gene. GAPDH was used as the internal control. **(D)** Proposed model explaining how terminators carrying elements similar to the one found in the tHSP can contribute to a robust gene expression. (i) In a balanced situation, the RNA POLYMERASE II (RNA Pol II) produces only a small fraction of read-through transcripts, which are readily degraded by the RNA decay pathway. (ii) In cases where the terminator efficiency is not sufficient to cope with high rates of transcription (for instance, when strong promoters are used), the occurrence of read-through transcription increases and the resulting molecules overcome the RNA decay pathway, leading to the production of RDR6-dependent sRNAs and gene silencing. (iii) Terminators containing elements like the one found in the tHSP sequence, contribute to improving transcription termination, which results in low levels of aberrant transcripts being produced. As a consequence, sRNA generation is inhibited, allowing for a robust gene expression.

The formation of the mRNA 3′ end also plays a critical role in the termination of the transcriptional process mediated by the RNA polymerase II (Zorio and Bentley, 2004; Mandel et al., 2007). We have therefore analysed the levels of transcriptional read-through and its correlation with the presence or not of the tHSP 5′ region in the terminator sequence by using RT-qPCR. Compared to its wild-type version, tHSP_5′Δ32 shows 3-fold increase in the amount of read-through detected and decreased expression of the reporter. In contrast, all “improved” terminators, which have the tHSP 5′ region, had a dramatic reduction in read-through followed by a sharp increase in GFP expression (Figure 7A and Supplementary Figure 7). Interestingly, the only construct where two copies of the initial 32 nt of the tHSP further decreased the levels of read-through was tRBCS_2x_tHSP_5′, which was also the only case where doubling this element resulted in even greater levels of GFP fluorescence (Figures 4C,D).

It is known that aberrant transcripts, like the ones resulting from transcriptional read-through, can be potent inducers of RDR6-dependent sRNAs generation (de Felippes and Waterhouse, 2020). Therefore, our next step was to compare the levels of sRNAs originating from GFP under the control of the different terminators. As expected, a strong positive correlation can be observed between the levels of read-through and the presence of sRNAs matching the reporter (Figure 7B). Without exception, all terminators containing the tHSP 5′ fragment presented lower levels of sRNAs when compared to constructs missing this region. Curiously, tRBCS_2x_tHSP_5′ again displayed a clear difference to tRBCS_tHSP_5′, showing decreased levels of sRNAs against GFP. Thus, we conclude that the newly identified element in the tHSP 5′ fragment promotes higher levels of gene expression by reducing transcriptional read-through and consequently, limiting the generation of aberrant transcripts that could become a template for the production of sRNA targeting the reporter gene.

### Splicing Is Affected by the Presence of the tHSP 5′ Fragment

We have previously shown that different terminators can interfere with splicing and consequently affect gene expression (de Felippes et al., 2020). This phenomenon is more prominent in the processing of the second intron in the GiFiP reporter and seems to be correlated to the presence of sRNAs. Since we could detect increasing amounts of sRNA in constructs missing the initial 32 nt of the tHSP, we investigated whether the presence of this region had any effect on the splicing of the GFP transcript. Indeed, constructs lacking the tHSP fragment accumulated the non-spliced form of the GFP reporter, especially when the second intron was analysed (Figure 7C and Supplementary Figure 7). In contrast, transcripts originating from constructs carrying the wild-type tHSP or the chimeric terminators showed little or no signs of intron retention. Once more, the only case where two copies of the tHSP fragment in the same chimeric terminator had an impact on the analysed trait was with tRBCS_2x_tHSP_5′, further corroborating the idea that GFP expression is directly affected by read-throughs and the levels of sRNAs. Therefore, these results indicate that the variation in GFP expression seen between wild-type and “improved” terminators can also be partially credited to differences in splicing efficiency.

## Discussion

In addition to their essential role in transcription termination and mRNA biogenesis, an increasing amount of evidence shows that terminators can dramatically impact the levels of gene expression (Ingelbrecht et al., 1989; Richter et al., 2000; Nagaya et al., 2009; Yang et al., 2009; Hirai et al., 2011; Diamos and Mason, 2018; Pérez-González and Caro, 2018; Rosenthal et al., 2018; de Felippes et al., 2020). Yet, relatively little is known about the elements and mechanisms associated with the terminator function in plants. The *A. thaliana HSP18.2* 3′ regulatory region is an example of a plant terminator that can promote strong gene expression, increase transcript stability and decrease the mRNA exposure to the sRNA silencing pathway (Nagaya et al., 2009; Hirai et al., 2011; Kurokawa et al., 2013; Limkul et al., 2015; Diamos and Mason, 2018; Pérez-González and Caro, 2018; de Felippes et al., 2020). The lack of information about which elements in the tHSP are responsible for its efficiency prompted us to investigate this terminator in more detail. We identified a region located in the initial 18 nt of the terminator sequence to be involved with its capacity to sustain a high level of gene expression. This new element contributes to an efficient transcription termination and lower incidence of read-through transcripts, hence preventing gene silencing caused by sRNAs derived from aberrant mRNAs. Moreover, we show that this region can be used to create chimeric terminators, more efficient than their original counterparts, providing a new strategy to improve transgene expression and dramatically expanding the availability of effective 3′ regulatory sequences.

The cross-talk between transcription termination and mRNA 3′ end formation is well-known. The C-terminal domain of RNA POLYMERASE II is involved in the coordination of transcription with the major pre-mRNA processing steps, including 3′ end formation. Indeed, the presence of a poly(A) site was shown to be required for efficient termination of transcription in mammals and yeast (Zorio and Bentley, 2004; Mandel et al., 2007). In plants, the link between the end of transcription and polyadenylation has also been documented. Plants carrying mutations in genes involved in the poly(A) formation were shown to have increased levels of transcriptional read-through (Herr et al., 2006; de Felippes et al., 2020). Our data strongly indicates that the 5′ end of the tHSP has elements important for efficient transcription termination, as indicated by the low levels of read-through detected when terminators carrying this sequence were used (Figure 7A). Given the lack of self-complementary regions that could result in important secondary structures and the presence of sequences with the potential to be RNA binding protein domains (Figure 5D and Supplementary Figure 5), we hypothesise that the tHSP 5′ end serves as a platform for the interaction of proteins with the ability to improve transcription termination efficiency.

One of the consequences of the presence of read-through mRNAs is the generation of RDR6-dependent sRNAs. Luo and Chen (2007) showed that molecules originating from read-through events were targeted by RDR6, most likely due to the aberrant characteristic of these transcripts, which often lack a poly(A) tail. Indeed, the presence of a poly(A) tail seems to inhibit the activity of RDR6 (Baeg et al., 2017). Furthermore, the strategy of improving termination with the use of double terminators in transgenic constructs was shown to be an efficient way to avoid RDR6 from utilizing such mRNAs as substrate, protecting them from becoming silenced (Luo and Chen, 2007; Nicholson and Srivastava, 2009; Beyene et al., 2011; Yamamoto et al., 2018). In agreement with the above scenario, we have detected a clear association between the levels of read-through transcripts and sRNAs originating from the reporter gene. The levels of these two molecules were also inversely proportional to the intensity of gene expression, which was stronger for constructs carrying the tHSP 5′ fragment (Figures 7A,B). Thus, the variations in gene expression observed in this work are most likely a consequence of the reduced levels of aberrant transcripts being generated when the tHSP fragment is present. Interestingly, we did not detect in conditions missing the tHSP 5′ region an increase in mRNAs with shorter or missing the poly(A) (Figure 6 and Supplementary Figure 6). It is likely that such transcripts are readily degraded or processed by RDR6, making them difficult to be detected. Another factor that needs to be taken into consideration is the sensitivity of PCR-based methods, like the one used here for the poly(A) analyses. Read-throughs most likely affect only a small percentage of the mRNAs and since PCR is biased toward the abundance of the template, likely, alterations in the poly(A) length would only be detected by large scale experiments. It is also possible that limitations in sample sizes and sequencing contributed for the lack of differences observed.

Another molecular phenotype linked to the tHSP 5′ fragment refers to the splicing of introns in the *GiFiP* reporter. We noticed a reduction in intron retention in lines using the wild-type tHSP or the “improved” terminators. Given the known connection between transcription and splicing (Zorio and Bentley, 2004), it is conceivable that induction of transcription termination (by elements in the tHSP 5′ terminus) could affect the processing of introns. For instance, changes in the RNA Polymerase elongation rate have been shown to affect splicing (Howe et al., 2003; Mata et al., 2003). Thus, it is also possible that, by inhibiting read-throughs and stimulating transcriptional termination, the RNA Polymerase activity is altered, resulting in more efficient removal of introns, which could impact gene expression. We favour a different scenario, however. Splicing was also shown to have an important impact on transgene silencing (Christie et al., 2011), an observation further supported by the identification of a splicing cofactor mutant showing enhanced RNA silencing (Herr et al., 2006). In accordance, transgenes carrying introns show higher expression levels and accumulate fewer sRNAs than constructs lacking this element (Christie et al., 2011; Dadami et al., 2013). In our experiments, we also detected an association between the presence of siRNAs and unspliced mRNAs (Figures 7B,C). We have previously seen this same association between sRNAs and splicing when studying the effect of different terminators on gene expression (de Felippes et al., 2020). Curiously, when the sRNAs were inactivated by the presence of the viral suppressor of RNA silencing P19, intron retention could not be further detected, indicating a direct role of RNA silencing in splicing efficiency. Thus, we believe that the defects in splicing observed here are a consequence of the presence of sRNAs rather than a direct effect of transcription termination on the processing of introns. Nonetheless, the mechanism of how sRNAs would disturb the splicing process is still elusive and will require further investigation.

Taking into consideration all of what has been discussed above, we propose the following model to explain the high expression efficiency obtained when terminators such as the tHSP are used as a 3′ regulatory unit (Figure 7D): in most cases, where a balance between transcription levels and termination exists, the generation of aberrant read-through transcripts is kept to a minimum, with such molecules being degraded by XRN4 and the RNA exosome (Souret et al., 2004; Moreno et al., 2013; Branscheid et al., 2015; Yu et al., 2015; Zhang et al., 2015). As a consequence, defective mRNAs are not available to enter the RDR6 pathway, which would lead to sRNA production. This scenario is supported by numerous studies showing an increase of sRNAs and gene silencing in plants carrying mutations in factors involved in the RNA decay pathway (Gazzani et al., 2004; Gy et al., 2007; Gregory et al., 2008; Moreno et al., 2013; Branscheid et al., 2015; Yu et al., 2015; Zhang et al., 2015; Lange et al., 2019). If for some reason (for instance, transgenic systems using strong promoters), the terminator efficiency is not sufficient to cope with transcriptional rates, the levels of aberrant mRNAs originating from read-through events reach a threshold where some of these defective transcripts escape the RNA decay pathway and are targeted by RDR6, resulting in sRNA production and gene silencing. Different from other terminators tested in this work, the tHSP carries a newly-identified element in its 5′ end which is involved in improving transcription termination, reducing the amounts of aberrant transcripts to a level where they are efficiently degraded by the RNA decay pathway and, thus, not available to RDR6. As a result, the production of sRNAs from the respective locus, and consequently, gene silencing, is kept to a minimum. In addition, the resulting low levels of sRNAs are insufficient to disturb the splicing process, allowing for robust gene expression.

An interesting aspect of the newly identified element in the tHSP is its transferability. We showed that the efficiency of weaker terminators can be improved with the presence of the tHSP 5′ region in their sequences, providing a new approach to modulate gene expression based on the manipulation of 3′ regulatory sequences. The conversion of weaker terminators into more efficient regulatory units also has a dramatic impact on the availability of sequences that can be used to deliver good gene expression levels, which is especially attractive in situations where the use of a variety of terminators is preferred. In addition, a fundamental understanding of the different elements in a plant terminator is essential for the rational design of synthetic terminators, which could lead to smaller, more efficient regulatory regions (Deaner and Alper, 2018). The generation of such synthetic units is still incipient in plants, especially when compared to yeast, where several examples of rationally designed terminators already exist (Curran et al., 2015; MacPherson and Saka, 2017; Ahmed et al., 2019; Wang et al., 2019). Thus, the tHSP 5′ element appears as a good candidate to be included in the design of future synthetic terminators in plants. This sequence can also be an important tool to identify new efficient terminators for transgene expression. Indeed, a preliminary BLAST search revealed several 3′ UTR regions in *A. thaliana* carrying a sequence with some degree of similarity with the tHSP 5′ region. However, the efficiency of these regions as a terminator and the role of the putative tHSP homologous sequence in gene expression would still need to be tested.

Gene expression is a complex outcome involving different levels of regulation, from transcription to post-translational events. The participation of terminators in this process, however, is still not fully appreciated. The findings presented here, make an important contribution to understanding the natural design and role of plant terminators, open new avenues of gene manipulation, and describe yet another layer of gene control involving RDR6-dependent sRNAs.

## Data Availability Statement

The original contributions presented in the study are included in the article/Supplementary Material, further inquiries can be directed to the corresponding author.

## Author Contributions

FFF designed the experiments. FFF and KS conducted the research. FFF and PW analysed the data and wrote the manuscript. All authors contributed to the article and approved the submitted version.

## Conflict of Interest

The authors declare that the research was conducted in the absence of any commercial or financial relationships that could be construed as a potential conflict of interest.

## Publisher’s Note

All claims expressed in this article are solely those of the authors and do not necessarily represent those of their affiliated organizations, or those of the publisher, the editors and the reviewers. Any product that may be evaluated in this article, or claim that may be made by its manufacturer, is not guaranteed or endorsed by the publisher.
