# Supplementary material for: Identification of a Transferrable Terminator Element That Inhibits Small RNA Production and Improves Transgene Expression Levels
Source: Front Plant Sci. 2022 May 16;13:877793. doi: 10.3389/fpls.2022.877793 (PMC9149433; doi:10.3389/fpls.2022.877793)
Supplement: Supplementary file 2 [file Data_Sheet_1.docx]

**Supplementary Data 1.** The sequence of the different terminators used in the present work is given bellow. Mutations are indicated with underlined letters, whereas the 5’ fragment of the tHSP is represented in **bold**. Sequences from the tNOS and tRBCS added to the tACS2 is shown in *italic*.

***HSP* terminator**

**ATATGAAGATGAAGATGAAATATTTGGTGTGT**CAAATAAAAAGCTTGTGTGCTTAAGTTTGTGTTTTTTTCTTGGCTTGTTGTGTTATGAATTTGTGGCTTTTTCTAATATTAAATGAATGTAAGATCTCATTATAATGAATAAACAAATGTTTCTATAATCCATTGTGAATGTTTTGTTGGATCTCTTCTGCAGCATATAACTACTGTATGTGCTATGGTATGGACTATGGAATATGATTAAAGATAAG

***ACS2* terminator**

AAATCTTAAGGCATAACGTCTGAGAGATTGGATTAACTCGTCCGCGTTTCACTCCGTGTTAATTAATCTTAAATTAGTAAGTGATTAAGTAAATGTTTTTTCTTTCATTGTAAGATTGGAATAATTCAATTTCGACATTAGGGTTGTTTTTGACGGCCAGCTTTTTTCCTGGGGTCAAATGGTAACTTTTAAGATTTTATGTGTTTGATTCTGTTTCTTTTTTCCGCTTAGGATTTTAATCGATGGATTGTCCTAGTGGTGCTGGTGTGTAGCATATATGCTTTTCTTATATGTTTTTGTGTGTAATAAATGAAACATTGTCTTTTGATAAGGATCACCAGAGTTTATTAGTTGGGGAGGTTGATAATGTTTTGTGAGTAATGGAGGATTTGTTAACCTAATTTATTCGATTTTTTCTAGAACCGCATTTTCTTGTTCGCCCAATACGTCACACGAGCATGCCAACATGCCTATCCTTTTTCTAAAATAATCATTAT

***RBCS1A* terminator**

TTTCCCTTTGCTTTTGTGTAAACCTCAAAACTTTATCCCCCATCTTTGATTTTATCCCTTGTTTTTCTGCTTTTTTCTTCTTTCTTGGGTTTTAATTTCCGGACTTAACGTTTGTTTTCCGGTTTGCGAGACATATTCTATCGGATTCTCAACTGTCTGATGAAATAAATATGTAATGTTCTATAAGTCTTTCAATTTGATATGCATATCAACAAAAAGAAAATAGGACAATGCGGCTACAAATATGAAATTTACAAGTTTAAGAACCATGAGTCGCTAAAGAAATCATTAAGAAAATTAGTTTCACATTCAATTCTTGTCACATGATTAACGAGCTTGAGAGGTTTAGAGTAACAATATCTTGAAGCAAAAGATGACCCACTTGAAATCTAGTGATGGATACATAAGTGGACGTGCCTTGTTTAGGATAGGATTCTGGATAAGAGTCTCGAATATTCATTTTTACCAAGTATATTCAAGGATCTTGTGGATCATATATTTCCTCAATCAAAGGGACTTGACCCAAATTCACATAAAGATATTTTGGAGTCGTTTGTTTAGGTACTTTCGAAATAGGATAAGCTATGTGTGGGTCGTAAAAACTCAAAATTTTCTTTCATTTTTTTTTTTAATTTTTTCCTAATTAGTTACTGATATAGTGTAAAGTTTTGAAATATTTTAAACGATTTTTACCCCTTTTTAATAATTTTTGTACCATTTAATCTCAAGAATTATACATATTGTTTACCAAAAGGTTTTGGTCACCATCGAAAACATCGAAATGCTCAAATACCACAGAAAGATTGAAAGATAGGAGATACCAAAGATTTGTCTTTTTTTTCTTTTTTTCACAATTTGTTTTTGTTTTCATGTGTTGTTTTTTTGTTAAATAAACAAATTAGGTGGGAGTGATAAAGCAAAAAAACAAAGTGGGTGGGTCAAAAGGCCCATTAATACCAAAGCAAATAAGTTATCATACCAAATAGCCCACC

***NOS* terminator**

GATCGTTCAAACATTTGGCAATAAAGTTTCTTAAGATTGAATCCTGTTGCCGGTCTTGCGATGATTATCATATAATTTCTGTTGAATTACGTTAAGCATGTAATAATTAACATGTAATGCATGACGTTATTTATGAGATGGGTTTTTATGATTAGAGTCCCGCAATTATACATTTAATACGCGATAGAAAACAAAATATAGCGCGCAAACTAGGATAAATTATCGCGCGCGGTGTCATCTATGTTACTAGATC

***H4* terminator**

TTAGAGTTTTTCAGATCCGCGTTTGTGTTTTCTGGGTTTCTCACTTAAGCGTCTGCGTTTTACTTTTGTATTGGGTTTGGCGTTTAGTAGTTTGCGGTAGCGTTCTTGTTATGTGTAATTACGCTTTTTCTTCTTGCTTCAGCAGTTTCGGTTGAAATATAAATCGAATCAAGTTTCACTTTATCAGCGTTGTTTTAAATTTTGGCATTAAATTGGTGAAAATTGCTTCAATTTTGTATCTAAATAGAAG

**1^st^_NUE_mut**

**ATATGAAGATGAAGATGAAATATTTGGTGTGT**CATTGTGTAAGCTTGTGTGCTTAAGTTTGTGTTTTTTTCTTGGCTTGTTGTGTTATGAATTTGTGGCTTTTTCTAATATTAAATGAATGTAAGATCTCATTATAATGAATAAACAAATGTTTCTATAATCCATTGTGAATGTTTTGTTGGATCTCTTCTGCAGCATATAACTACTGTATGTGCTATGGTATGGACTATGGAATATGATTAAAGATAAG

**2^nd^_NUE_mut**

**ATATGAAGATGAAGATGAAATATTTGGTGTGT**CAAATAAAAAGCTTGTGTGCTTAAGTTTGTGTTTTTTTCTTGGCTTGTTGTGTTATGAATTTGTGGCTTTTTCTAATATTAAATGAATGTAAGATCTCATTATAATGTTGTGTCAAATGTTTCTATAATCCATTGTGAATGTTTTGTTGGATCTCTTCTGCAGCATATAACTACTGTATGTGCTATGGTATGGACTATGGAATATGATTAAAGATAAG

**Both_NUE_mut**

**ATATGAAGATGAAGATGAAATATTTGGTGTGT**CATTGTGTAAGCTTGTGTGCTTAAGTTTGTGTTTTTTTCTTGGCTTGTTGTGTTATGAATTTGTGGCTTTTTCTAATATTAAATGAATGTAAGATCTCATTATAATGTTGTGTCAAATGTTTCTATAATCCATTGTGAATGTTTTGTTGGATCTCTTCTGCAGCATATAACTACTGTATGTGCTATGGTATGGACTATGGAATATGATTAAAGATAAG

**tHSP_3’∆43**

**ATATGAAGATGAAGATGAAATATTTGGTGTGT**CAAATAAAAAGCTTGTGTGCTTAAGTTTGTGTTTTTTTCTTGGCTTGTTGTGTTATGAATTTGTGGCTTTTTCTAATATTAAATGAATGTAAGATCTCATTATAATGAATAAACAAATGTTTCTATAATCCATTGTGAATGTTTTGTTGGATCTCTTCTGCAGCATATAACTACT

**tHSP_5’∆32**

CAAATAAAAAGCTTGTGTGCTTAAGTTTGTGTTTTTTTCTTGGCTTGTTGTGTTATGAATTTGTGGCTTTTTCTAATATTAAATGAATGTAAGATCTCATTATAATGAATAAACAAATGTTTCTATAATCCATTGTGAATGTTTTGTTGGATCTCTTCTGCAGCATATAACTACTGTATGTGCTATGGTATGGACTATGGAATATGATTAAAGATAAG

**tHSP_5’∆42**

GCTTGTGTGCTTAAGTTTGTGTTTTTTTCTTGGCTTGTTGTGTTATGAATTTGTGGCTTTTTCTAATATTAAATGAATGTAAGATCTCATTATAATGAATAAACAAATGTTTCTATAATCCATTGTGAATGTTTTGTTGGATCTCTTCTGCAGCATATAACTACTGTATGTGCTATGGTATGGACTATGGAATATGATTAAAGATAAG

**tHSP_5’∆104**

CTAATATTAAATGAATGTAAGATCTCATTATAATGAATAAACAAATGTTTCTATAATCCATTGTGAATGTTTTGTTGGATCTCTTCTGCAGCATATAACTACTGTATGTGCTATGGTATGGACTATGGAATATGATTAAAGATAAG

**tACS2_tHSP_5’**

**ATATGAAGATGAAGATGAAATATTTGGTGTGT**TATGTGTTTGATTCTGTTTCTTTTTTCCGCTTAGGATTTTAATCGATGGATTGTCCTAGTGGTGCTGGTGTGTAGCATATATGCTTTTCTTATATGTTTTTGTGTGTAATAAATGAAACATTGTCTTTTGATAAGGATCACCAGAGTTTATTAGTTGGGGAGGTTGATAATGTTTTGTGAGTAATGGAGGATTTGTTAACCTAATTTATTCGATTTTTTCTAGAACCGCATTTTCTTGTTCGCCCAATACGTCACACGAGCATGCCAACATGCCTATCCTTTTTCTAAAATAATCATTAT

**tACS2_tHSP_5’_short**

**ATATGAAGATGAAGATGAAATATTTGGTGTGT**AATAAATGAAACATTGTCTTTTGATAAGGATCACCAGAGTTTATTAGTTGGGGAGGTTGATAATGTTTTGTGAGTAATGGAGGATTTGTTAACCTAATTTATTCGATTTTTTCTAGAACCGCATTTTCTTGTTCGCCCAATACGTCACACGAGCATGCCAACATGCCTATCCTTTTTCTAAAATAATCATTAT

**tACS2_tHSP_5’_long**

**ATATGAAGATGAAGATGAAATATTTGGTGTGTGT**GATTAAGTAAATGTTTTTTCTTTCATTGTAAGATTGGAATAATTCAATTTCGACATTAGGGTTGTTTTTGACGGCCAGCTTTTTTCCTGGGGTCAAATGGTAACTTTTAAGATTTTATGTGTTTGATTCTGTTTCTTTTTTCCGCTTAGGATTTTAATCGATGGATTGTCCTAGTGGTGCTGGTGTGTAGCATATATGCTTTTCTTATATGTTTTTGTGTGTAATAAATGAAACATTGTCTTTTGATAAGGATCACCAGAGTTTATTAGTTGGGGAGGTTGATAATGTTTTGTGAGTAATGGAGGATTTGTTAACCTAATTTATTCGATTTTTTCTAGAACCGCATTTTCTTGTTCGCCCAATACGTCACACGAGCATGCCAACATGCCTATCCTTTTTCTAAAATAATCATTAT

**tNOS_tHSP_5’**

**ATATGAAGATGAAGATGAAATATTTGGTGTGT**GATCGTTCAAACATTTGGCAATAAAGTTTCTTAAGATTGAATCCTGTTGCCGGTCTTGCGATGATTATCATATAATTTCTGTTGAATTACGTTAAGCATGTAATAATTAACATGTAATGCATGACGTTATTTATGAGATGGGTTTTTATGATTAGAGTCCCGCAATTATACATTTAATACGCGATAGAAAACAAAATATAGCGCGCAAACTAGGATAAATTATCGCGCGCGGTGTCATCTATGTTACTAGATC

**tRBCS_tHSP_5’**

**ATATGAAGATGAAGATGAAATATTTGGTGTGT**CCTTGTTTTTCTGCTTTTTTCTTCTTTCTTGGGTTTTAATTTCCGGACTTAACGTTTGTTTTCCGGTTTGCGAGACATATTCTATCGGATTCTCAACTGTCTGATGAAATAAATATGTAATGTTCTATAAGTCTTTCAATTTGATATGCATATCAACAAAAAGAAAATAGGACAATGCGGCTACAAATATGAAATTTACAAGTTTAAGAACCATGAGTCGCTAAAGAAATCATTAAGAAAATTAGTTTCACATTCAATTCTTGTCACATGATTAACGAGCTTGAGAGGTTTAGAGTAACAATATCTTGAAGCAAAAGATGACCCACTTGAAATCTAGTGATGGATACATAAGTGGACGTGCCTTGTTTAGGATAGGATTCTGGATAAGAGTCTCGAATATTCATTTTTACCAAGTATATTCAAGGATCTTGTGGATCATATATTTCCTCAATCAAAGGGACTTGACCCAAATTCACATAAAGATATTTTGGAGTCGTTTGTTTAGGTACTTTCGAAATAGGATAAGCTATGTGTGGGTCGTAAAAACTCAAAATTTTCTTTCATTTTTTTTTTTAATTTTTTCCTAATTAGTTACTGATATAGTGTAAAGTTTTGAAATATTTTAAACGATTTTTACCCCTTTTTAATAATTTTTGTACCATTTAATCTCAAGAATTATACATATTGTTTACCAAAAGGTTTTGGTCACCATCGAAAACATCGAAATGCTCAAATACCACAGAAAGATTGAAAGATAGGAGATACCAAAGATTTGTCTTTTTTTTCTTTTTTTCACAATTTGTTTTTGTTTTCATGTGTTGTTTTTTTGTTAAATAAACAAATTAGGTGGGAGTGATAAAGCAAAAAAACAAAGTGGGTGGGTCAAAAGGCCCATTAATACCAAAGCAAATAAGTTATCATACCAAATAGCCCACC

**tH4_tHSP_5’**

**ATATGAAGATGAAGATGAAATATTTGGTGTGT**GTCTGCGTTTTACTTTTGTATTGGGTTTGGCGTTTAGTAGTTTGCGGTAGCGTTCTTGTTATGTGTAATTACGCTTTTTCTTCTTGCTTCAGCAGTTTCGGTTGAAATATAAATCGAATCAAGTTTCACTTTATCAGCGTTGTTTTAAATTTTGGCATTAAATTGGTGAAAATTGCTTCAATTTTGTATCTAAATAGAAG

**tACS2_2x_tHSP_5’**

**ATATGAAGATGAAGATGAAATATTTGGTGTGT**GGGCCGC**ATATGAAGATGAAGATGAAATATTTGGTGTGT**TATGTGTTTGATTCTGTTTCTTTTTTCCGCTTAGGATTTTAATCGATGGATTGTCCTAGTGGTGCTGGTGTGTAGCATATATGCTTTTCTTATATGTTTTTGTGTGTAATAAATGAAACATTGTCTTTTGATAAGGATCACCAGAGTTTATTAGTTGGGGAGGTTGATAATGTTTTGTGAGTAATGGAGGATTTGTTAACCTAATTTATTCGATTTTTTCTAGAACCGCATTTTCTTGTTCGCCCAATACGTCACACGAGCATGCCAACATGCCTATCCTTTTTCTAAAATAATCATTAT

**tNOS_2x_tHSP_5’**

**ATATGAAGATGAAGATGAAATATTTGGTGTGT**GGGCCGC**ATATGAAGATGAAGATGAAATATTTGGTGTGT**GATCGTTCAAACATTTGGCAATAAAGTTTCTTAAGATTGAATCCTGTTGCCGGTCTTGCGATGATTATCATATAATTTCTGTTGAATTACGTTAAGCATGTAATAATTAACATGTAATGCATGACGTTATTTATGAGATGGGTTTTTATGATTAGAGTCCCGCAATTATACATTTAATACGCGATAGAAAACAAAATATAGCGCGCAAACTAGGATAAATTATCGCGCGCGGTGTCATCTATGTTACTAGATC

**tRBCS_2x_tHSP_5’**

**ATATGAAGATGAAGATGAAATATTTGGTGTGT**GGGCCGC**ATATGAAGATGAAGATGAAATATTTGGTGTGT**CCTTGTTTTTCTGCTTTTTTCTTCTTTCTTGGGTTTTAATTTCCGGACTTAACGTTTGTTTTCCGGTTTGCGAGACATATTCTATCGGATTCTCAACTGTCTGATGAAATAAATATGTAATGTTCTATAAGTCTTTCAATTTGATATGCATATCAACAAAAAGAAAATAGGACAATGCGGCTACAAATATGAAATTTACAAGTTTAAGAACCATGAGTCGCTAAAGAAATCATTAAGAAAATTAGTTTCACATTCAATTCTTGTCACATGATTAACGAGCTTGAGAGGTTTAGAGTAACAATATCTTGAAGCAAAAGATGACCCACTTGAAATCTAGTGATGGATACATAAGTGGACGTGCCTTGTTTAGGATAGGATTCTGGATAAGAGTCTCGAATATTCATTTTTACCAAGTATATTCAAGGATCTTGTGGATCATATATTTCCTCAATCAAAGGGACTTGACCCAAATTCACATAAAGATATTTTGGAGTCGTTTGTTTAGGTACTTTCGAAATAGGATAAGCTATGTGTGGGTCGTAAAAACTCAAAATTTTCTTTCATTTTTTTTTTTAATTTTTTCCTAATTAGTTACTGATATAGTGTAAAGTTTTGAAATATTTTAAACGATTTTTACCCCTTTTTAATAATTTTTGTACCATTTAATCTCAAGAATTATACATATTGTTTACCAAAAGGTTTTGGTCACCATCGAAAACATCGAAATGCTCAAATACCACAGAAAGATTGAAAGATAGGAGATACCAAAGATTTGTCTTTTTTTTCTTTTTTTCACAATTTGTTTTTGTTTTCATGTGTTGTTTTTTTGTTAAATAAACAAATTAGGTGGGAGTGATAAAGCAAAAAAACAAAGTGGGTGGGTCAAAAGGCCCATTAATACCAAAGCAAATAAGTTATCATACCAAATAGCCCACC

**tH4_2x_tHSP_5’**

**ATATGAAGATGAAGATGAAATATTTGGTGTGT**GGGCCGC**ATATGAAGATGAAGATGAAATATTTGGTGTGT**GTCTGCGTTTTACTTTTGTATTGGGTTTGGCGTTTAGTAGTTTGCGGTAGCGTTCTTGTTATGTGTAATTACGCTTTTTCTTCTTGCTTCAGCAGTTTCGGTTGAAATATAAATCGAATCAAGTTTCACTTTATCAGCGTTGTTTTAAATTTTGGCATTAAATTGGTGAAAATTGCTTCAATTTTGTATCTAAATAGAAG

**tH4_tHSP_5’_min**

**ATATGAAGATGAAGATGAAATAT**GTCTGCGTTTTACTTTTGTATTGGGTTTGGCGTTTAGTAGTTTGCGGTAGCGTTCTTGTTATGTGTAATTACGCTTTTTCTTCTTGCTTCAGCAGTTTCGGTTGAAATATAAATCGAATCAAGTTTCACTTTATCAGCGTTGTTTTAAATTTTGGCATTAAATTGGTGAAAATTGCTTCAATTTTGTATCTAAATAGAAG

**tH4_tHSP_5’_mut1**

**CACCTTAGATGAAGATGAAATATTTGGTGTGT**GTCTGCGTTTTACTTTTGTATTGGGTTTGGCGTTTAGTAGTTTGCGGTAGCGTTCTTGTTATGTGTAATTACGCTTTTTCTTCTTGCTTCAGCAGTTTCGGTTGAAATATAAATCGAATCAAGTTTCACTTTATCAGCGTTGTTTTAAATTTTGGCATTAAATTGGTGAAAATTGCTTCAATTTTGTATCTAAATAGAAG

**tH4_tHSP_5’_mut2**

**ATATGATCTACTAGATGAAATATTTGGTGTGT**GTCTGCGTTTTACTTTTGTATTGGGTTTGGCGTTTAGTAGTTTGCGGTAGCGTTCTTGTTATGTGTAATTACGCTTTTTCTTCTTGCTTCAGCAGTTTCGGTTGAAATATAAATCGAATCAAGTTTCACTTTATCAGCGTTGTTTTAAATTTTGGCATTAAATTGGTGAAAATTGCTTCAATTTTGTATCTAAATAGAAG

**tH4_tHSP_5’_mut3**

**ATATGAAGATGATTCCCTAATATTTGGTGTGT**GTCTGCGTTTTACTTTTGTATTGGGTTTGGCGTTTAGTAGTTTGCGGTAGCGTTCTTGTTATGTGTAATTACGCTTTTTCTTCTTGCTTCAGCAGTTTCGGTTGAAATATAAATCGAATCAAGTTTCACTTTATCAGCGTTGTTTTAAATTTTGGCATTAAATTGGTGAAAATTGCTTCAATTTTGTATCTAAATAGAAG

**tH4_tHSP_5’_mut4**

**ATATGAAGATGAAGATGATCCCCATGGTGTGT**GTCTGCGTTTTACTTTTGTATTGGGTTTGGCGTTTAGTAGTTTGCGGTAGCGTTCTTGTTATGTGTAATTACGCTTTTTCTTCTTGCTTCAGCAGTTTCGGTTGAAATATAAATCGAATCAAGTTTCACTTTATCAGCGTTGTTTTAAATTTTGGCATTAAATTGGTGAAAATTGCTTCAATTTTGTATCTAAATAGAAG

**tH4_tHSP_5’_mut5**

**ATATGAAGATGAAGATGAAATATTACCACACA**GTCTGCGTTTTACTTTTGTATTGGGTTTGGCGTTTAGTAGTTTGCGGTAGCGTTCTTGTTATGTGTAATTACGCTTTTTCTTCTTGCTTCAGCAGTTTCGGTTGAAATATAAATCGAATCAAGTTTCACTTTATCAGCGTTGTTTTAAATTTTGGCATTAAATTGGTGAAAATTGCTTCAATTTTGTATCTAAATAGAAG

**tACS2_5’_del**

TATGTGTTTGATTCTGTTTCTTTTTTCCGCTTAGGATTTTAATCGATGGATTGTCCTAGTGGTGCTGGTGTGTAGCATATATGCTTTTCTTATATGTTTTTGTGTGTAATAAATGAAACATTGTCTTTTGATAAGGATCACCAGAGTTTATTAGTTGGGGAGGTTGATAATGTTTTGTGAGTAATGGAGGATTTGTTAACCTAATTTATTCGATTTTTTCTAGAACCGCATTTTCTTGTTCGCCCAATACGTCACACGAGCATGCCAACATGCCTATCCTTTTTCTAAAATAATCATTAT

**tACS2_tNOS_5’**

*GATCGTTCAAACATTTGGCAATAAAGTTTCTT*TATGTGTTTGATTCTGTTTCTTTTTTCCGCTTAGGATTTTAATCGATGGATTGTCCTAGTGGTGCTGGTGTGTAGCATATATGCTTTTCTTATATGTTTTTGTGTGTAATAAATGAAACATTGTCTTTTGATAAGGATCACCAGAGTTTATTAGTTGGGGAGGTTGATAATGTTTTGTGAGTAATGGAGGATTTGTTAACCTAATTTATTCGATTTTTTCTAGAACCGCATTTTCTTGTTCGCCCAATACGTCACACGAGCATGCCAACATGCCTATCCTTTTTCTAAAATAATCATTAT

**tACS2_tRBCS_5’**

*TTTCCCTTTGCTTTTGTGTAAACCTCAAAACT*TATGTGTTTGATTCTGTTTCTTTTTTCCGCTTAGGATTTTAATCGATGGATTGTCCTAGTGGTGCTGGTGTGTAGCATATATGCTTTTCTTATATGTTTTTGTGTGTAATAAATGAAACATTGTCTTTTGATAAGGATCACCAGAGTTTATTAGTTGGGGAGGTTGATAATGTTTTGTGAGTAATGGAGGATTTGTTAACCTAATTTATTCGATTTTTTCTAGAACCGCATTTTCTTGTTCGCCCAATACGTCACACGAGCATGCCAACATGCCTATCCTTTTTCTAAAATAATCATTAT

**tHSP_int∆72**

**ATATGAAGATGAAGATGAAATATTTGGTGTGT**CTAATATTAAATGAATGTAAGATCTCATTATAATGAATAAACAAATGTTTCTATAATCCATTGTGAATGTTTTGTTGGATCTCTTCTGCAGCATATAACTACTGTATGTGCTATGGTATGGACTATGGAATATGATTAAAGATAAG
