# Supplementary material for: Identification of a Transferrable Terminator Element That Inhibits Small RNA Production and Improves Transgene Expression Levels
Source: Front Plant Sci. 2022 May 16;13:877793. doi: 10.3389/fpls.2022.877793 (PMC9149433; doi:10.3389/fpls.2022.877793)
Supplement: Supplementary file 3 [file Data_Sheet_2.docx]

**Supplementary Table 1.** List of primers and probes used in this work. Restriction enzyme sites are shown underlined.

| Primer name | sequence | target | Observation |
| --- | --- | --- | --- |
| *tHSP mutations* | | | |
| tHSP(notI)-F | ACTGCGGCCGCATATGAAGATGAAGATGAAATAT | tHSP | NotI site |
| FQ-052 | AAACCATGGCTTATCTTTAATCATATTCCATAGT | tHSP | NcoI site |
| *tACS2 and tH4 amplification* | | | |
| FQ-076 | AAAGCGGCCGCAAATCTTAAGGCATAACG | tACS2 | NotI site |
| FQ-077 | AAACCATGGATAATGATTATTTTAGAAAAAG | tACS2 | NcoI site |
| FQ-218 | AAAGCGGCCGCTTAGAGTTTTTCAGATCCGCGTTTG | tH4 | NotI site |
| FQ-216 | AAACCATGGCTTCTATTTAGATACAAAATTGAAGC | tH4 | NcoI site |
| *tHSP deletions* |  |  |  |
| FQ-052 | AAACCATGGCTTATCTTTAATCATATTCCATAGT | tHSP | NcoI site |
| FQ-094 | AAAGCGGCCGCCAAATAAAAAGCTTGTGTGC | tHSP | NotI site |
| FQ-092 | AAAGCGGCCGCGCTTGTGTGCTTAAGTTTG | tHSP | NotI site |
| FQ-093 | AAAGCGGCCGCCTAATATTAAATGAATGTAAG | tHSP | NotI site |
| FQ-067 | AAACCATGGAGTAGTTATATGCTGCAG | tHSP | NcoI site |
| tHSP(notI)-F | ACTGCGGCCGCATATGAAGATGAAGATGAAATAT | tHSP | NotI site |
| *Insertion of one tHSP 5’ copy* | | | |
| tHSP(notI)-F | ACTGCGGCCGCATATGAAGATGAAGATGAAATAT | tHSP | NotI site |
| FQ-077 | AAACCATGGATAATGATTATTTTAGAAAAAG | tACS2 | NcoI site |
| FQ-184 | AAACCATGGGATCTAGTAACATAGATGACACC | tNOS | NcoI site |
| FQ-185 | AAACCATGGGGTGGGCTATTTGGTATGATAA | tRBCS | NcoI site |
| FQ-187 | AAACCATGGCTTCTATTTAGATAC | tH4 | NcoI site |
| *Addition of a second copy of the tHSP 5’ fragment* | | | |
| FQ-231 | AAAGGGCCCATATGAAGATGAAGATGAAATATTTGGTGTGTGGGCCCAAA |  | PspOMI sites |
| FQ-232 | TTTGGGCCCACACACCAAATATTTCATCTTCATCTTCATATGGGCCCTTT |  | PspOMI sites |
| *Characterization of the tHSP 5’ end* |  |  |  |
| tHSP(notI)-F | ACTGCGGCCGCATATGAAGATGAAGATGAAATAT | tHSP | NotI site |
| FQ-216 | AAACCATGGCTTCTATTTAGATACAAAATTGAAGC | tH4 | NcoI site |
| FQ-241 | AAAGCGGCCGCCACCTTAGATGAAGATGAAATAT | tHSP | NotI site |
| FQ-242 | AAAGCGGCCGCATATGATCTACTAGATGAAATAT | tHSP | NotI site |
| FQ-243 | AAAGCGGCCGCATATGAAGATGATTCCCTAATAT | tHSP | NotI site |
| FQ-245 | AAAGCGGCCGCATATGAAGATGAAGATGATCCCC | tHSP | NotI site |
| *RT-PCR and RT-qPCR* | | | |
| FQ-102 (RT-primer) | TCCCGACTGATGTCAGAGC | HPL1 promoter | region downstream of terminator |
| FQ-003 | AGATCCGCCACAACATCGAG | GFP | Used for intron 2 test |
| FQ-004 | TTGTACAGCTCGTCCATGCC | GFP | Used for intron 2 test/RT-qPCR |
| Ben972 | GCAAGCTGACCCTGAAGTTC | GFP | Used for intron 1 test |
| FQ-002 | CGATGCCCTTCAGCTCGATG | GFP | Used for intron 1 test |
| FQ-237 | TTGCACTACCAACTGCCTTG | GAPDH | Used for intron test |
| FQ-238 | CAGCTCTTCCACCTCTCCAG | GAPDH | Used for intron test |
| FQ-103 (read-through forward) | CGTGGATACTTGGCAGTGG | HPL1 promoter | region downstream of terminator |
| FQ-104 (read-through reverse) | TGTTGTAGCGTTACTATGAAGACC | HPL1 promoter | region downstream of terminator |
| Ben881 | AGCAAAGACCCCAACGAGAA | GFP | Used for RT-qPCR |
| Ben0010F | CACTACCAACTGCCTTGCAC | GAPDH | Used for RT-qPCR |
| Ben0011R | ATGAAGCAGCTCTTCCACCT | GAPDH | Used for RT-qPCR |
| *Poly(A) characterization* | | | |
| FQ-168 (cDNA /1^st^ PCR) | GAACTTCAGGGTCAGCTTGC | GFP |  |
| Ben881F (1^st^ PCR) | AGCAAAGACCCCAACGAGAA | GFP |  |
| FQ-167 (nested PCR) | GTCCAGCTCGACCAGGATG | GFP |  |
| Ben934F (nested PCR) | GCATGGACGAGCTGTACAAG | GFP |  |
| *sRNA blot probes* |  |  |  |
| FQ-003 | AGATCCGCCACAACATCGAG | GFP |  |
| FQ-004 | TTGTACAGCTCGTCCATGCC | GFP |  |
| FQ-107 | AGGGGCCATGCTAATCTTCTC | U6 |  |
