# Supplementary material for: Identification of a Transferrable Terminator Element That Inhibits Small RNA Production and Improves Transgene Expression Levels
Source: Front Plant Sci. 2022 May 16;13:877793. doi: 10.3389/fpls.2022.877793 (PMC9149433; doi:10.3389/fpls.2022.877793)

cRT-PCR sequences

tHSP

1C70ZAB036.ab1

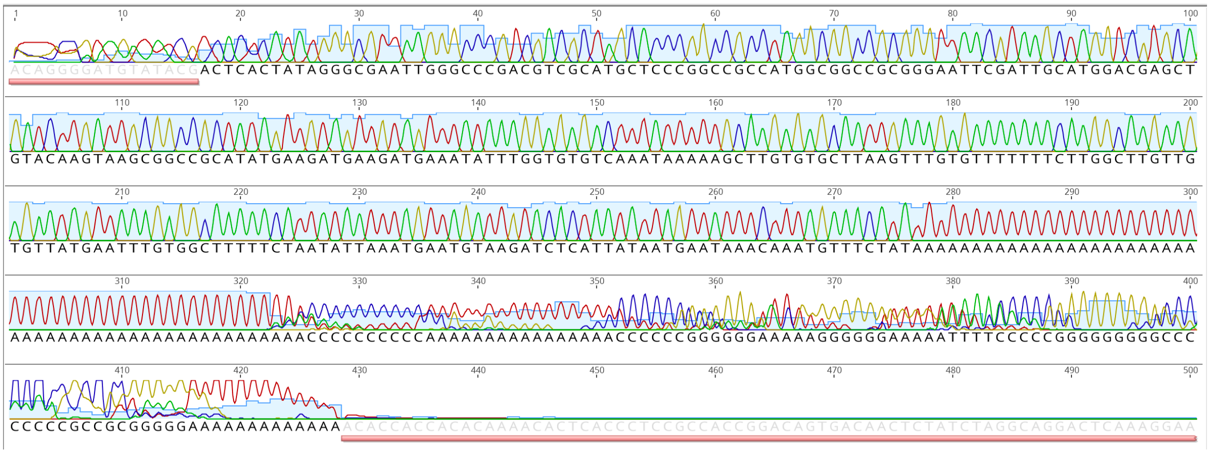

1C70ZAB037.ab1

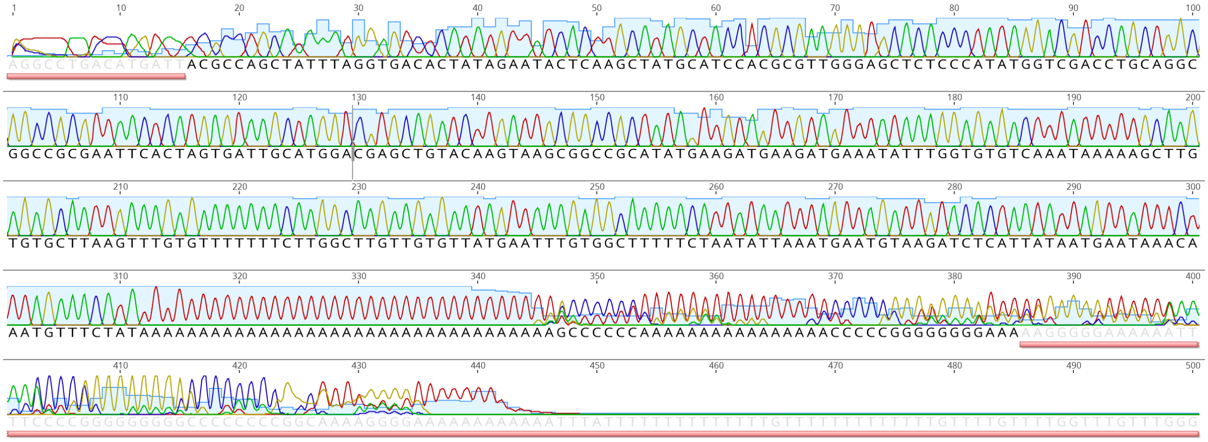

1C70ZAB038.ab1

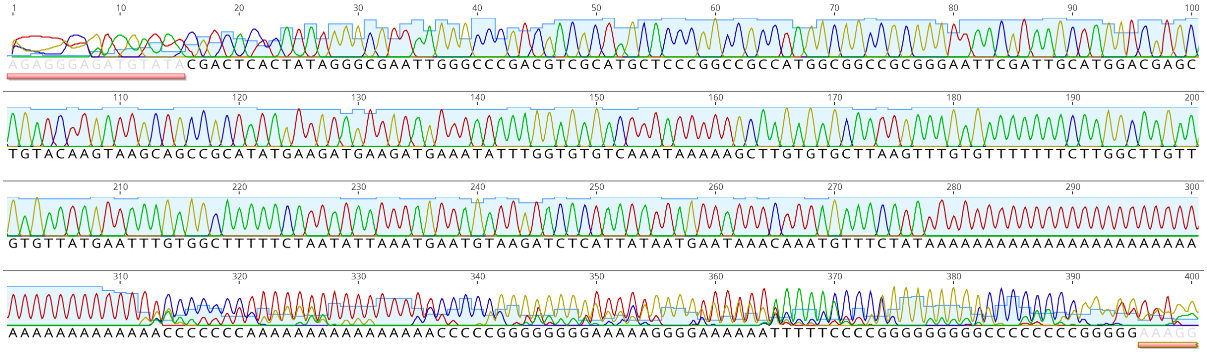

1C70ZAB039.ab1

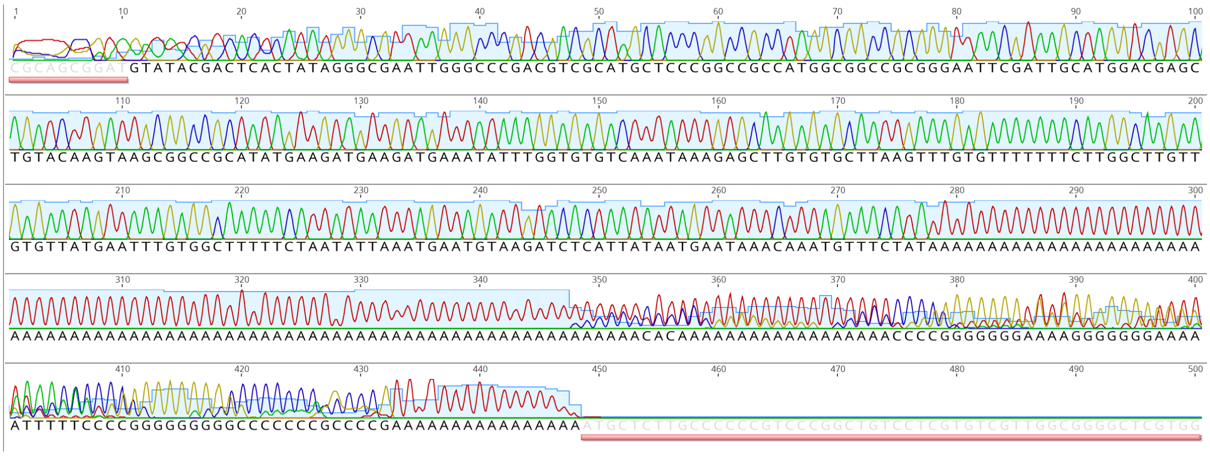

1C70ZAB040.ab1

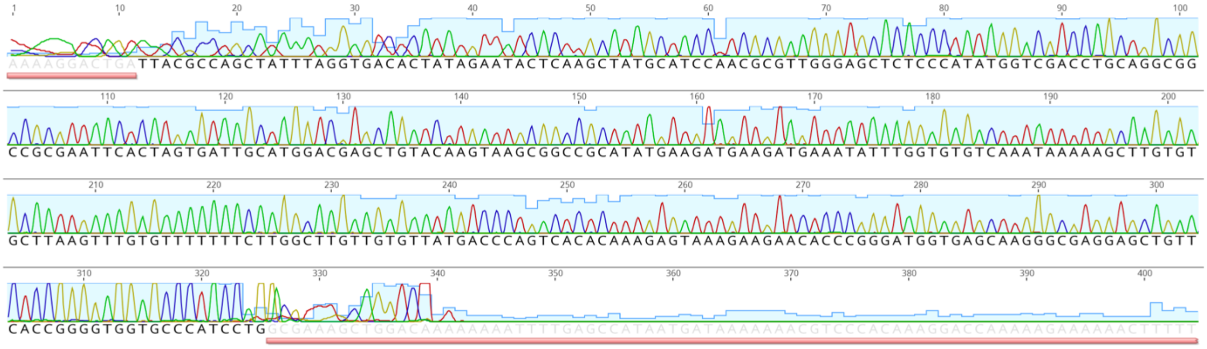

1C70ZAB041.ab1

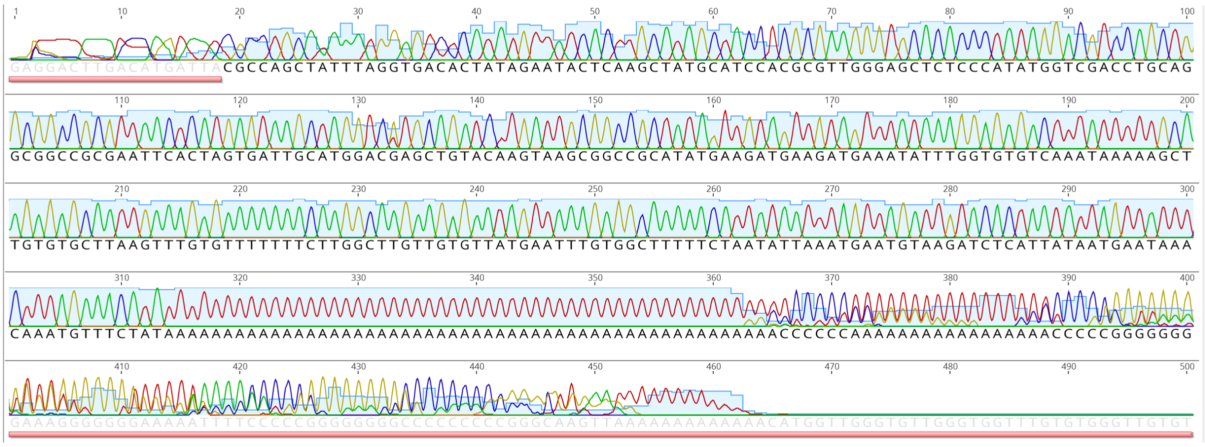

1C70ZAB042.ab1

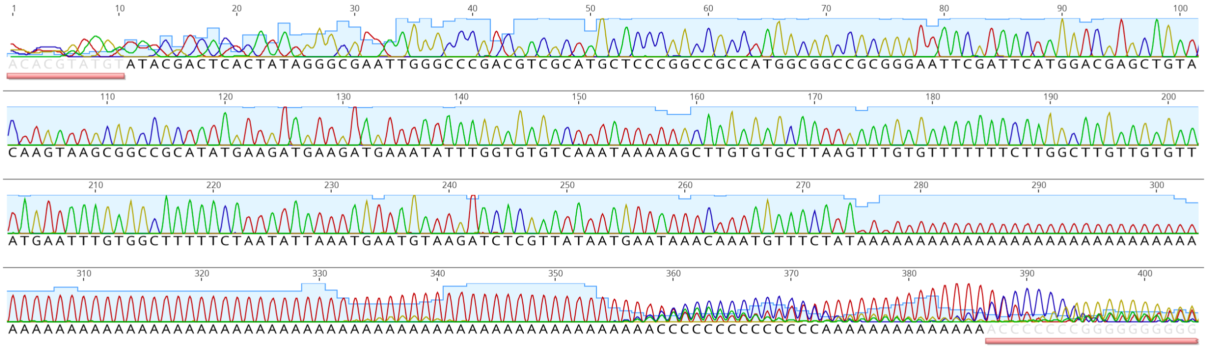

1C70ZAB043.ab1

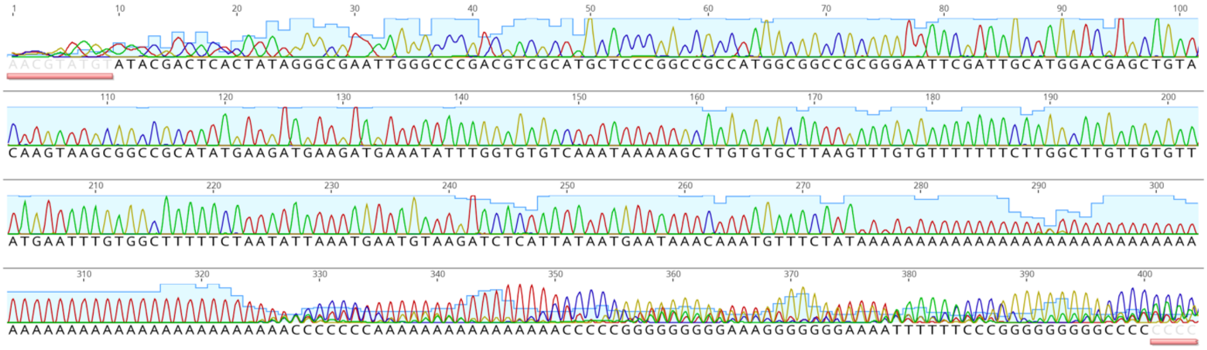

1C70ZAB044.ab1

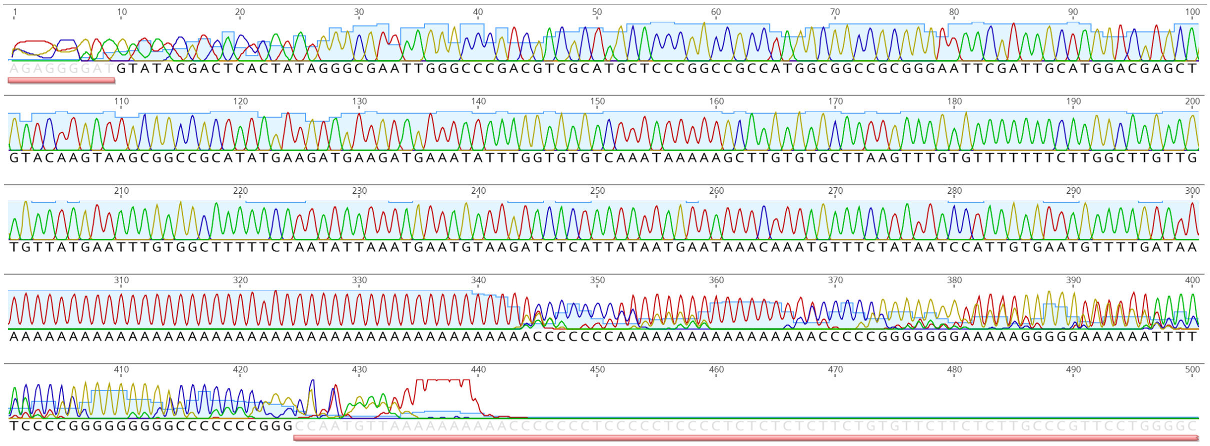

1C70ZAB045.ab1

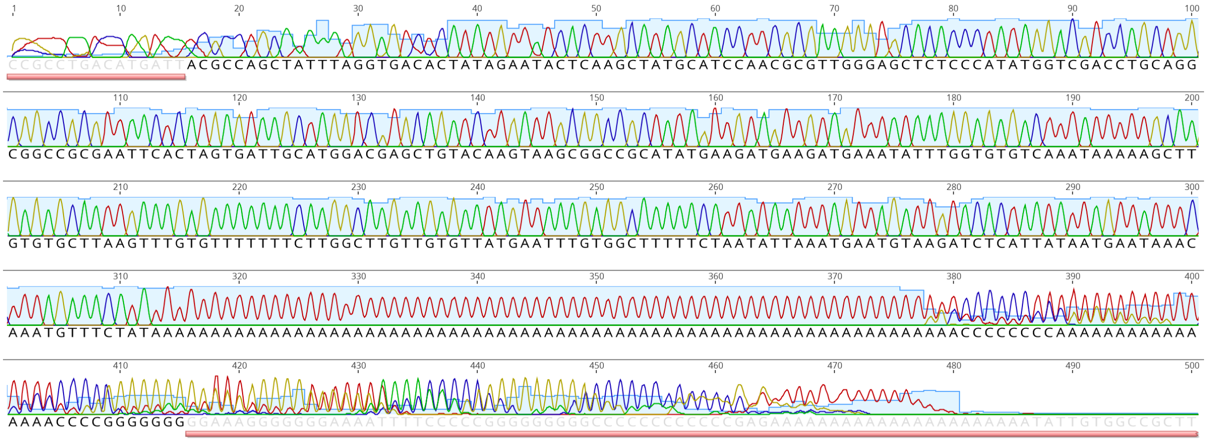

1D6BHAA001_A1.ab1

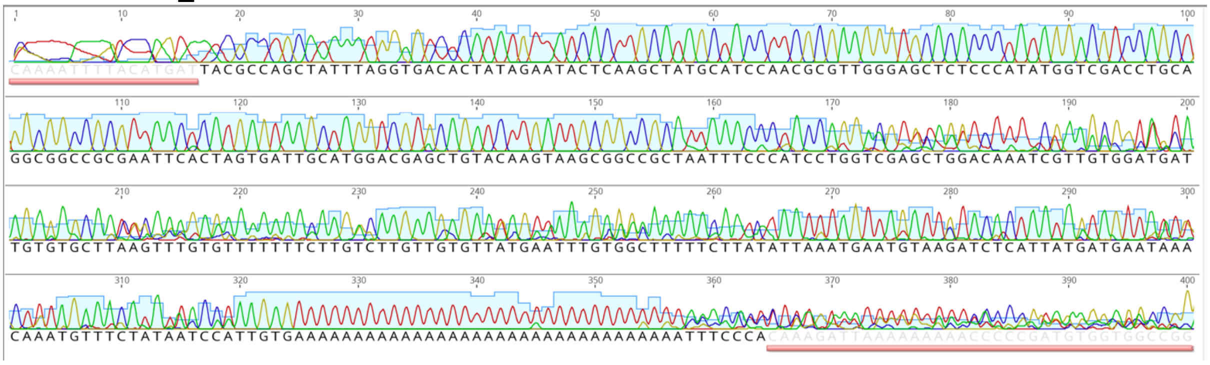

1D6BHAA001_B1.ab1

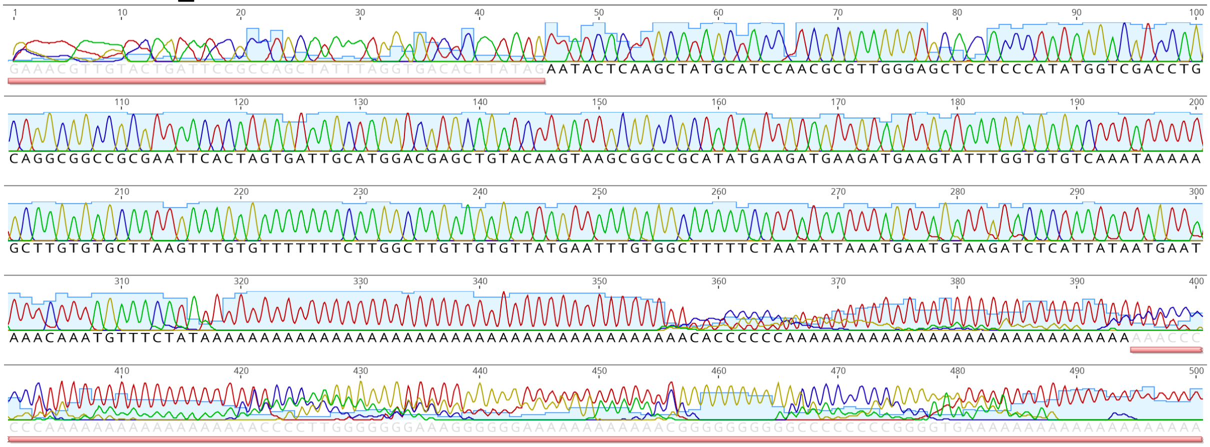

1D6BHAA001_C1.ab1

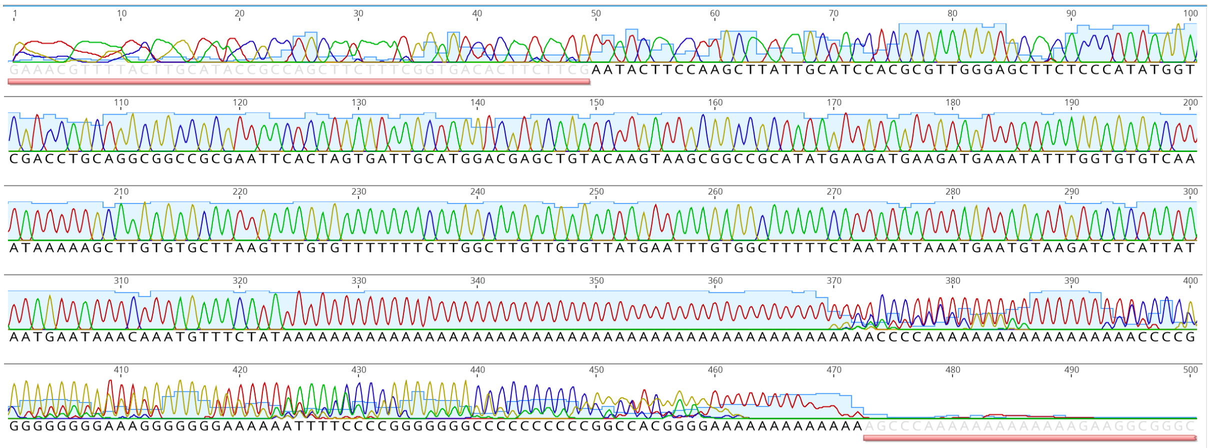

1D6BHAA001_D1.ab1

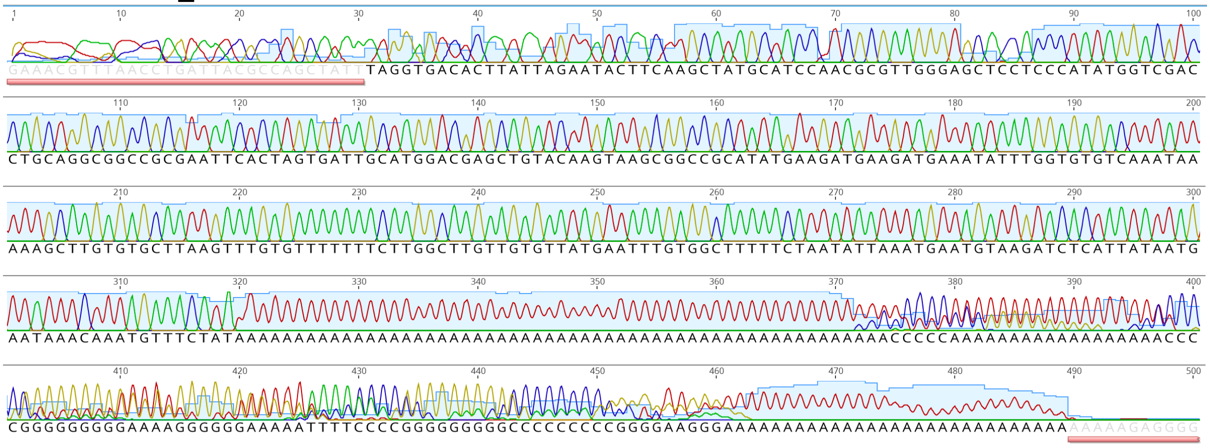

1D6BHAA001_E1.ab1

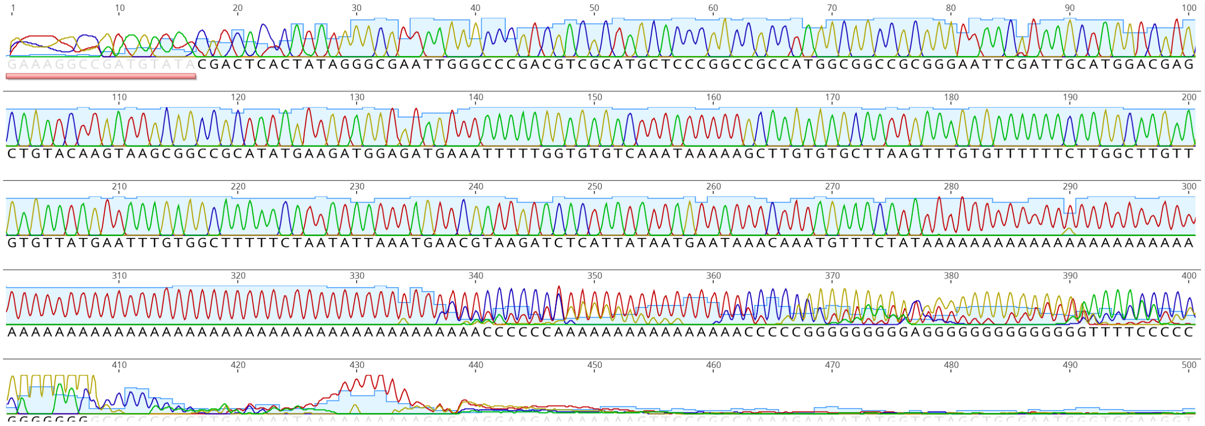

tHSP_5'Δ32

1BABHAA002_A1.ab1

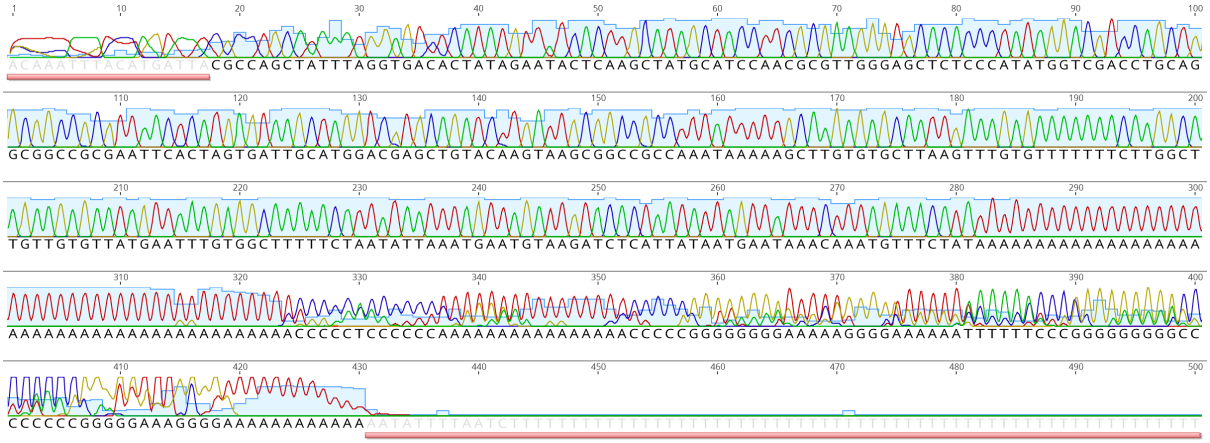

1BABHAA002_B1.ab1

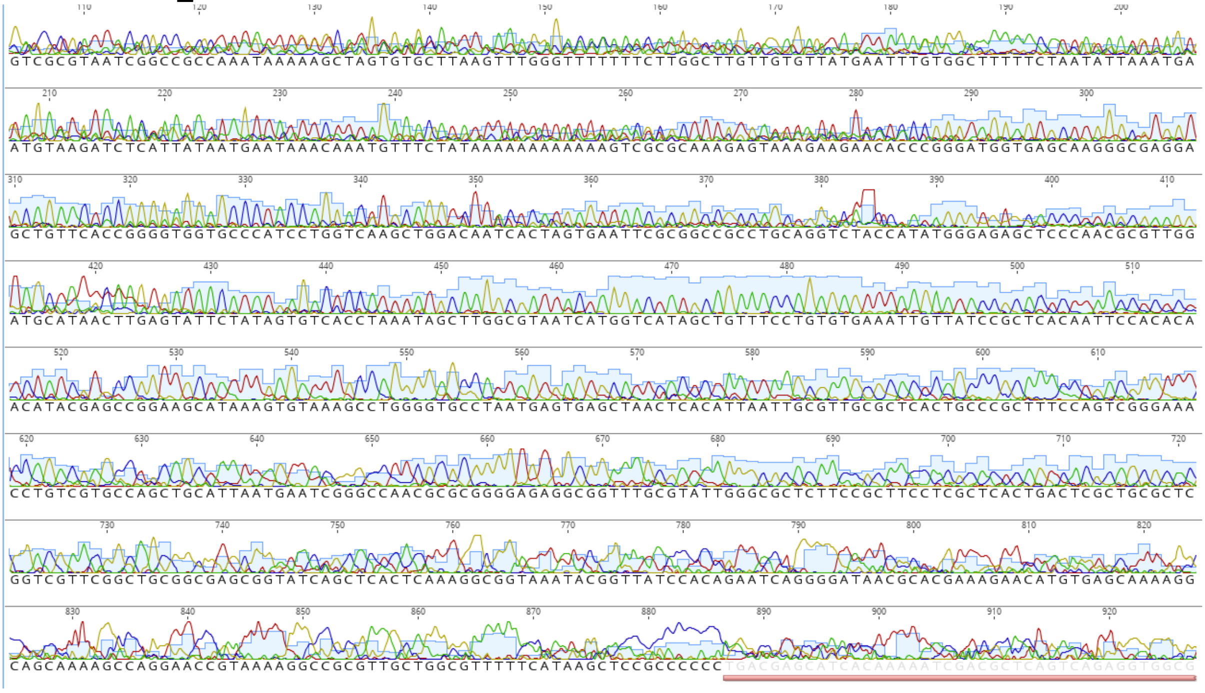

1BABHAA002_C1.ab1

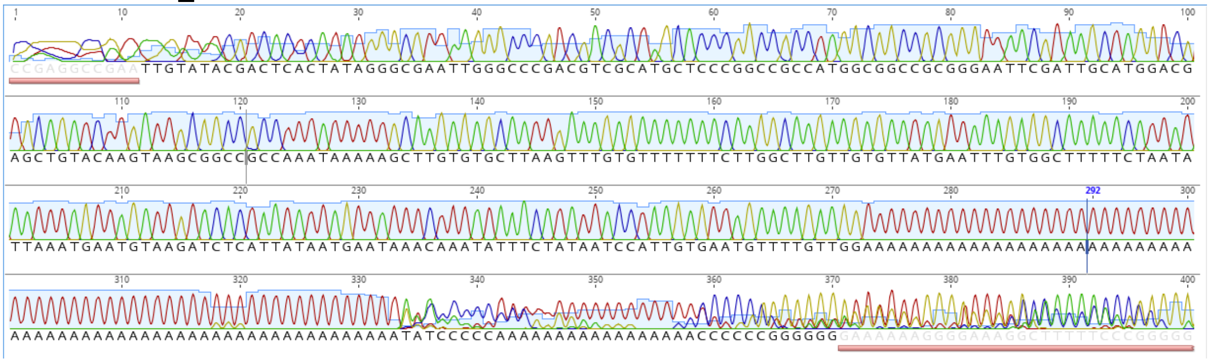

1BABHAA002_D1.ab1

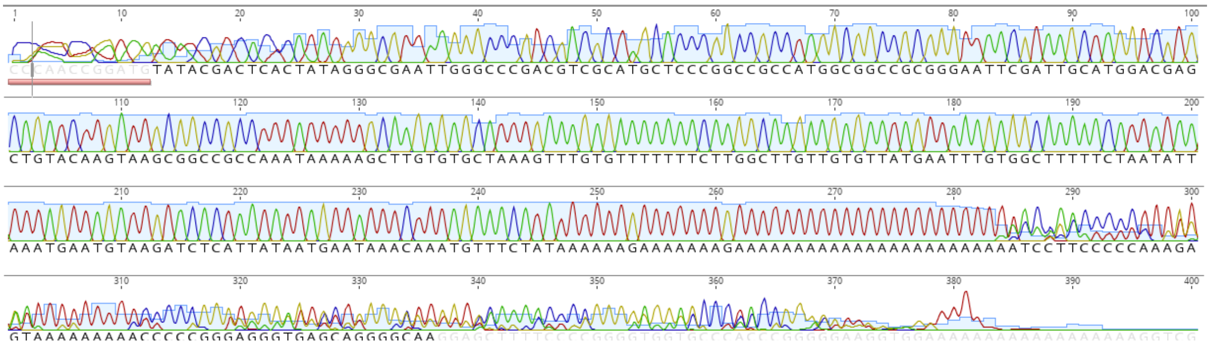

1BABHAA002_E1.ab1

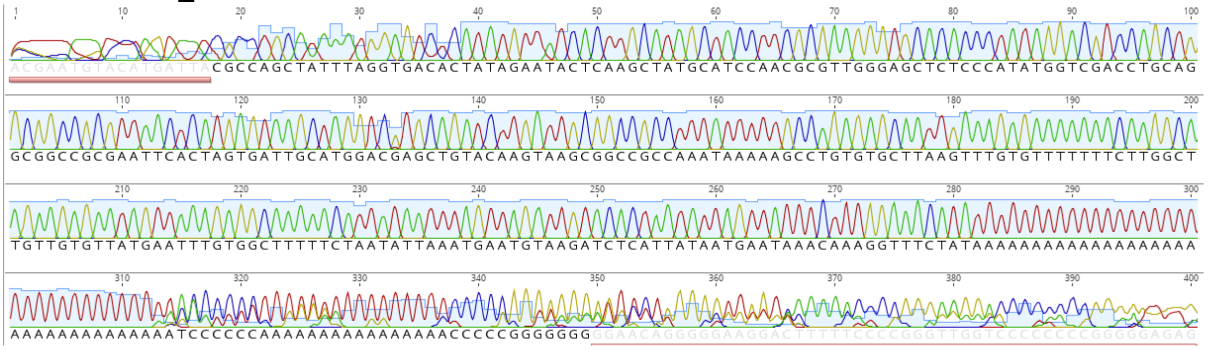

1BABHAA002_F1.ab1

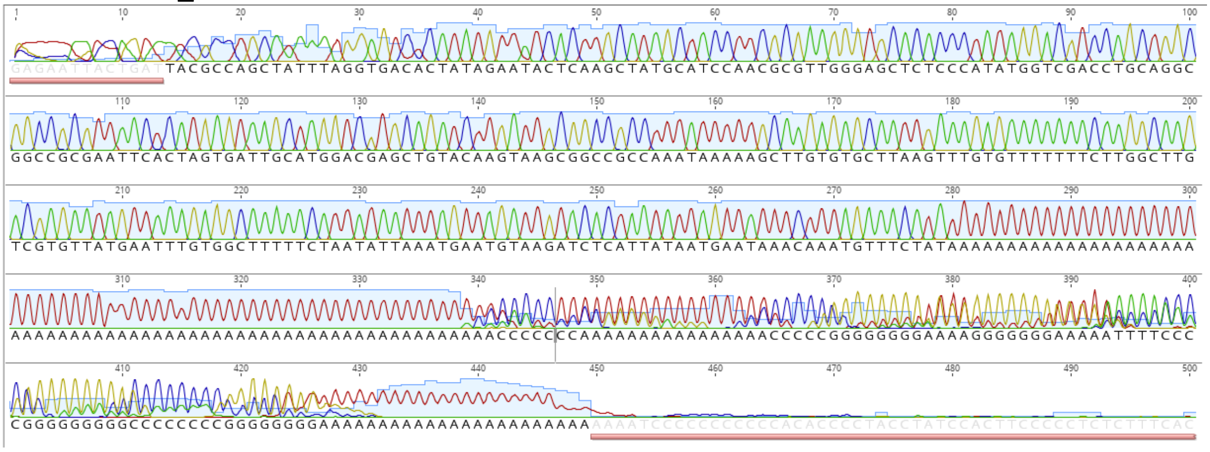

1BABHAA002_G1.ab1

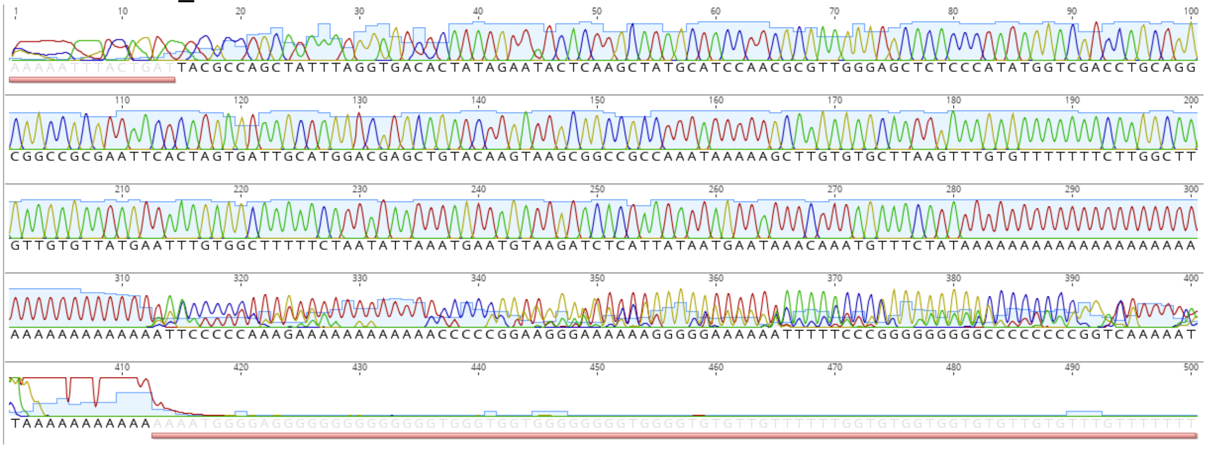

1BABHAA002_H1.ab1

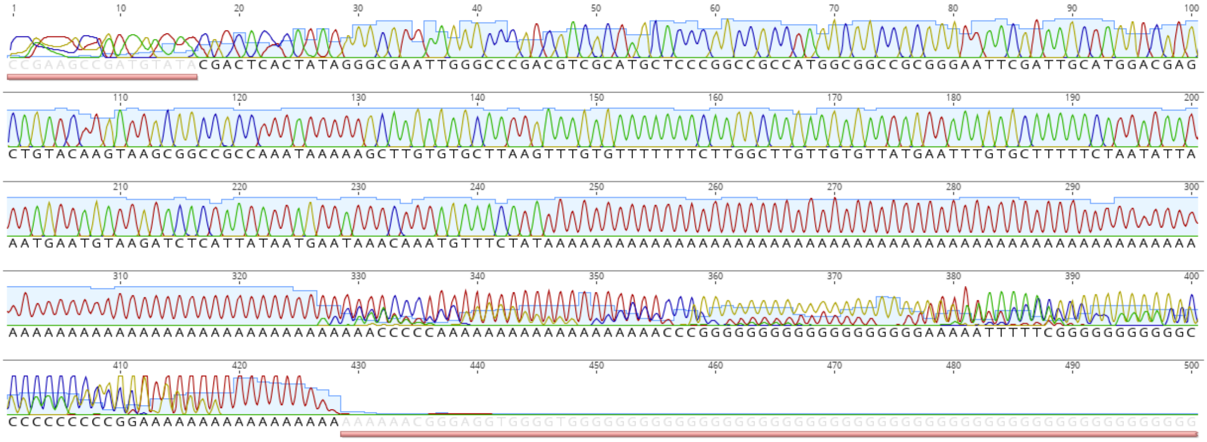

1BABHAA002_B2.ab1

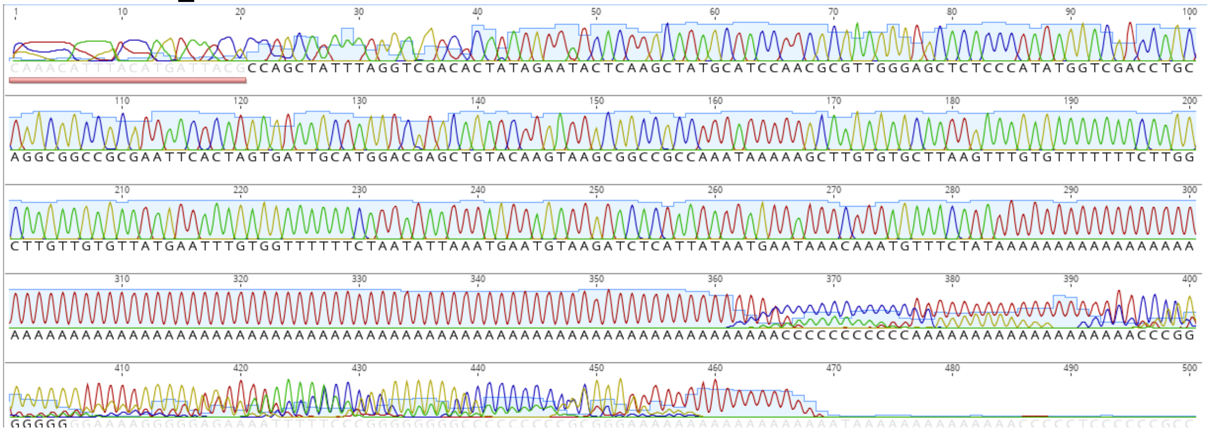

1BABHAA002_C2.ab1

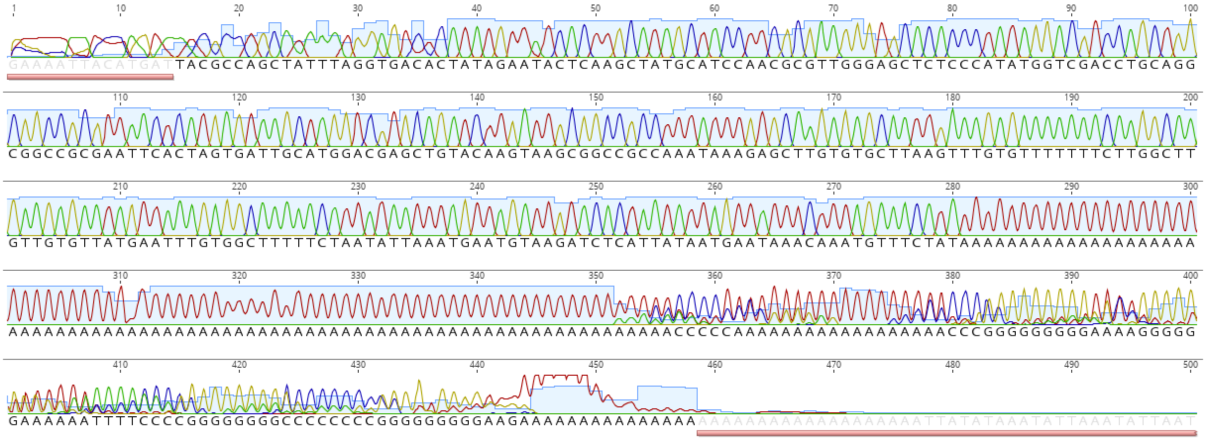

1D6BHAA001_E2.ab1

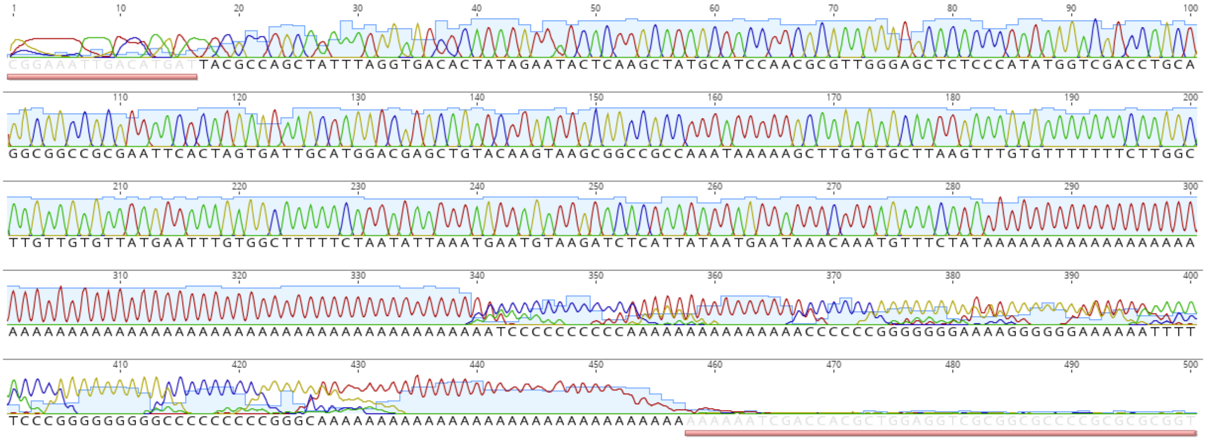

1D6BHAA001_F2.ab1

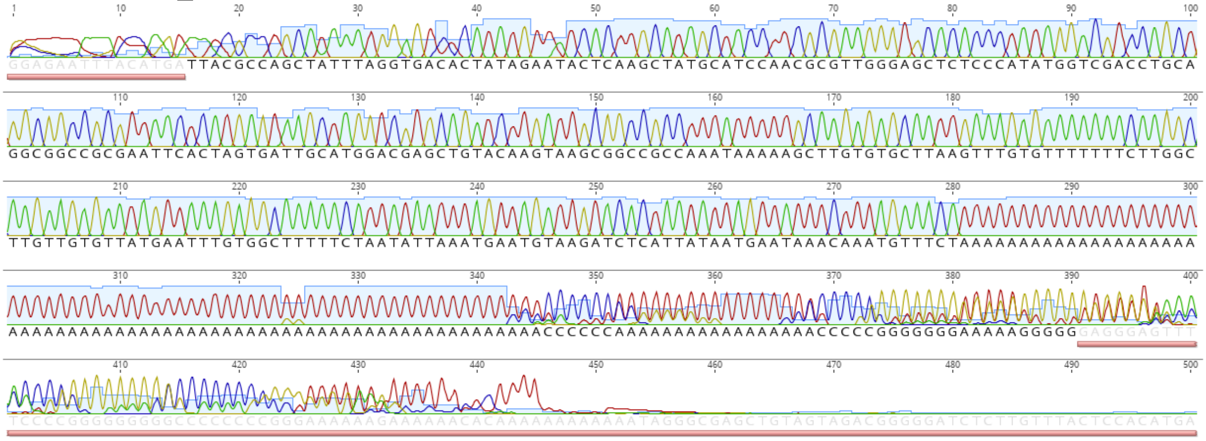

1D6BHAA001_G2.ab1

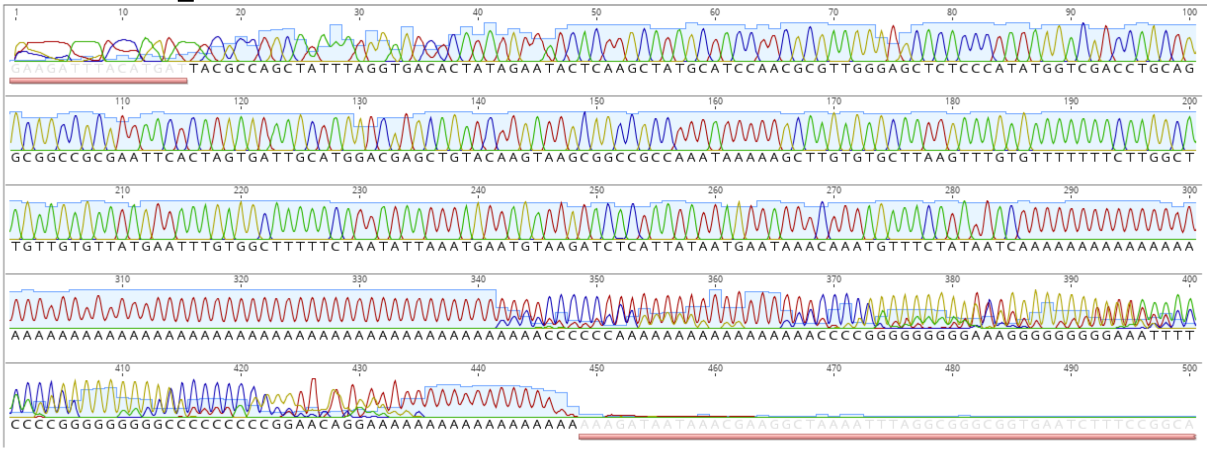

1D6BHAA001_H2.ab1

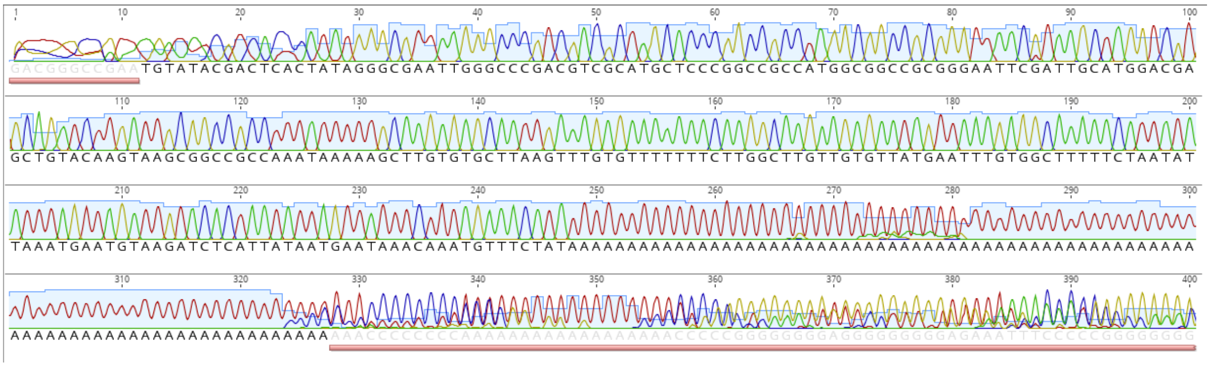

tACS2

1EA6HAB000_A01_premix.ab1

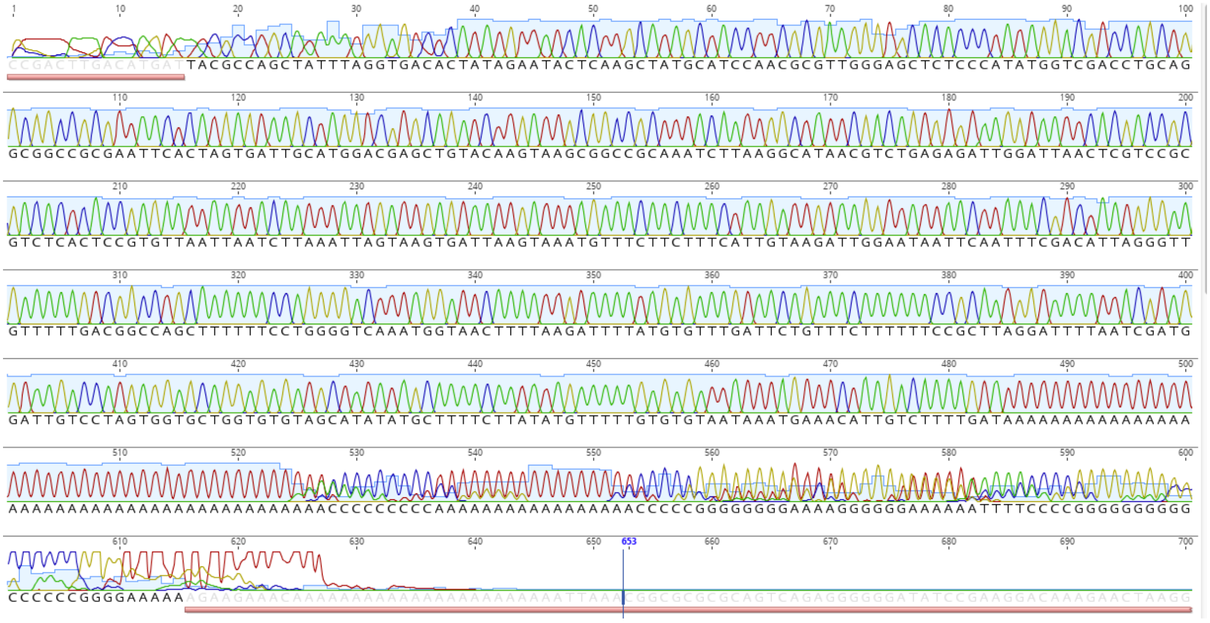

1EA6HAB000_B01_premix.ab1

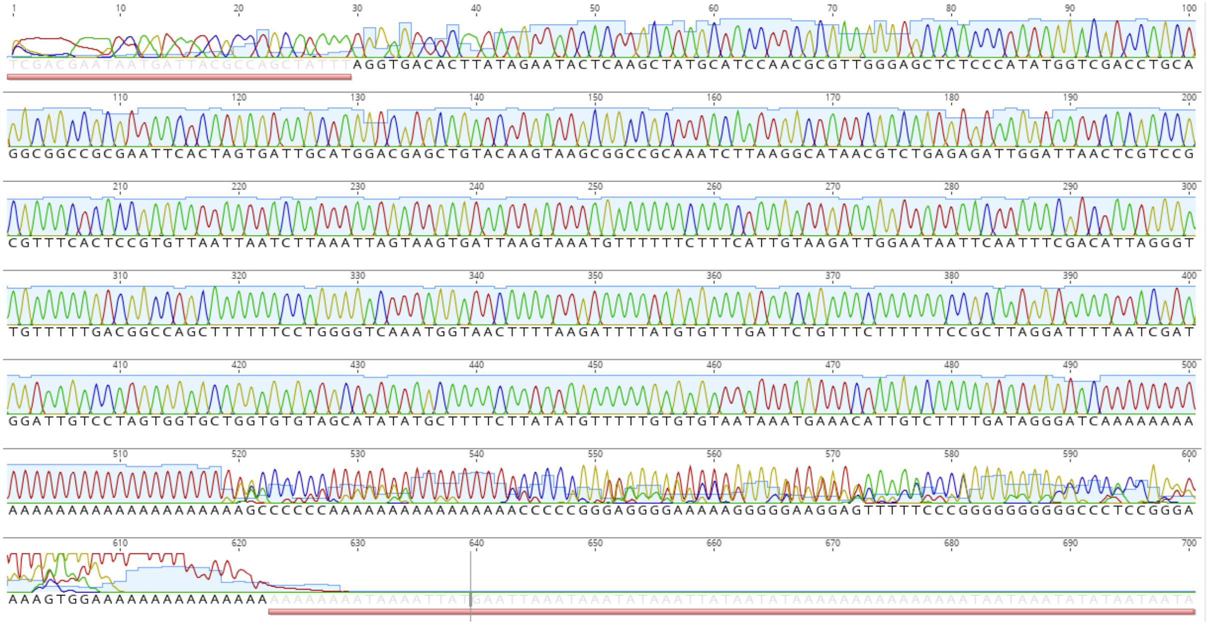

1EA6HAB000_C01_premix.ab1

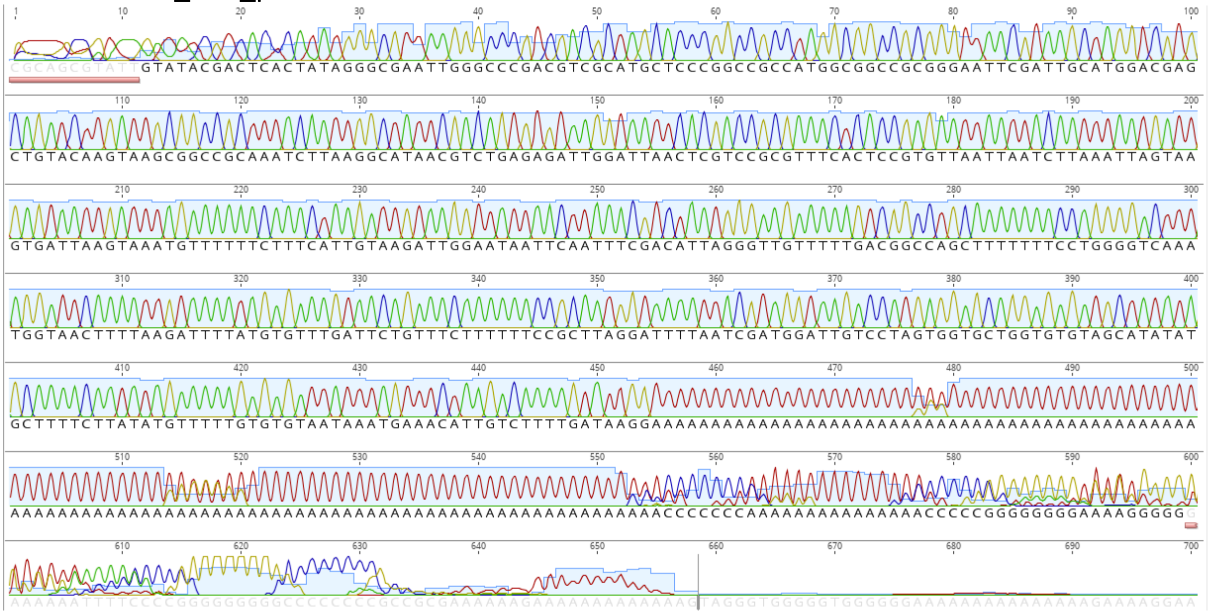

1EA6HAB000_D01_premix.ab1

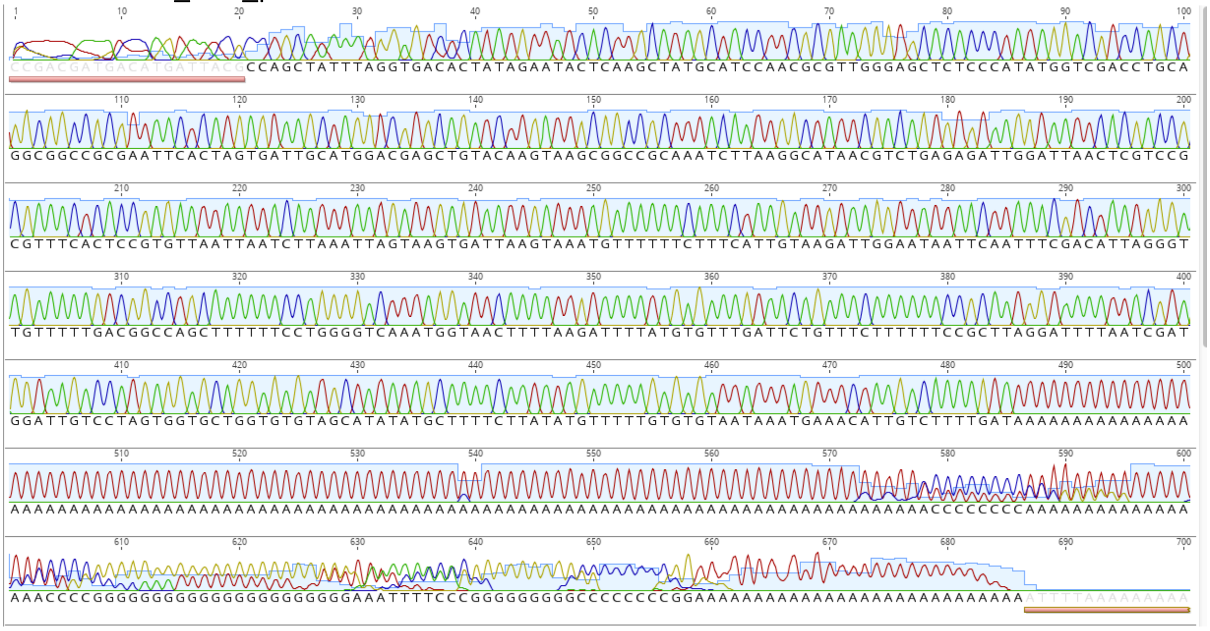

1EA6HAB000_E01_premix.ab1

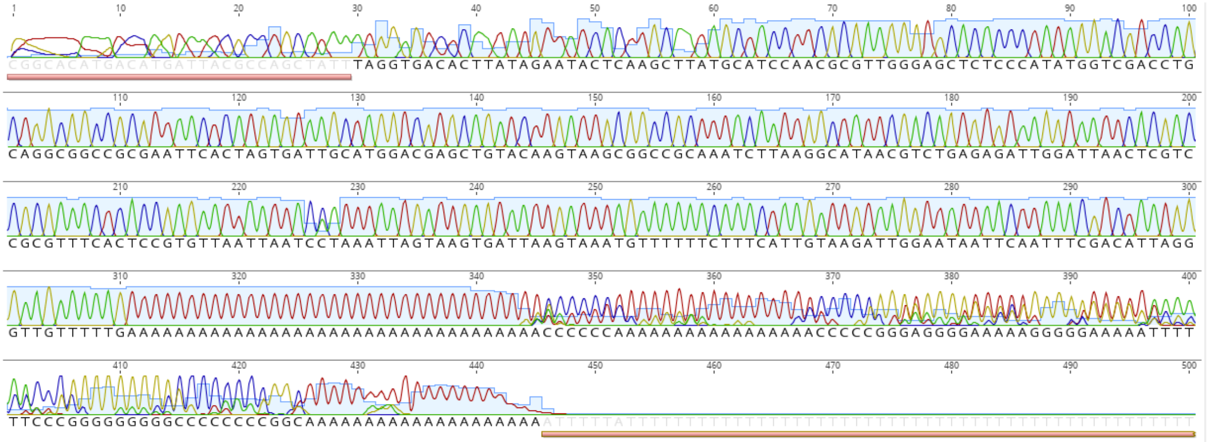

1EA6HAB000_F01_premix.ab1

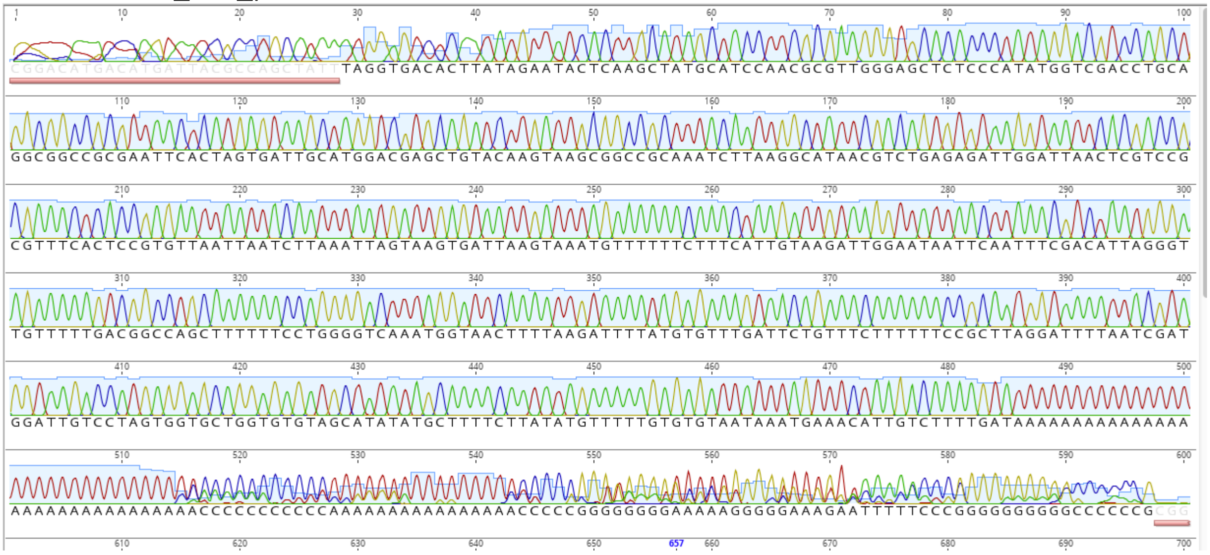

1EA6HAB000_H01_premix.ab1

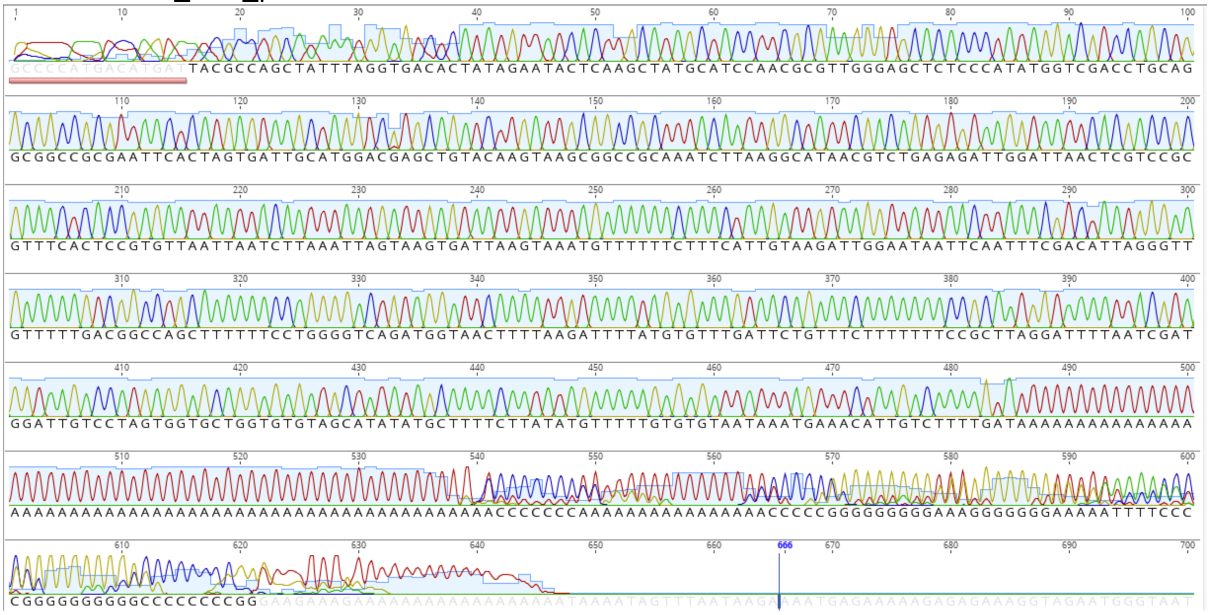

1EA6SAA000_G04_premix.ab1

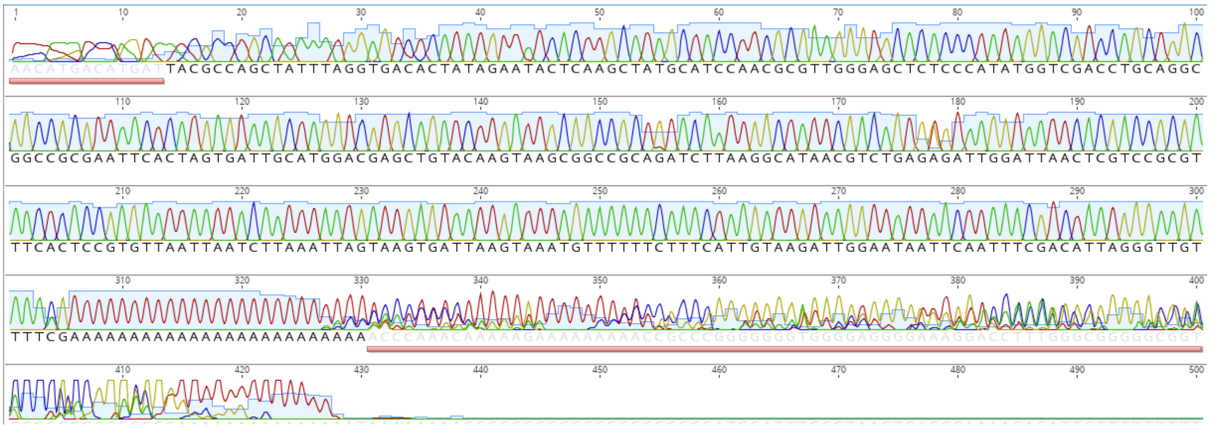

1EA6SAA000_A05_premix.ab1

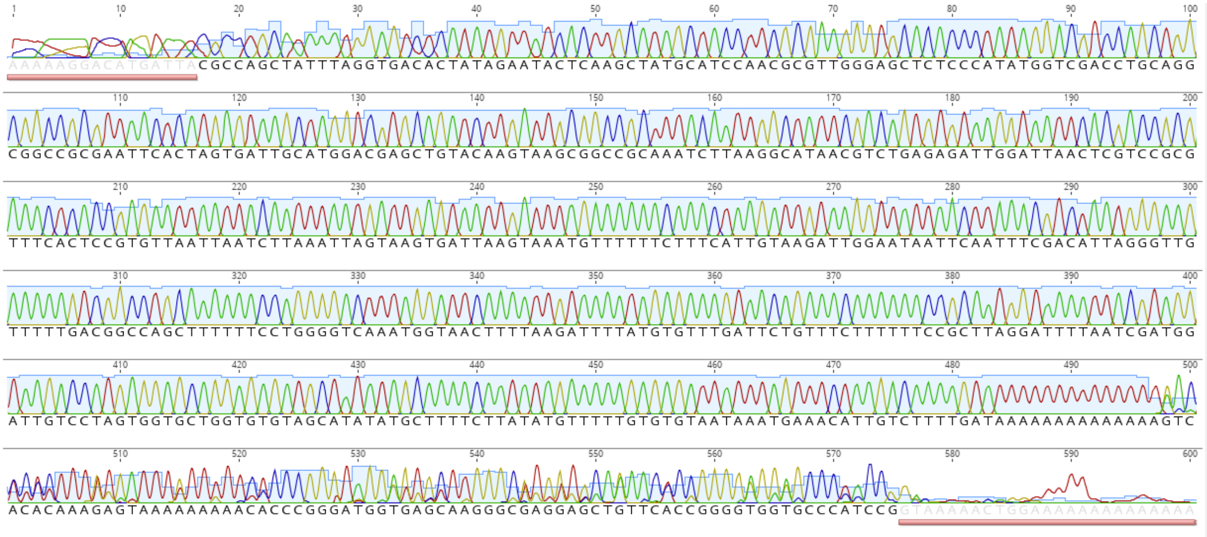

1EA6SAA000_B05_premix.ab1

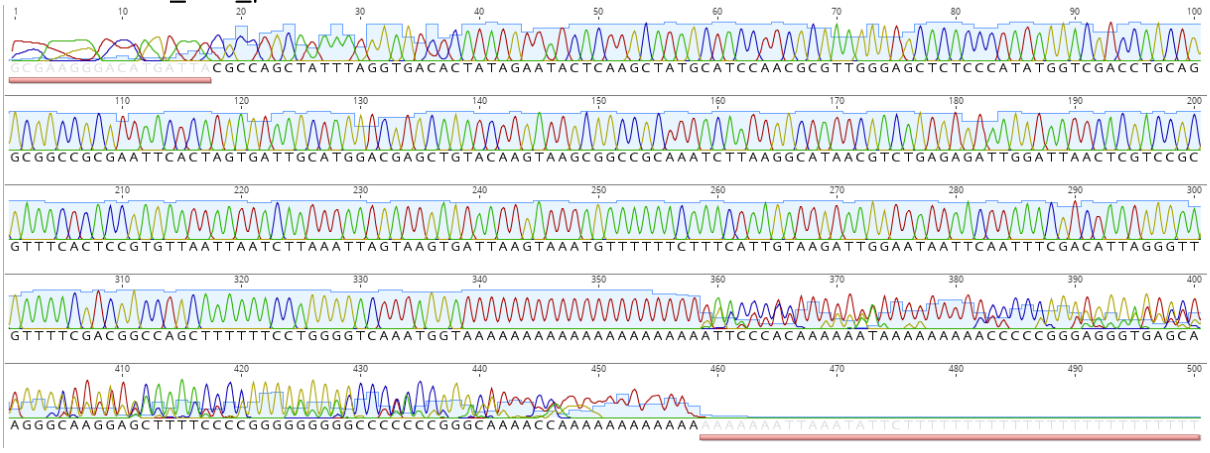

1EA6SAA000_C05_premix.ab1

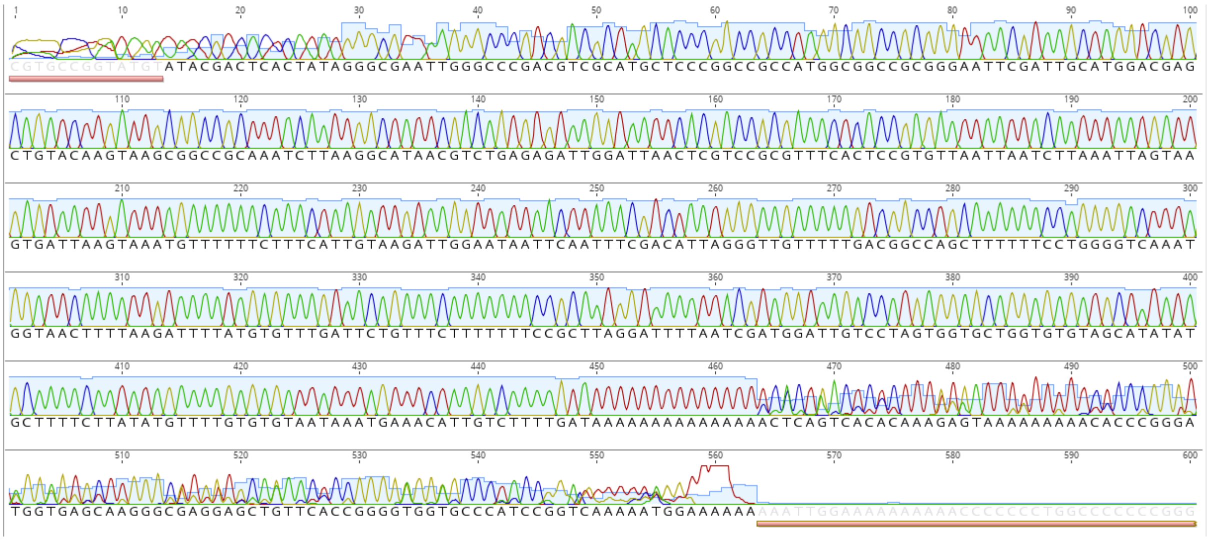

1EA6SAA000_D05_premix.ab1

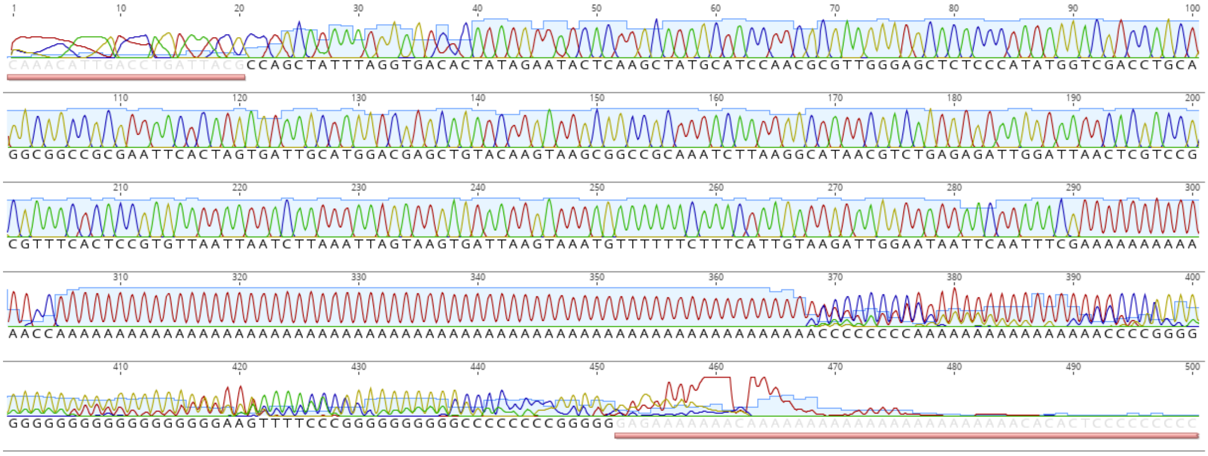

1EA6SAA000_E05_premix.ab1

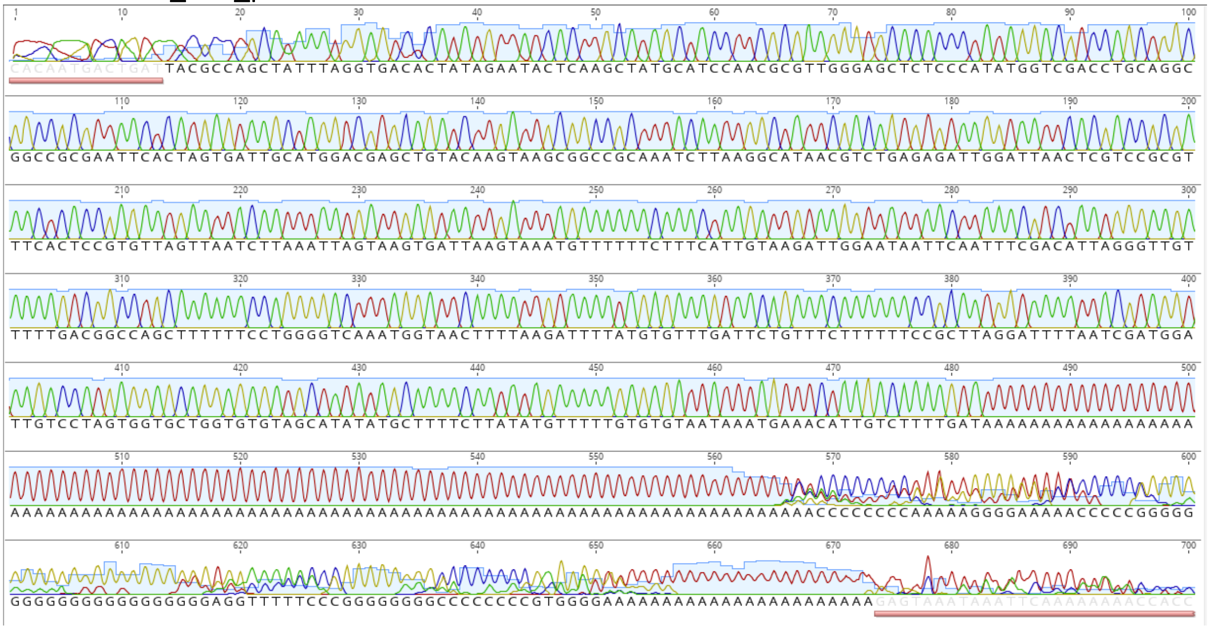

1EA6SAA000_F05_premix.ab1

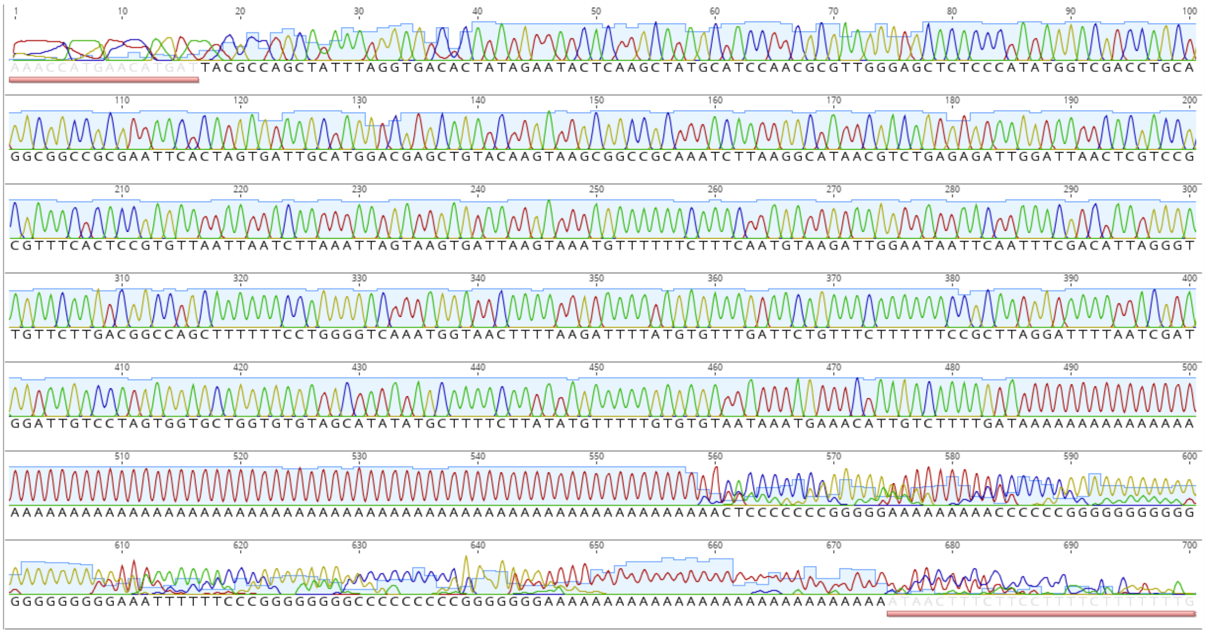

1EA6SAA000_G05_premix.ab1

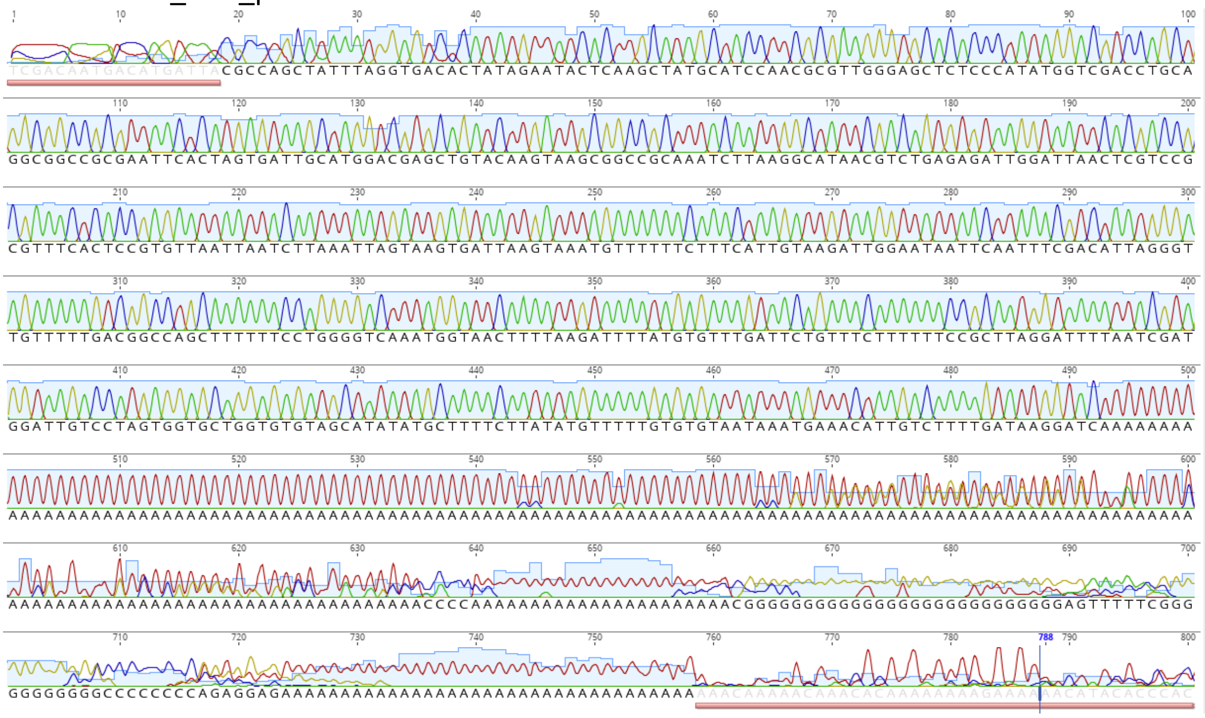

1EA6SAA000_H05_premix.ab1

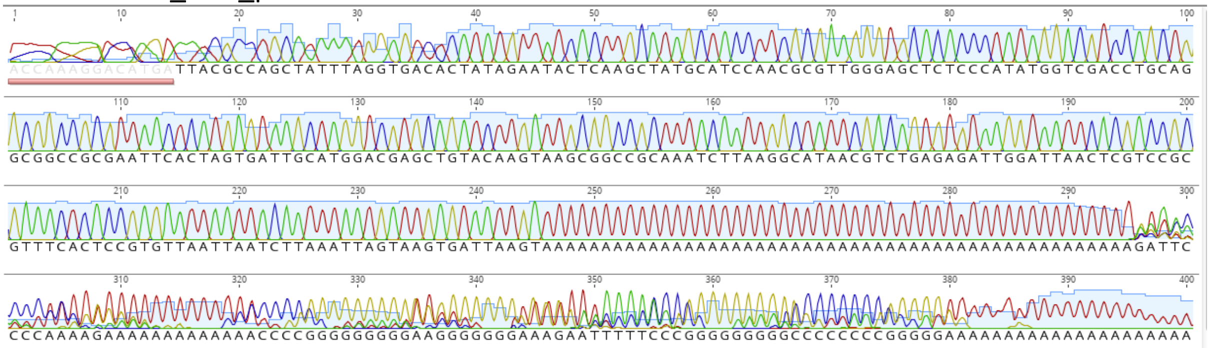

1EA6SAA000_A06_premix.ab1

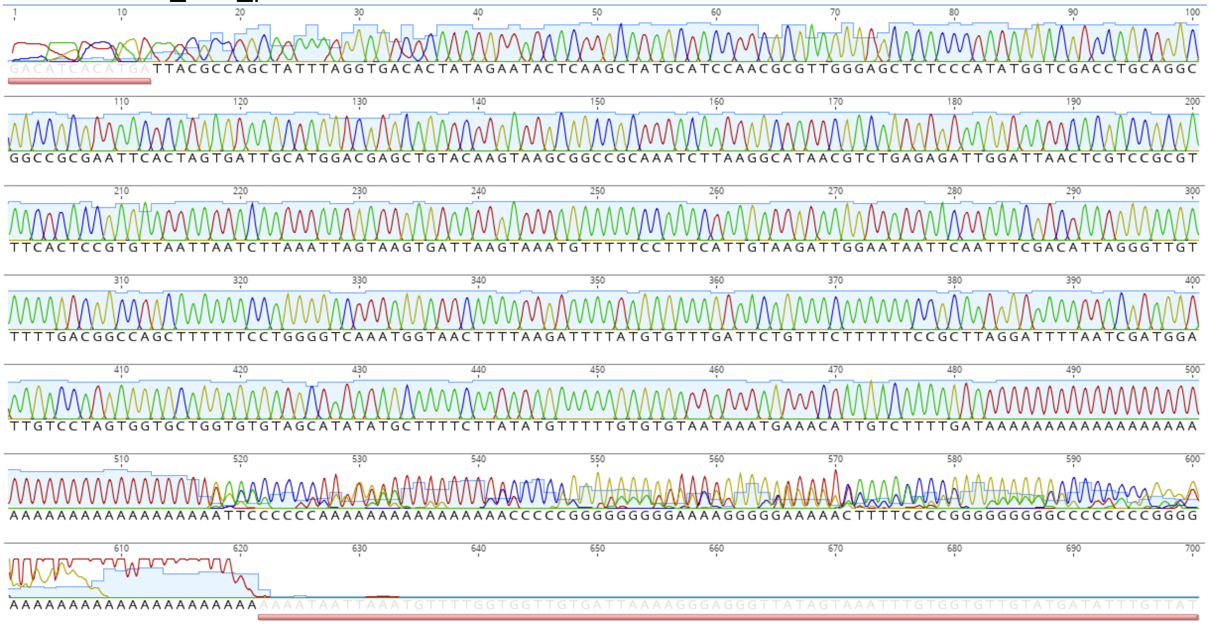

1EA6SAA000_B06_premix.ab1

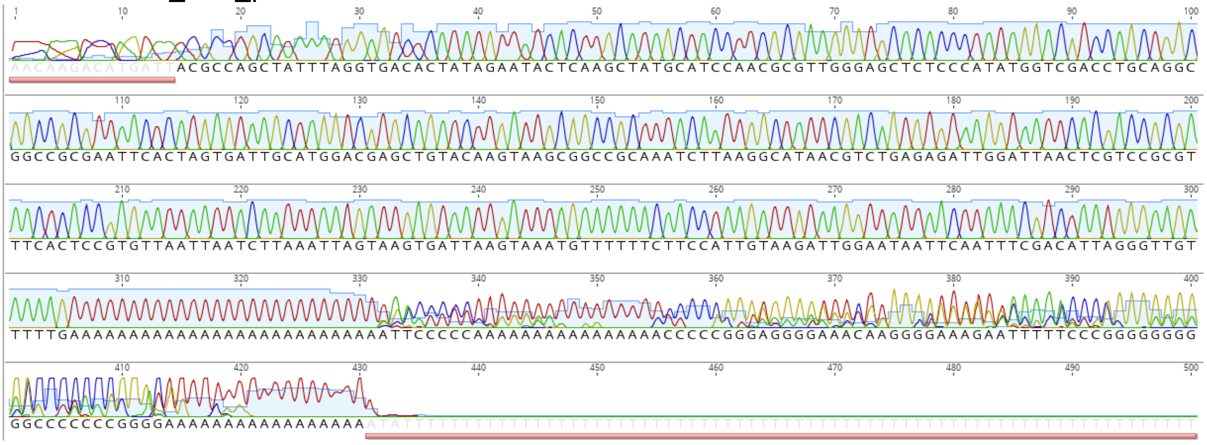

1EA6SAA000_C06_premix.ab1

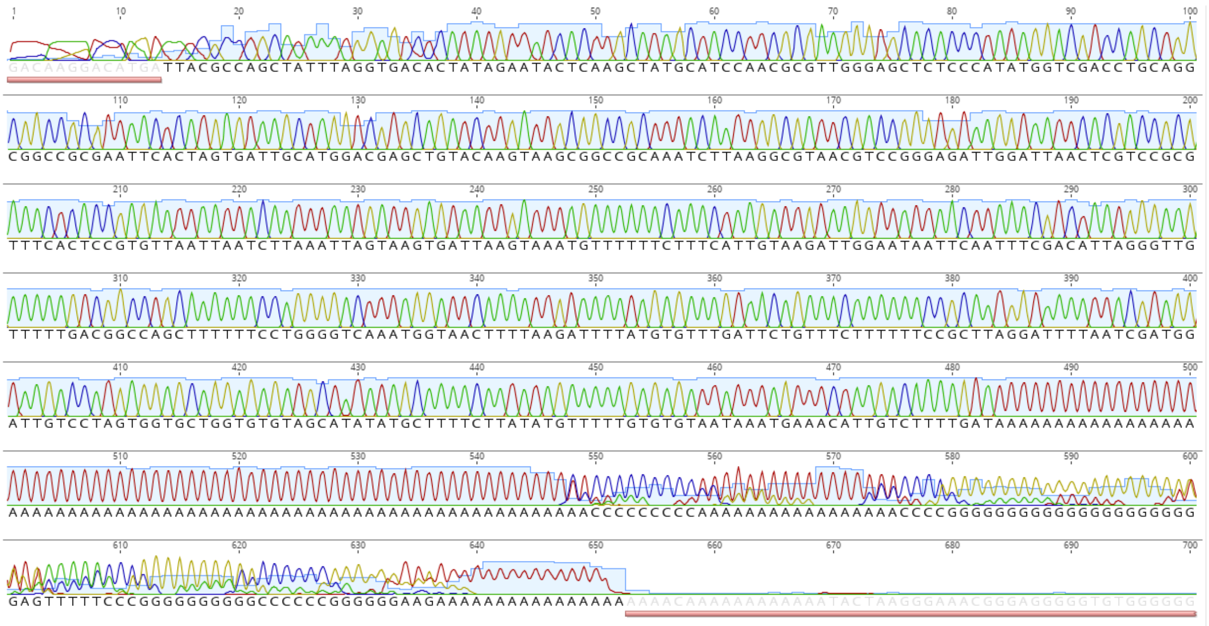

1EA6SAA000_E06_premix.ab1

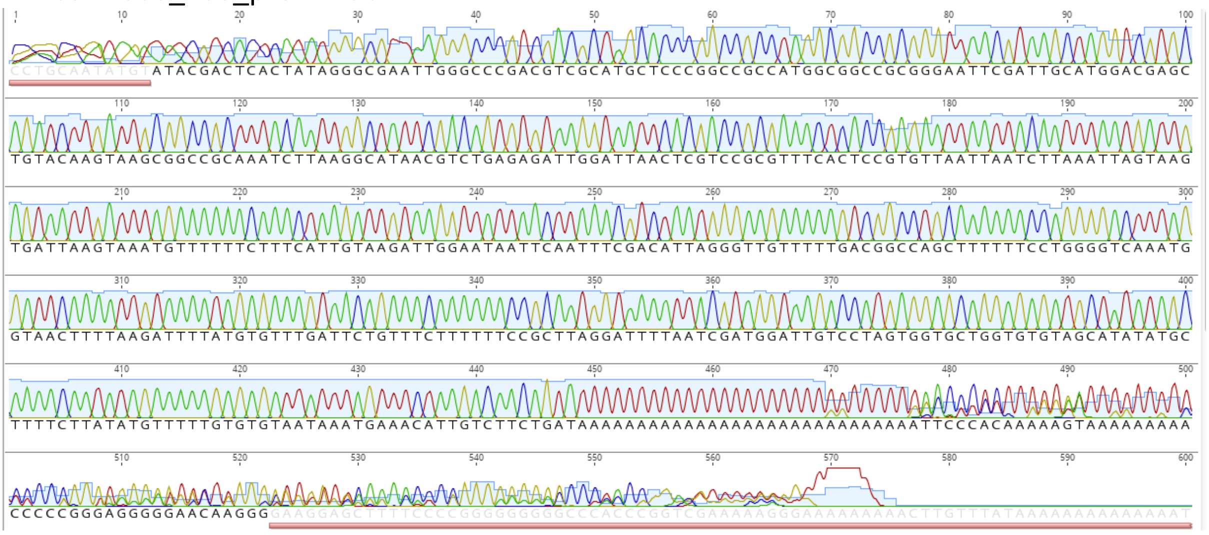

tACS2_tHSP_5'

1EA6HAB000_H03_premix.ab1

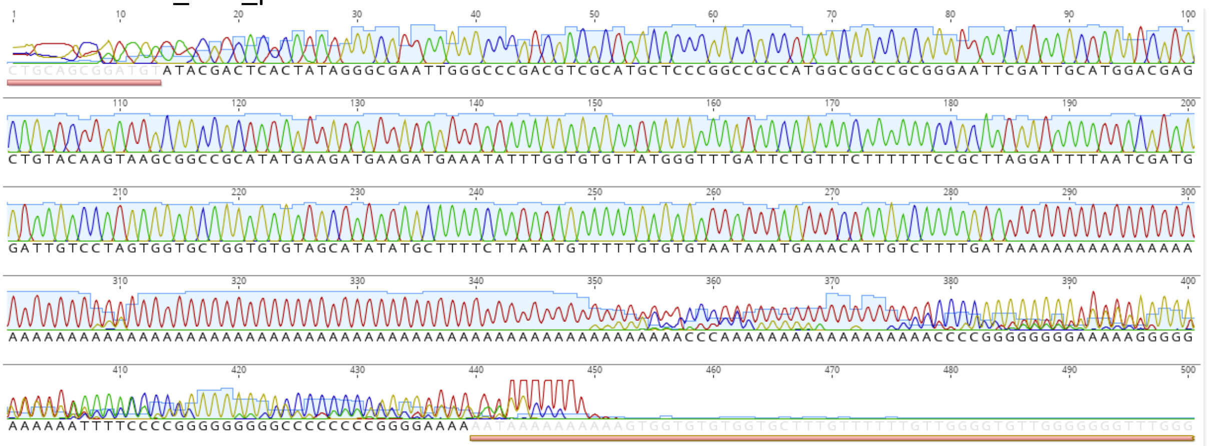

1EA6HAB000_A04_premix.ab1

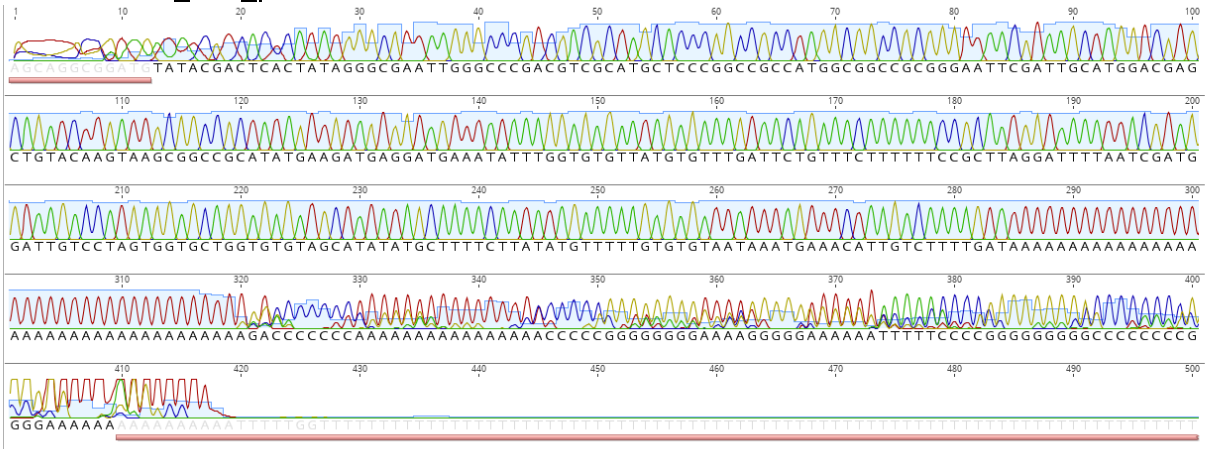

1EA6HAB000_B04_premix.ab1

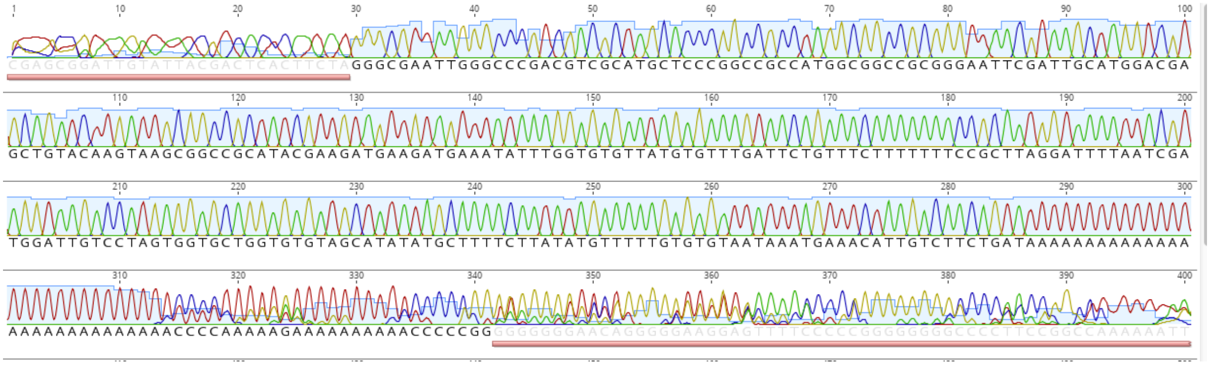

1EA6HAB000_E04_premix.ab1

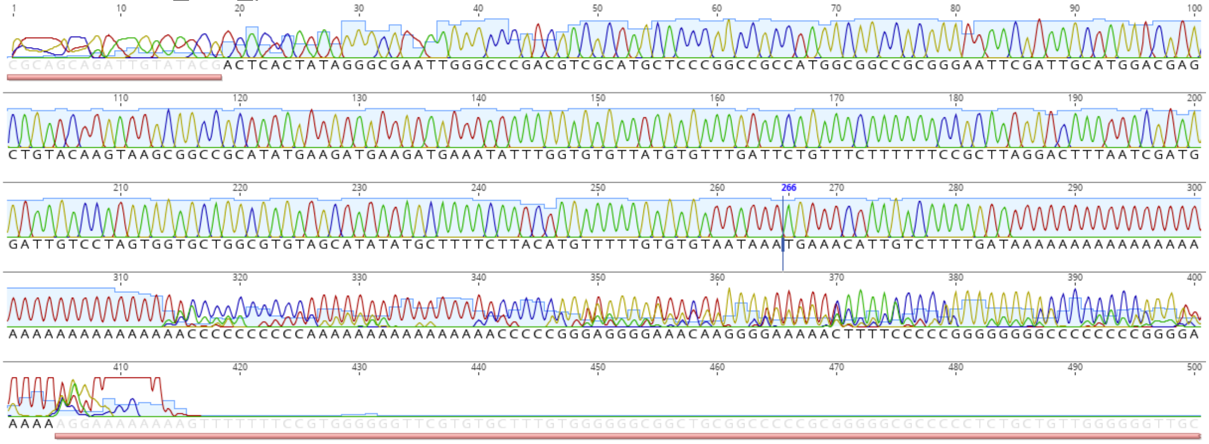

1EA6HAB000_F04_premix.ab1

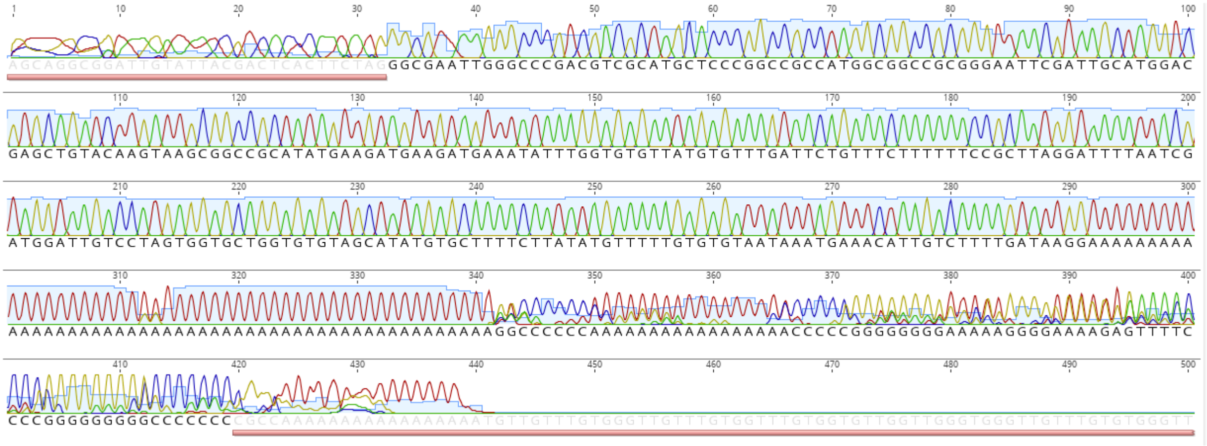

1EA6HAB000_G04_premix.ab1

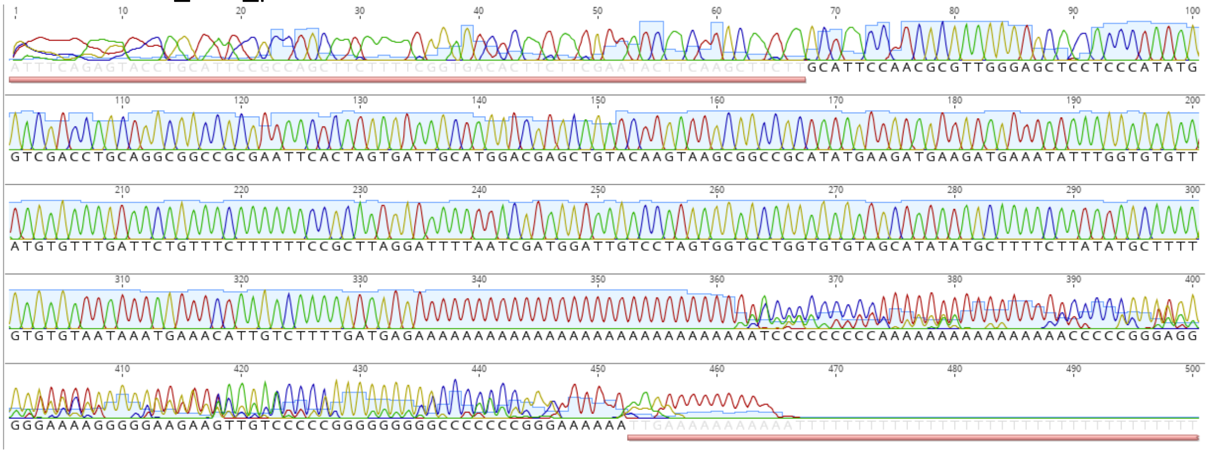

1EA6HAB000_H04_premix.ab1

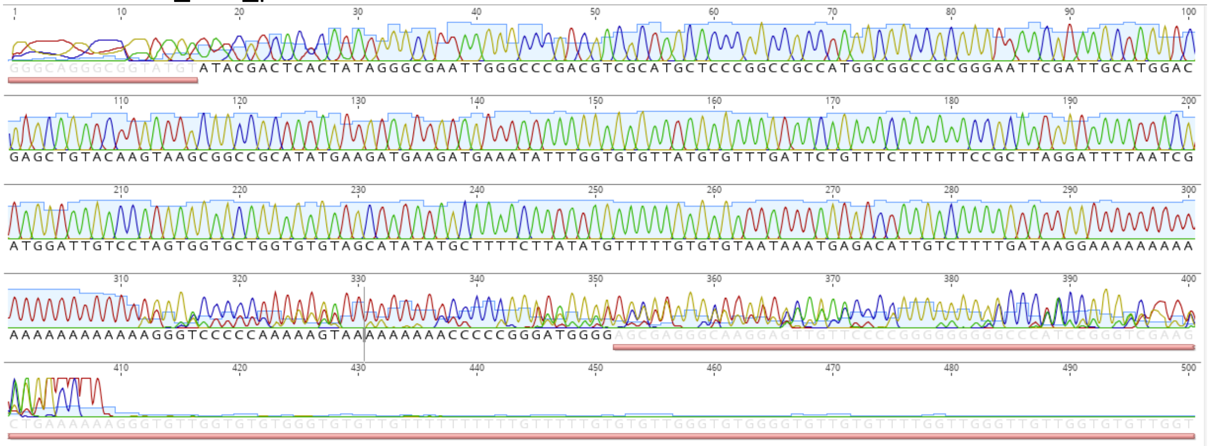

1EA6HAB000_A05_premix.ab1

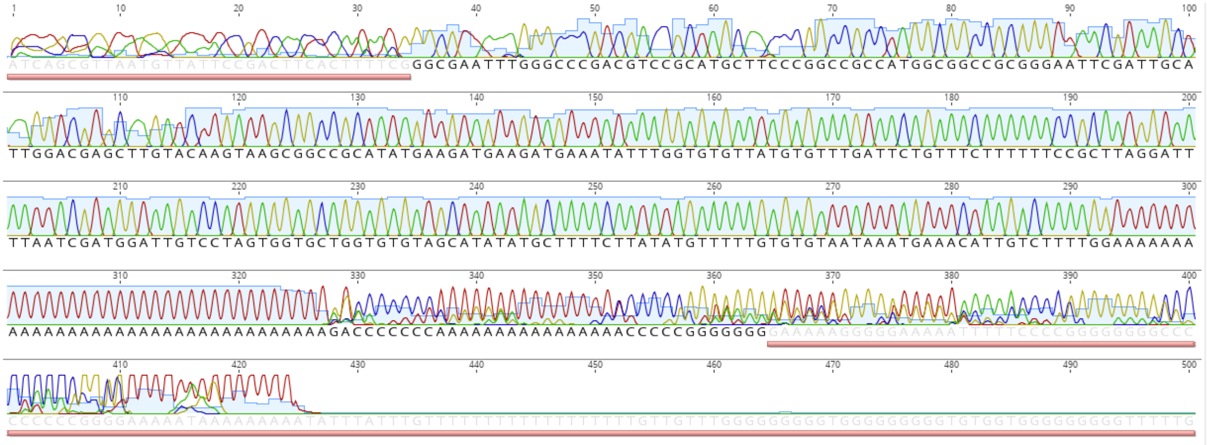

1EA6SAA000_F06_premix.ab1

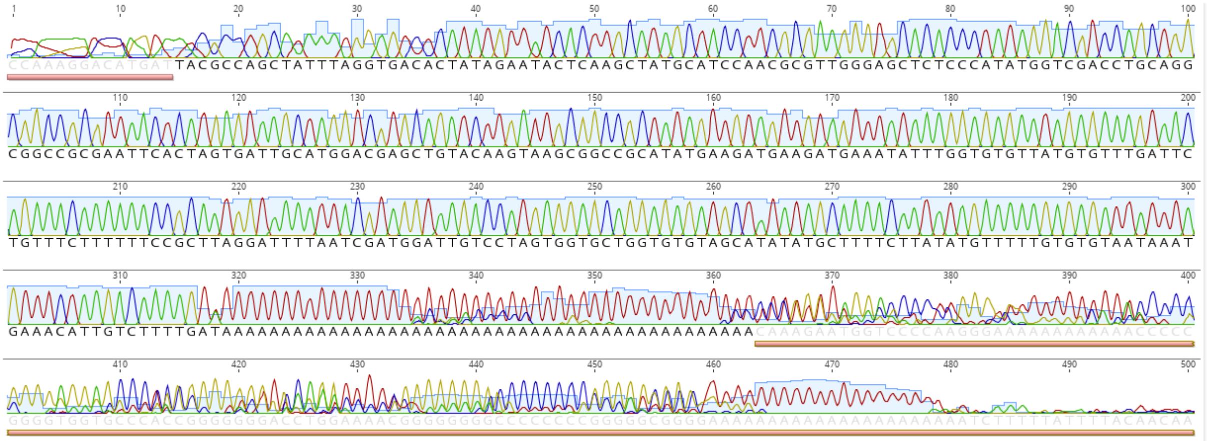

1EA6SAA000_G06_premix.ab1

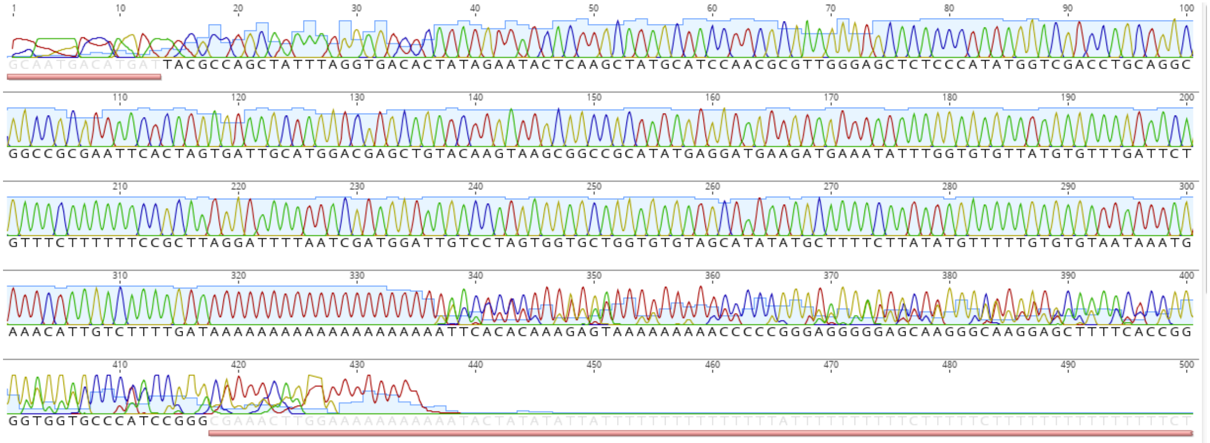

1EA6SAA000_A07_premix.ab1

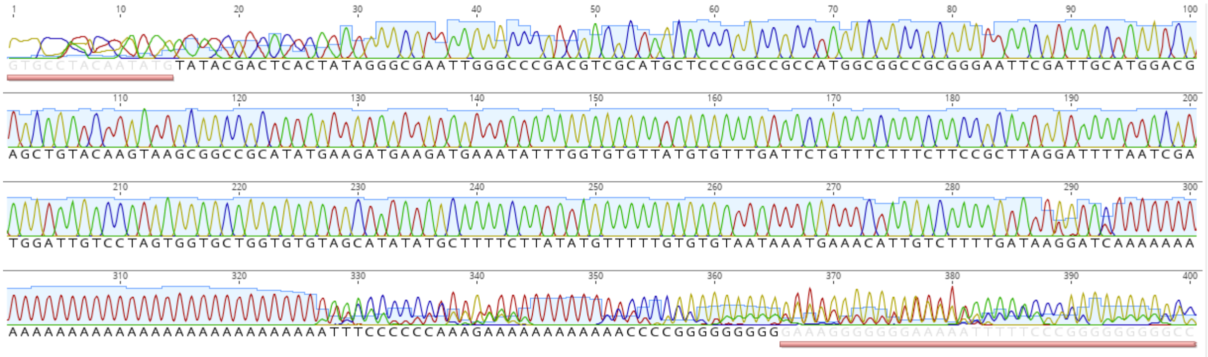

1EA6SAA000_B07_premix.ab1

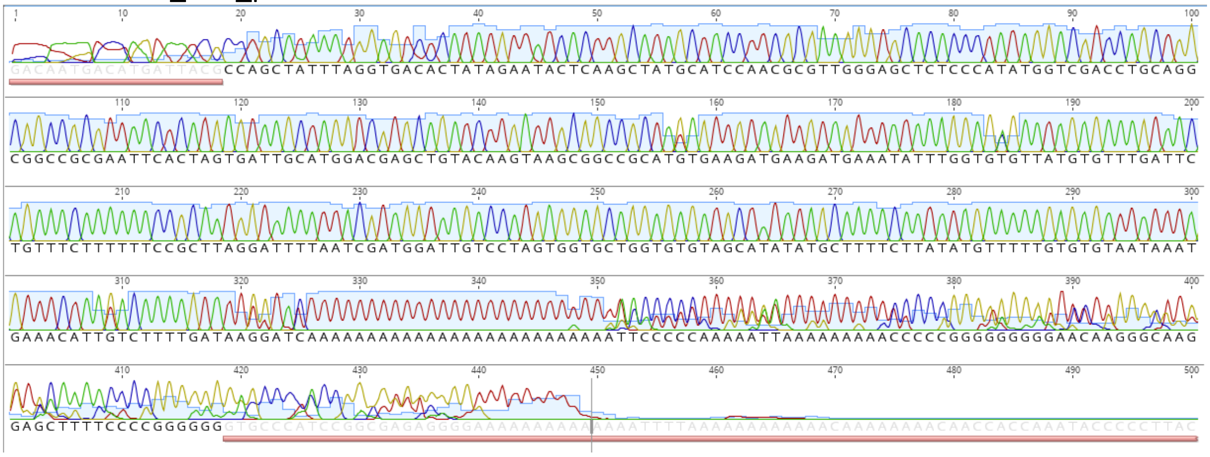

1EA6SAA000_D07_premix.ab1

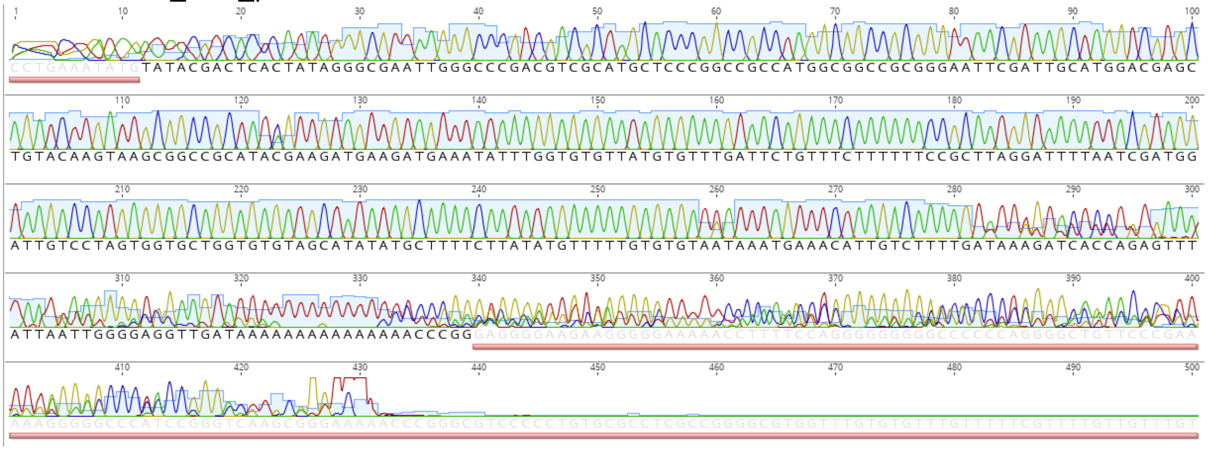

1EA6SAA000_E07_premix.ab1

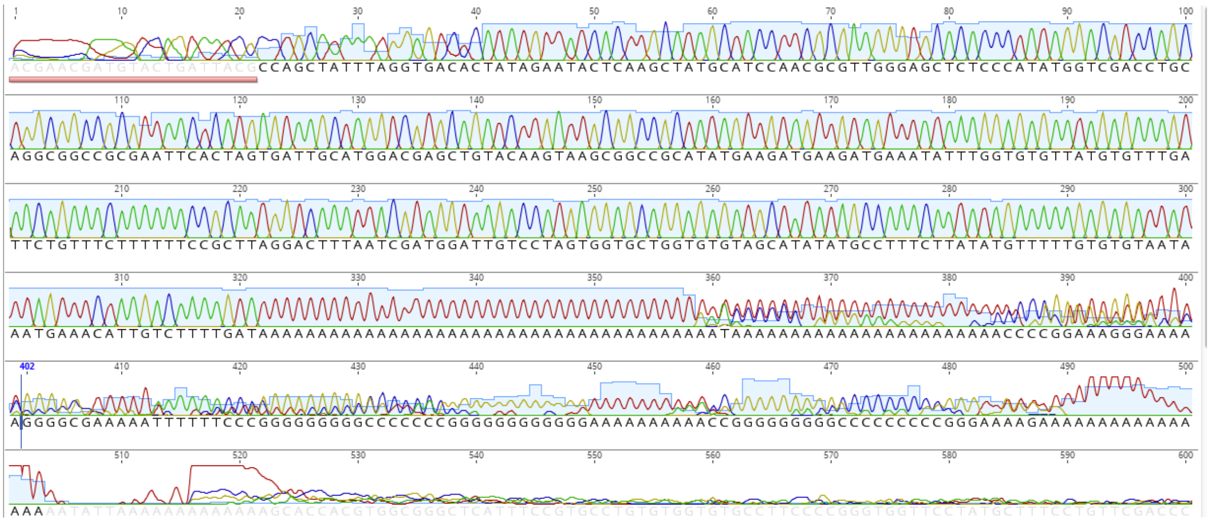

1EA6SAA000_F07_premix.ab1

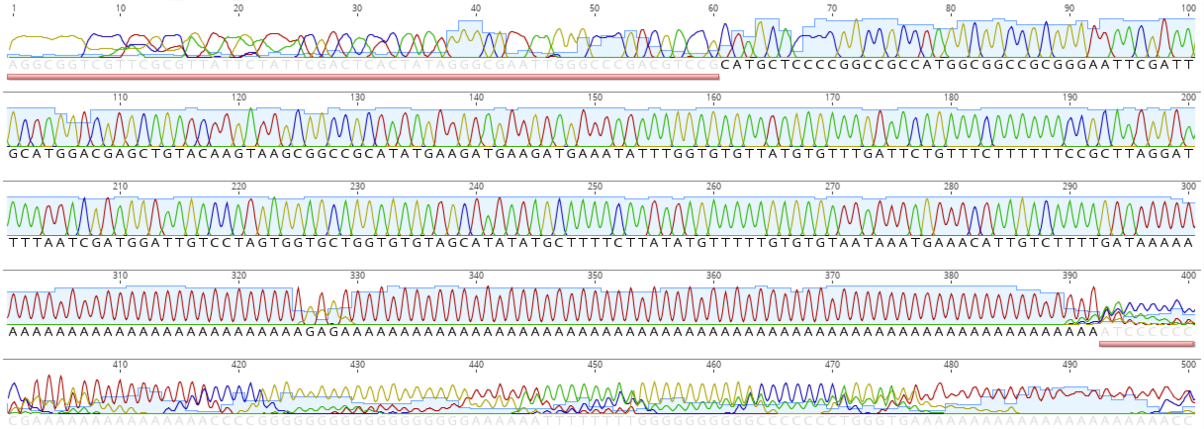

1EA6SAA000_G07_premix.ab1

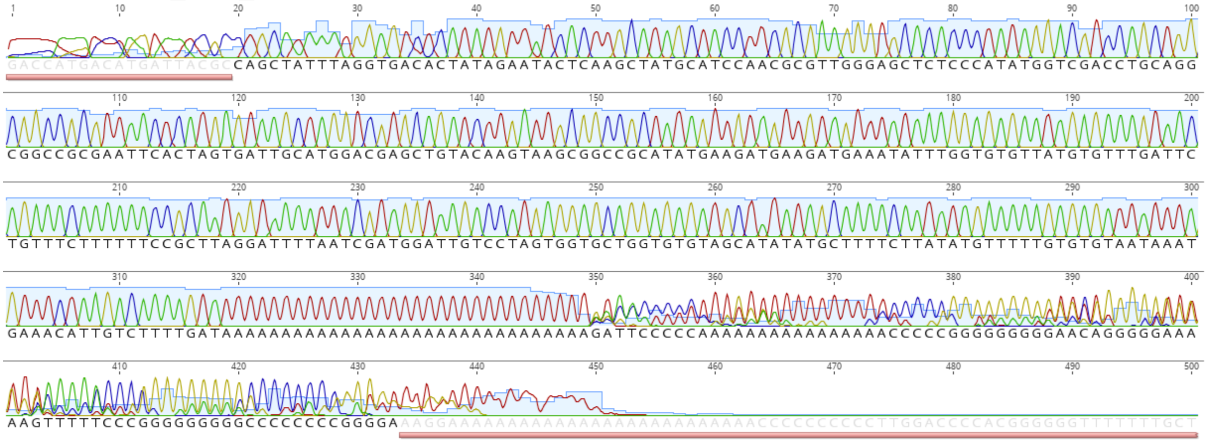

1EA6SAA000_B08_premix.ab1

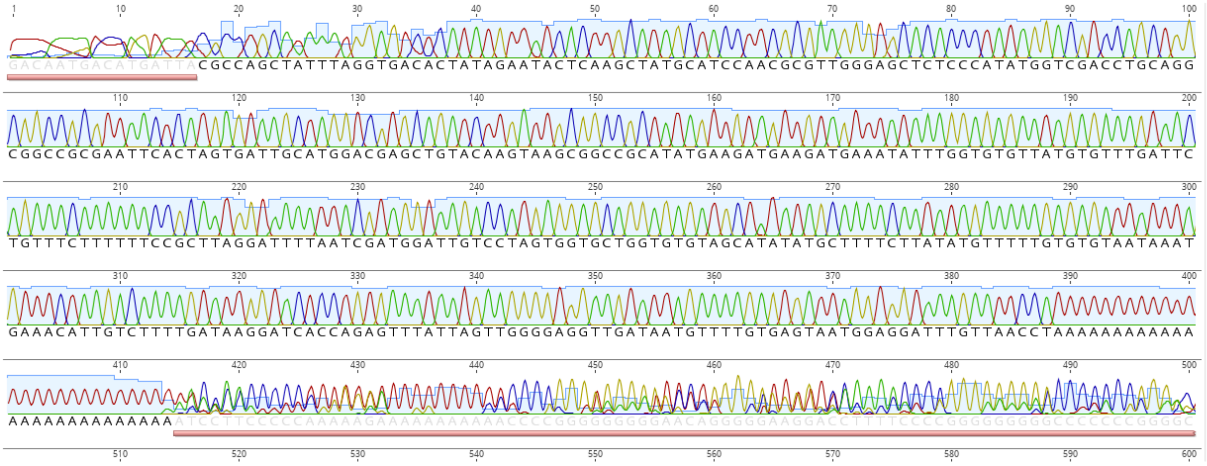

1EA6SAA000_C08_premix.ab1

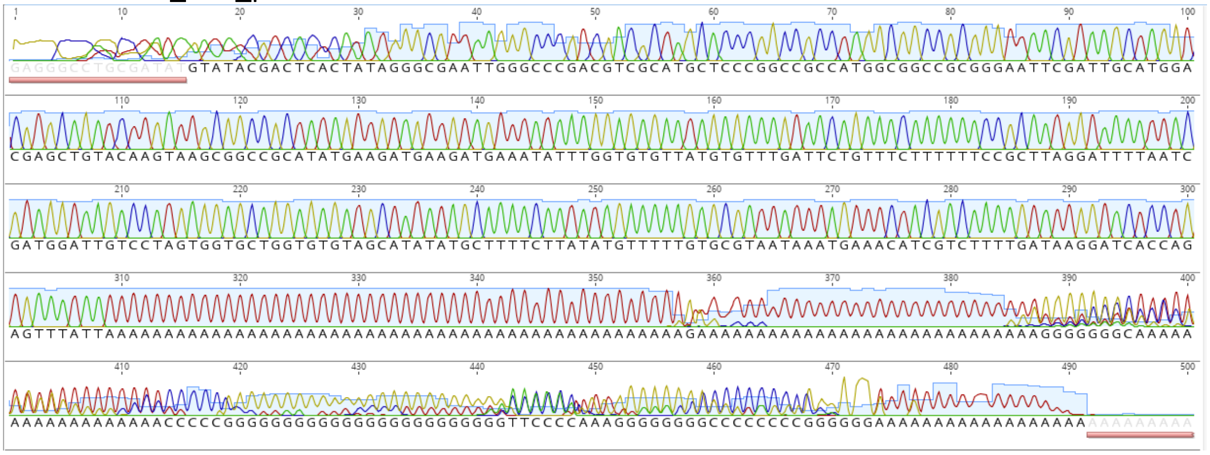

tRBCS

1EA6SAA000_A01_premix.ab1

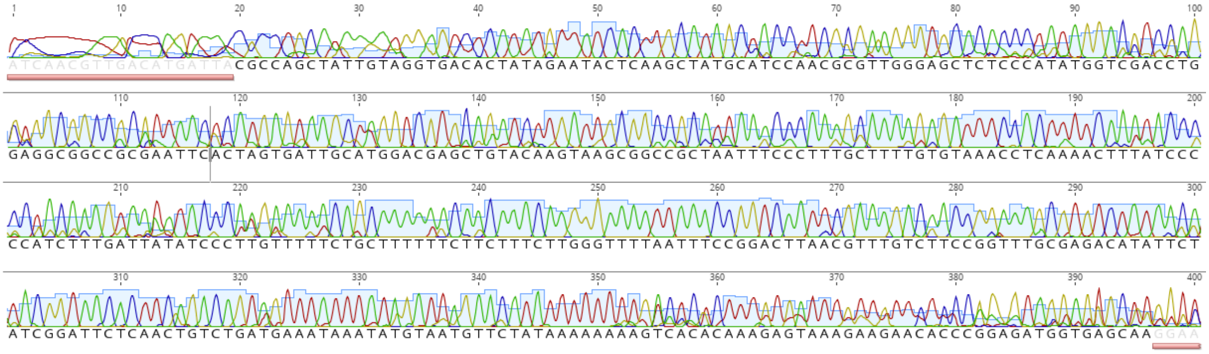

1EA6SAA000_B01_premix.ab1

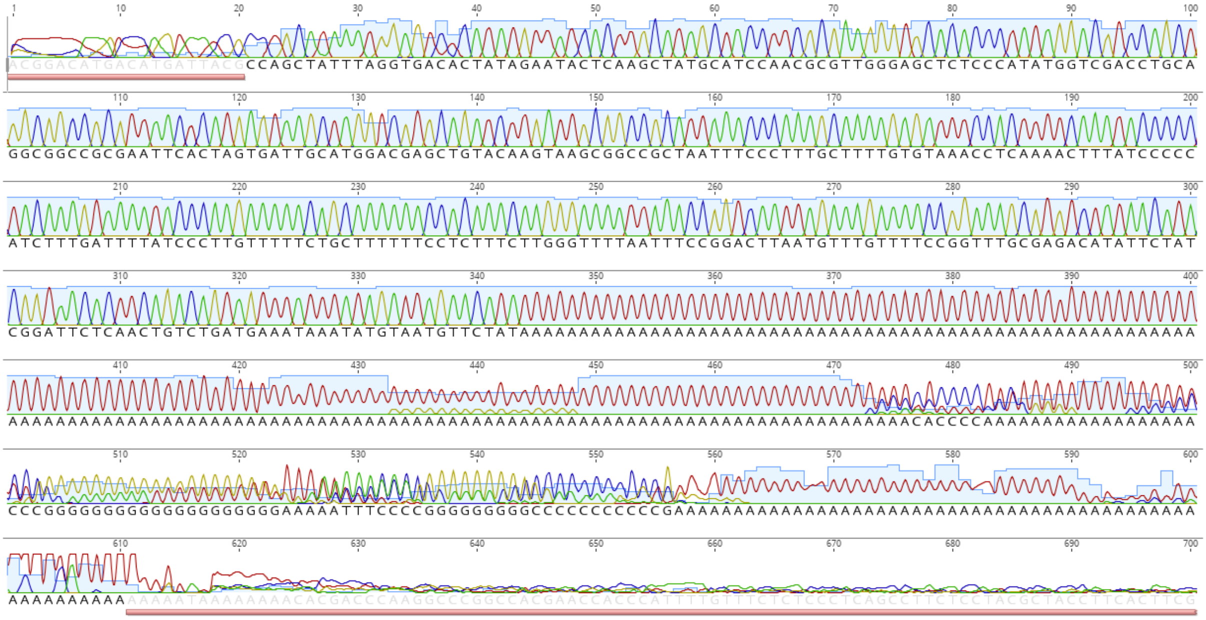

1EA6SAA000_D01_premix.ab1

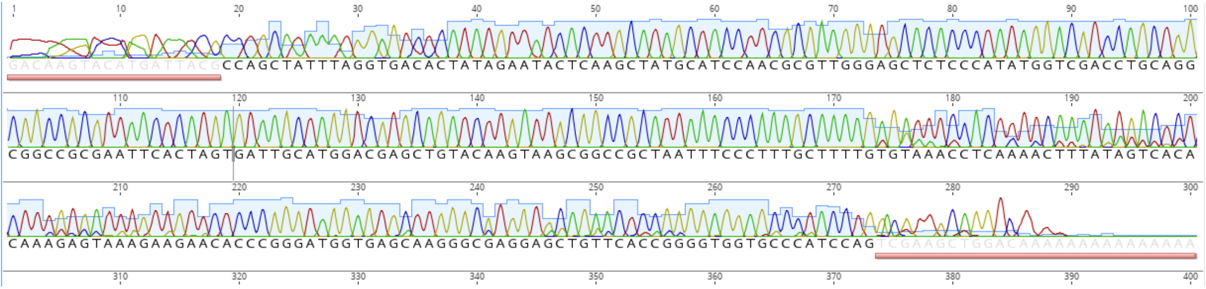

1EA6SAA000_E01_premix.ab1

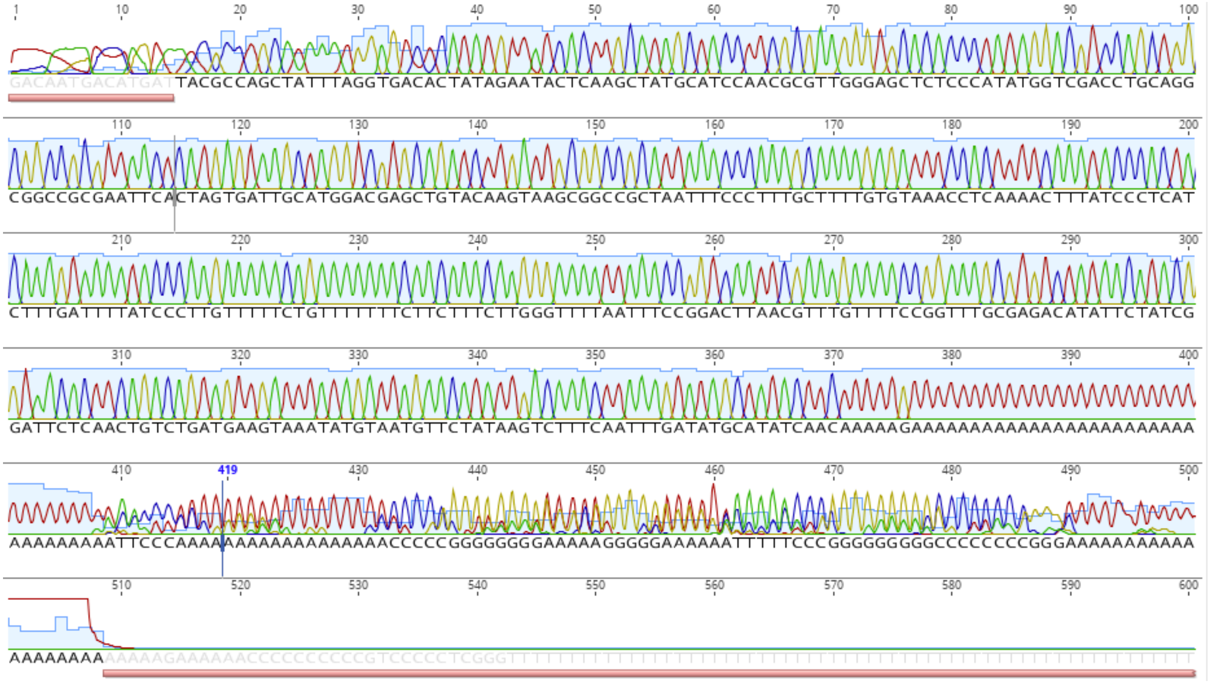

1EA6SAA000_E02_premix.ab1

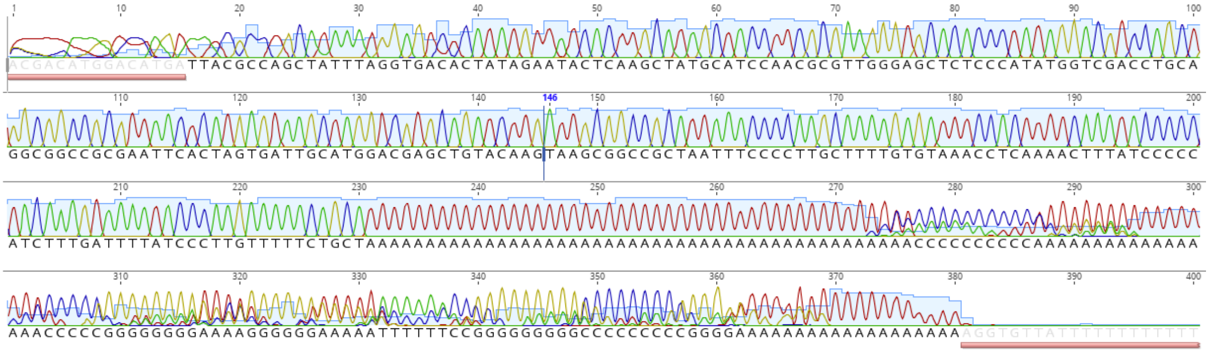

1EA6SAA000_G02_premix.ab1

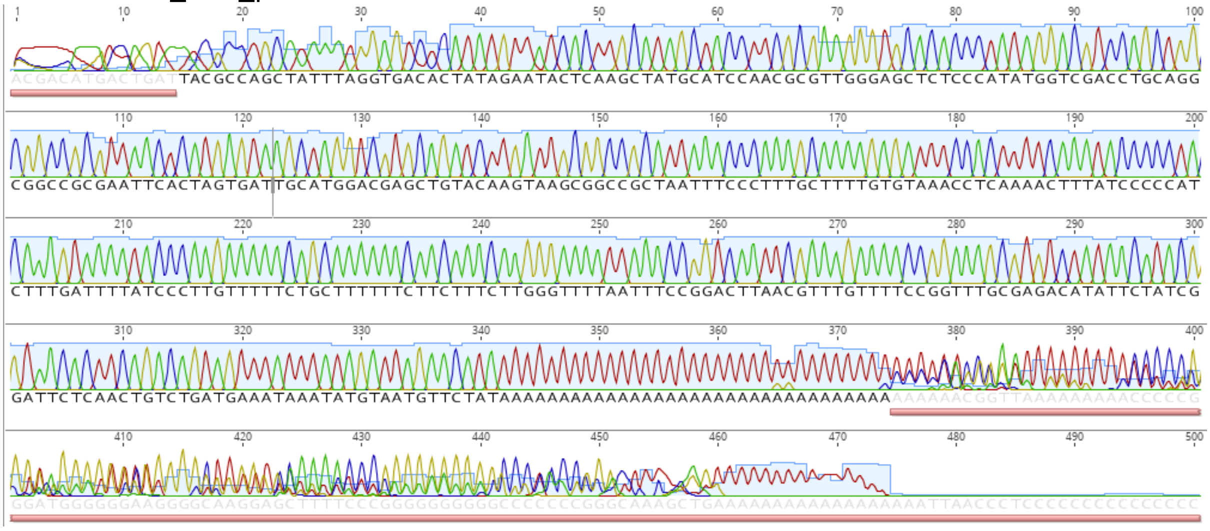

1EA6HAB000_B05_premix.ab1

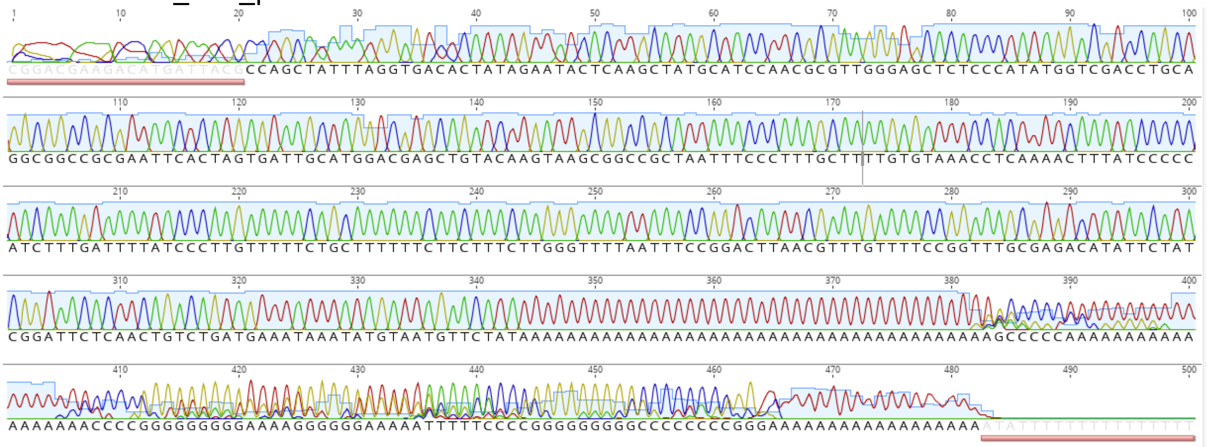

1EA6HAB000_C05_premix.ab1

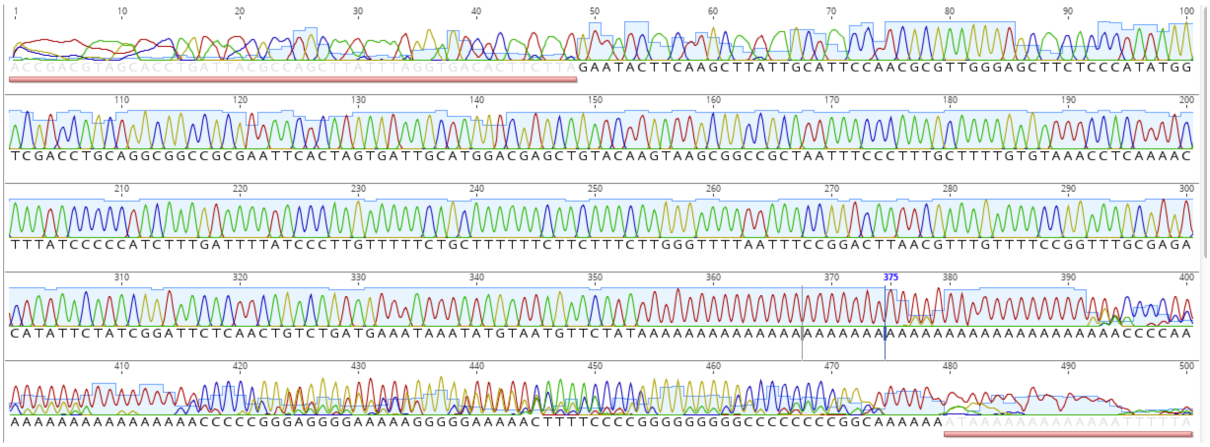

1EA6HAB000_D05_premix.ab1

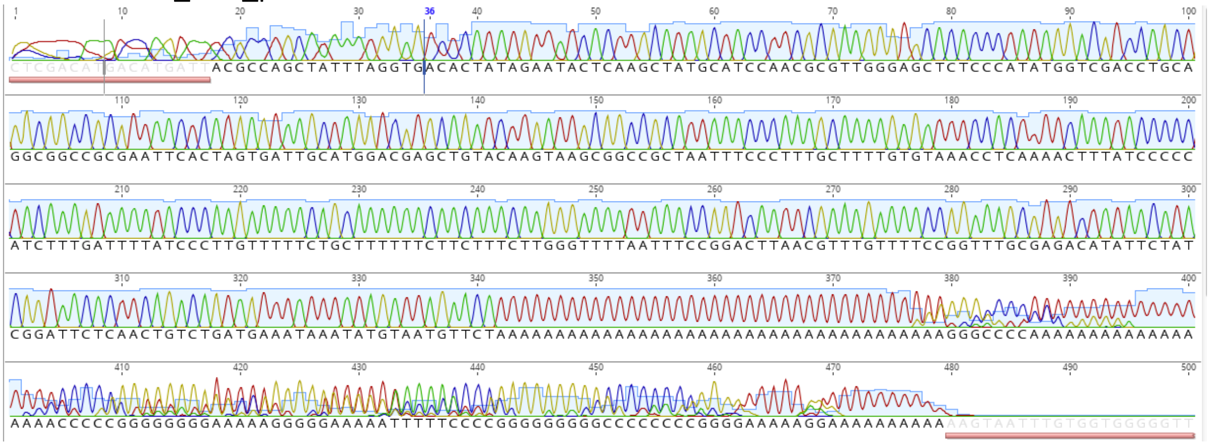

1EA6HAB000_E05_premix.ab1

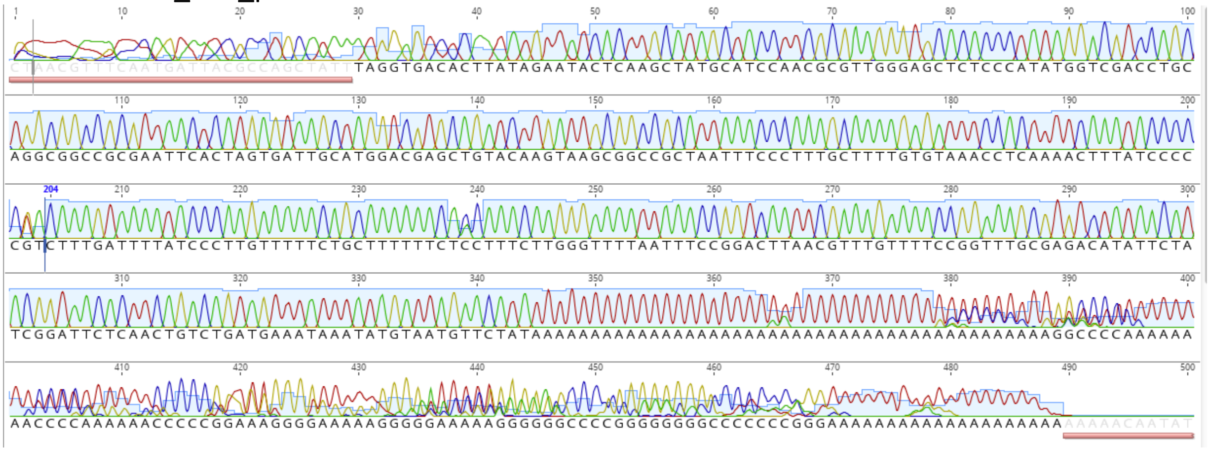

1EA6HAB000_F05_premix.ab1

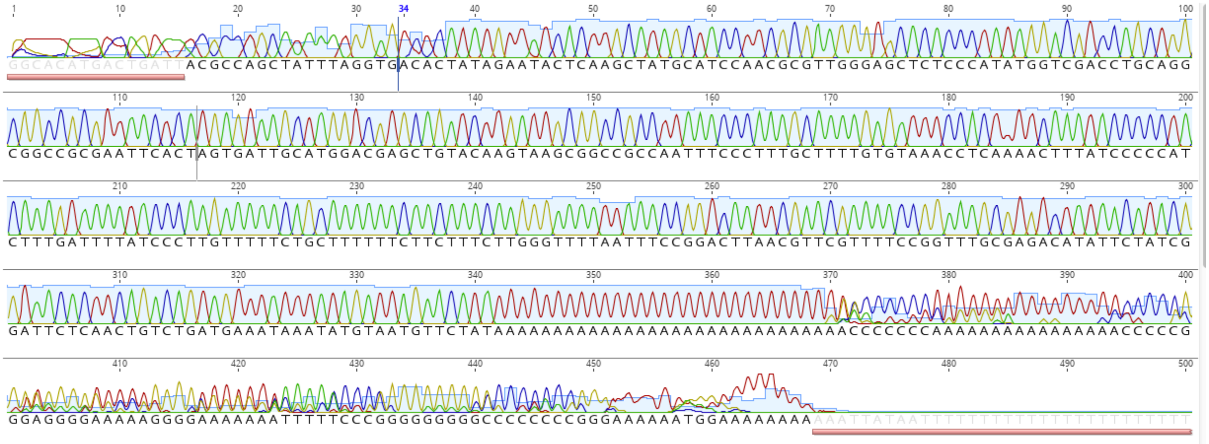

1EA6HAB000_G05_premix.ab1

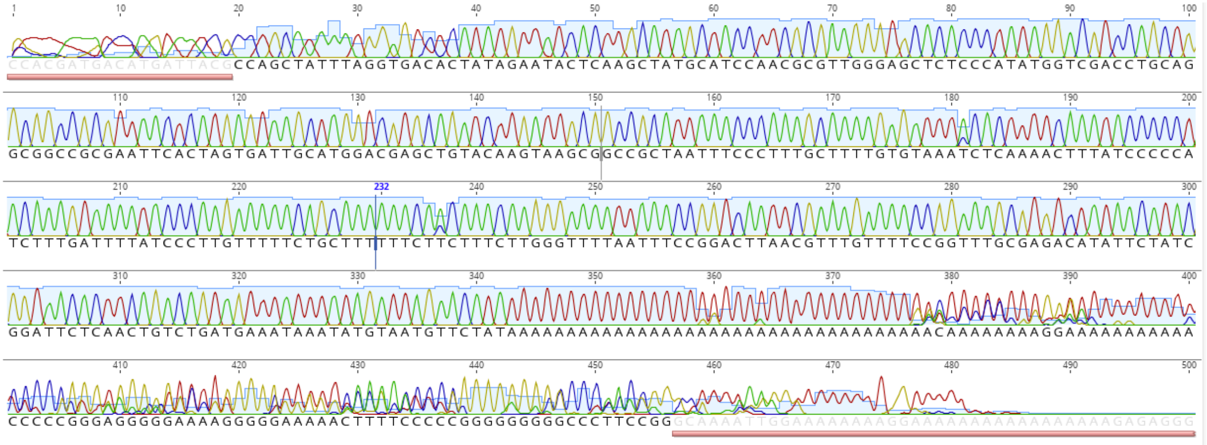

1EA6HAB000_H05_premix.ab1

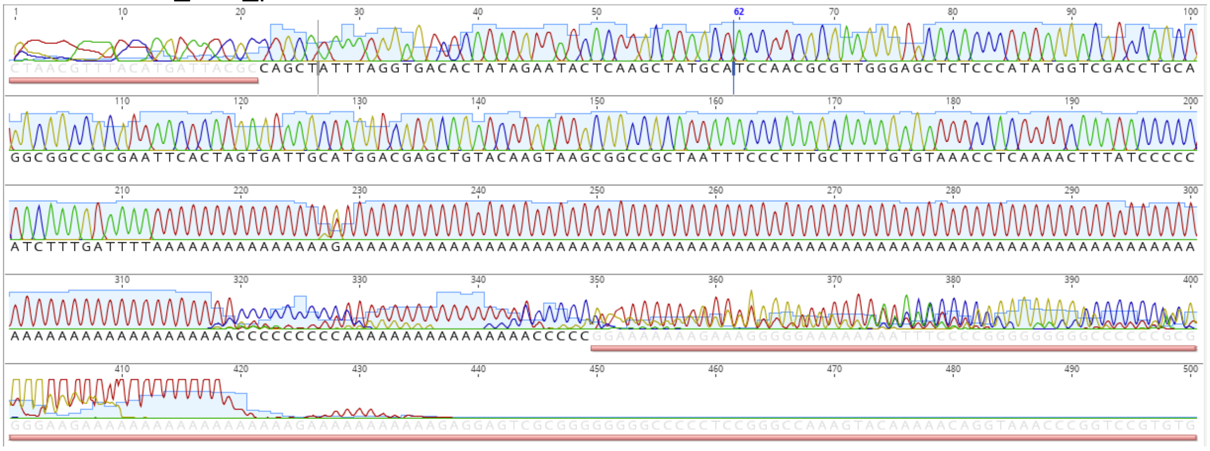

1EA6HAB000_A06_premix.ab1

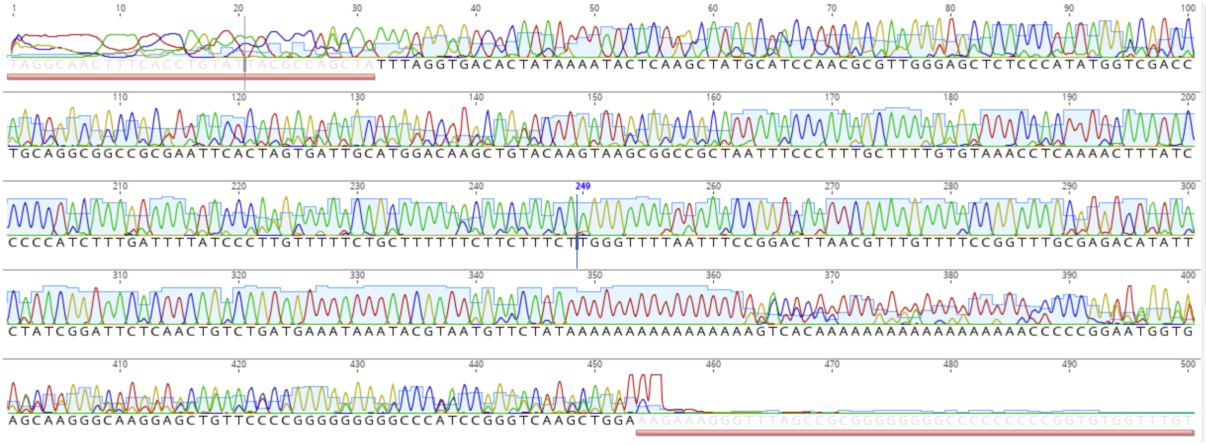

1EA6HAB000_B06_premix.ab1

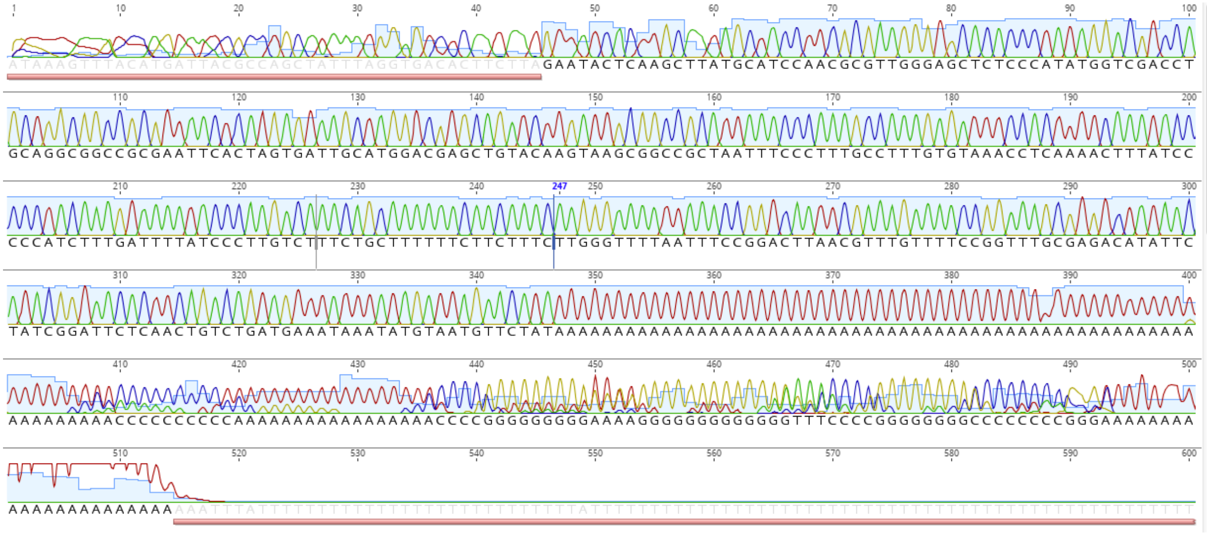

1EA6HAB000_C06_premix.ab1

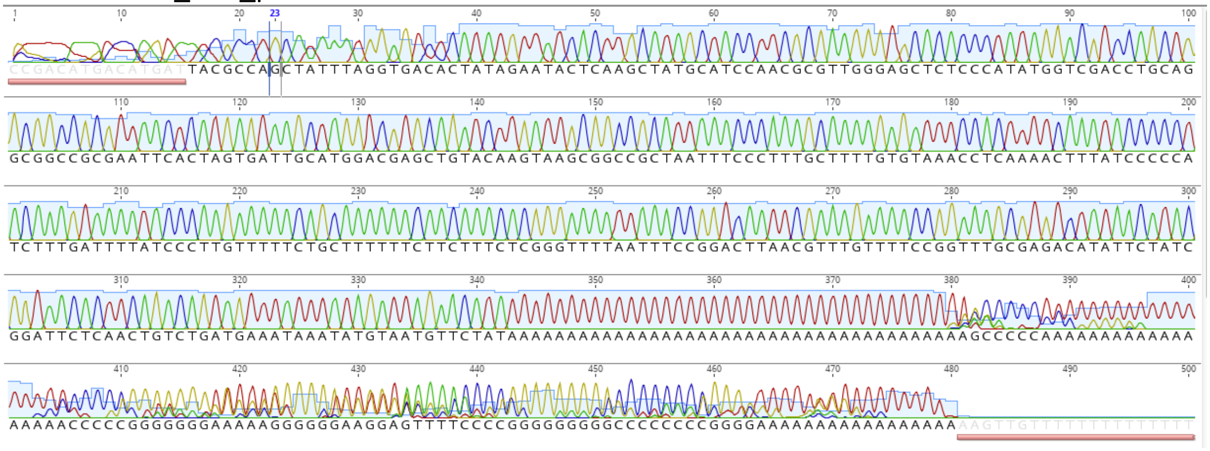

1EA6HAB000_D06_premix.ab1

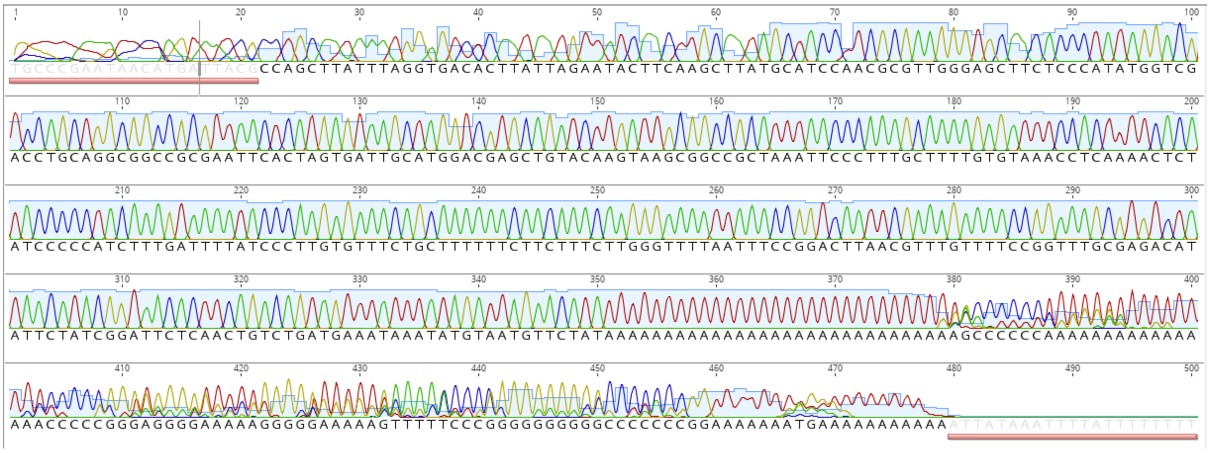

1EA6HAB000_E06_premix.ab1

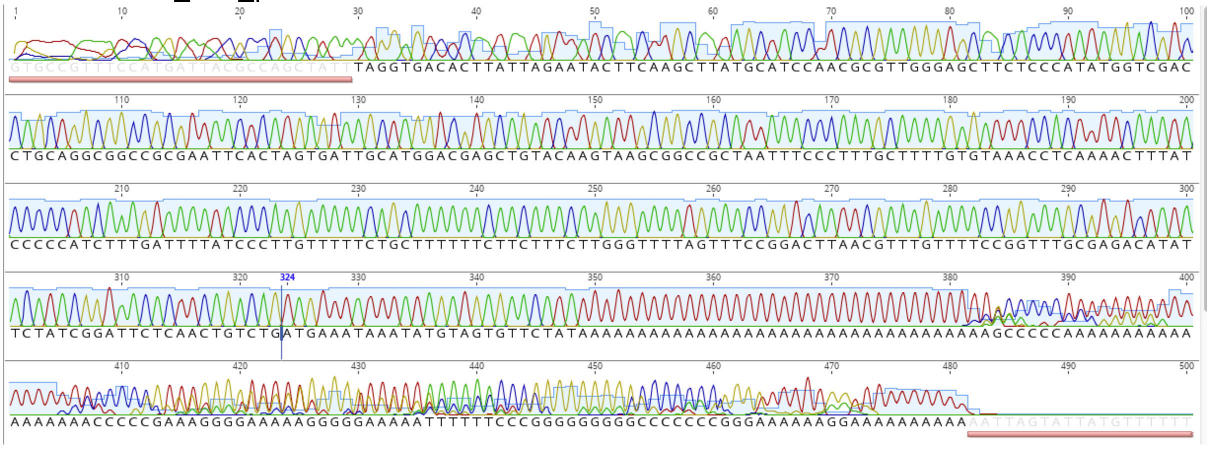

tRBCS_tHSP_5'

1EA6HAB000_H02_premix.ab1

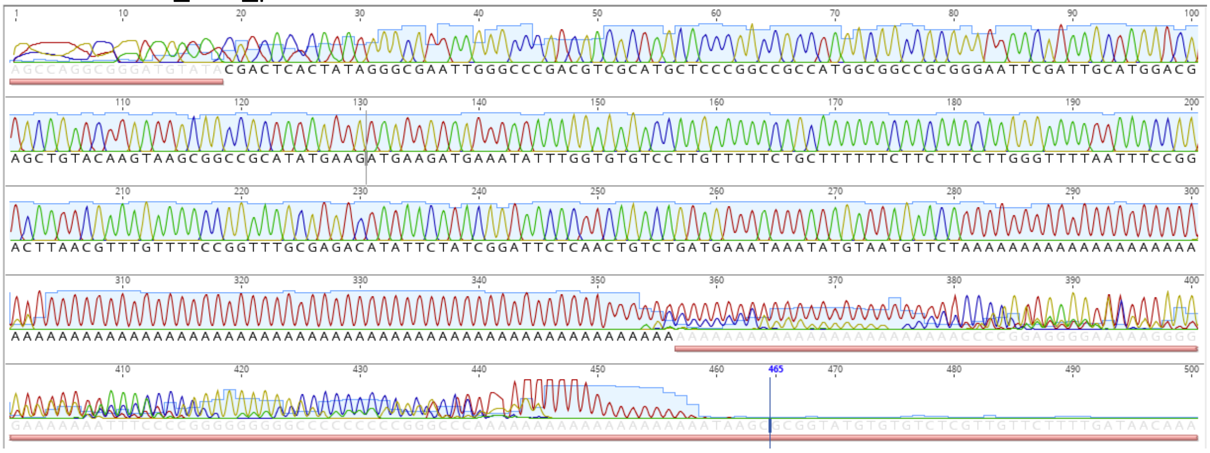

1EA6HAB000_A03_premix.ab1

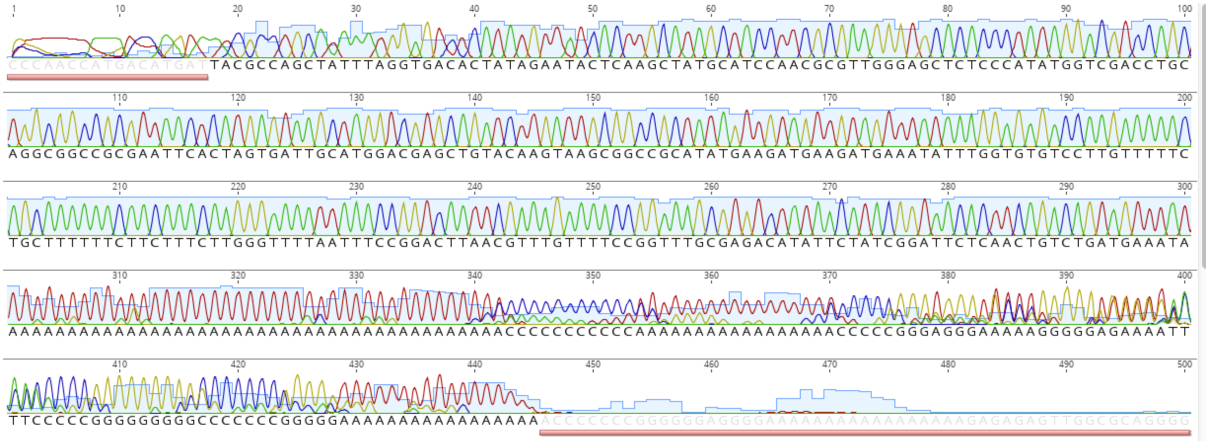

1EA6HAB000_B03_premix.ab1

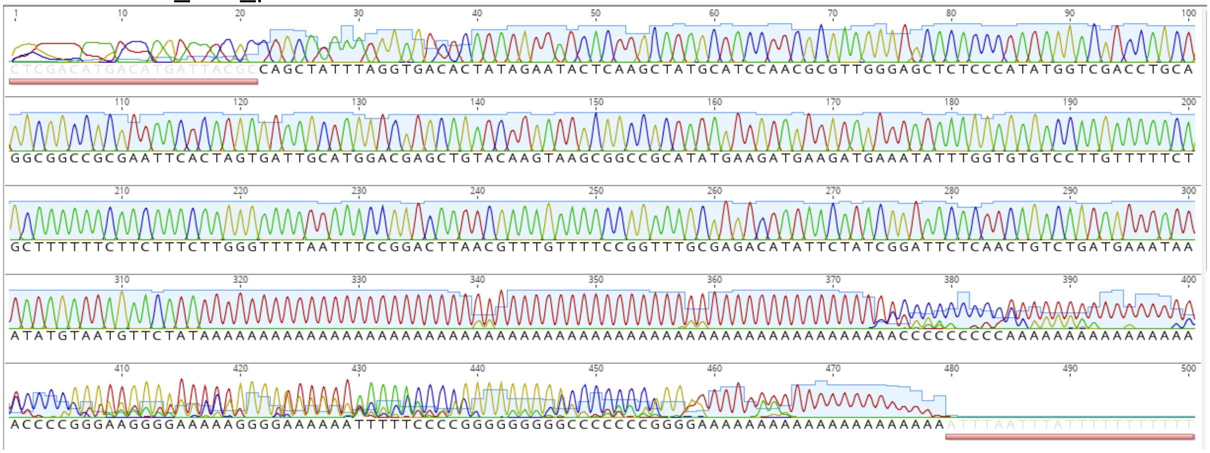

1EA6HAB000_C03_premix.ab1

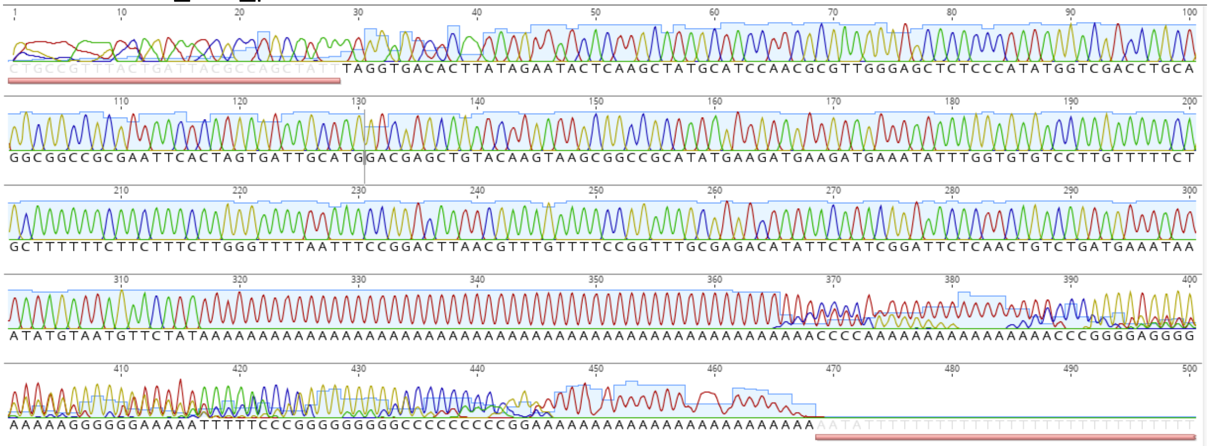

1EA6HAB000_D03_premix.ab1

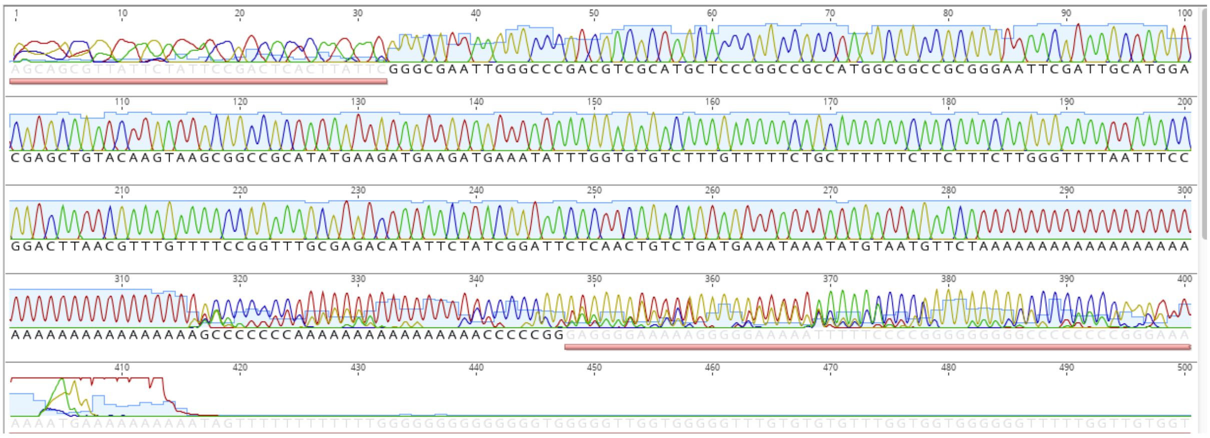

1EA6HAB000_E03_premix.ab1

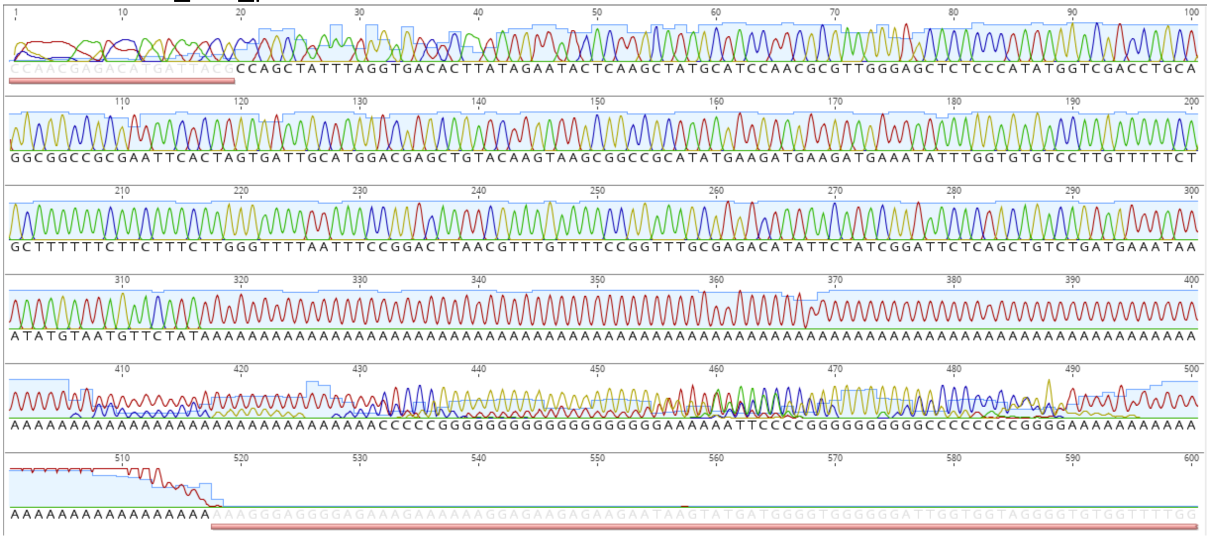

1EA6HAB000_F03_premix.ab1

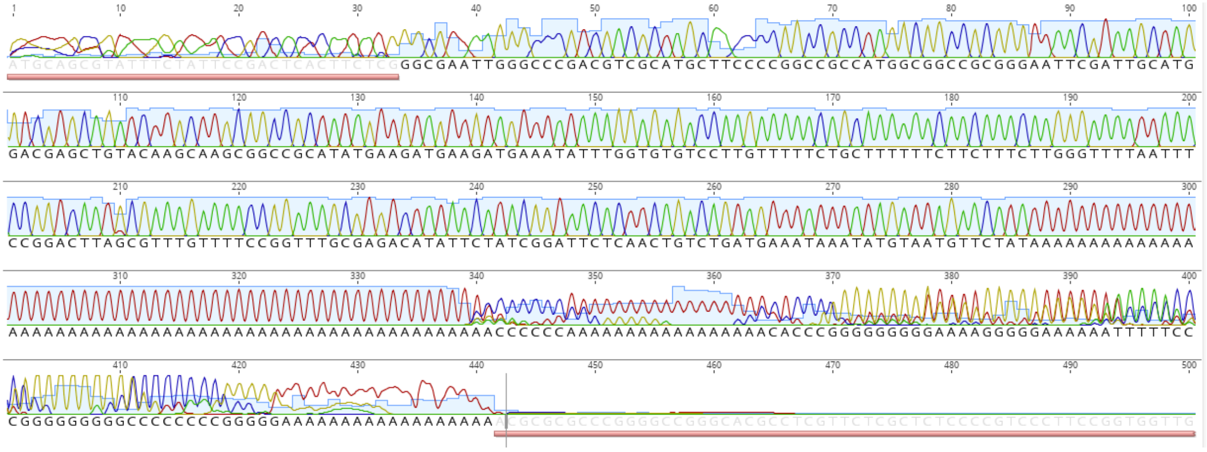

1EA6HAB000_G03_premix.ab1

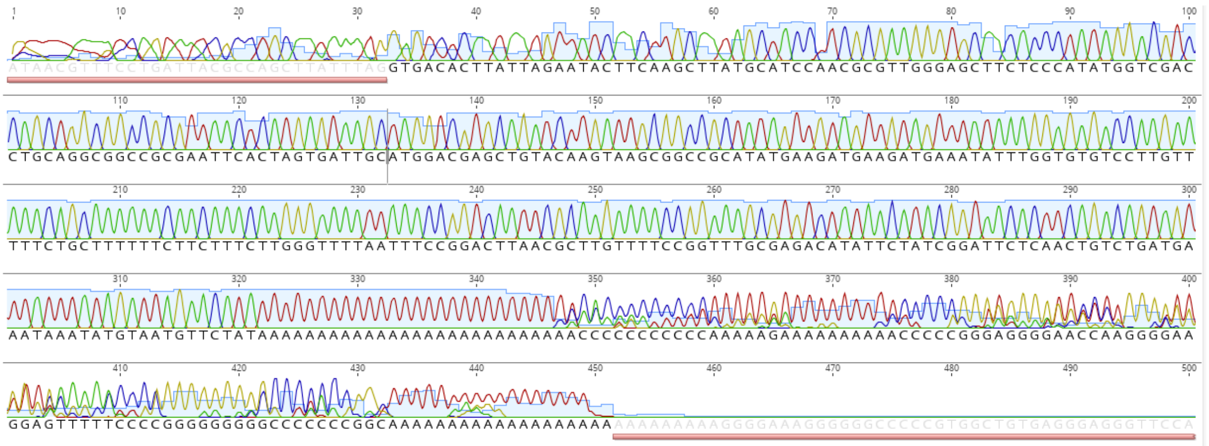

1EA6SAA000_D10_premix.ab1

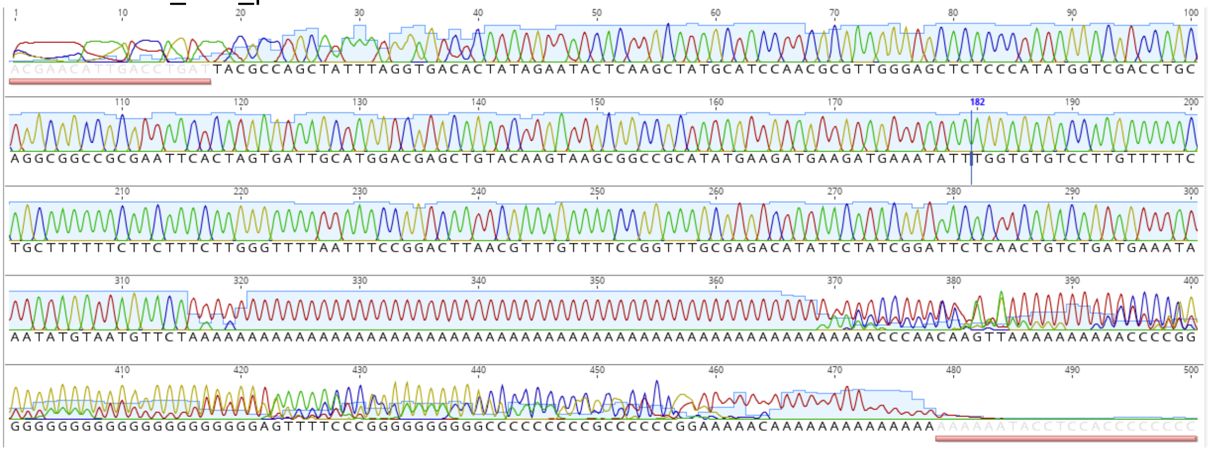

1EA6SAA000_E10_premix.ab1

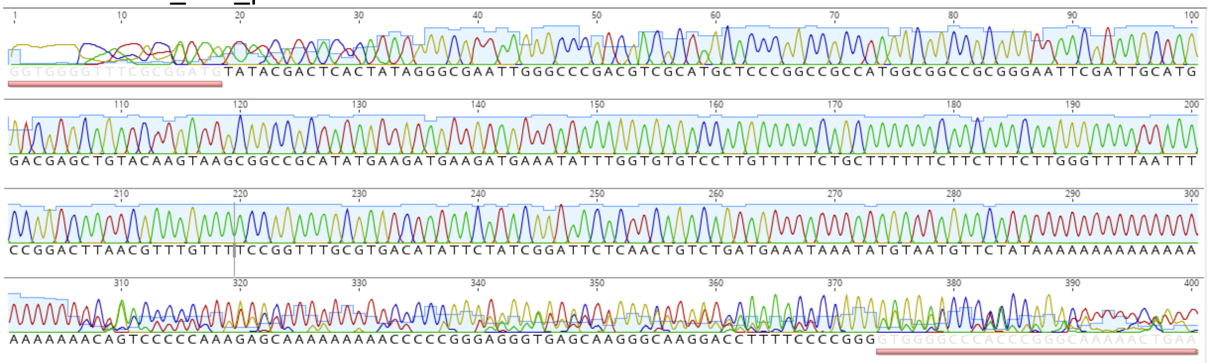

1EA6SAA000_F10_premix.ab1

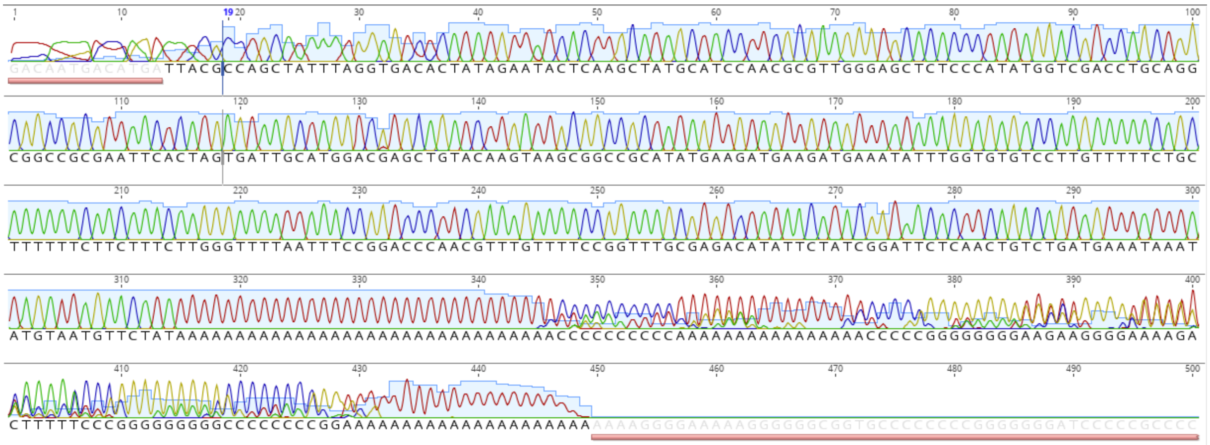

1EA6SAA000_G10_premix.ab1

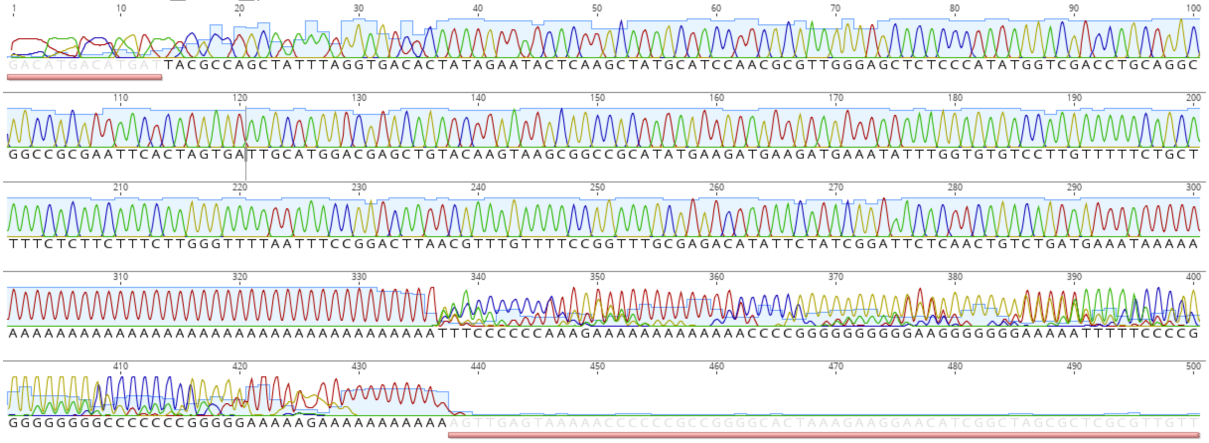

1EA6SAA000_H10_premix.ab1

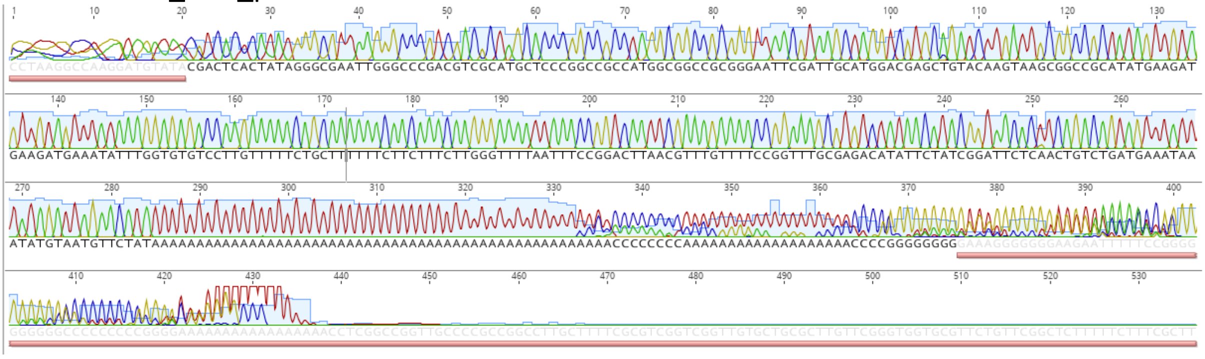

1EA6SAA000_A11_premix.ab1

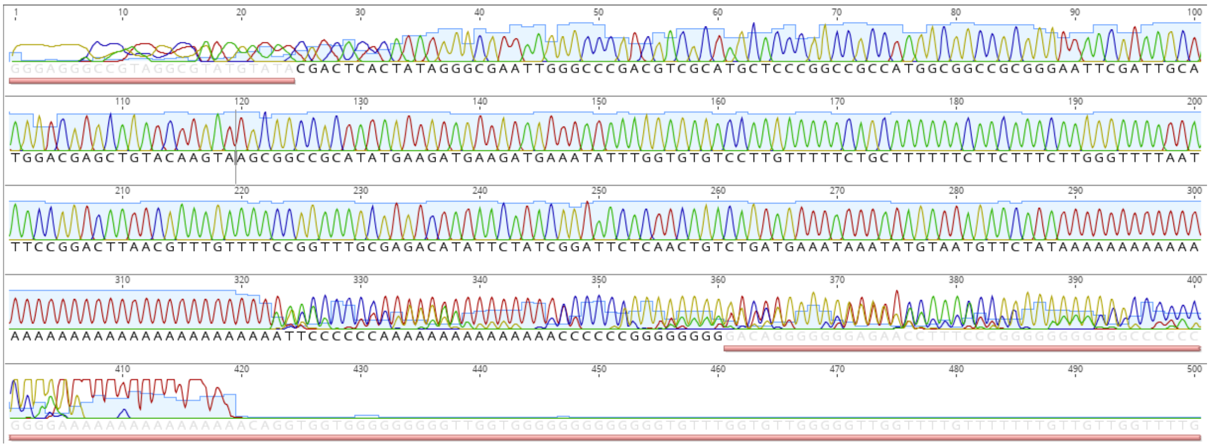

1EA6SAA000_B11_premix.ab1

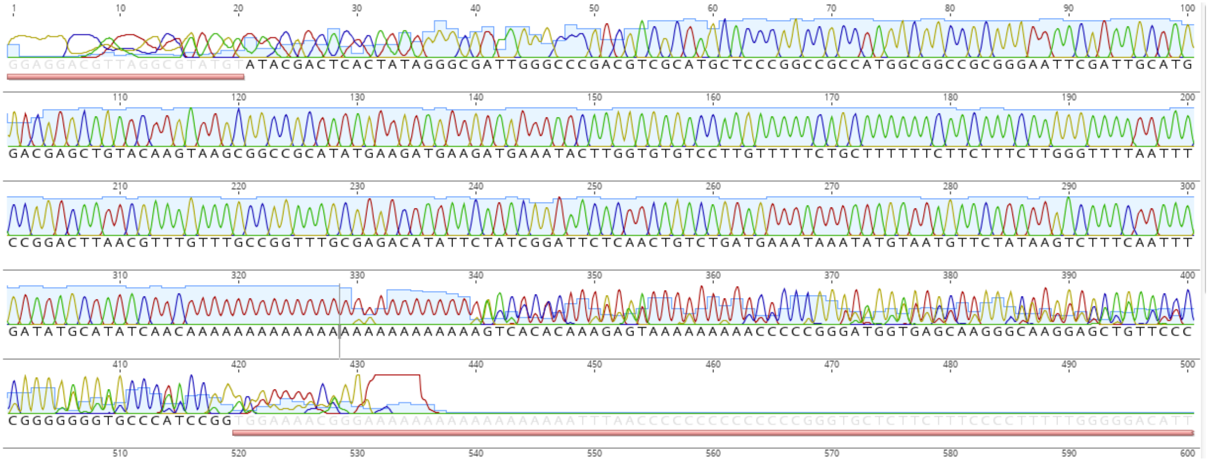

1EA6SAA000_C11_premix.ab1

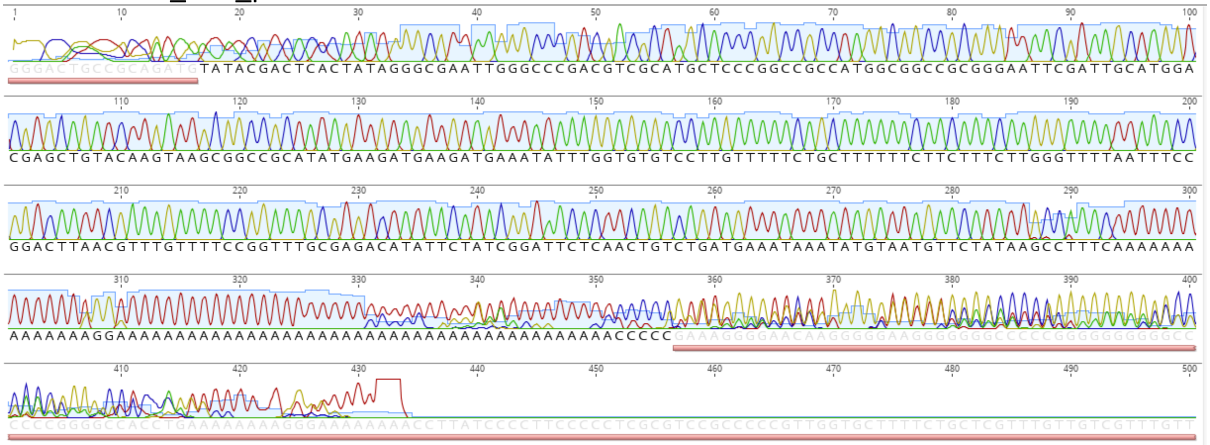

1EA6SAA000_D11_premix.ab1

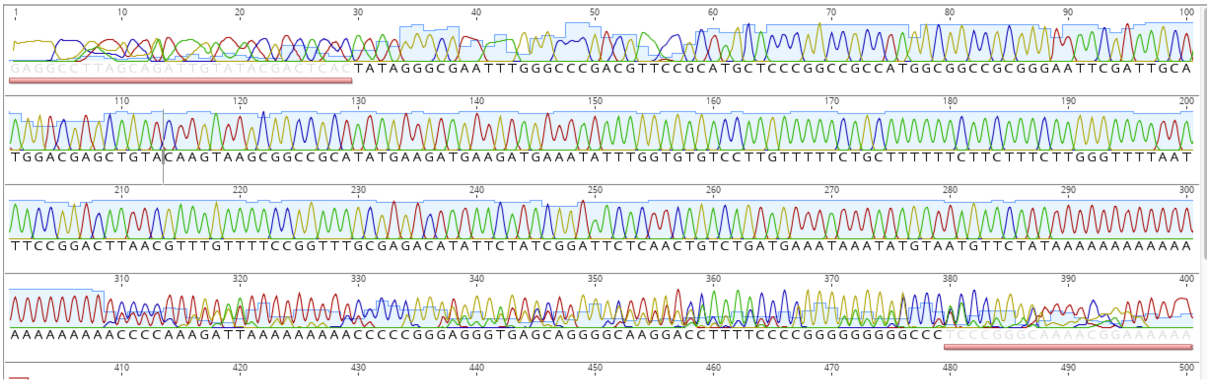

1EA6SAA000_E11_premix.ab1

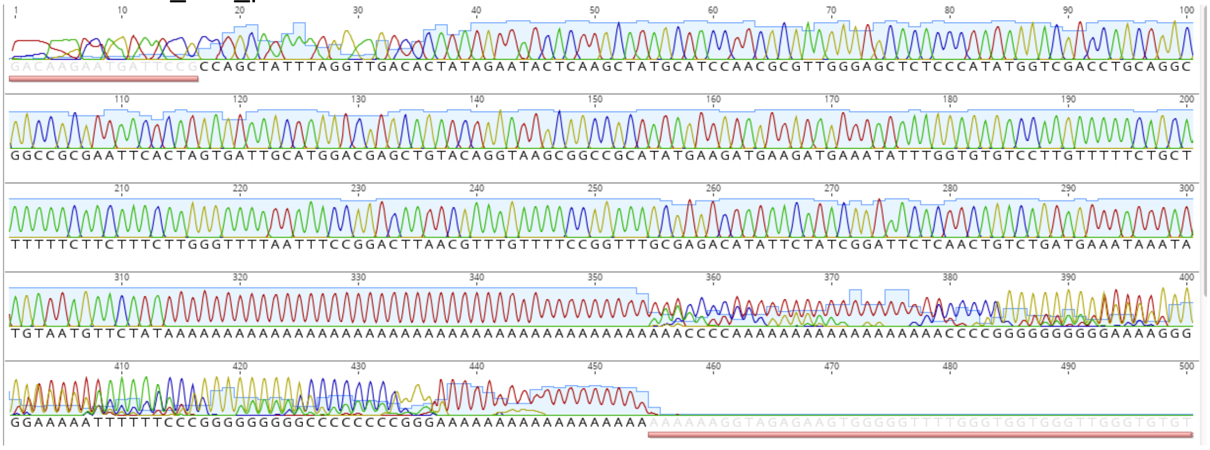

1EA6SAA000_F11_premix.ab1

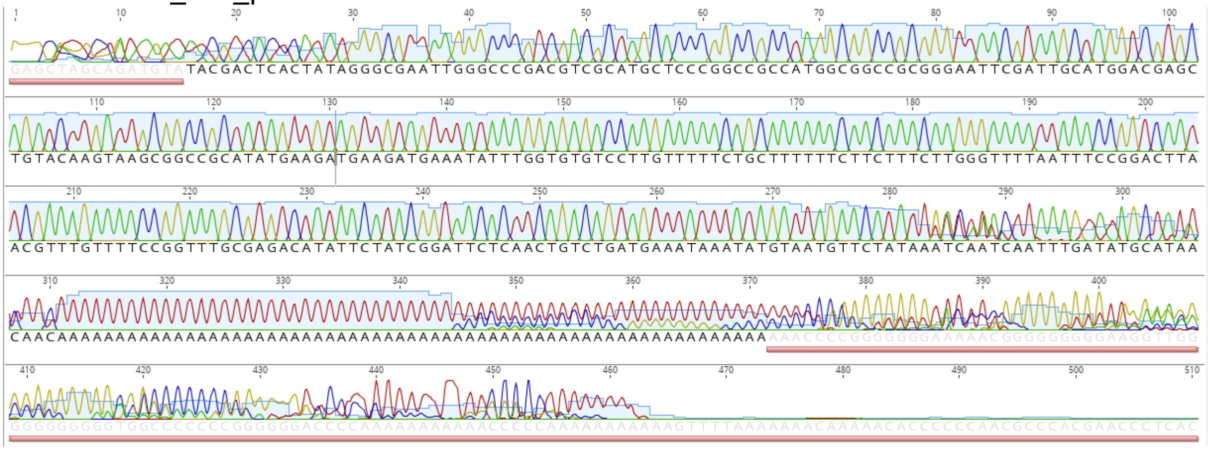

1EA6SAA000_G11_premix.ab1

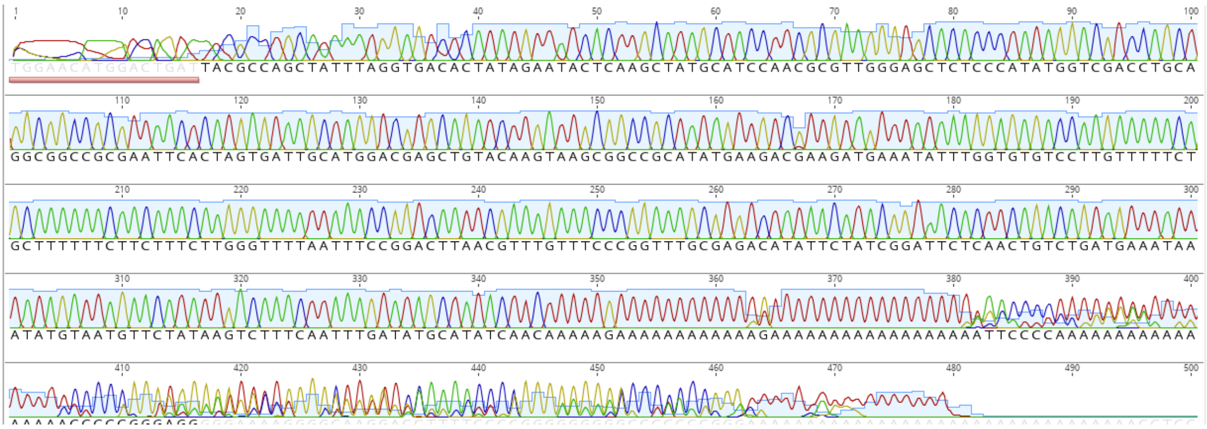

1EA6SAA000_H11_premix.ab1

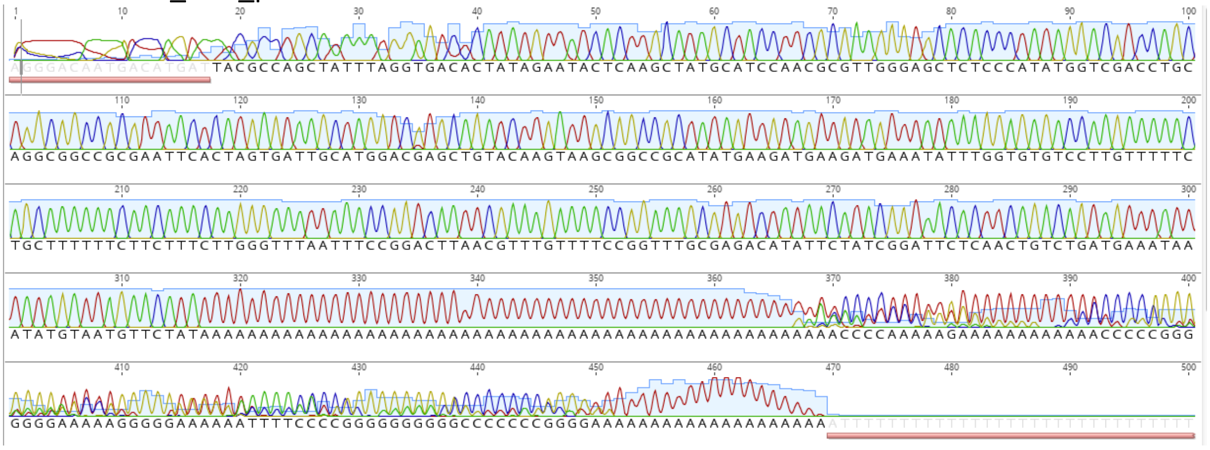

1EA6SAA000_A12_premix.ab1

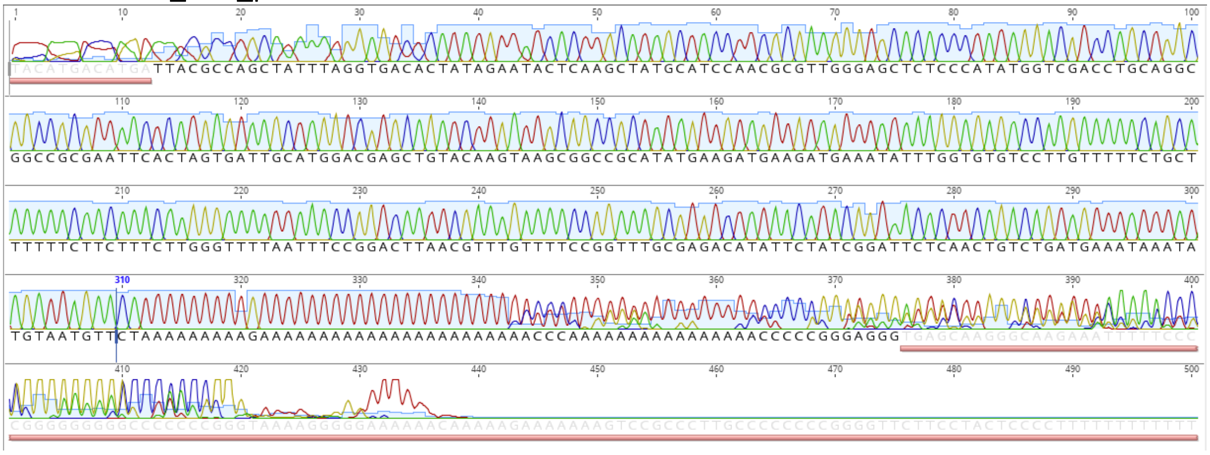

1EA6SAA000_B12_premix.ab1

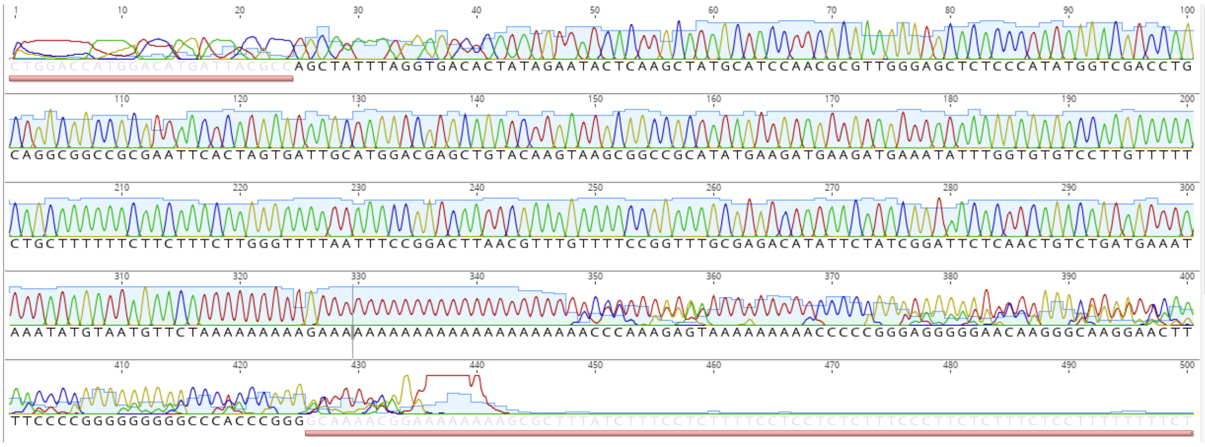

tNOS

1EA6HAB000_A02_premix.ab1

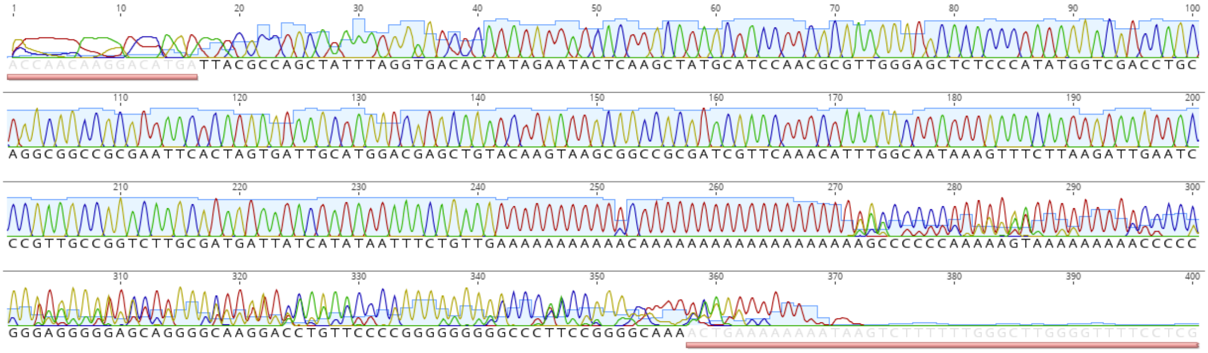

1EA6HAB000_B02_premix.ab1

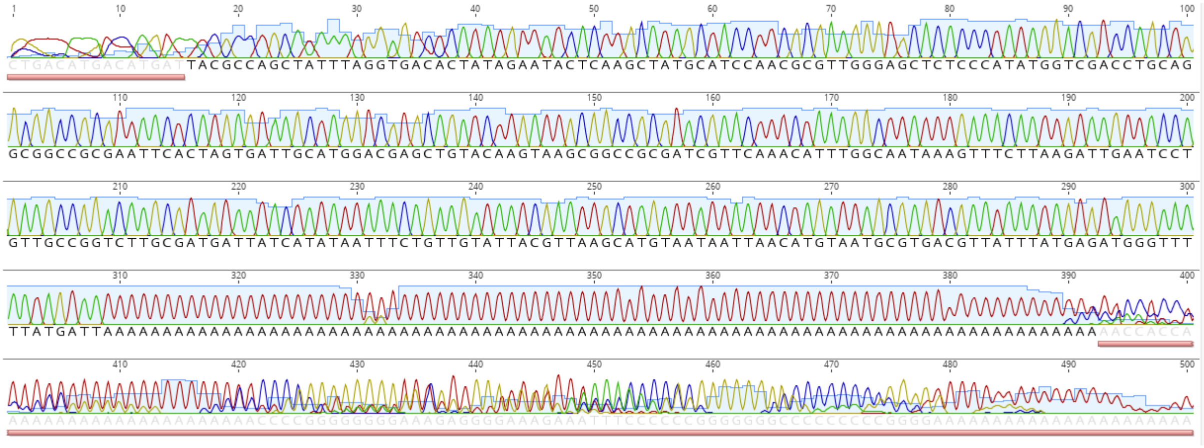

1EA6HAB000_C02_premix.ab1

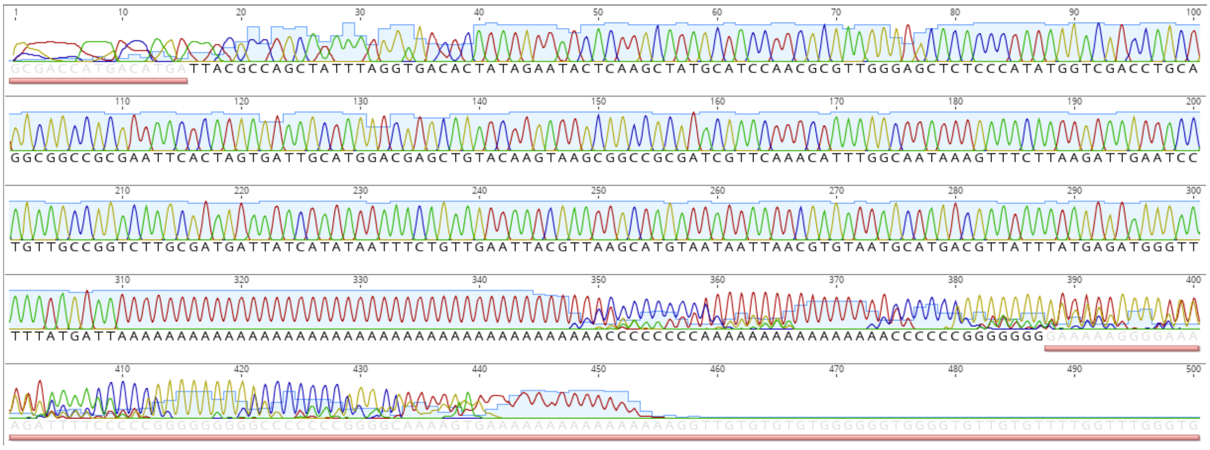

1EA6HAB000_D02_premix.ab1

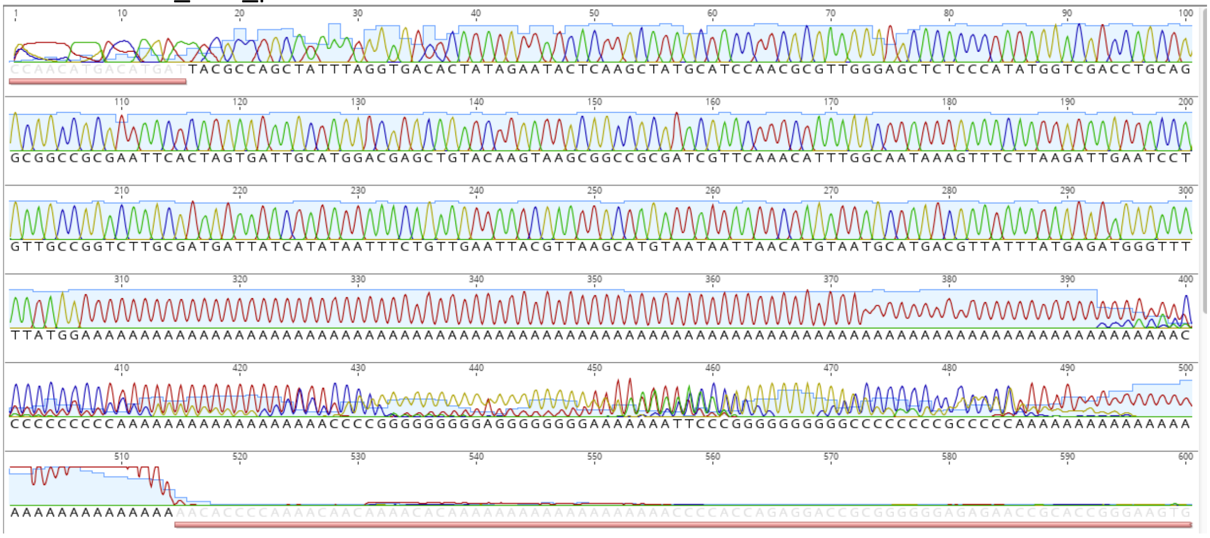

1EA6HAB000_E02_premix.ab1

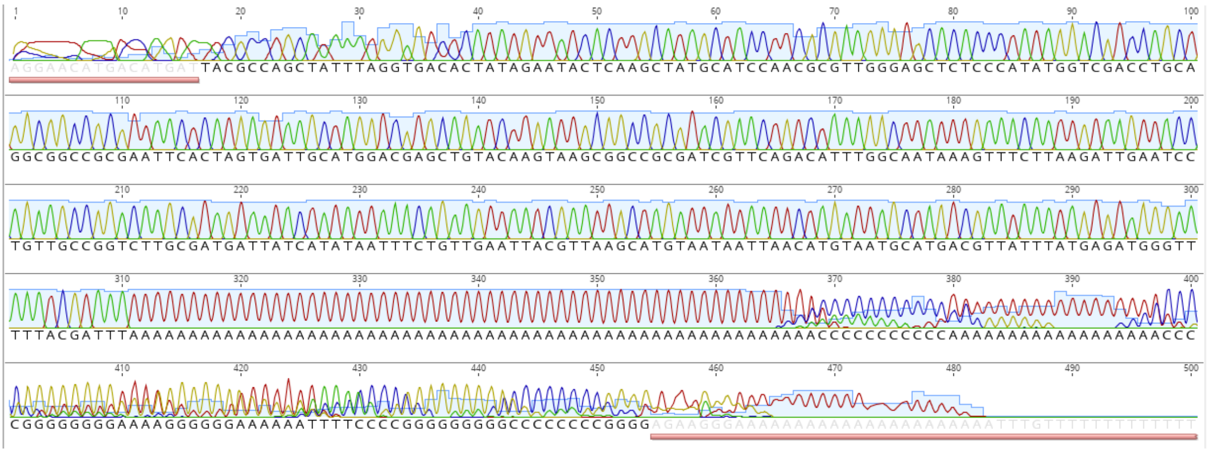

1EA6HAB000_F02_premix.ab1

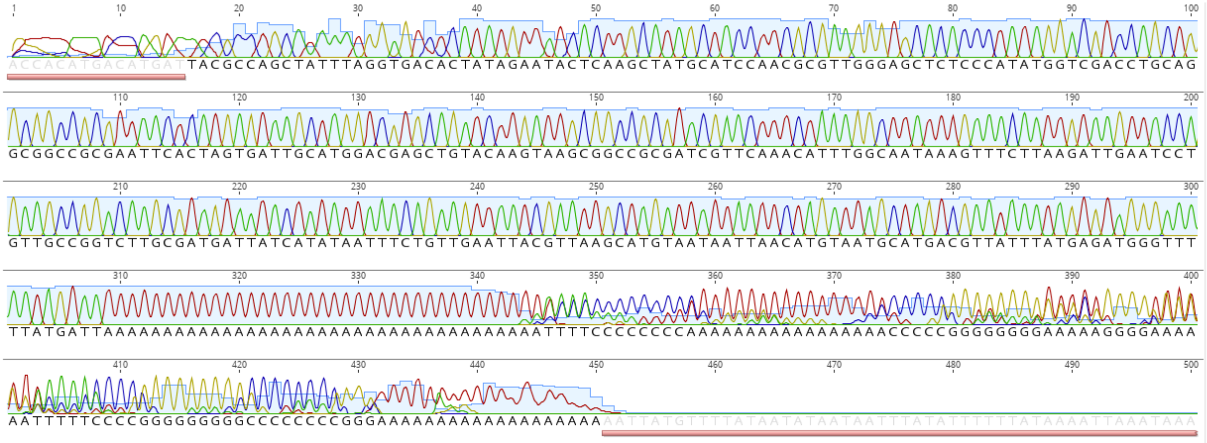

1EA6HAB000_G02_premix.ab1

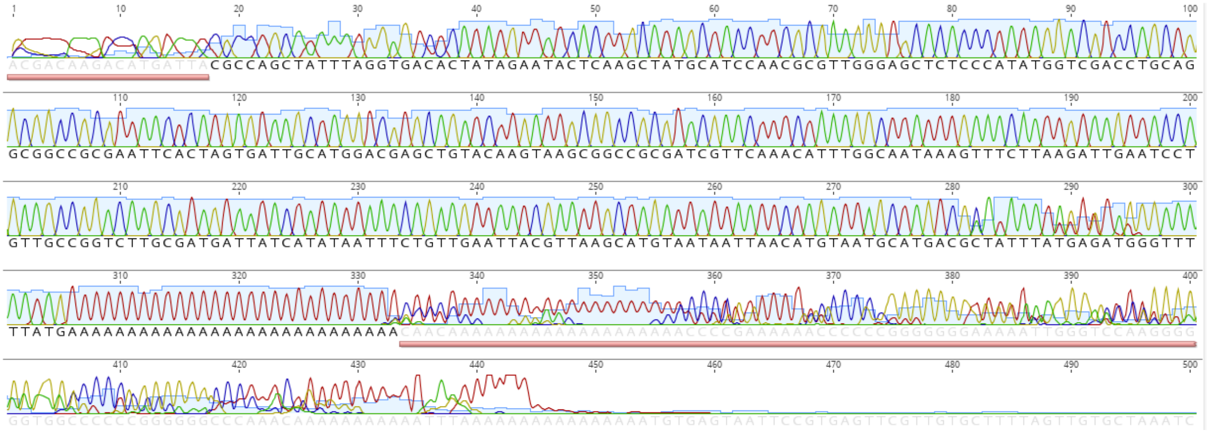

1EA6SAA000_H02_premix.ab1

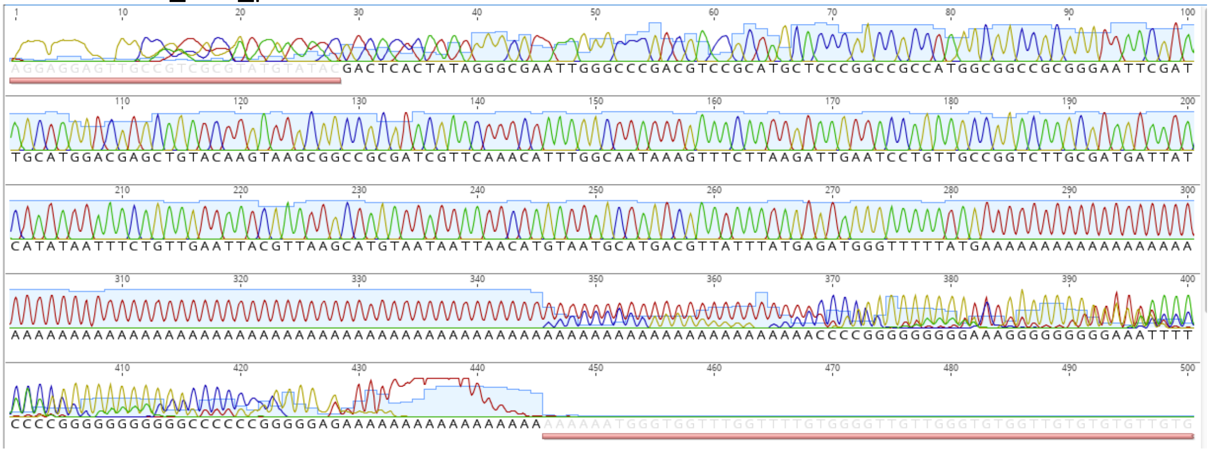

1EA6SAA000_A03_premix.ab1

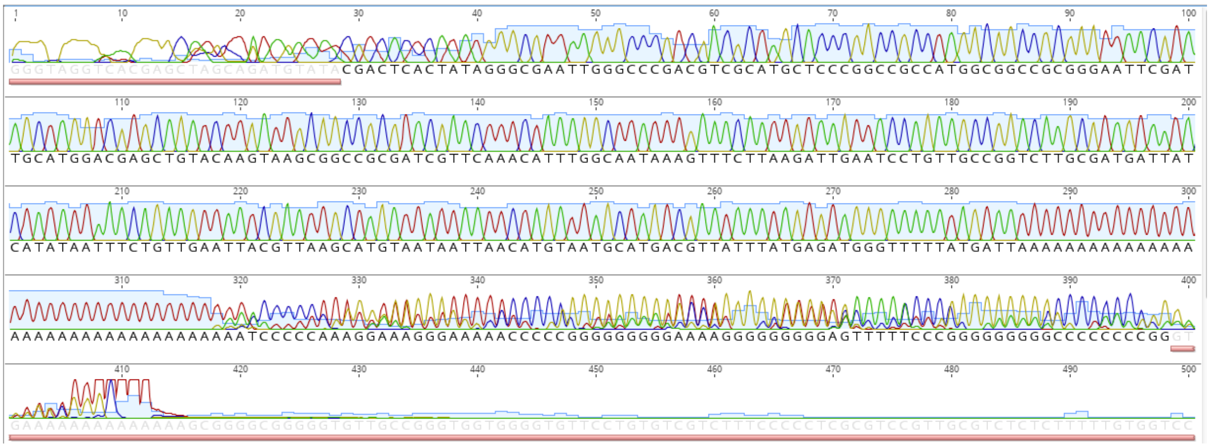

1EA6SAA000_B03_premix.ab1

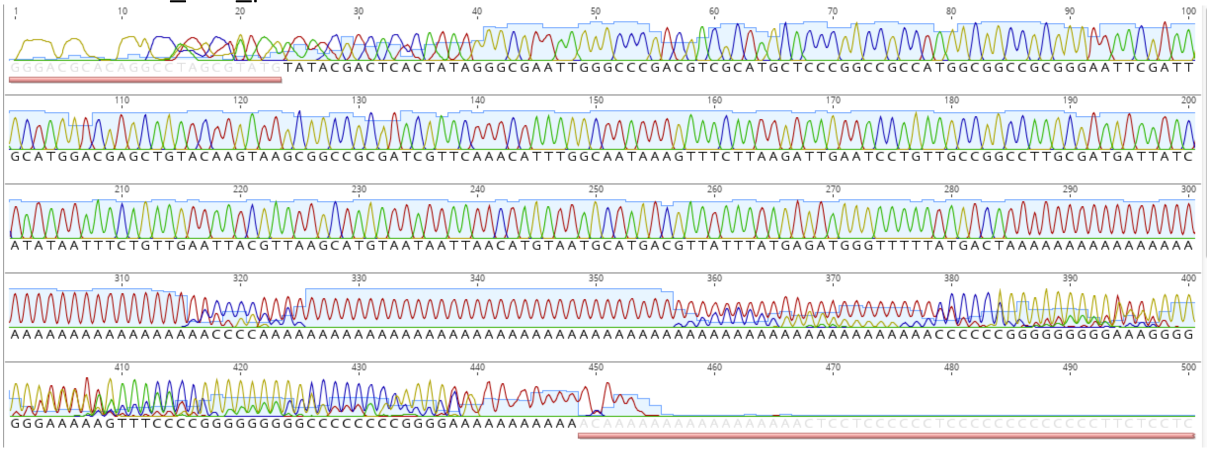

1EA6SAA000_C03_premix.ab1

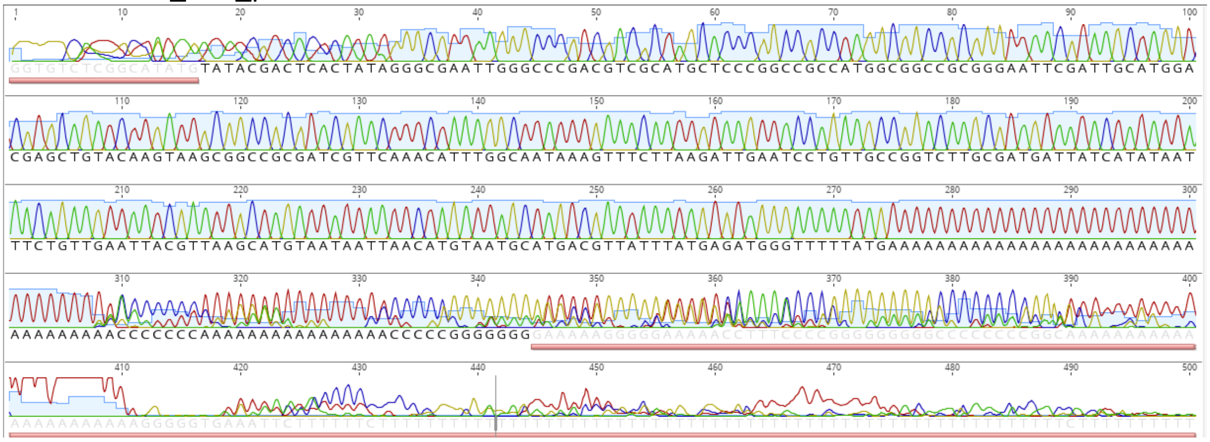

1EA6SAA000_D03_premix.ab1

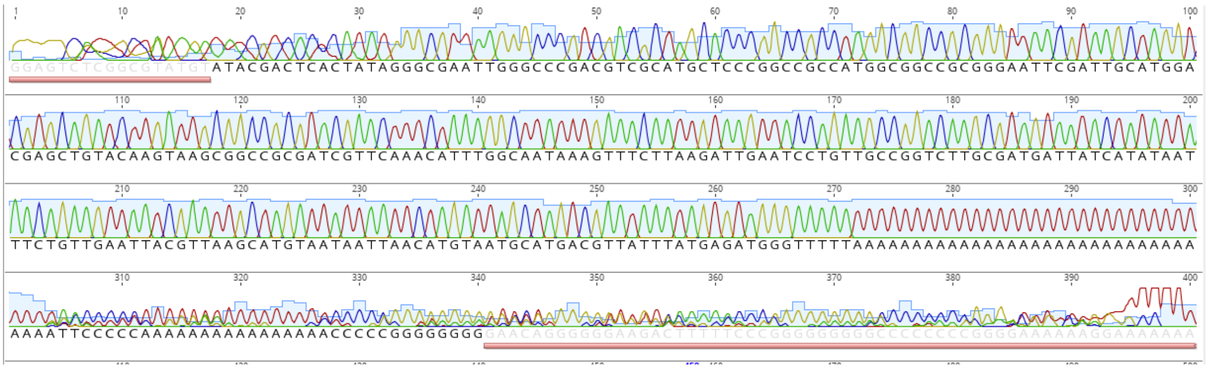

1EA6SAA000_E03_premix.ab1

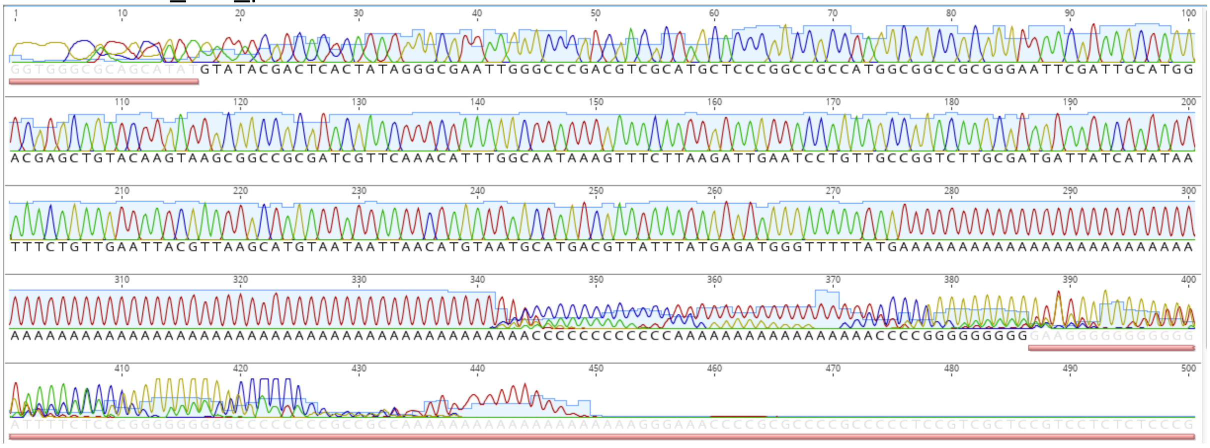

1EA6SAA000_F03_premix.ab1

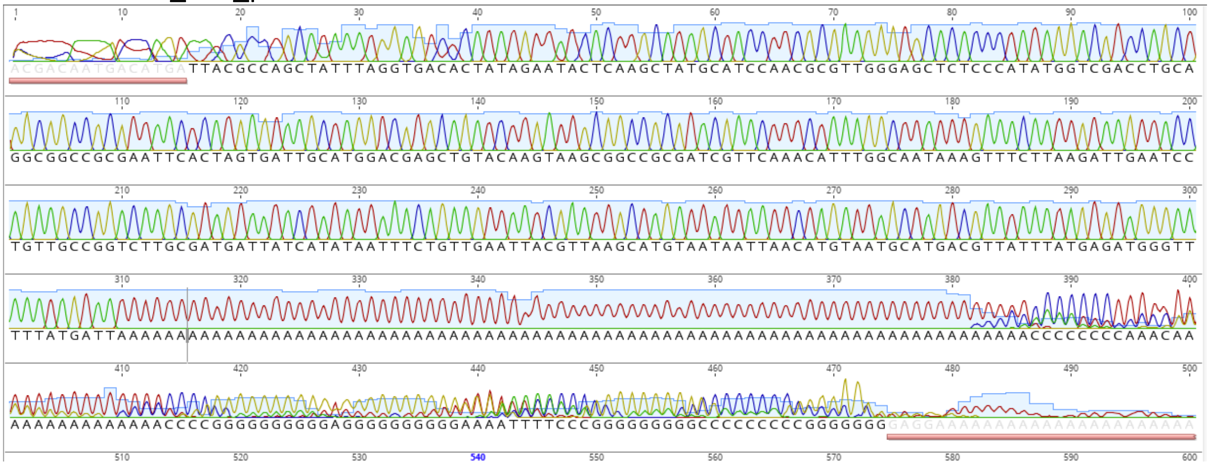

1EA6SAA000_G03_premix.ab1

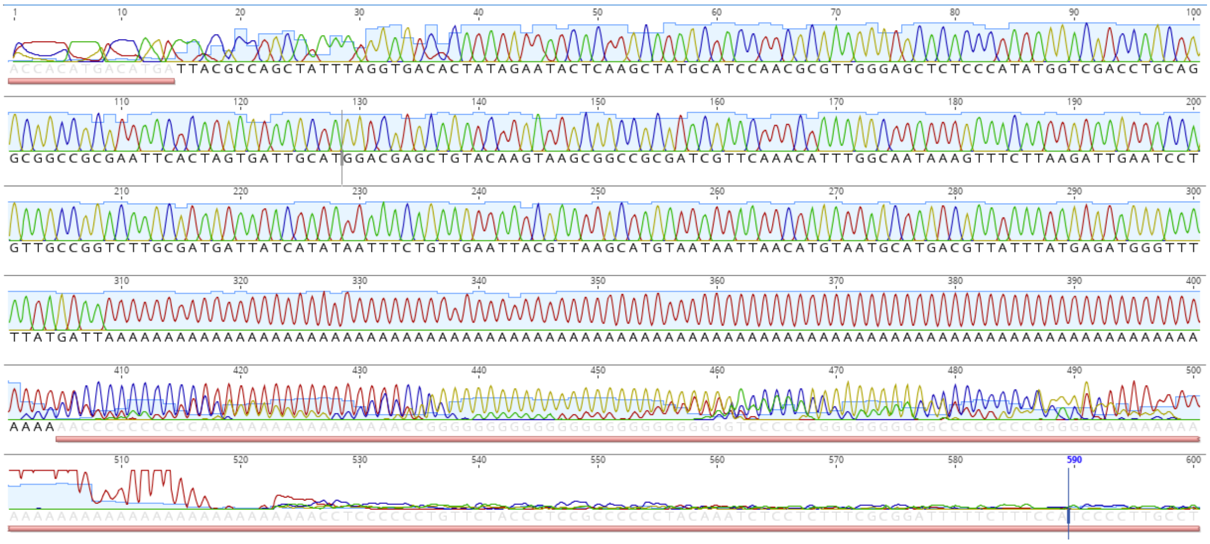

1EA6SAA000_H03_premix.ab1

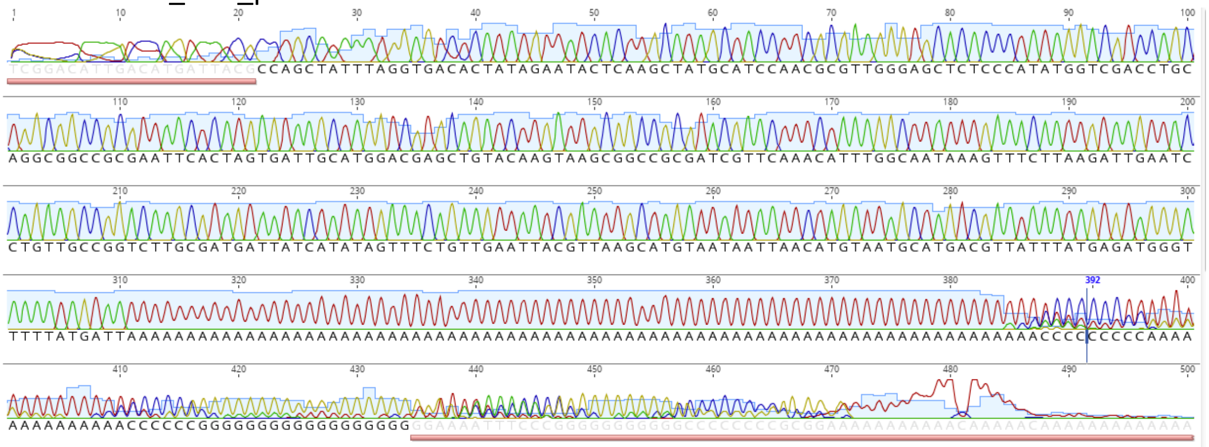

1EA6SAA000_A04_premix.ab1

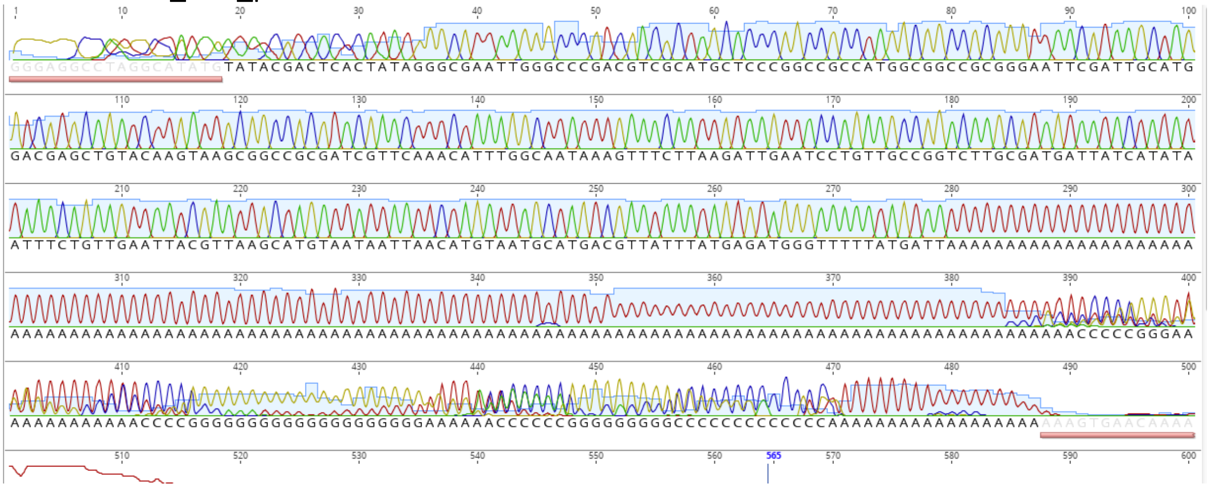

1EA6SAA000_B04_premix.ab1

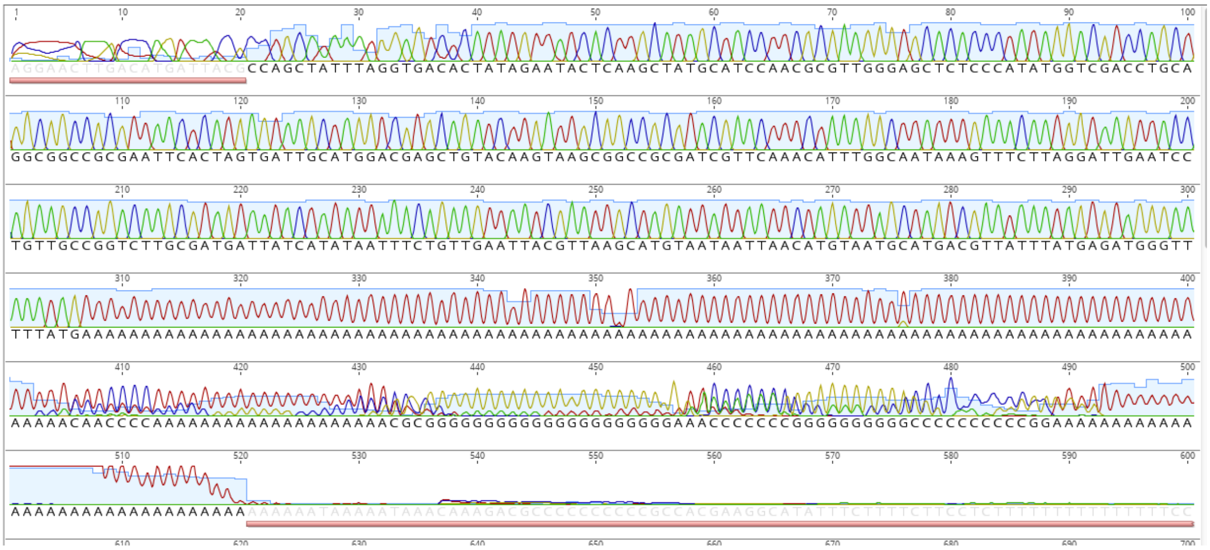

1EA6SAA000_D04_premix.ab1

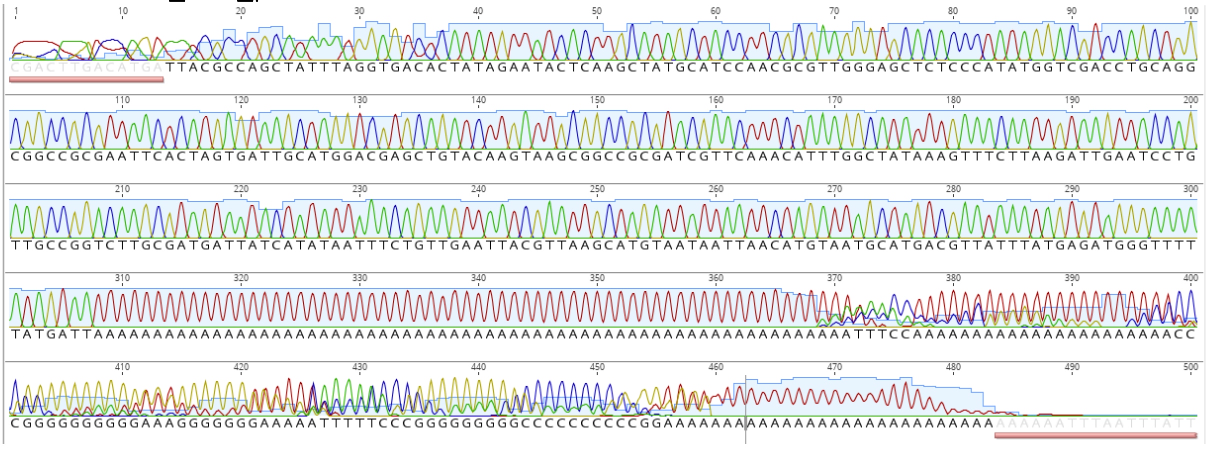

1EA6SAA000_E04_premix.ab1

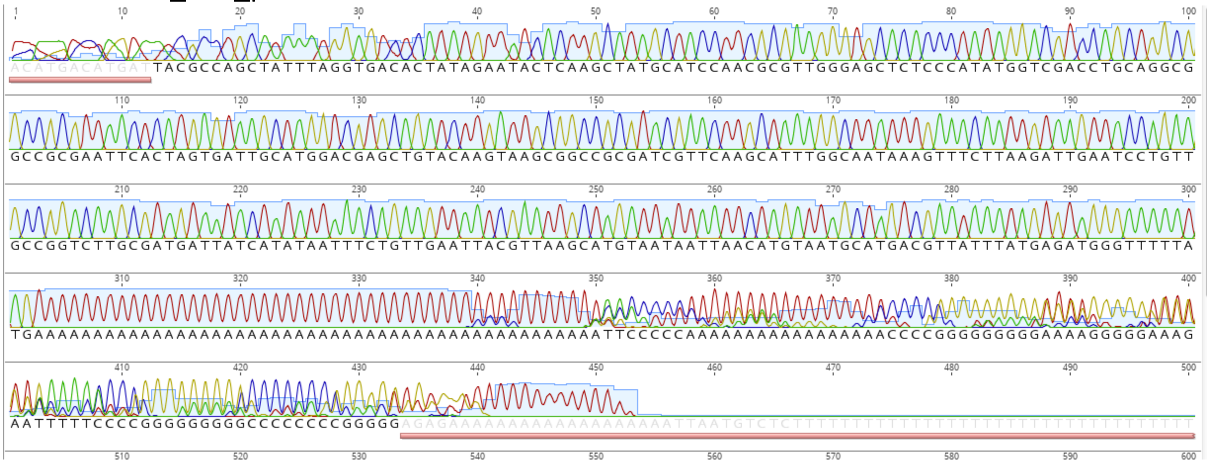

1EA6SAA000_F04_premix.ab1

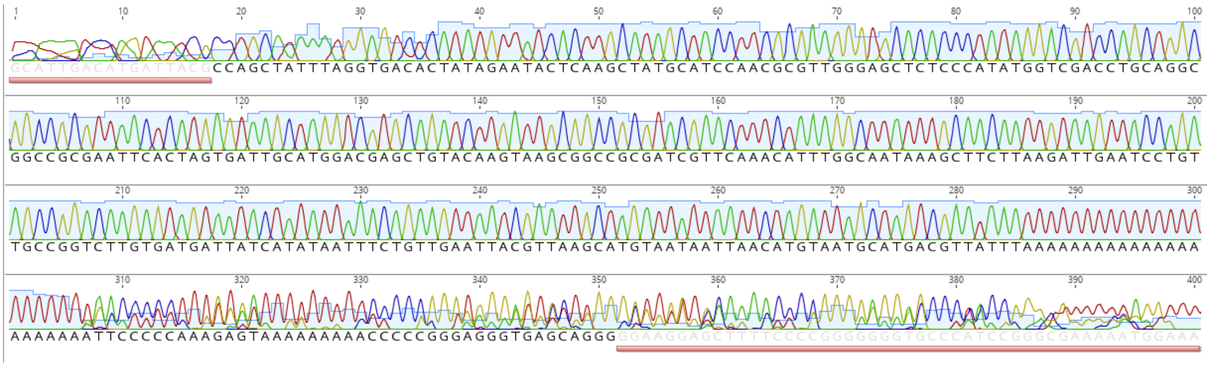

tNOS_tHSP_5'

1EA6SAA000_E08_premix.ab1

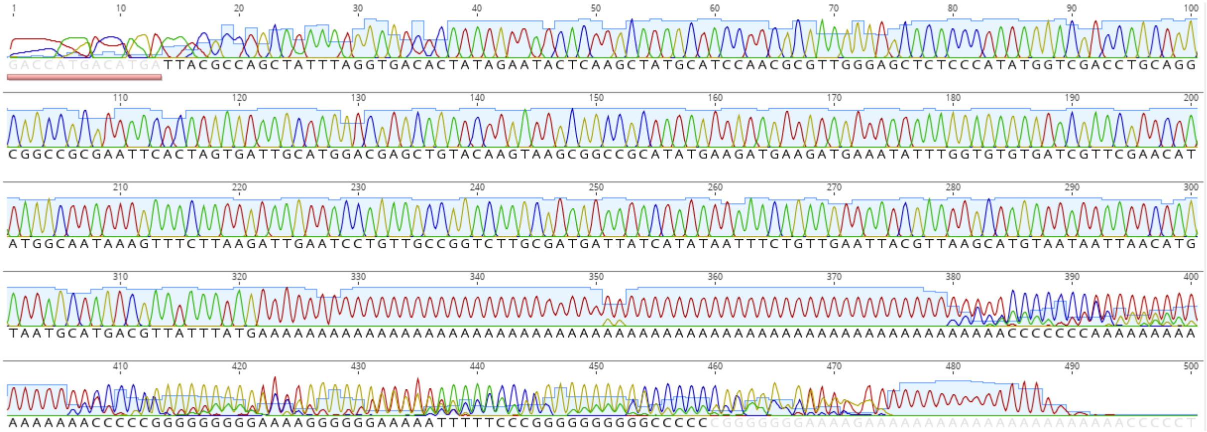

1EA6SAA000_F08_premix.ab1

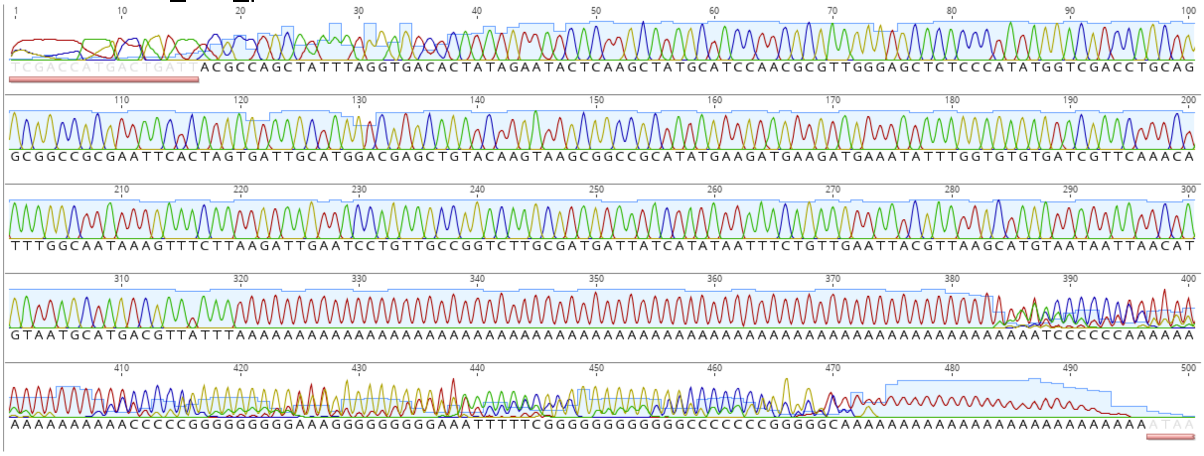

1EA6SAA000_G08_premix.ab1

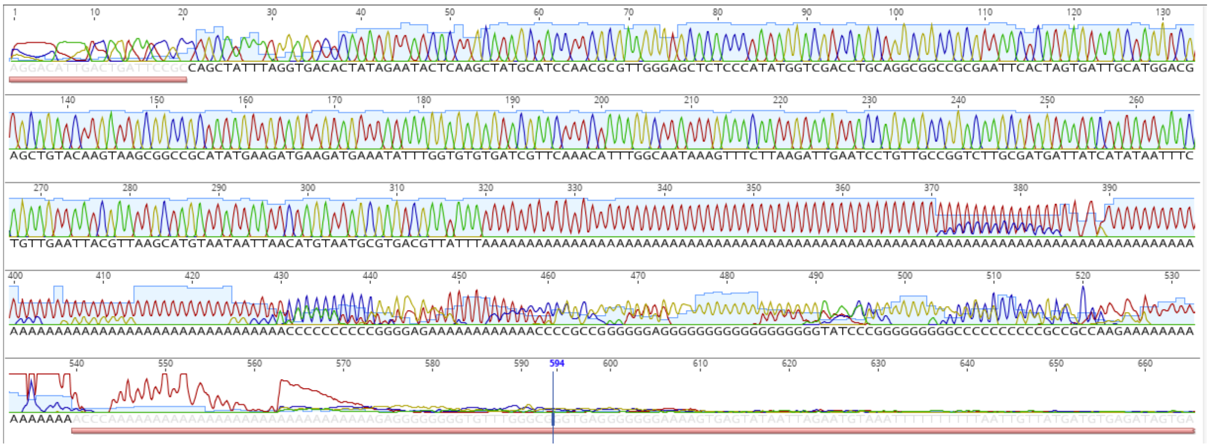

1EA6SAA000_H08_premix.ab1

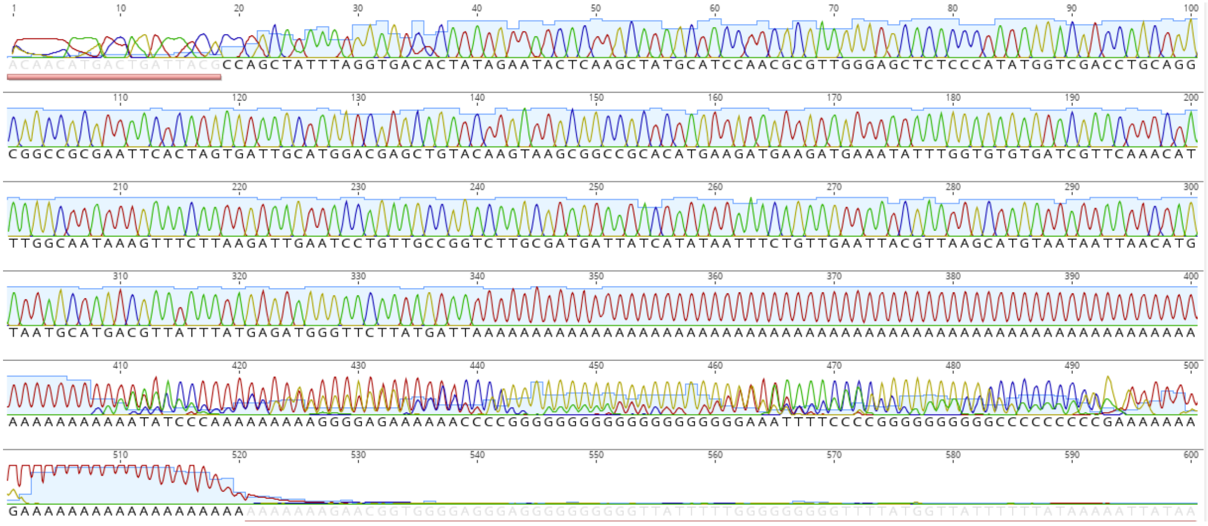

1EA6SAA000_B09_premix.ab1

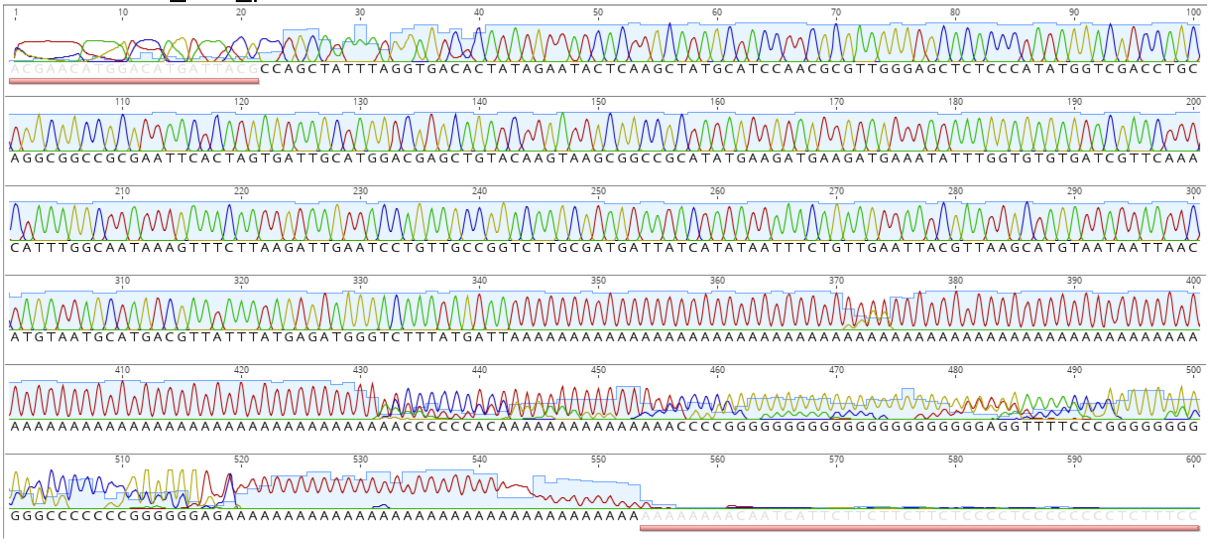

1EA6SAA000_C09_premix.ab1

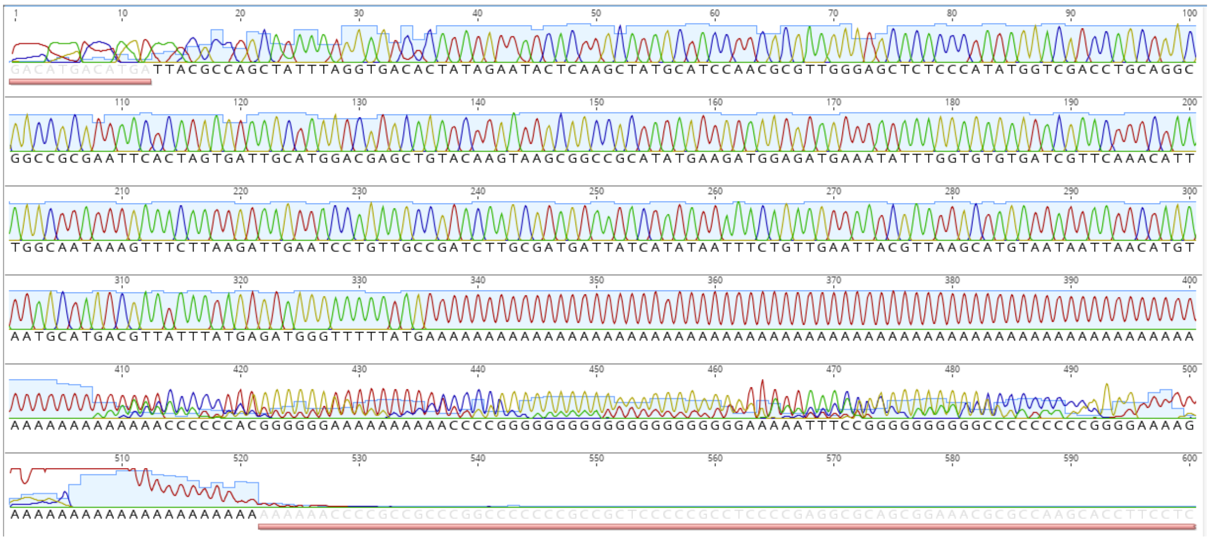

1EA6SAA000_D09_premix.ab1

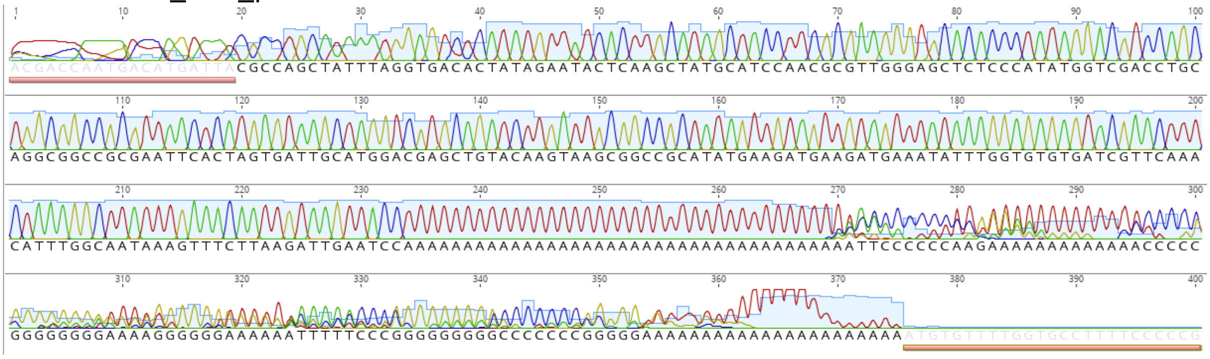

1EA6SAA000_E09_premix.ab1

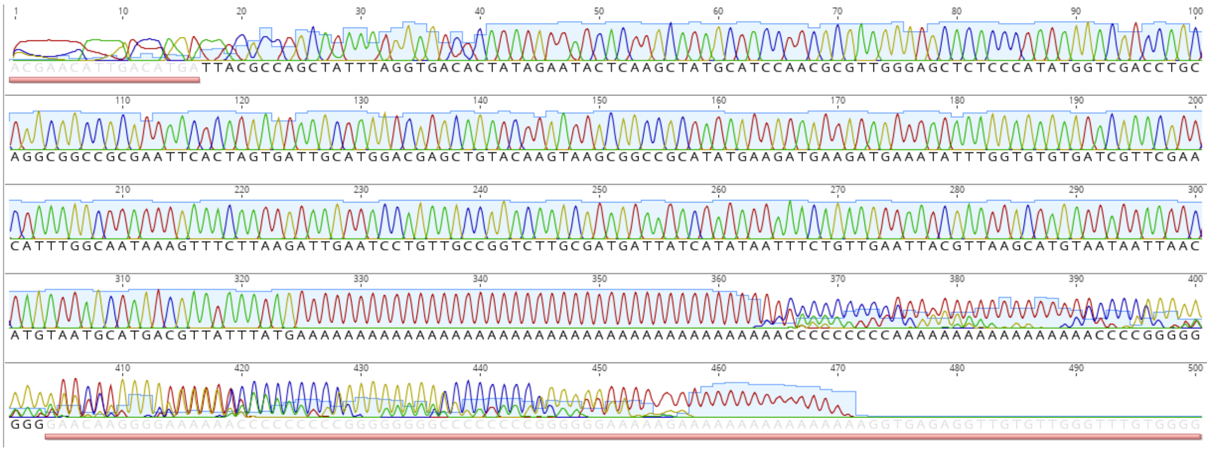

1EA6SAA000_F09_premix.ab1

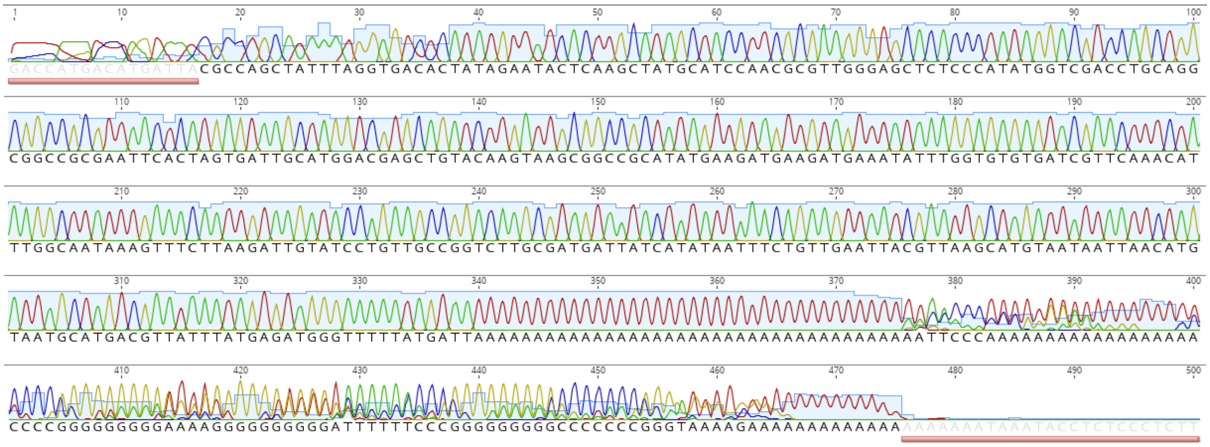

1EA6SAA000_G09_premix.ab1

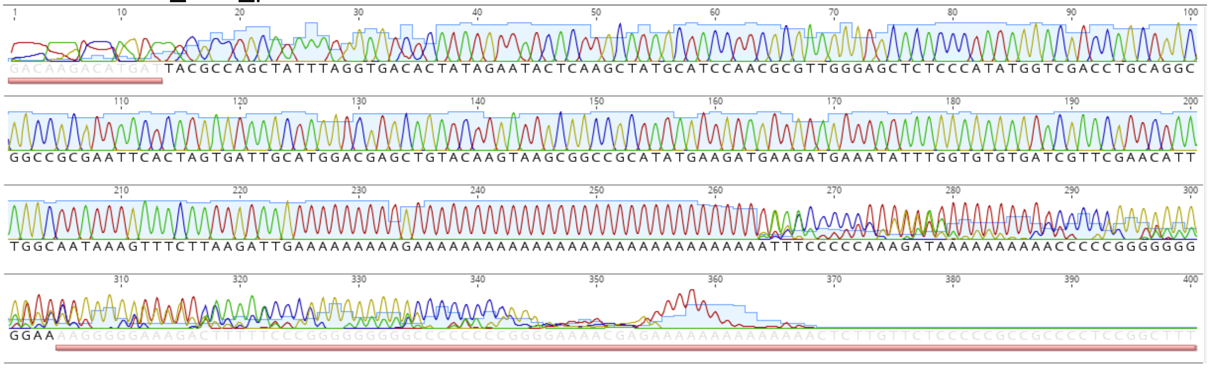

1EA6SAA000_H09_premix.ab1

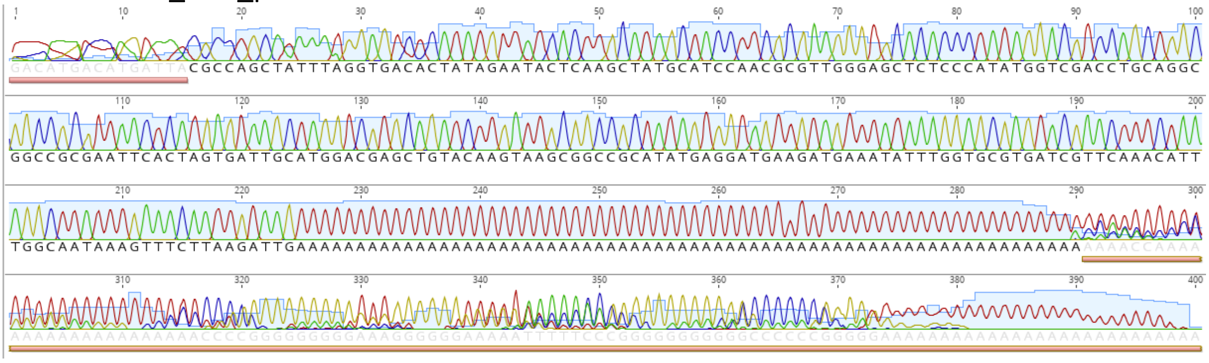

1EA6SAA000_A10_premix.ab1

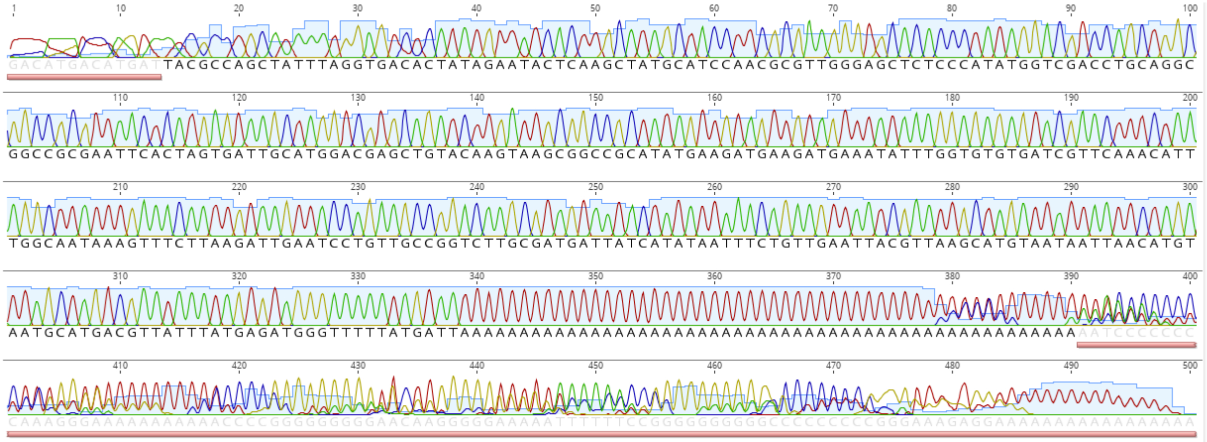

1EA6SAA000_B10_premix.ab1

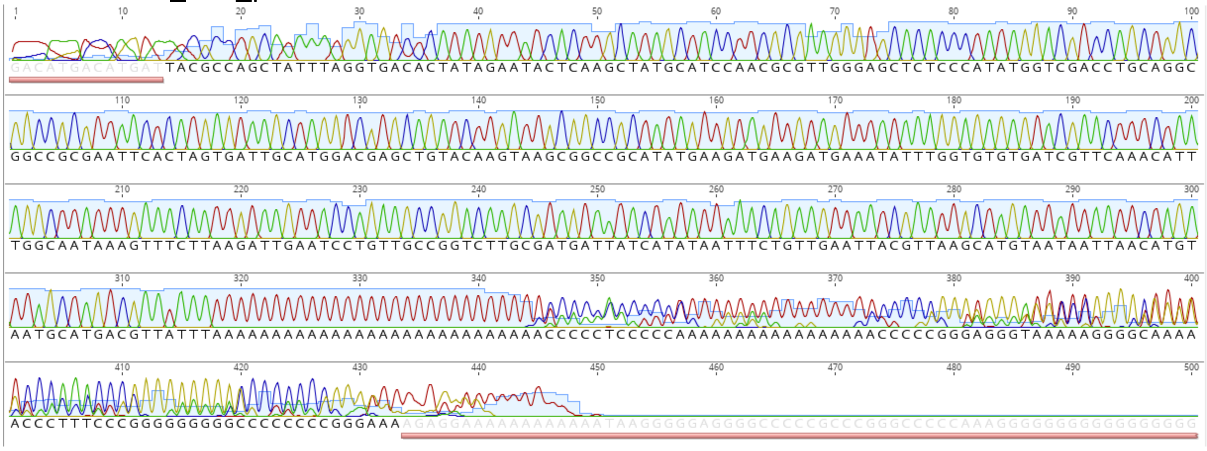

1EA6SAA000_C10_premix.ab1

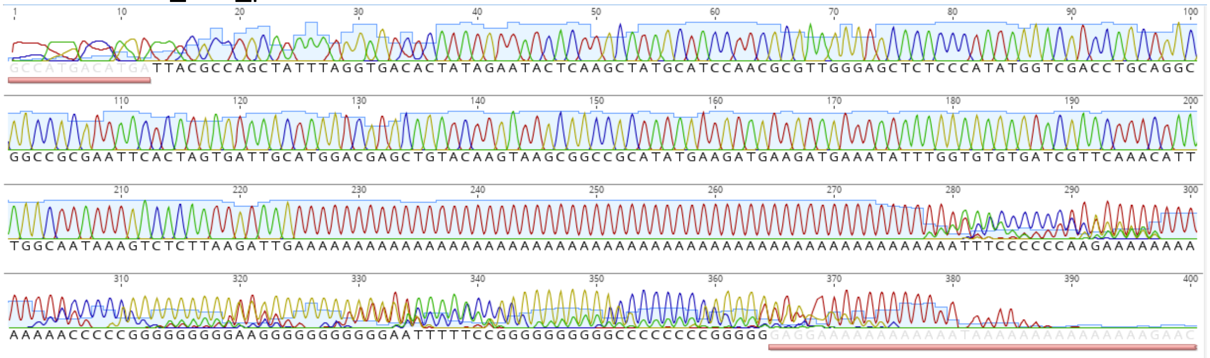

tH4

1E20ZAC084_premix.ab1

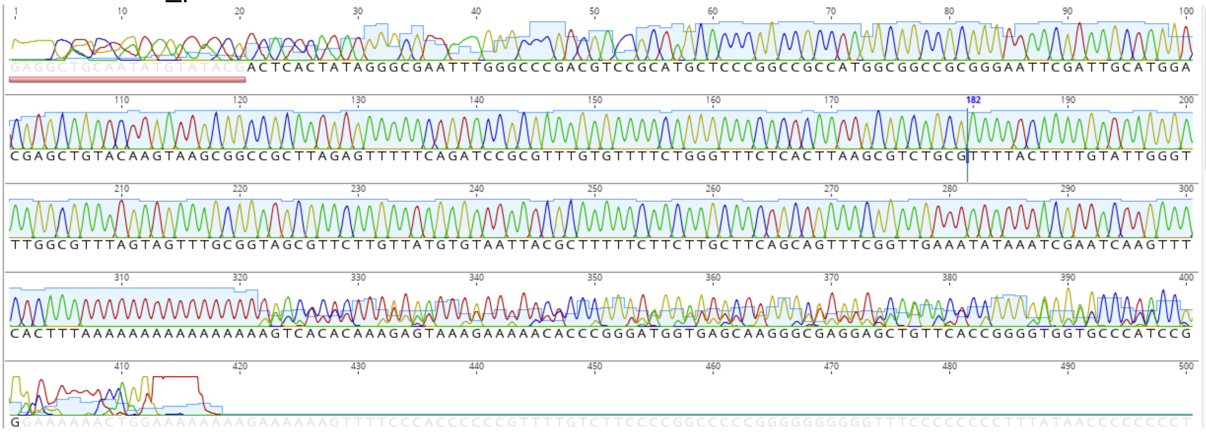

1E20ZAC085_premix.ab1

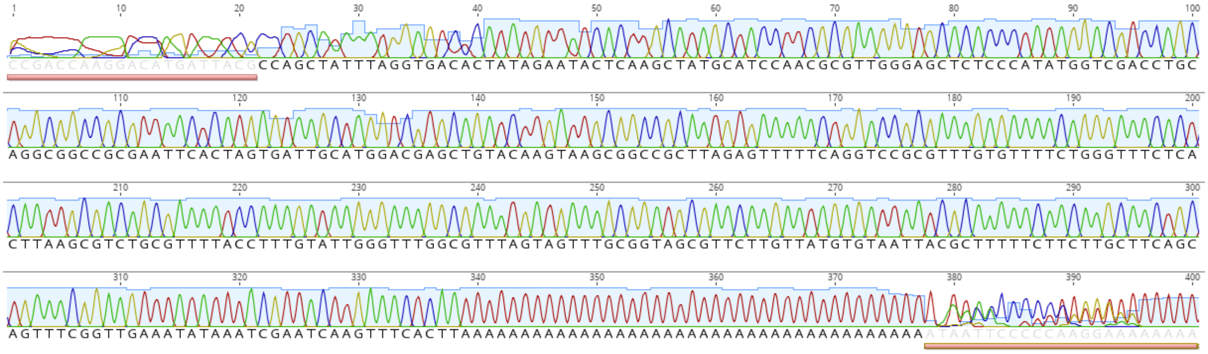

1E20ZAC086_premix.ab1

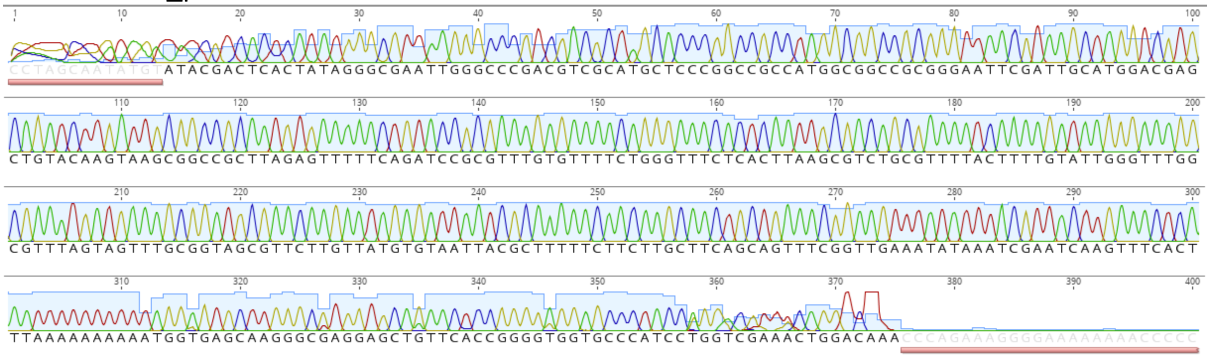

1E20ZAC087_premix.ab1

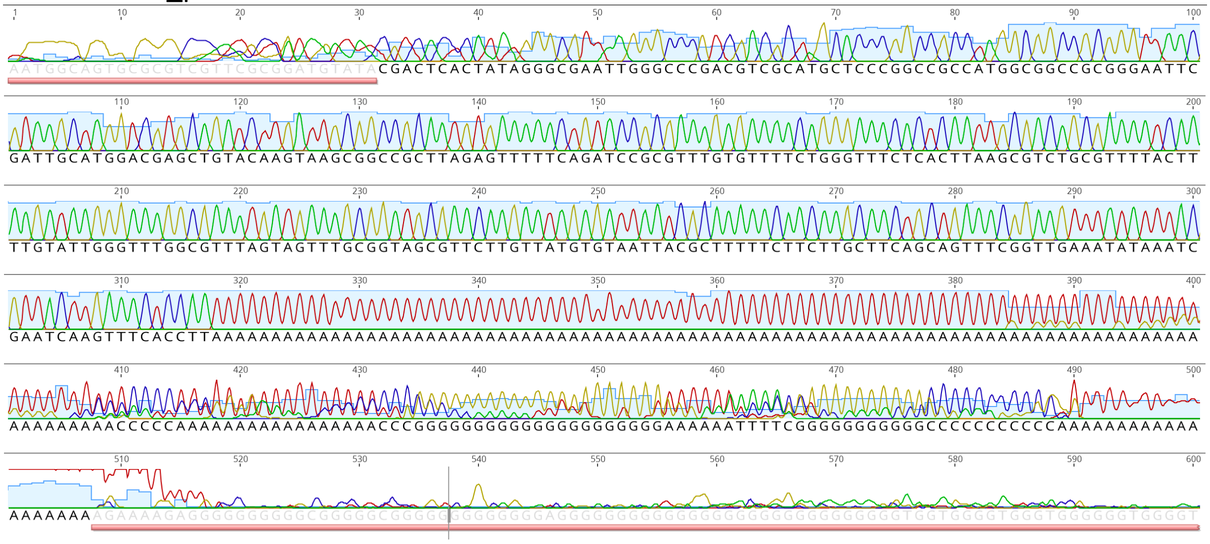

1E20ZAC088_premix.ab1

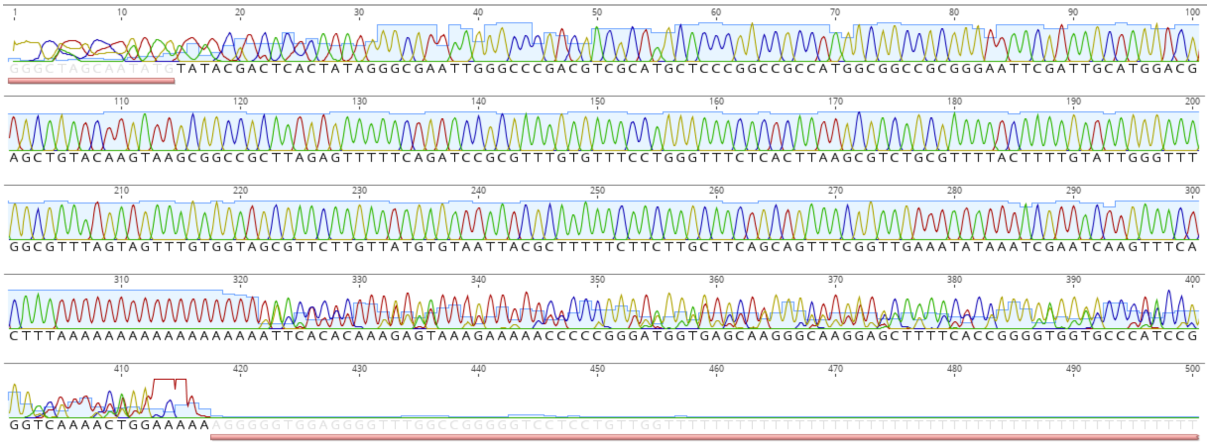

1E20ZAC089_premix.ab1

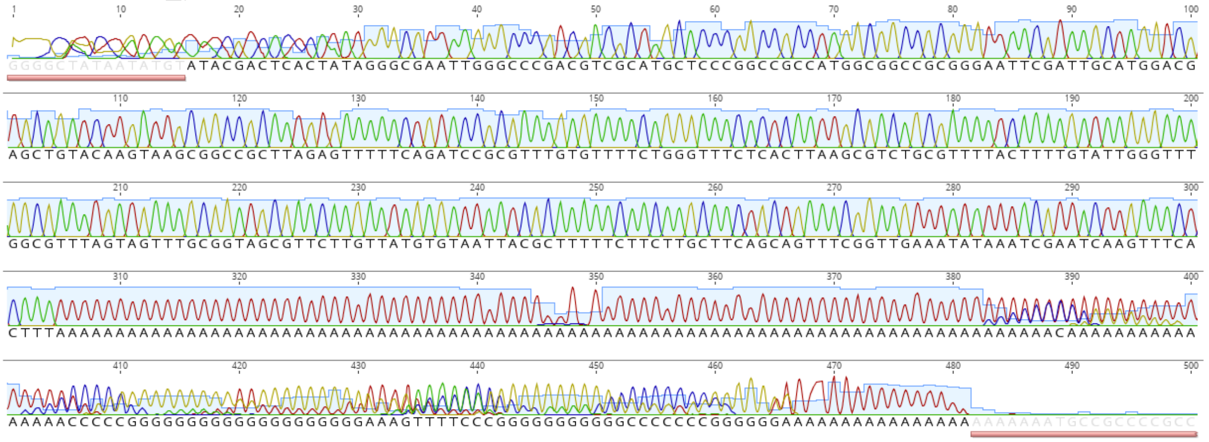

1E20ZAC090_premix.ab1

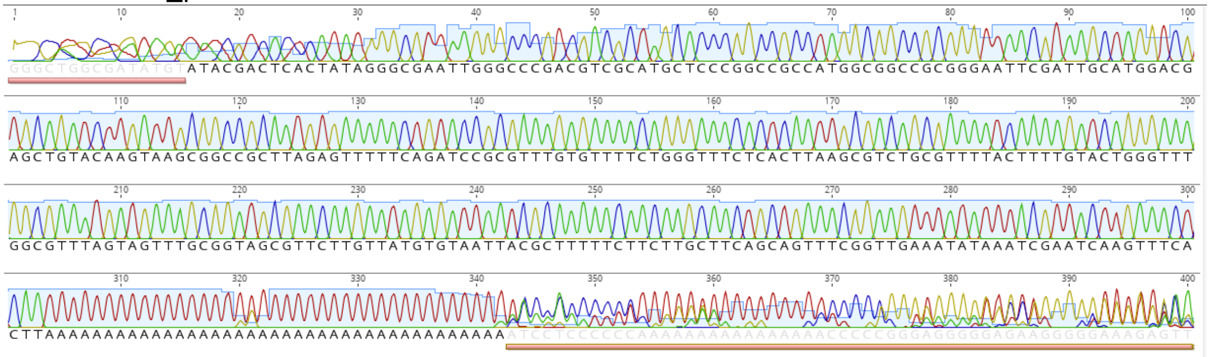

1E20ZAC091_premix.ab1

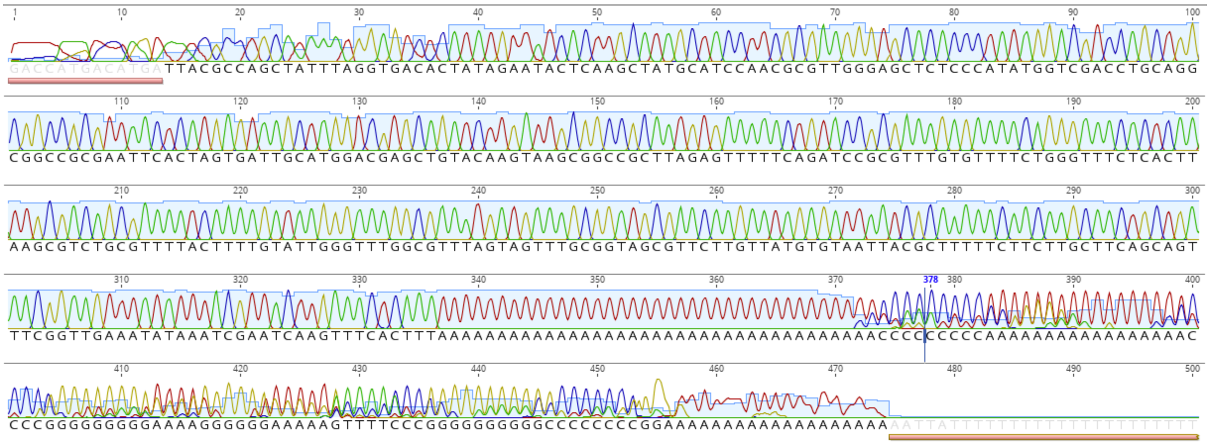

1E20ZAC092_premix.ab1

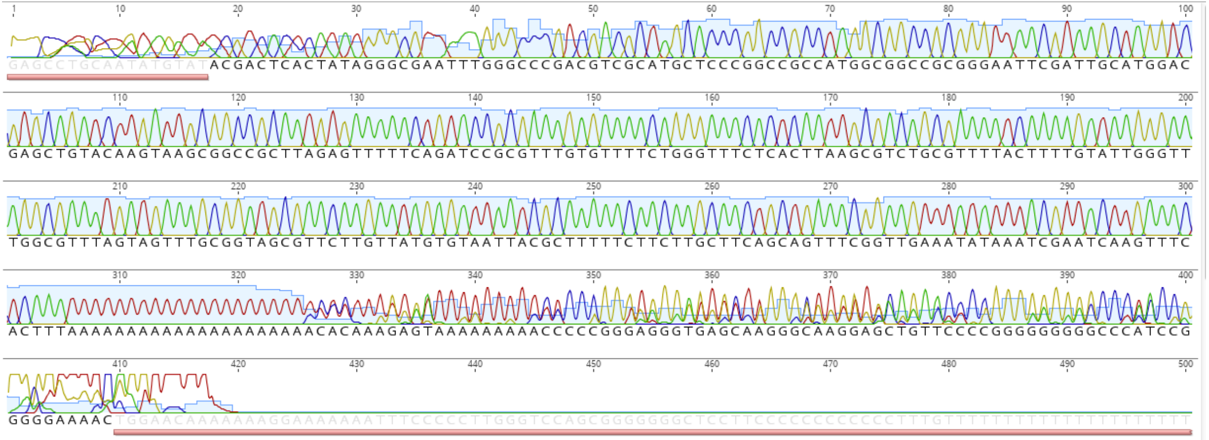

1E20ZAC093_premix.ab1

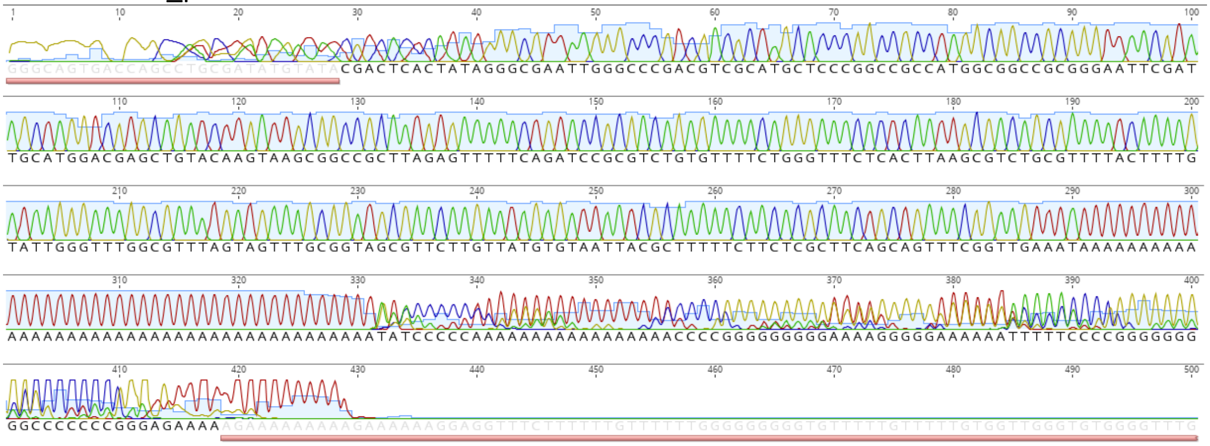

1E20ZAC094_premix.ab1

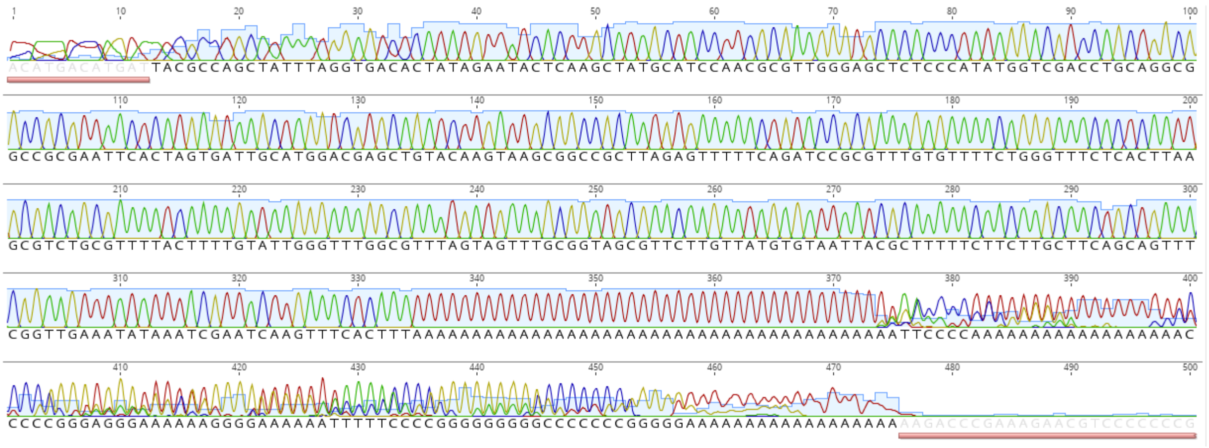

1E20ZAC095_premix.ab1

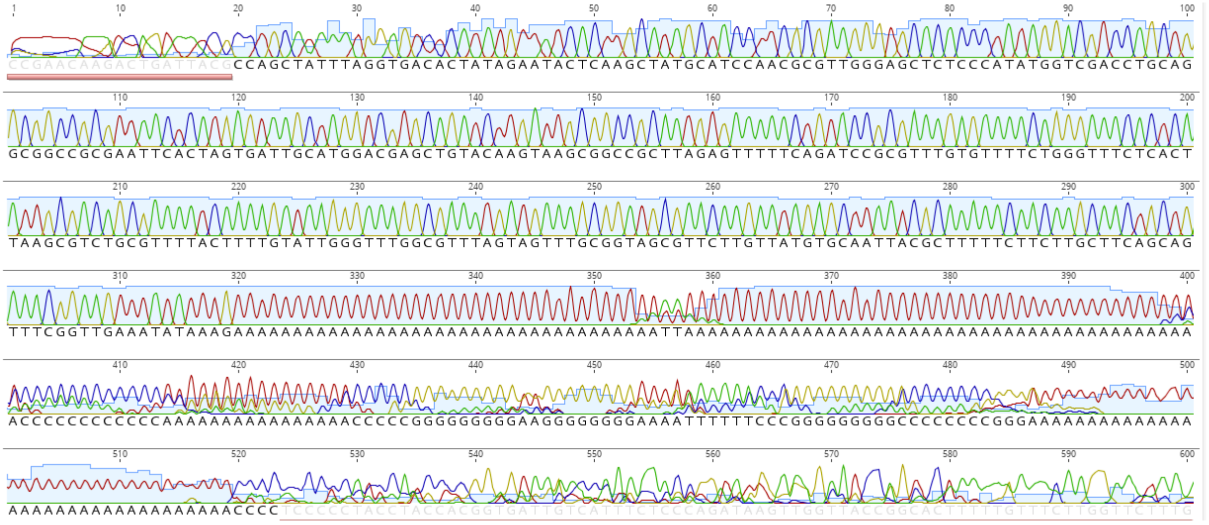

1E20ZAC096_premix.ab1

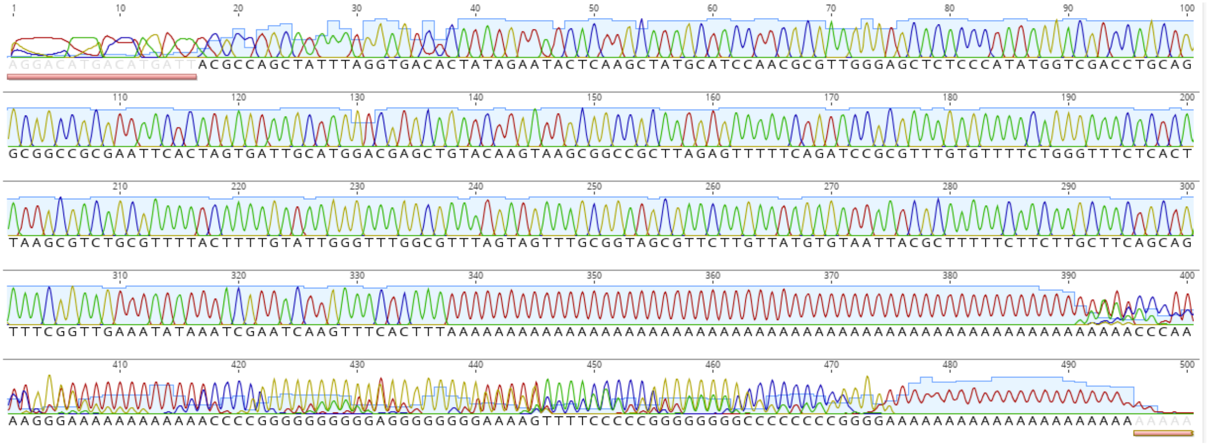

1E20ZAC097_premix.ab1

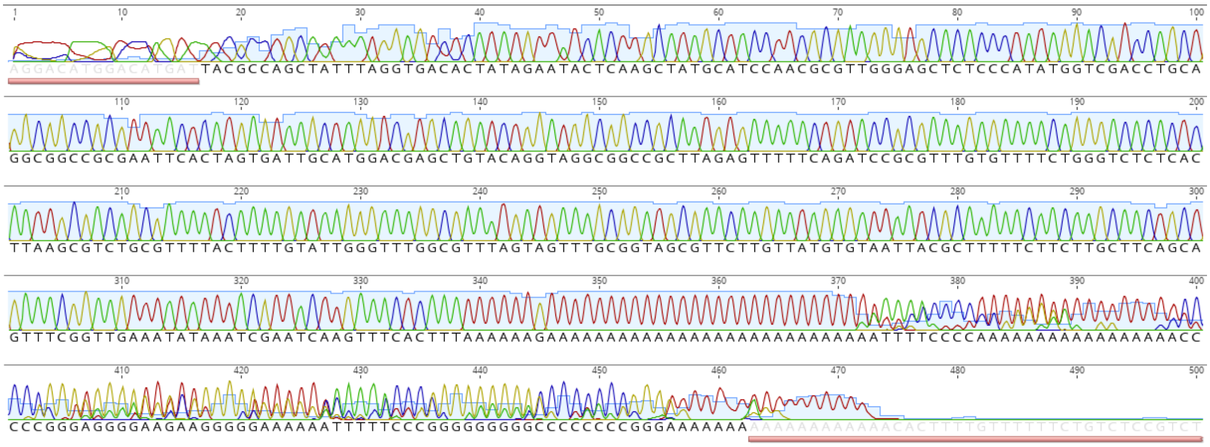

1E20ZAC098_premix.ab1

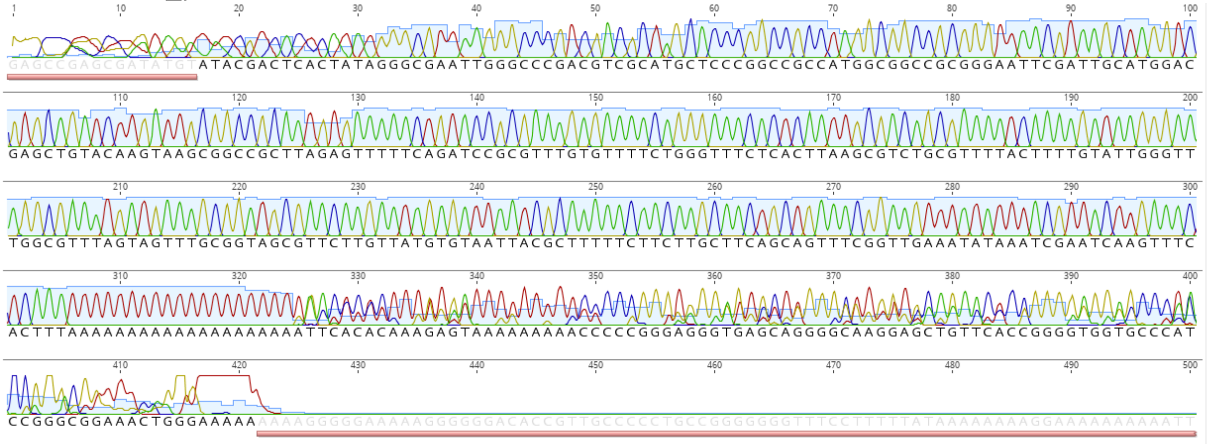

tH4_tHSP_5’

1E20ZAC075_premix.ab1

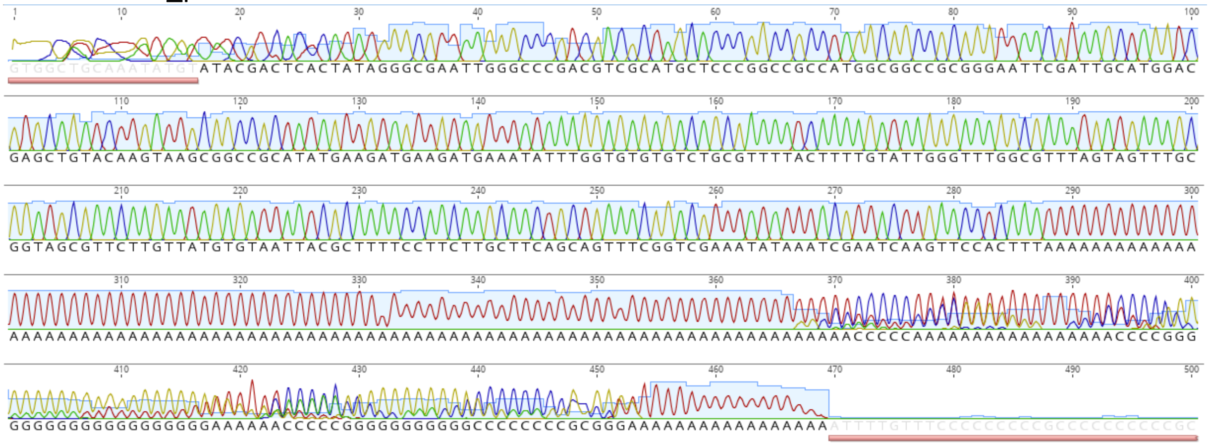

1E20ZAC076_premix.ab1

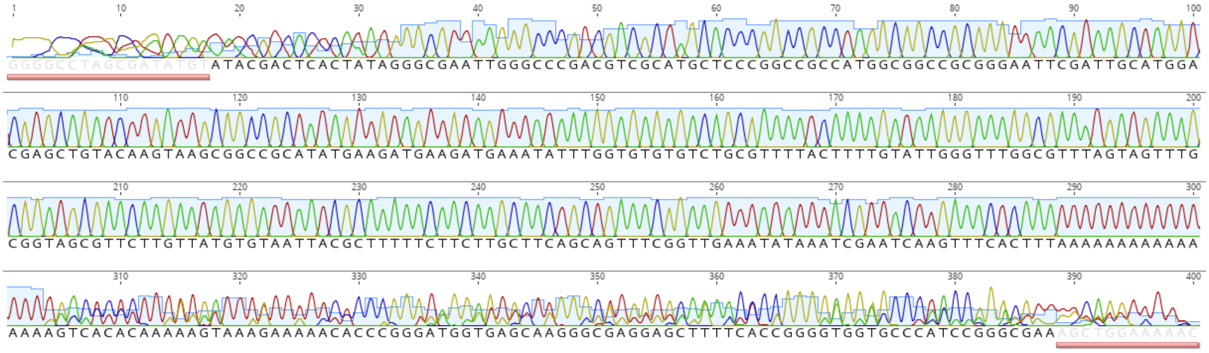

1E20ZAC077_premix.ab1

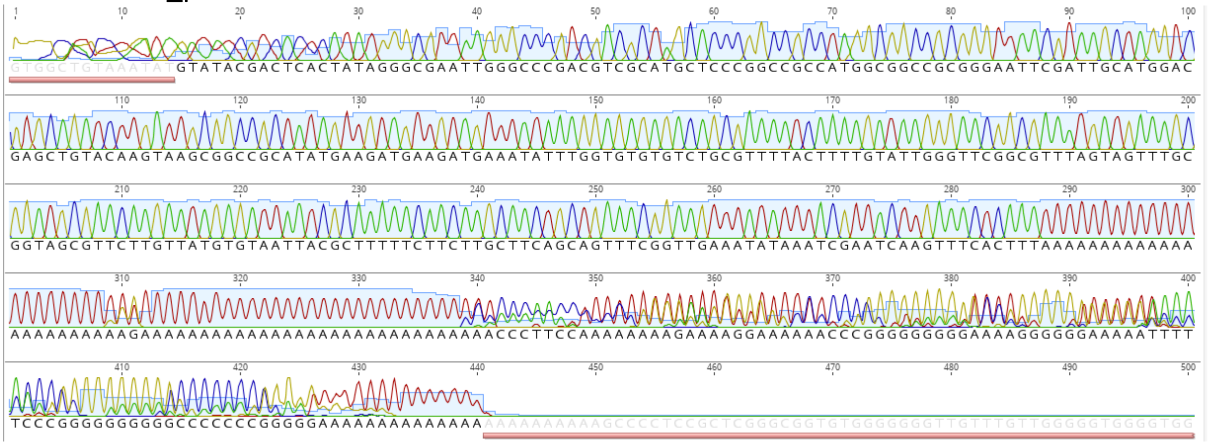

1E20ZAC078_premix.ab1

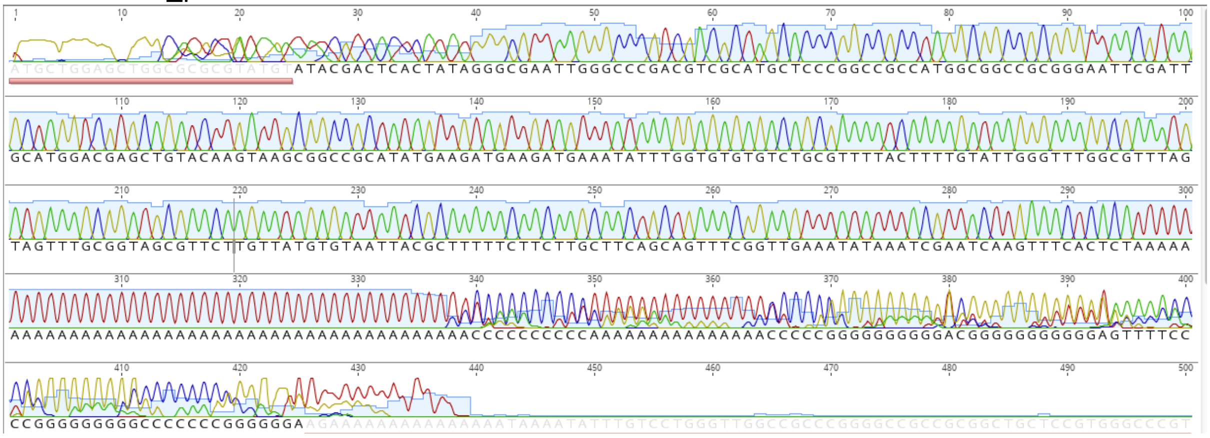

1E20ZAC079_premix.ab1

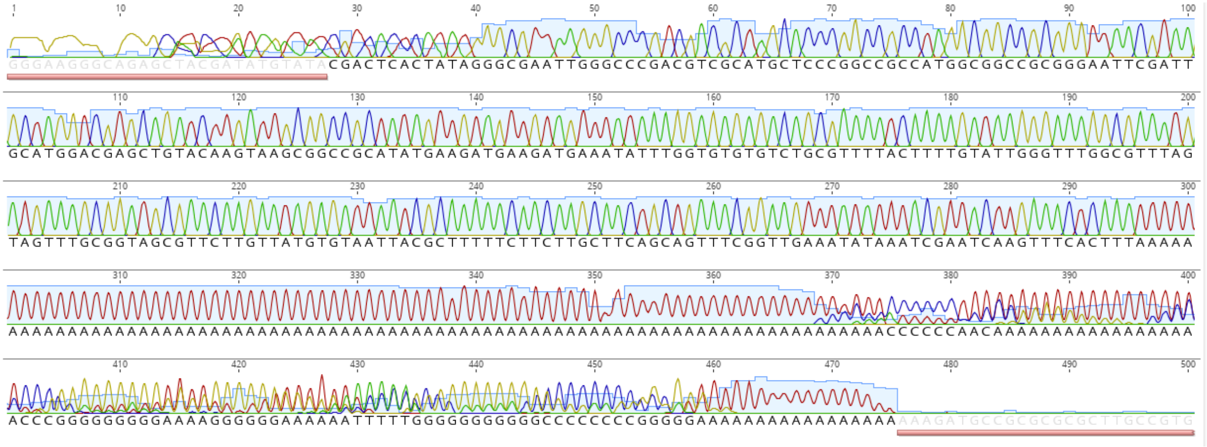

1E20ZAC080_premix.ab1

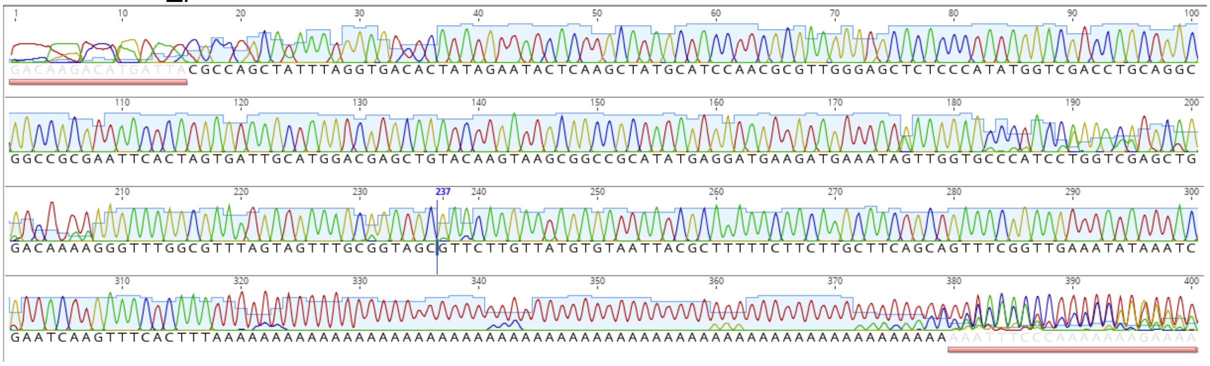

1E20ZAC081_premix.ab1

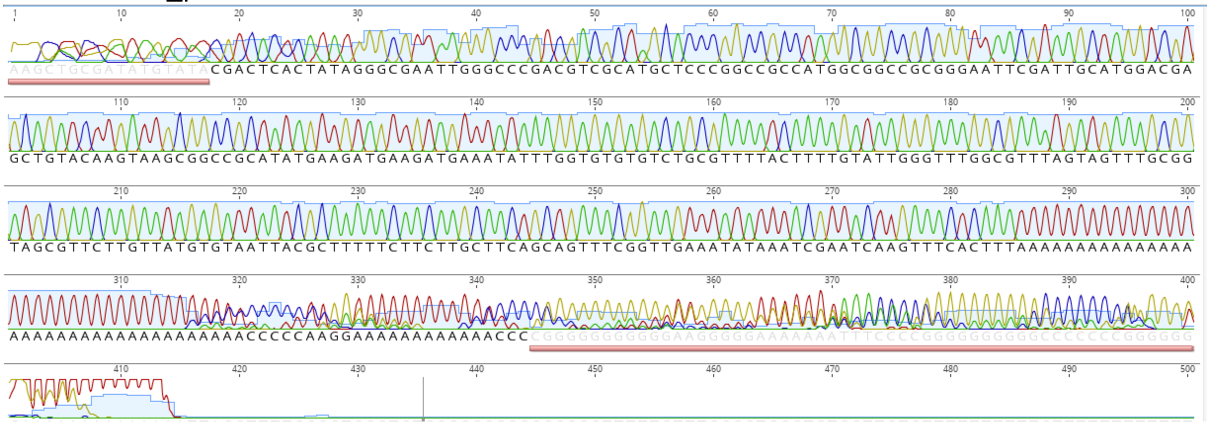

1E20ZAC082_premix.ab1

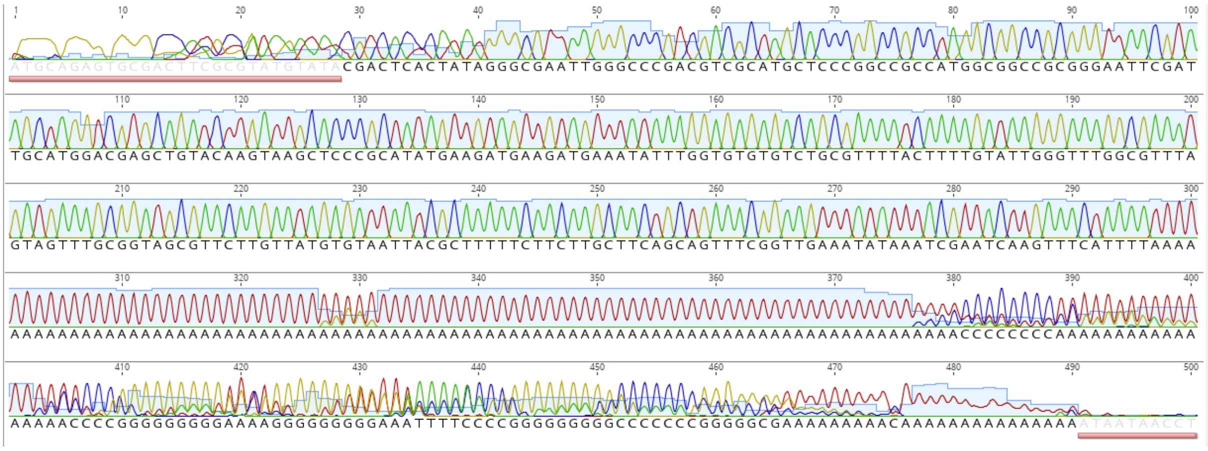

1E20ZAC083_premix.ab1

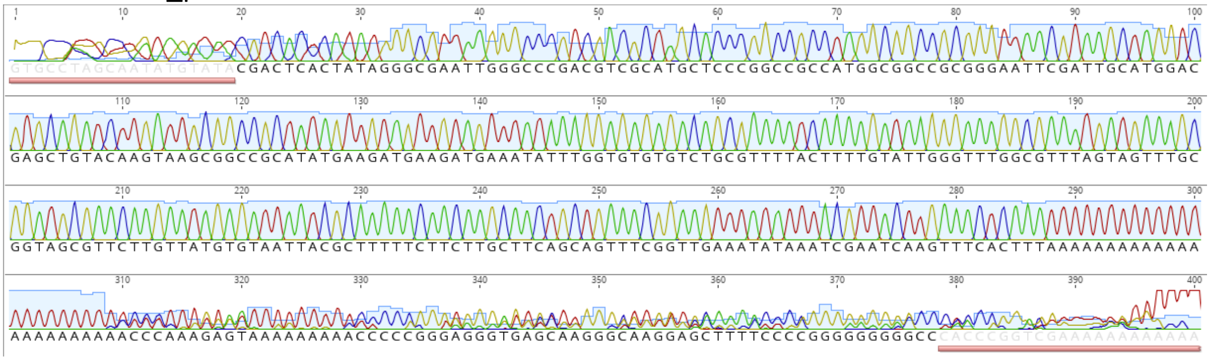

1EA6SAA000_C12_premix.ab1

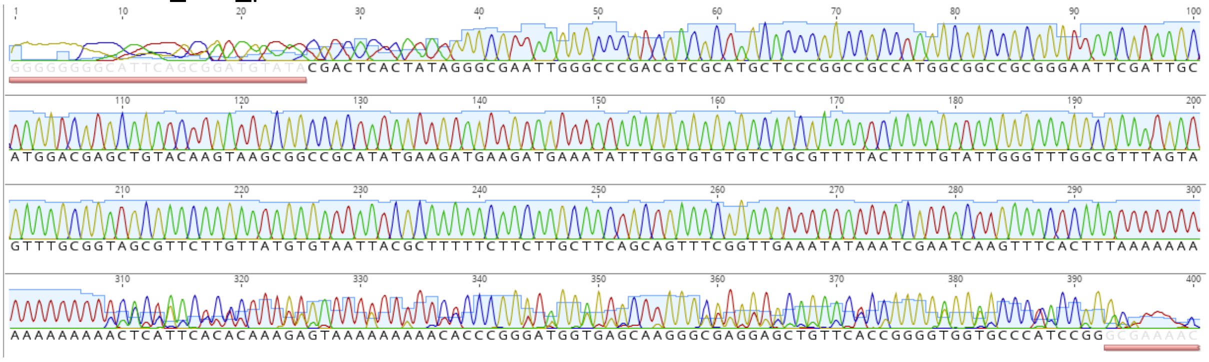

1EA6SAA000_D12_premix.ab1

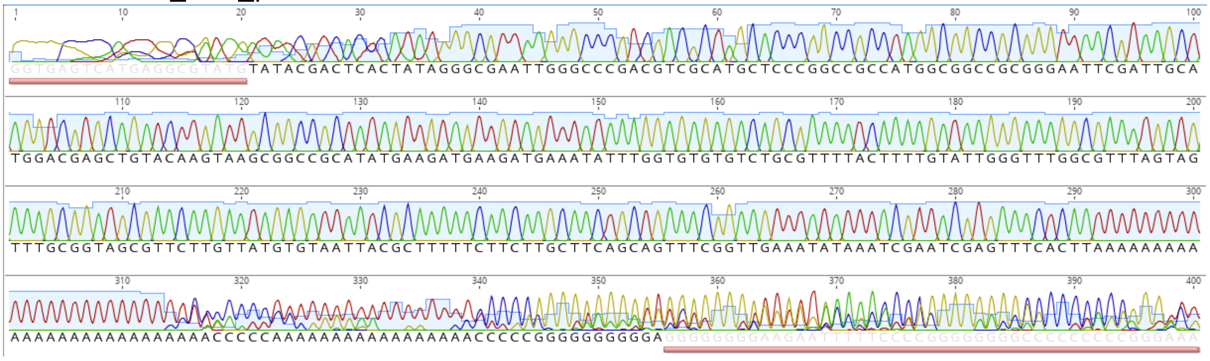

1EA6SAA000_F12_premix.ab1

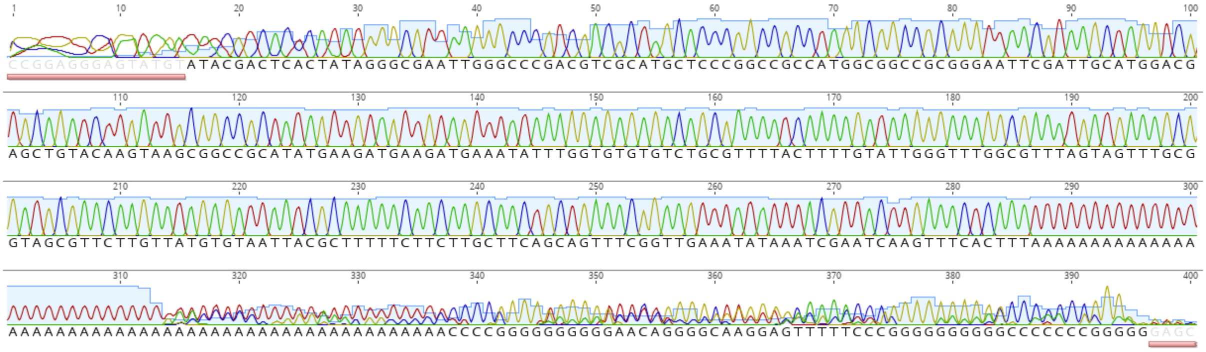

1EA6SAA000_G12_premix.ab1

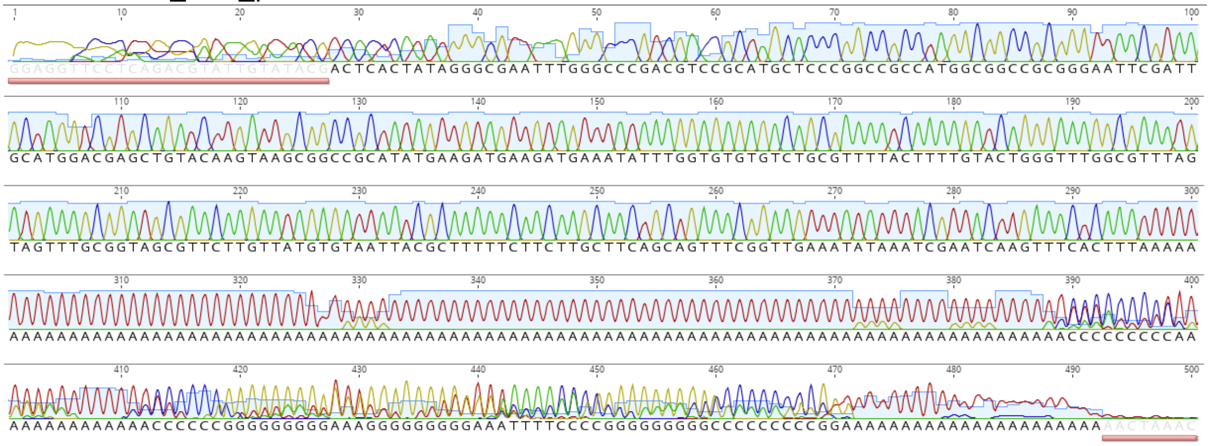

1EA6SAA000_H12_premix.ab1

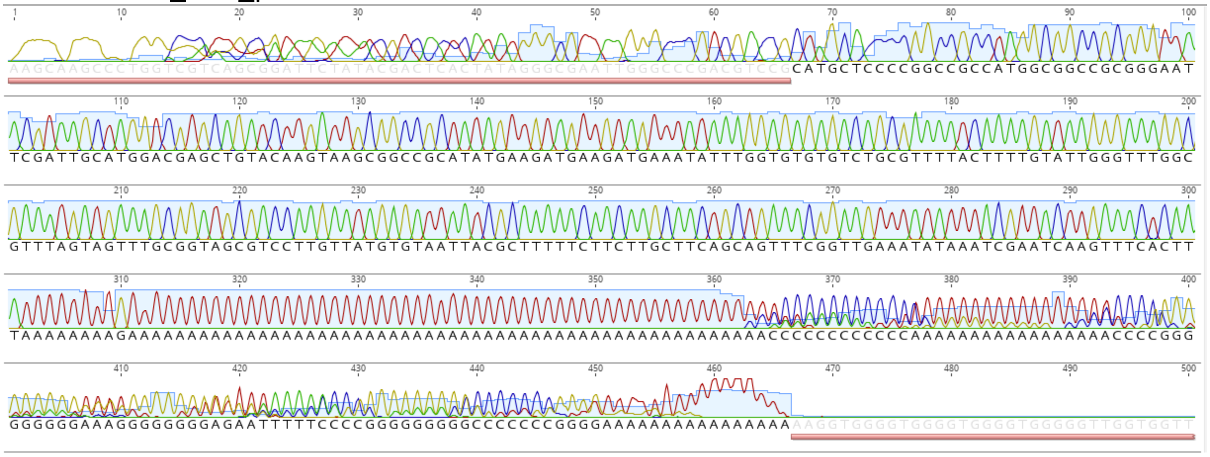

Supplement: Supplementary file 4 [file Data_Sheet_3.PDF]
