# Supplementary material for: Identification of a Transferrable Terminator Element That Inhibits Small RNA Production and Improves Transgene Expression Levels
Source: Front Plant Sci. 2022 May 16;13:877793. doi: 10.3389/fpls.2022.877793 (PMC9149433; doi:10.3389/fpls.2022.877793)
Supplement: Supplementary file 6 [file Data_Sheet_5.docx]

**Supplementary Material and Methods**

*RNA secondary structure analysis*

RNA secondary structure *in silico* prediction was done using RNAfold (Gruber *et al*., The Vienna RNA Websuite. Nucleic Acids Research, Volume 36, Issue suppl_2, 1 July 2008, Pages W70-W74), standard parameters, but temperature set at 23ºC.

*Search for RNA binding motifs*

The analysis of putative motifs for RNA binding protein present in the tHSP 5’ region was done using the Tomtom motif comparison feature (Gupta *et al*., Quantifying similarity between motifs, Genome Biology, 8(2):R24, 2007) in the MEME Suite tool (meme-suite.org, Bailey *et al*., "The MEME Suite", Nucleic Acids Research, 43(W1):W39-W49, 2015). For this analysis, our input was the first 18 nt of the tHSP RNA sequence, with “RNA – Ray2013 all species” as the motif database.

*Plasmid design*

**tACS2_5’_del**: the deleted version of the tACS2 was generated by PCR using the primers FQ-182 (AAAGCGGCCGCTATGTGTTTGATTCTGTTTCTTTTTTCCGC) + FQ-077 (AAACCATGGATAATGATTATTTTAGAAAAAG). The fragment was used to replace the terminator in the pRBCS::GiFiP::tNOS vector using the NotI and NcoI sites.

**tACS2_tNOS_5’:** a gBlocks (Integrate DNA Technologies, IDT) with the terminator sequence was synthesized and PCR amplified using Nos(NotI)-F (ATTGCGGCCGCGATCGTTCAAACATTTGGCAA) and FQ-077 primers. The fragment was used to replace the terminator in the pRBCS::GiFiP::tNOS vector using the NotI and NcoI sites.

**tACS2_tRBCS_5’:** a gBlocks with the terminator sequence was synthesized and PCR amplified using FQ-183 (AAAGCGGCCGCTTTCCCTTTGCTTTTGTG) and FQ-077 primers. The fragment was used to replace the terminator in the pRBCS::GiFiP::tNOS vector using the NotI and NcoI sites.

**tHSP_int∆72:** a gBlocks with the terminator sequence was synthesized and PCR amplified using tHSP(notI)-F (ACTGCGGCCGCATATGAAGATGAAGATGAAATAT) and FQ-077 primers. The fragment was used to replace the terminator in the pRBCS::GiFiP::tHSP vector using the NotI and NcoI site.

*Stable transgenic plants*

*Arabidopsis thaliana* plants (Col-0) were grown in 16 h light/8 h dark conditions at 21ºC. Plants were transformed by floral dipping (Clough SJ and Bent AF, “Floral dip: a simplified method for Agrobacterium-mediated transformation of Arabidopsis thaliana”. Plant J.,16, 735–743, 1998). About 500 µl of T1 seeds were sterilized for 15 min with 75% ETOH + 0.05% Triton x-100 followed by 100% ETOH wash. Seeds were plated on ½ MS supplemented with 12 mg/ml of Glufosinate Ammonium (Sigma-Aldrich) and 250 mg/L Cefotaxime. Plates were kept in dark, at 4ºC for 3 days and then grown in normal conditions for 11 days. Resistant plants were then transferred to soil for another 10 days. The presence of the respective transgene was confirmed by PCR using primers Ben881 (AGCAAAGACCCCAACGAGAA) and FQ-179 (TTTCATCAGACAGTTGAGAATCC). Expected size for tRBCS, tRBCS_tHSP_5’ and tRBCS_2x_tHSP_5’ PCR products were 272 nt, 245 nt and 283 nt, respectively (Figure S4C). GFP was quantified as described in material and methods with the following changes: For input normalization, tissue was collected from the 4^th^ true leaf using a 4 mm cork borer; after grinding the tissue, the sample was resuspended in 100 µl of lysis buffer and 80 µl were further used for GFP fluorescence measurements.

*Estimation of poly(A) tail length using G/I tailing*

The “Poly(A) Tail-Length Assay Kit” (Thermo Fisher Scientific) was used as an alternative method to estimate the poly(A) tail size. G/I tailing, reverse transcription and PCR amplification was done according to manufacturer’s instructions. For gene specific PCR, the primer FQ-178 (GATCACTCTCGGCATGGAC) was used in combination with one of the following oligonucleotides: FQ-179 (TTTCATCAGACAGTTGAGAATCC, for tRBCS); FQ-180 (TTACATGCTTAACGTAATTCAACAG, for tNOS ); FQ-239 (CGAAACTGCTGAAGCAAGAA, for tH4) and FQ-240 (ATGCTACACACCAGCACCAC, for tACS2). For the poly(A) tail PCR, primers FQ-178 and the universal PCR reverse primer (supplied with the kit) were used.

*GFP quantification*

For Figure S2, GFP was quantified using ImageJ (FIJI, http://fiji.sc) (Schindelin et al., 2012 - doi: 10.1038/nmeth.2019). First, images were converted to 16-bit. Next, three independent measurements of each sample were taken using the measure tool. To assure measurements were from the same size, a circular region of interest was created (width = 36, height = 36, area = 1020). To compensate for variations associated to the infiltration method, values were normalized to the closest spot from a infiltration with a reference construct.
