# Supplementary material for: Identification of a Transferrable Terminator Element That Inhibits Small RNA Production and Improves Transgene Expression Levels
Source: Front Plant Sci. 2022 May 16;13:877793. doi: 10.3389/fpls.2022.877793 (PMC9149433; doi:10.3389/fpls.2022.877793)

tHSP

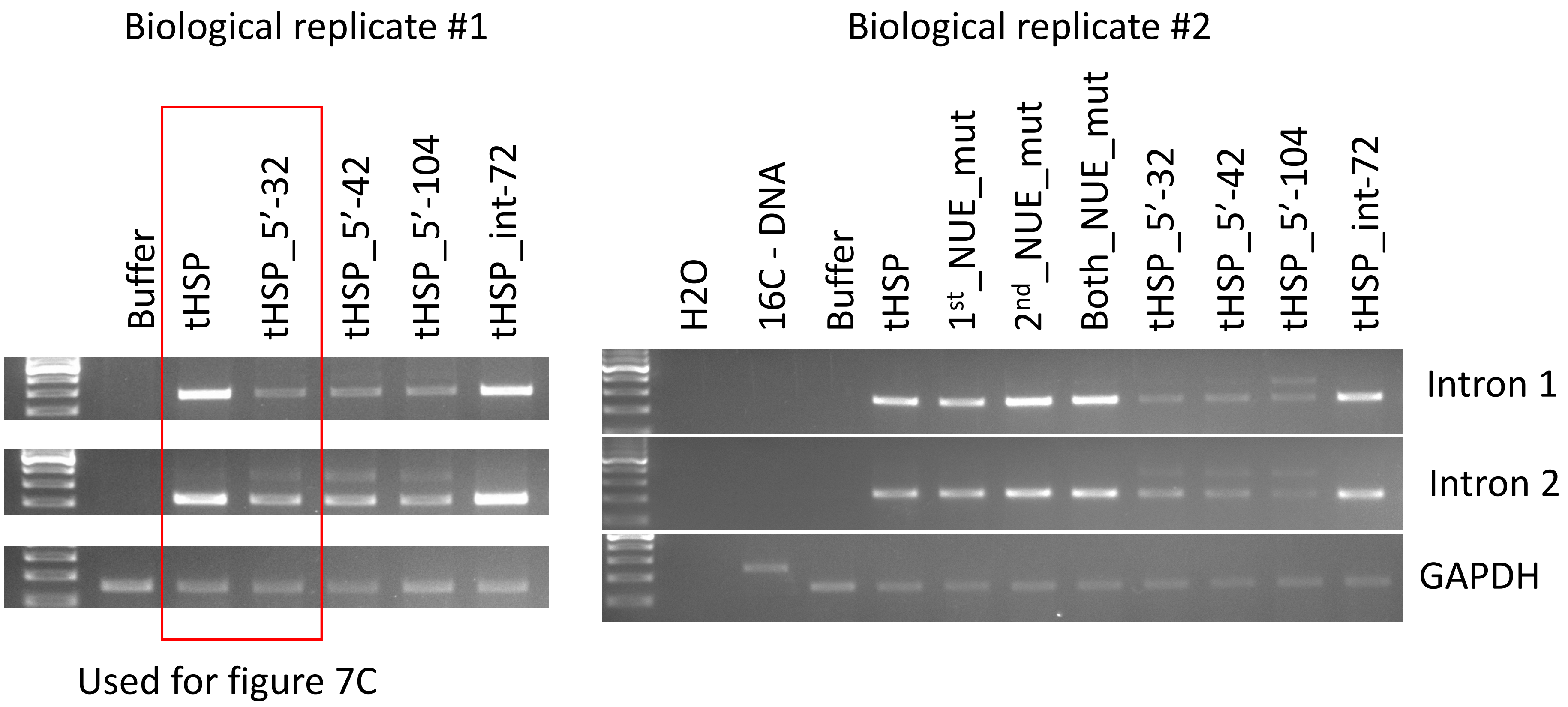

Biological replicate #1
Buffer
tHSP
tHSP_5'-32
tHSP_5'-42
tHSP_5'-104
tHSP_int-72
Used for figure 7C
Biological replicate #2
H2O
16C - DNA
Buffer
tHSP
1st_NUE_mut
2nd_NUE_mut
Both_NUE_mut
tHSP_5'-32
tHSP_5'-42
tHSP_5'-104
tHSP_int-72
Intron 1
Intron 2
GAPDH

tACS2

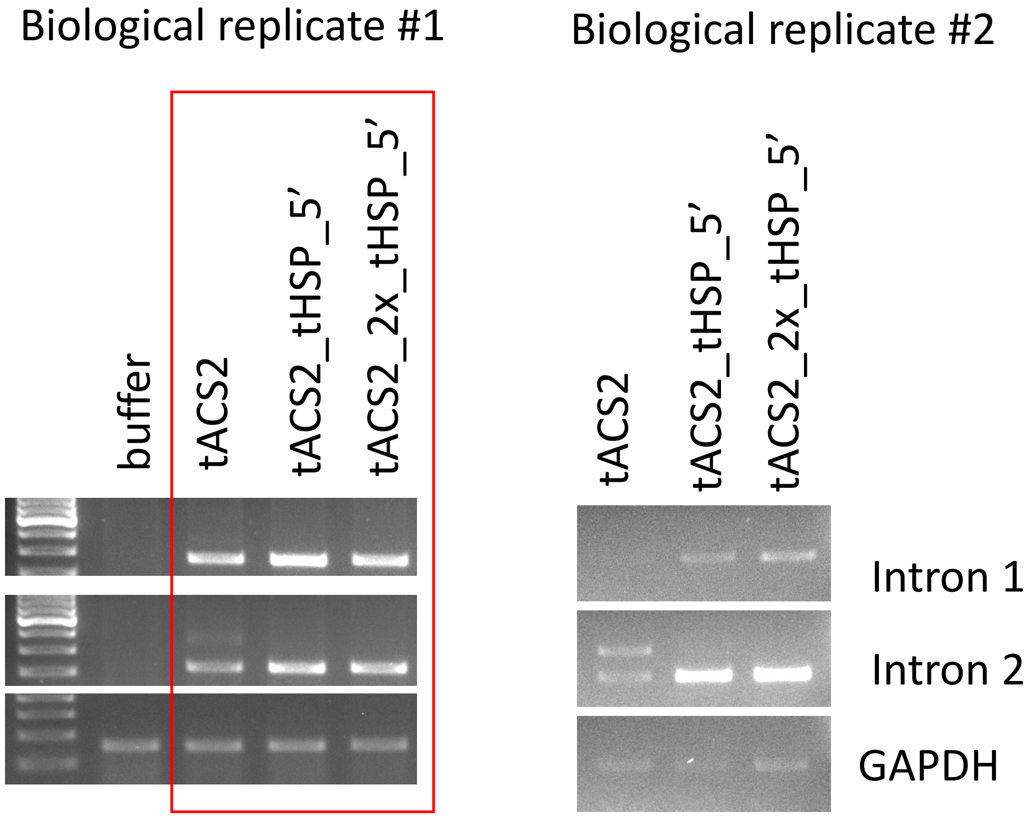

Used for figure 7C

tRBCS

Biological replicate #1
(used for figure 7C)

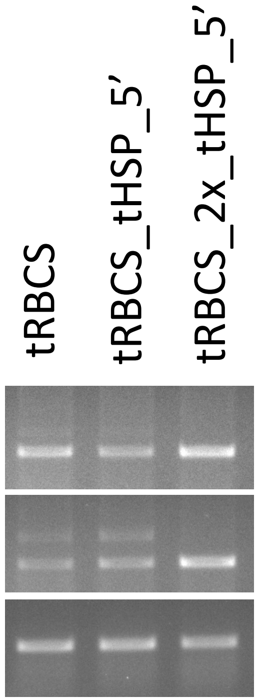

Biological replicate #2

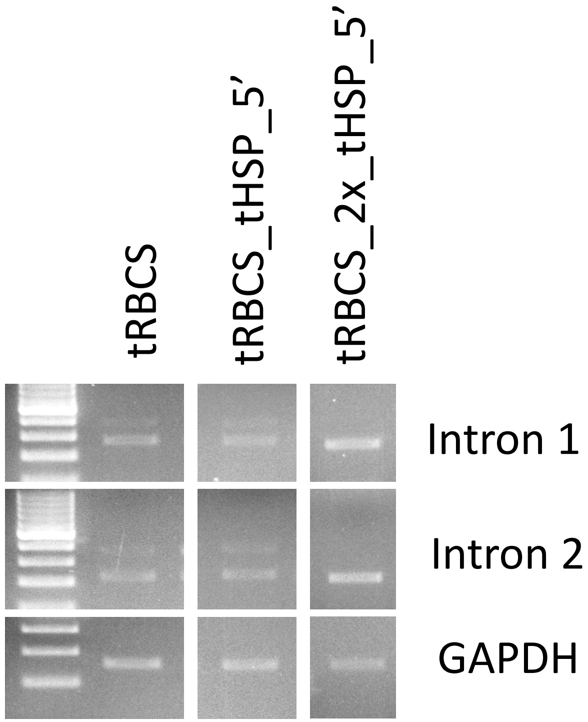

tNOS

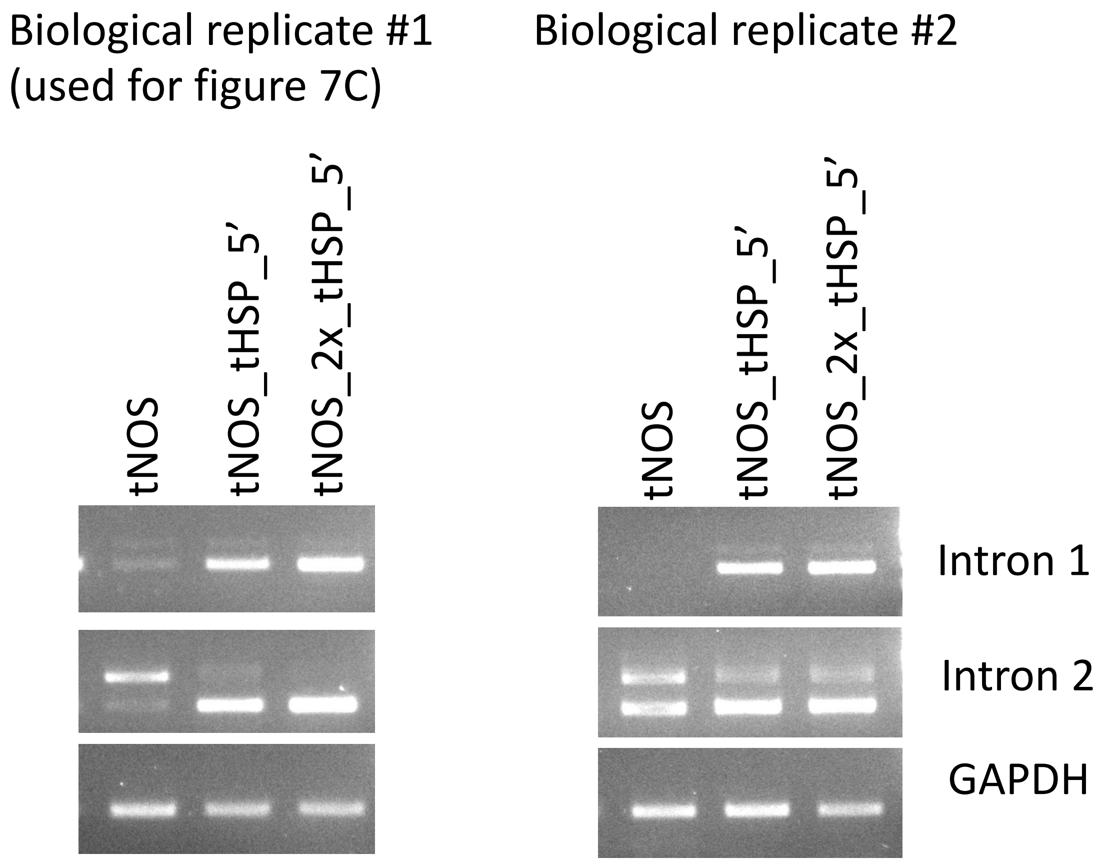
Biological replicate #1
(used for figure 7C)
Biological replicate #2
tNOS
tNOS_tHSP_5’
tNOS_2x_tHSP_5’
tNOS
tNOS_tHSP_5’
tNOS_2x_tHSP_5’
Intron 1
Intron 2
GAPDH

tH4

Biological replicate #1
(used for figure 7C)

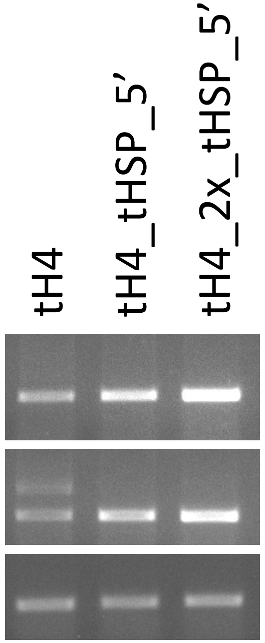

Biological replicate #2

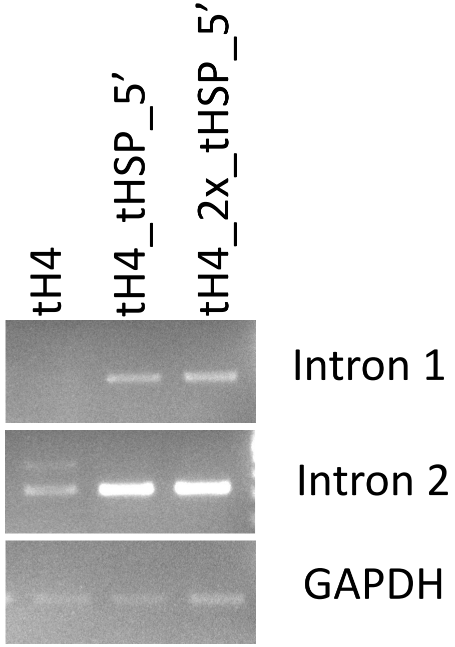

Supplement: Supplementary file 7 [file Data_Sheet_6.PDF]
