# Supplementary material for: Identification of a Transferrable Terminator Element That Inhibits Small RNA Production and Improves Transgene Expression Levels
Source: Front Plant Sci. 2022 May 16;13:877793. doi: 10.3389/fpls.2022.877793 (PMC9149433; doi:10.3389/fpls.2022.877793)
Supplement: Supplementary file 8 [file Data_Sheet_7.PDF]

tHSP

Bio rep #1
(used for figure 7B)

Bio rep #2

Bio rep #3

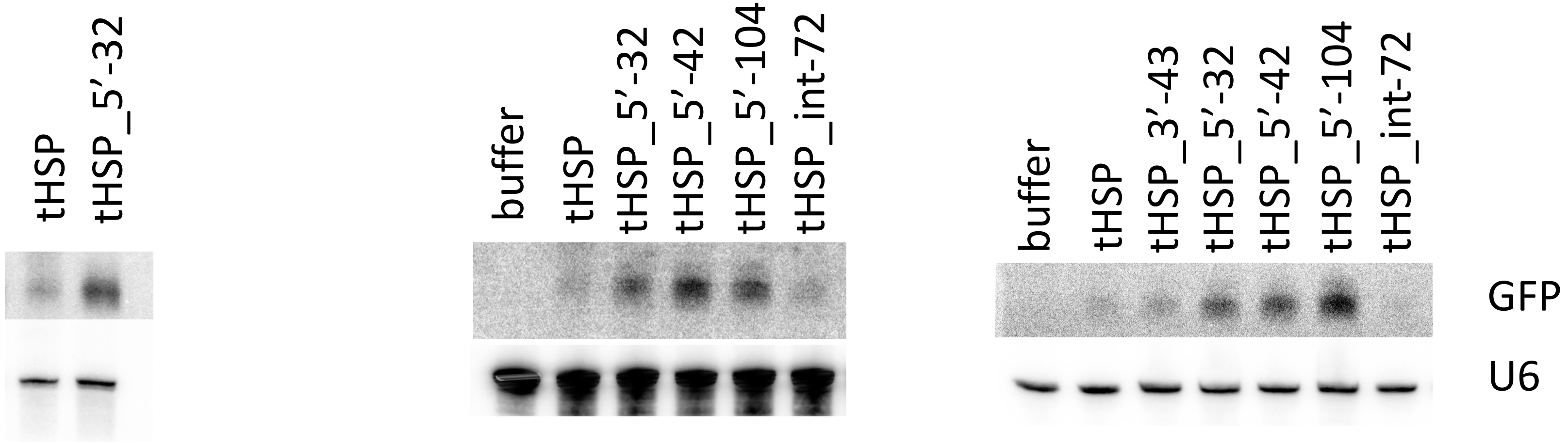

tACS2

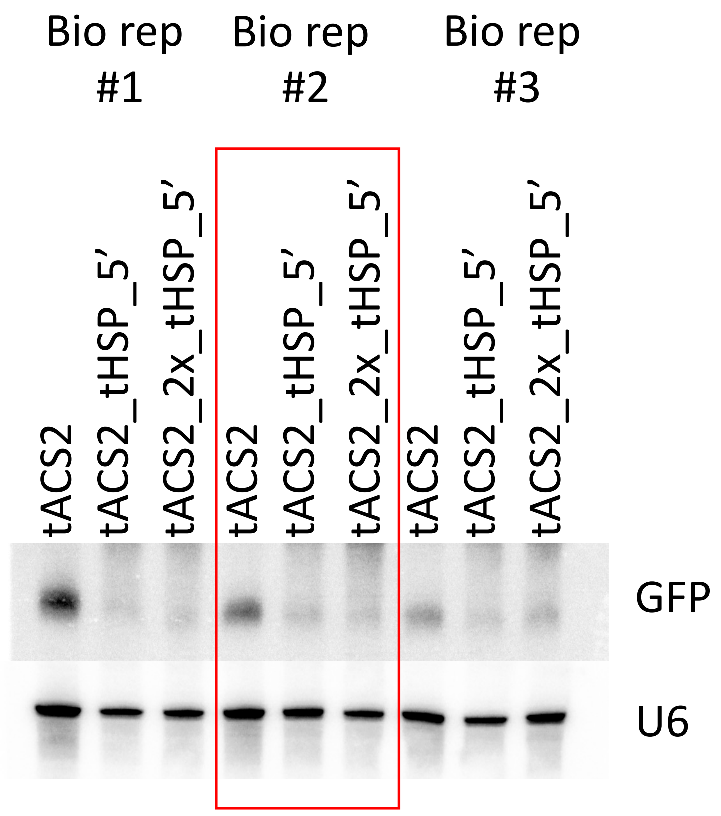

Used for figure 7B

tRBCS

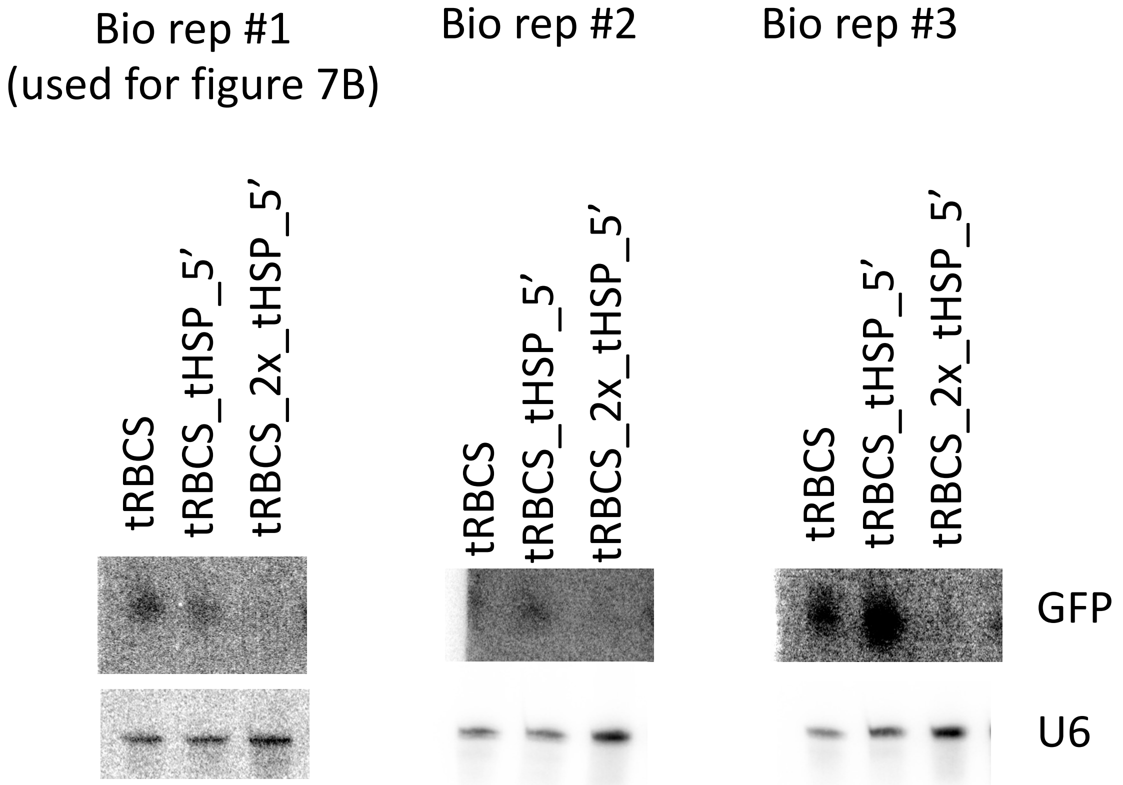

tNOS

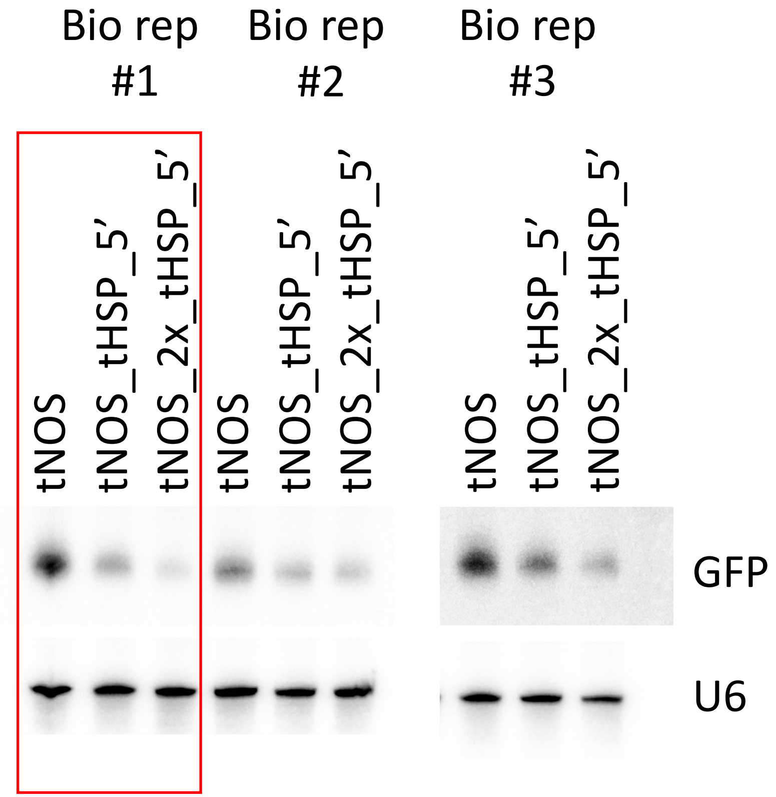

Used for figure 7B

tH4

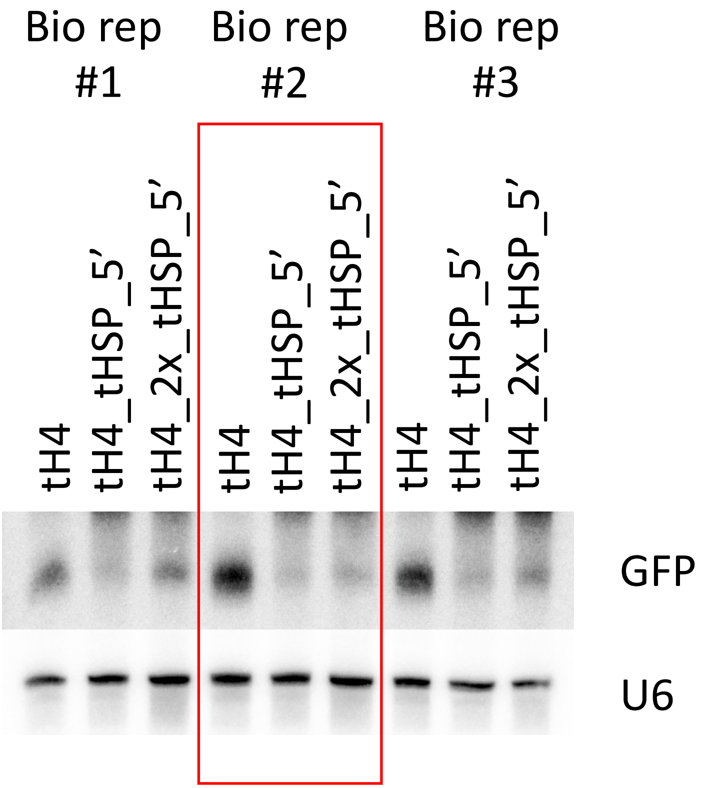

Used for figure 7B
