# Supplementary material for: Identification of a Transferrable Terminator Element That Inhibits Small RNA Production and Improves Transgene Expression Levels
Source: Front Plant Sci. 2022 May 16;13:877793. doi: 10.3389/fpls.2022.877793 (PMC9149433; doi:10.3389/fpls.2022.877793)
Supplement: Supplementary file 9 [file Data_Sheet_8.docx]

**Supplementary Figures**

**
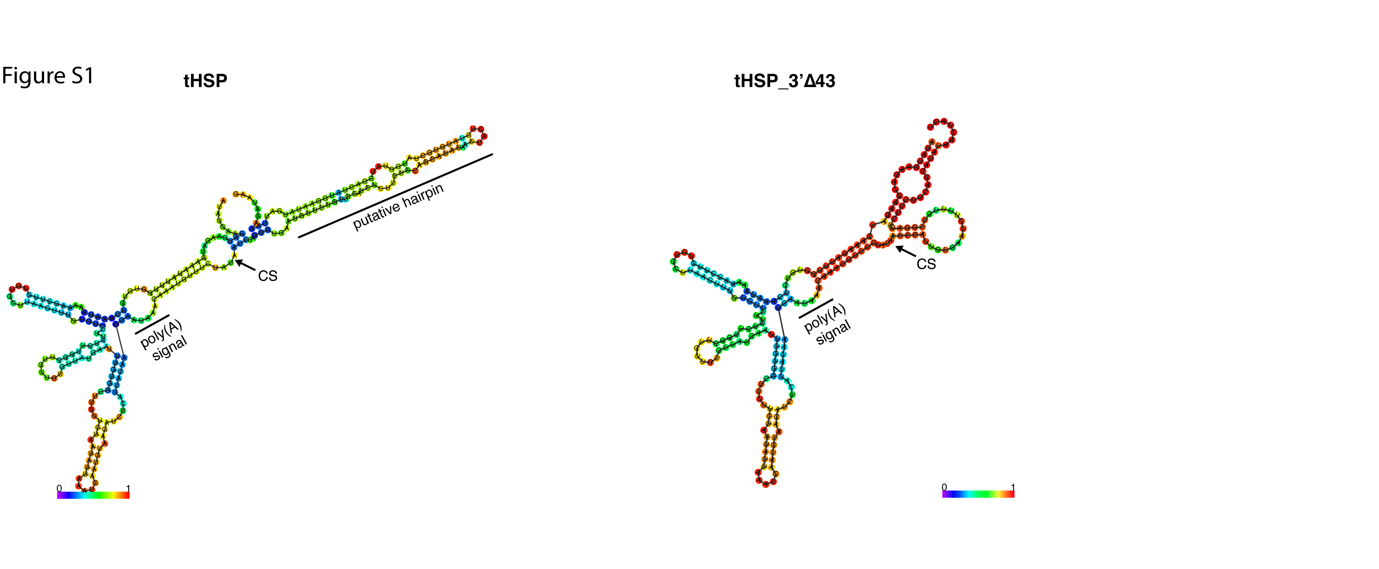
**

**Supplementary Figure 1. Secondary structure of the *HSP18.2* terminator and mutant version. I**n silico prediction of the *HSP18.2* terminator shows the presence of a putative hairpin with the potential to be processed by DCL4. The deletion in tHSP_3’∆43 was designed to eliminate this double-stranded structure without disturbing the remaining architecture of this regulatory element. The poly(A) signal and the cleavage site (CS) are also shown.

**
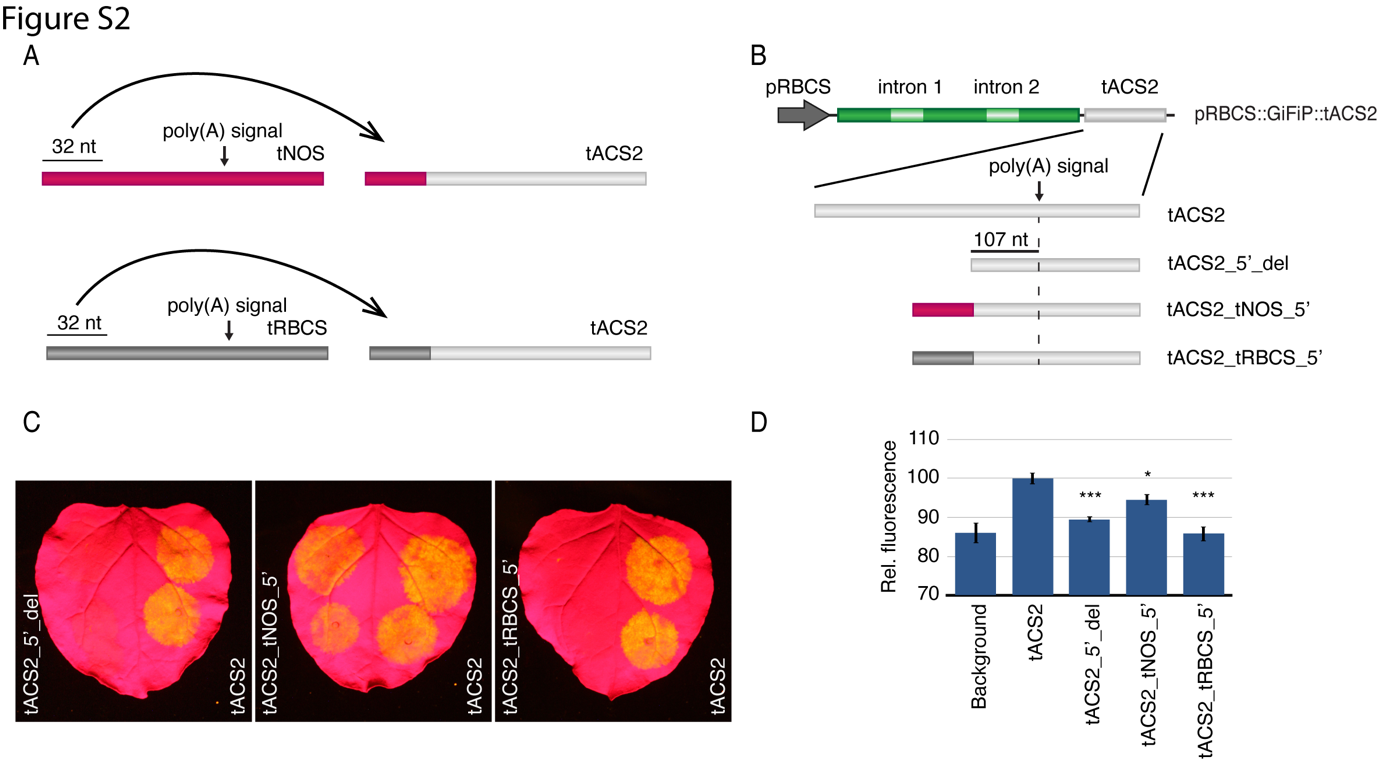
**

**Supplementary Figure 2. General impact on gene expression caused by modifications of the tACS2 5’ end.** Experiment design **(A)** and constructs **(B)** to test the general impact on transgene expression resulting from modifications in the 5’ termini of the *ACS2* terminator. **(C)** *N. benthamiana* agroinfiltrated leaves illustrating the effects of the different modifications on GFP expression. **(D)** The relative fluorescence of 11 infiltration spots (two per leaf, three leaves per plant, two plants per construct) with their respective standard error bars is shown. The Mann-Whitney U test was used to calculate the statistical significance of the differences among wild-type and mutant lines and is indicated by “*” (p ≤ 0.05) or “***” (p ≤ 0.001).

**
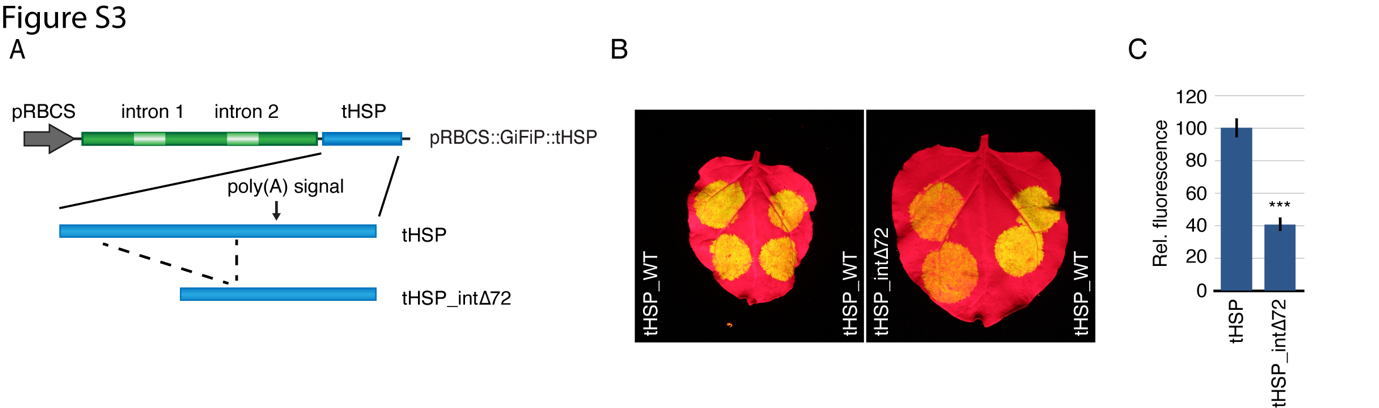
**

**Supplementary Figure 3. Effect of deleting a middle section of the tHSP on the terminator efficiency. (A)** Illustration of the constructs used to analyze the importance of a middle section on the tHSP function. The GFP phenotype **(B)** and relative fluorescence of 10 infiltration spots **(C)** are shown (two spots per leaf, one leaf per plant, five plants in total). P-value (p ≤ 0.001; indicated by “***”) obtained using the Mann-Whitney U test.


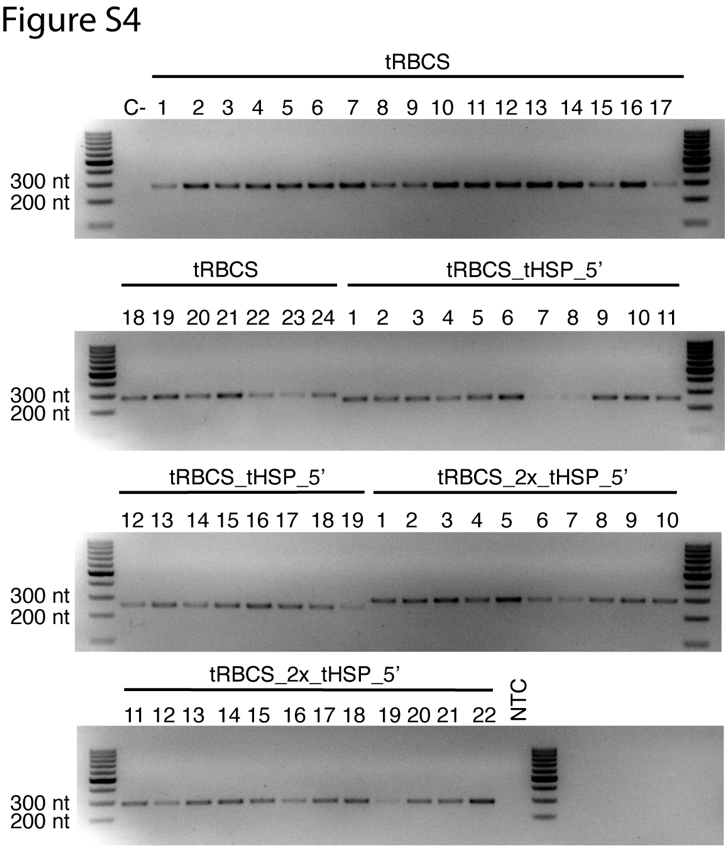


**Supplementary Figure 4. Genotyping of stably transformed *A. thaliana*.** PCR confirming the presence of the respective transgene in each of the plants analysed. C- refers to an unrelated transgenic plant and NTC (no template control) to a reaction without DNA.


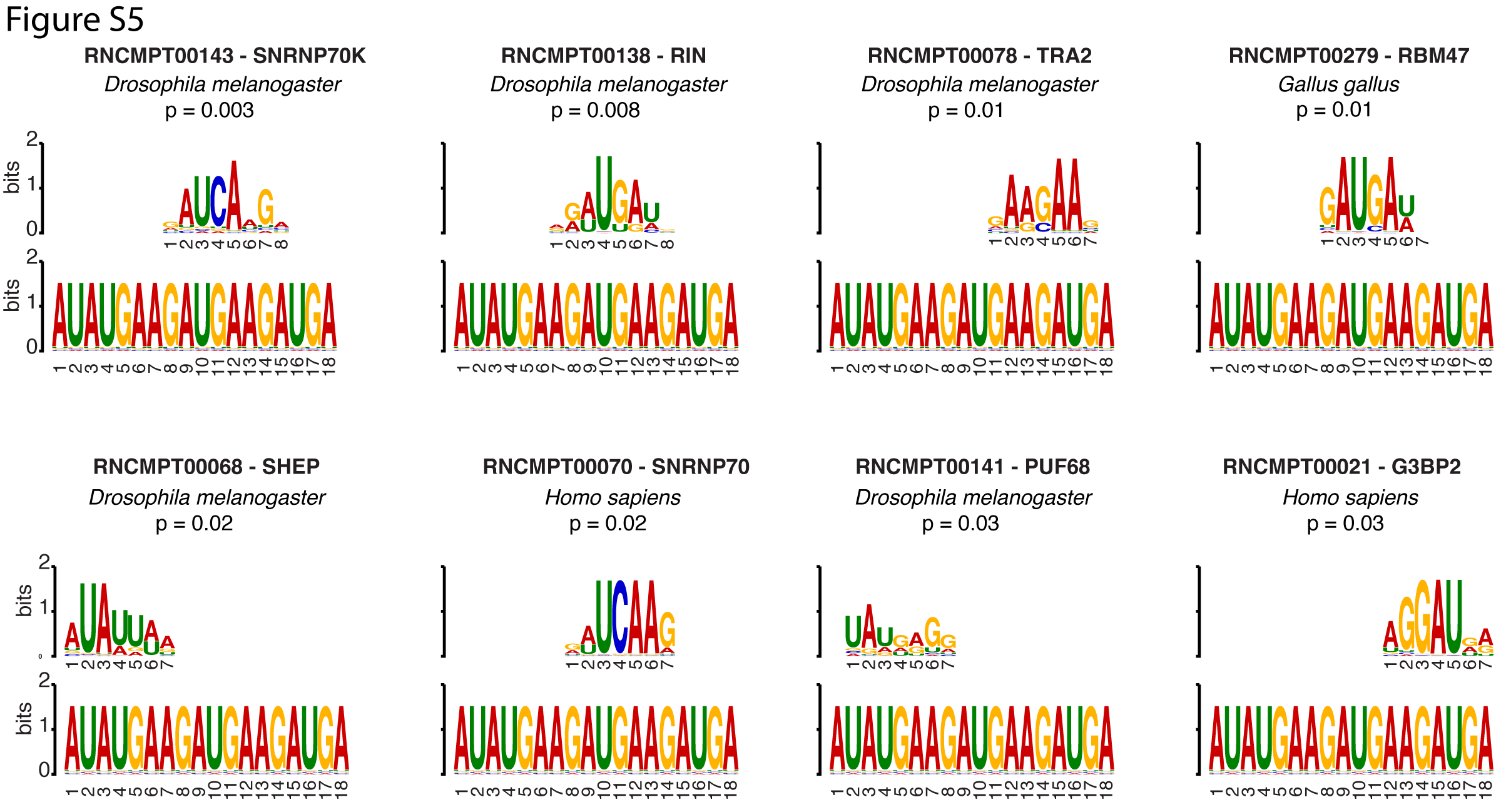


**Supplementary Figure 5. Search for conserved motifs contained in the tHSP 5’ fragment.** The first 18 nt of the tHSP 5’ termini (bottom sequence) was used as a template in a search for conserved binding motif (upper sequence) using Tomtom (Gupta et al., Genome Biology, 8(2): R24, 2007). For each predicted binding motif the following information is given: the conserved sequence with the frequency that each nucleotide appears, alignment of this sequence to the query fragment, protein name and database code, species of origin and statistical significance of the prediction (p = value).


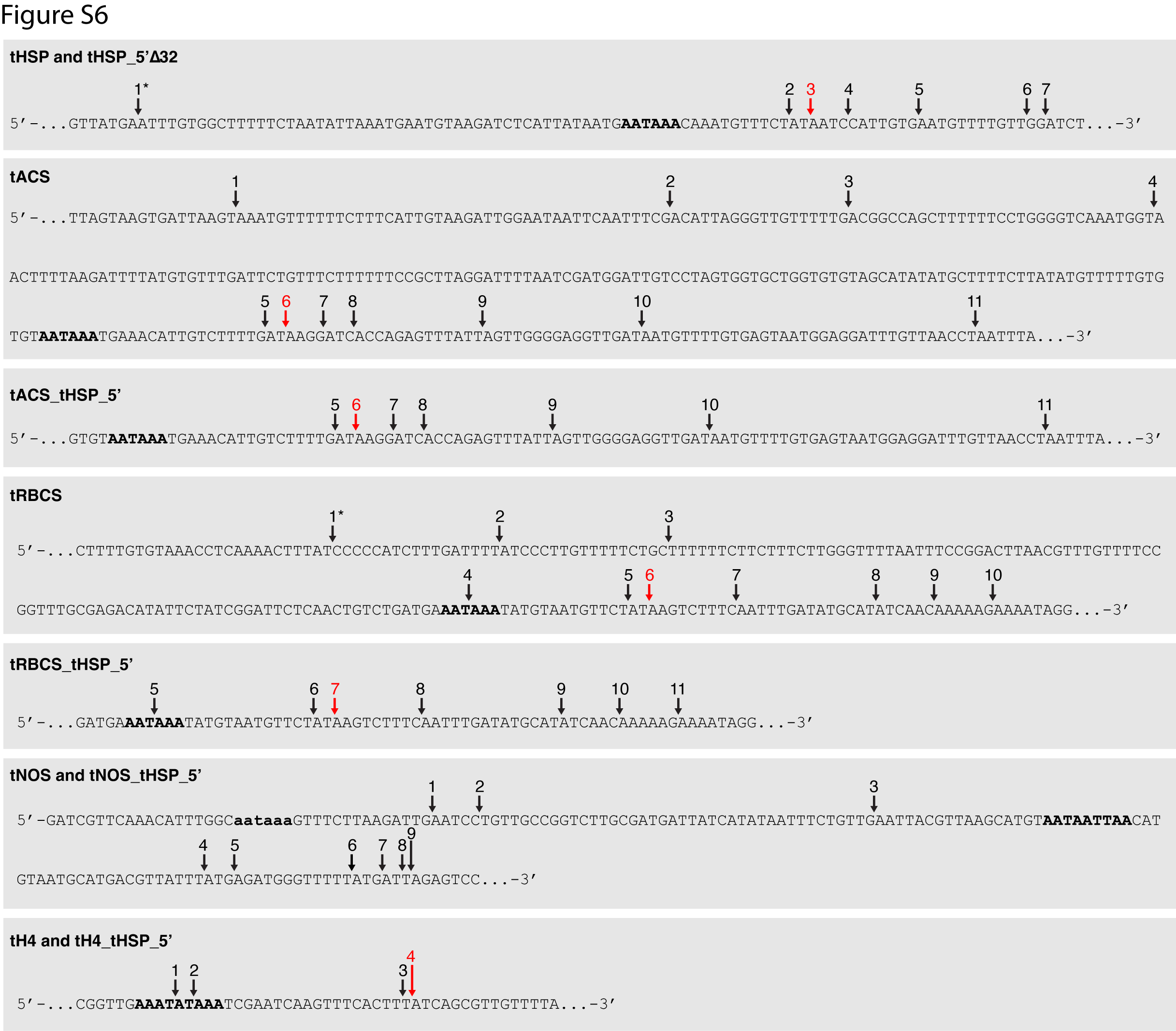


**Supplementary Figure 6. Location of the poly(A) sites in transcripts using wild-type and chimeric terminators.** The sequence of the mRNA 3’ end region containing the location of all mapped poly(A) sites. Numbered arrows refer to the different positions where a cleavage site (CS) was identified. Red arrows indicate the dominant poly(A) site, when applicable. Numbered arrows with a “*” point for CS where no poly(A) tail was detected. This figure complements the data shown in figure 6A.

**
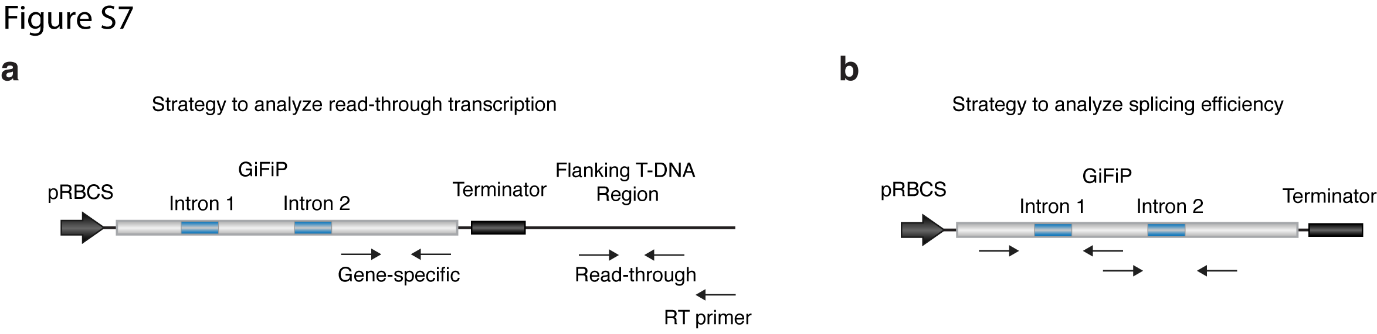
**

**Supplementary Figure 7. Strategy used to detect read-through transcription and splicing efficiency. (A)** Scheme showing the locations of primers used to detect the levels of transcriptional read-through coming from the reporter gene. **(B)** Splicing efficiency was assessed using primers flanking each one of the introns present in the GiFiP reporter gene.
